# A Series of Crystallographically Characterized Linear and Branched σ-Alkane Complexes of Rhodium: From Propane to 3-Methylpentane

**DOI:** 10.1021/jacs.1c00738

**Published:** 2021-03-26

**Authors:** Alexander J. Bukvic, Arron L. Burnage, Graham J. Tizzard, Antonio J. Martínez-Martínez, Alasdair I. McKay, Nicholas H. Rees, Bengt E. Tegner, Tobias Krämer, Heather Fish, Mark R. Warren, Simon J. Coles, Stuart A. Macgregor, Andrew S. Weller

**Affiliations:** † Department of Chemistry, University of York, Heslington, York YO10 5DD, U.K.; ‡ Department of Chemistry, Chemistry Research Laboratories, University of Oxford, Oxford OX1 3TA, U.K.; § Institute of Chemical Sciences, Heriot-Watt University, Edinburgh EH14 4AS. U.K.; ∥ UK National Crystallography Service, University of Southampton, Highfield, Southampton SO17 1BJ, U.K.; ⊥ Diamond Light Source Ltd., Diamond House, Harwell Science and Innovation Campus, Didcot OX11 0DE, U.K.

## Abstract

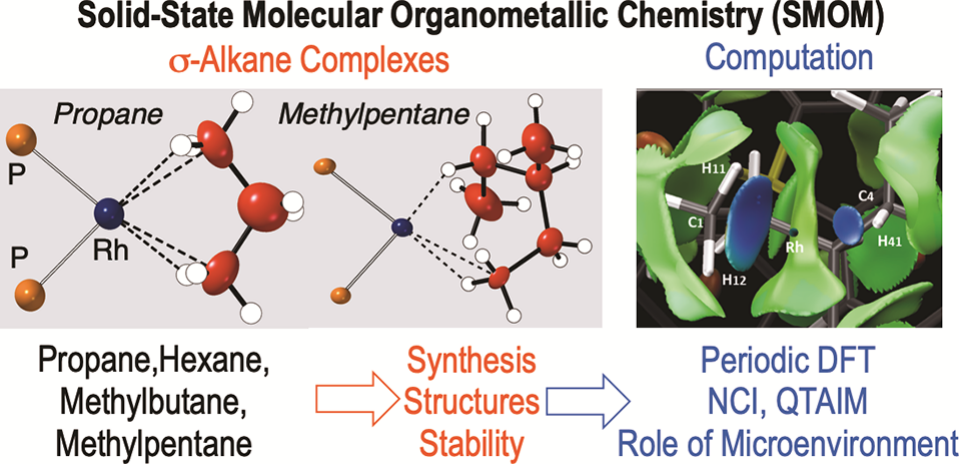

Using solid-state molecular organometallic (SMOM) techniques, in particular solid/gas single-crystal to single-crystal reactivity, a series of σ-alkane complexes of the general formula [Rh(Cy_2_PCH_2_CH_2_PCy_2_)(η^*n*^:η^*m*^-alkane)][BAr^F^_4_] have been prepared (alkane = propane, 2-methylbutane, hexane, 3-methylpentane; Ar^F^ = 3,5-(CF_3_)_2_C_6_H_3_). These new complexes have been characterized using single crystal X-ray diffraction, solid-state NMR spectroscopy and DFT computational techniques and present a variety of Rh(I)···H–C binding motifs at the metal coordination site: 1,2-η^2^:η^2^ (2-methylbutane), 1,3-η^2^:η^2^ (propane), 2,4-η^2^:η^2^ (hexane), and 1,4-η^1^:η^2^ (3-methylpentane). For the linear alkanes propane and hexane, some additional Rh(I)···H–C interactions with the geminal C–H bonds are also evident. The stability of these complexes with respect to alkane loss in the solid state varies with the identity of the alkane: from propane that decomposes rapidly at 295 K to 2-methylbutane that is stable and instead undergoes an acceptorless dehydrogenation to form a bound alkene complex. In each case the alkane sits in a binding pocket defined by the {Rh(Cy_2_PCH_2_CH_2_PCy_2_)}^+^ fragment and the surrounding array of [BAr^F^_4_]^−^ anions. For the propane complex, a small alkane binding energy, driven in part by a lack of stabilizing short contacts with the surrounding anions, correlates with the fleeting stability of this species. 2-Methylbutane forms more short contacts within the binding pocket, and as a result the complex is considerably more stable. However, the complex of the larger 3-methylpentane ligand shows lower stability. Empirically, there therefore appears to be an optimal fit between the size and shape of the alkane and overall stability. Such observations are related to guest/host interactions in solution supramolecular chemistry and the holistic role of 1°, 2°, and 3° environments in metalloenzymes.

## 1 Introduction

**Chart 1:**
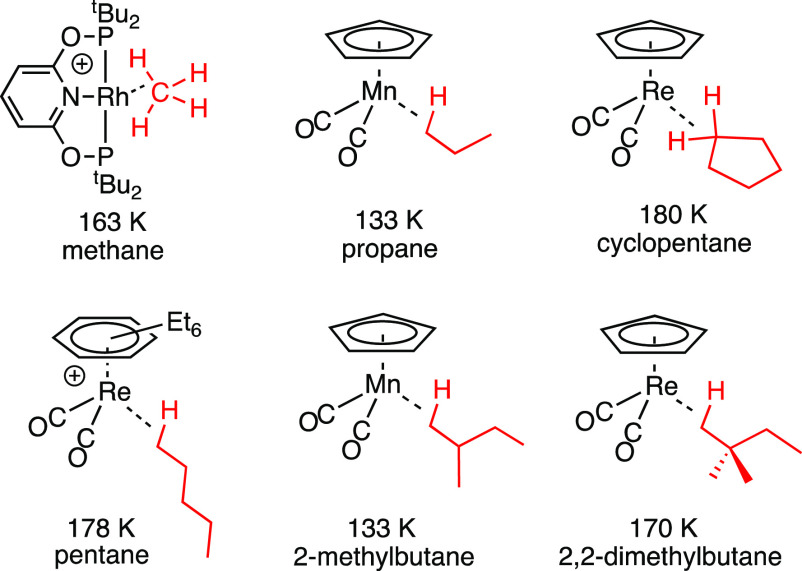
Examples of σ-Alkane Complexes Characterized Using *In Situ* NMR Techniquesa

The coordination of alkanes with metal centers and their subsequent C–H activation are central to chemical transformations that add value to these simple feedstocks,^1^ such as dehydrogenation,^2,3^ C–H functionalization,^4,5^ and isotopic enrichment through H/D exchange.^6^ Coordination with a metal center prior to C–H bond cleavage occurs through 3c-2e [M]···H–C interactions,^7^ forming so-called σ-complexes.^8^ For alkanes, such complexes are challenging to generate and observe under standard laboratory conditions. This is because the strong, nonpolar, and relatively sterically congested C–H bonds make alkanes very poor ligands—binding to metal centers often with bond enthalpies of 15 kcal/mol or less.^9^ In solution such complexes have only been observed using low-temperature *in situ* NMR spectroscopy (lifetimes of minutes),^10^ or on very short time scales (lifetimes of microseconds to seconds) using time-resolved infrared (TRIR)^11^ or XAFS techniques.^12^ These analyses are necessarily coupled with the generation of a vacant site on the metal center using ligand photoejection or protonation of a metal–alkyl bond. Using these methodologies, σ-alkane complexes from methane to dodecane have been generated.^13,14^Chart 1 shows examples characterized using NMR spectroscopy where methane,^15^ propane,^16^ cyclopentane,^17^ pentane,^18^ 2-methylbutane,^19^ and 2,2-dimethylbutane^20^ act as ligands.

a Anions are not shown.

These solution-based techniques provide unequivocal evidence for alkane coordination at a metal center. However, their use for the subsequent isolation of a crystalline material that allows for detailed structural characterization using single-crystal X-ray diffraction, or onward reactivity studies, has yet to be realized.^21,22^ This is because rapid alkane displacement by a solvent or a photogenerated ligand leads to lifetimes unsuitable for solution-based crystallization techniques, a situation compounded by the low temperatures used and less than 100% photoconversations achieved.

In response to this challenge, we have developed techniques where competing ligands (solvent or otherwise) and photogeneration of a vacant site are eliminated by using single-crystal to single-crystal (SC-SC^23^) solid/gas reactivity on molecular organometallic precursors.^24^ We term this solid-state molecular organometallic chemistry (SMOM).^25^

**Scheme 1:**
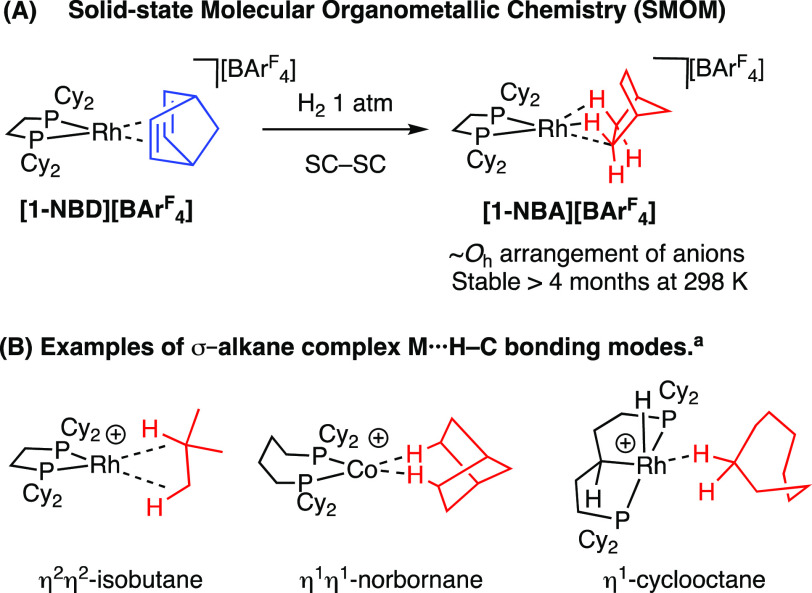
(A) The SMOM Synthetic Route and (B) Examples of Crystallography Characterized σ-Alkane Complexes (B) SC-SC = single crystal to single crystal. [BAr^F^_4_]^−^ anions not shown.

This approach is exemplified by addition of H_2_ to the simple precursor [Rh(Cy_2_PCH_2_CH_2_PCy_2_)(NBD)][BAr^F^_4_] (**[1-NBD][BAr**^**F**^_**4**_**]**), which results in the quantitative formation of [Rh(Cy_2_PCH_2_CH_2_PCy_2_)(NBA)][BAr^F^_4_] (**[1-NBA][BAr**^**F**^_**4**_**]**, NBD = norbornadiene, NBA = norbornane, Ar^F^ = 3,5-(CF_3_)_2_C_6_H_3_),^26^ (Scheme 1A). This σ-alkane complex is remarkably stable, surviving months at 298 K under an Ar atmosphere. The [BAr^F^_4_]^−^ anions play a key role in this stability, providing an approximately octahedral microenvironment around the cation, which supports the weak alkane binding with the metal center through multiple noncovalent interactions. This crystalline^27^ nanoreactor^28^ environment allows for long-range order to be retained, local coordinate flexibility at the reactive site, and hydrophobic pathways through the lattice from the CF_3_ groups.^29^ The retention of crystallinity also allows for detailed characterization by single-crystal X-ray diffraction and solid-state NMR spectroscopy (SSNMR).

When these structural and spectroscopic data are combined with an analysis of the electronic structures and noncovalent environment using periodic-DFT techniques,^30^ a detailed description of the bonding in these complexes is possible. For example, σ-alkane complexes have been characterized in which the alkane (e.g., isobutane) engages in two different η^2^:η^2^-C–H interactions with a Rh(I) center,^31^ η^1^:η^1^-NBA at a ^3^{Co(I)} center,^32^ or η^1^-cyclooctane with Rh(III) (Scheme 1B).^33^ The mobility and reactivity of the alkane ligand can also be studied using combined experimental and computational techniques: for example in H/D exchange,^31,34^ acceptorless dehydrogenation,^31^ and ligand substitution processes.^25^ A systematic variation of the ligand and anion^33,35,36^ can lead to systems that promote solid/gas SMOM catalysis: e.g., 1-butene isomerization under a continuous flow.^37^

**Scheme 2:**
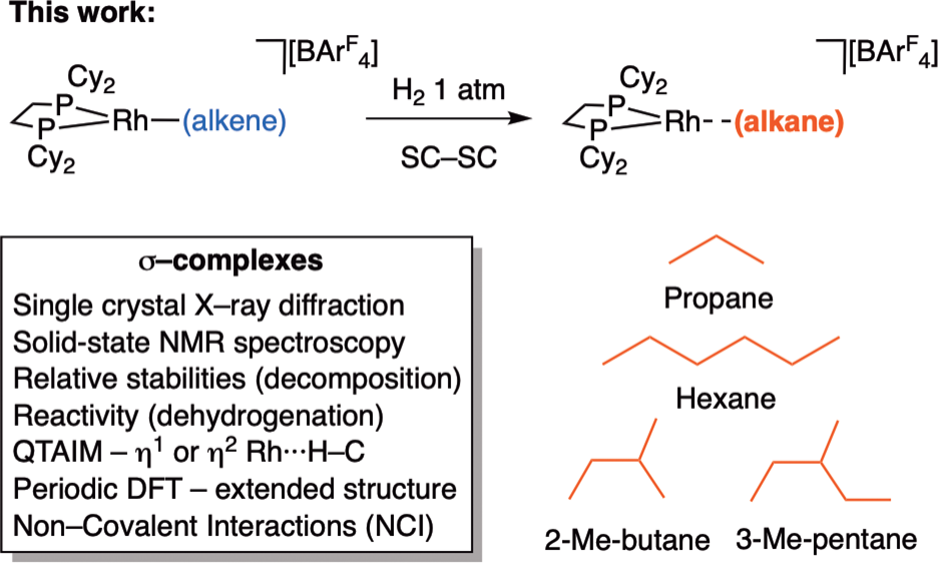
σ-Complexes Prepared in This Contribution Using SMOM Techniques

Despite these advances, a fundamental question is what are the limits of the SMOM methodology in terms of the smallest and largest alkane fragment that can be incorporated into the solid-state microenvironment provided by the [BAr^F^_4_]^−^ anions? Exploring this chemical space would provide structural data for the broadest set of σ-alkane complexes yet and also probe comparative reactivity and stability profiles. In this contribution we report the synthesis, structures, bonding, and reactivity of four new σ-alkane complexes of the [Rh(Cy_2_PCH_2_CH_2_PCy_2_)]^+^ fragment, ranging from propane to 3-methylpentane (Scheme 2). For one, a 2-methylbutane complex, a quantitative SC-SC acceptorless dehydrogenation occurs at room temperature—an endothermic process that normally requires high temperatures.^38^

## 2 Results and Discussion

### 2.1 Synthesis and Solid-State Structures Using SC-SC Techniques

#### 2.1.1 General Methodology

**Figure 1:**
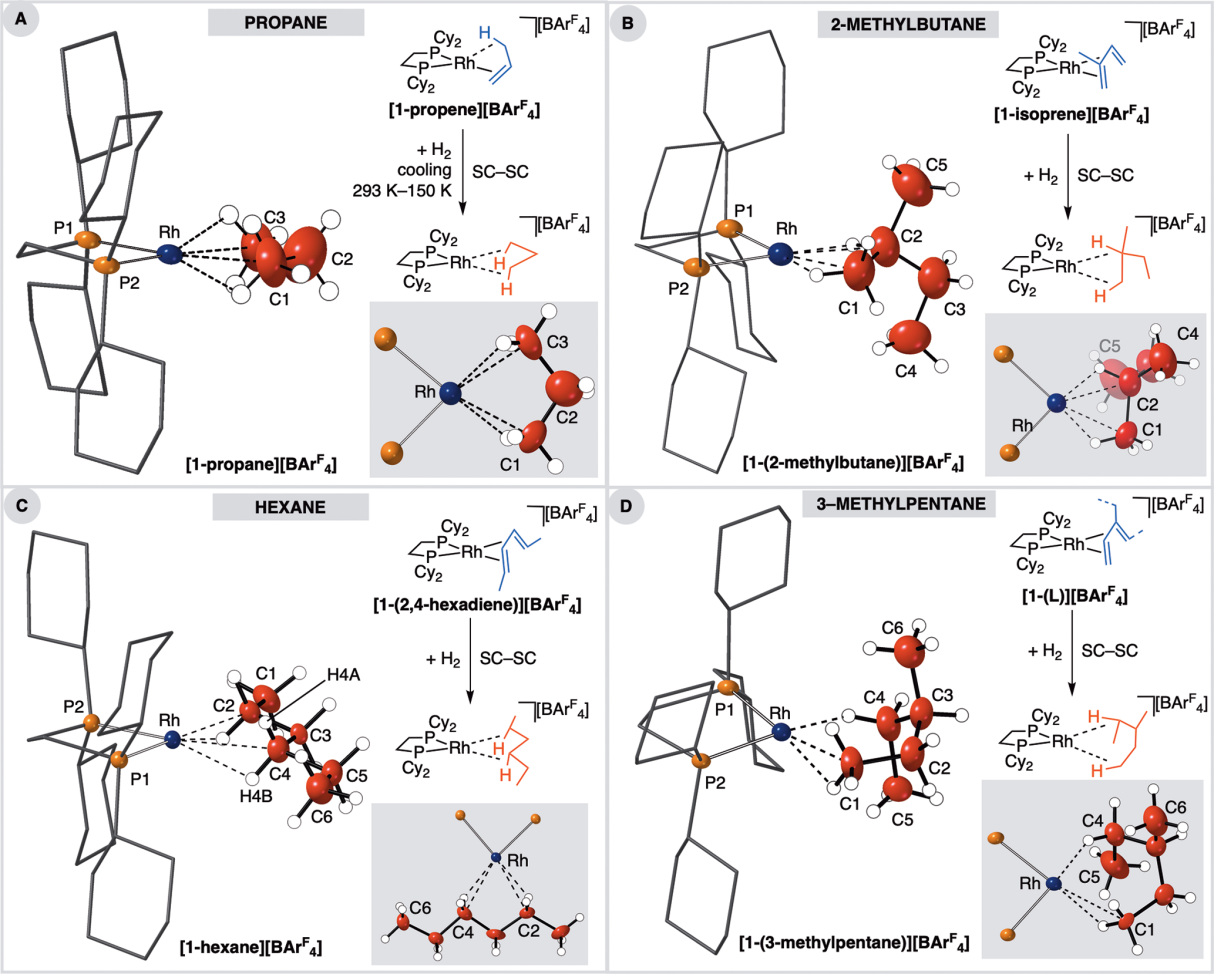
Solid-state molecular structures of the new cationic σ-alkane complexes (insets highlight Rh–alkane coordination) and synthetic details: (A) **[1-propane][BAr**^**F**^_**4**_**]**; (B) **[1-(2-methylbutane)][BAr**^**F**^_**4**_**]**; (C) **[1-hexane][BAr**^**F**^_**4**_**]**; (D) **[1-(3-methylpentane)][BAr**^**F**^_**4**_**]** (L = 2-methyl-1,3-pentadiene, 3-ethylbutadiene). Displacement ellipsoids are shown at the 30% probability level. Minor disordered components are not shown (see the Supporting Information). Table 1 gives selected bond lengths and angles.

Our synthetic methodology is one where 40–50 mg of the appropriate crystalline monoalkene or diene precursor [Rh(CyPCH_2_CH_2_PCy_2_)(alkene)][BAr^F^_4_] is treated with H_2_ (1 atm) between 3 and 25 min at 298 K (optimized). This results in formation of the alkane complex and a color change from orange (alkene) or cherry red (diene) to plum red (alkane complex). The rapid transfer of selected crystals to a precooled diffractometer allows for structural analysis at 150 K. In addition, an analysis of the bulk reaction sample was performed by ^31^P{^1^H} or ^13^C{^1^H} solid-state NMR spectroscopy (SSNMR) (section 2.2). This technique also allows for the relative stability toward decomposition by loss of alkane, or onward reactivity, of the σ-alkane complexes to be assessed at 298 K, by monitoring the evolution of the system with time. For complexes that are particularly sensitive to alkane loss and decomposition, synchrotron radiation at the Diamond Light Source (Beamline I19) was combined with a bespoke gas cell that allows for addition of H_2_ to a selected single crystal with concurrent cooling (see the Supporting Information). Figure 1 shows the structurally characterized σ-alkane complexes and the precursor alkene complexes used (which are fully described in the Supporting Information). Table 1 gives selected structural metrics.

**Table 1:** Selected Bond Lengths and Angles for the New σ-Alkane Complexes

complex	Rh···C/Å	Rh–P/Å	C–C/Å^a^	C–Rh–C/deg	PPRh–RhCC/deg^b^
**[1-propane][BAr**^**F**^_**4**_**]**	2.46(2), 2.45(2)	2.206(4), 2.226(6)	1.54(2), 1.52(2)	61.3(8)	3.7 (81.4^39^)
**[1-(2-methylbutane)][BAr**^**F**^_**4**_**]**	2.348(9), 2.39(1)	2.187(2), 2.185(2)	1.60(1), 1.51(2)	39.4(3)	5.3 (73.1)
**[1-hexane][BAr**^**F**^_**4**_**]**	2.527(3), 2.549(4)	2.2002(6), 2.1910(6)	1.511(5), 1.528(6)	58.5(1)	2.2 (83.7)
**[1-(3-methylpentane)][BAr**^**F**^_**4**_**]**	2.430(4), 2.788(6)	2.1959(7), 2.1965(7)	1.504(7), 1.475(8)	71.0(2)	16.5 (75.7)

a C–C distances associated with the hydrogenated alkene groups (Figure 1).

b Angle between planes defined by P1P2Rh and RhCC (σ-interaction). Angles in parentheses are for the equivalent measurement in the precursor alkene complexes.

#### 2.1.2 Ethane

**Chart 2:**
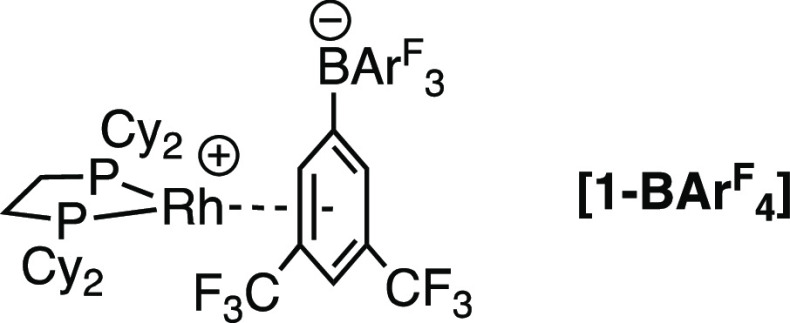
Structure of **[1-BAr^F^_4_]**

With selected single crystals of the bis-ethene complex [Rh(Cy_2_PCH_2_CH_2_PCy_2_)(η^2^-H_2_C=CH_2_)_2_][BAr^F^_4_] as the starting material,^25^ addition of H_2_ with concurrent cooling from 298 to 150 K *in situ* on the I19 Beamline resulted in loss of diffraction. While the ethene is likely hydrogenated to ethane under these conditions (*vide infra*), the subsequent σ-alkane complex is not sufficiently stable to allow for a structural determination. This suggests a lower size limit for σ-alkane complex formation using this metal/ligand/anion combination. The interaction of ethane with metal centers has been described using *in situ* solution NMR spectroscopy for Mn(η^5^-C_5_H_5_)(CO)_2_(ethane) (135 K)^19^ and [Rh(PONOP)(ethane)][BAr^F^_4_] (123 K)^40^ and *in situ* powder neutron diffraction for the MOF M_2_(dobdc) (M = Fe, Co; 4–10 K).^41−43^ In the bulk at 298 K the pale yellow anion-coordinated zwitterion **[1-BAr**^**F**^_**4**_**]**(26) (Chart 2) is formed immediately on addition of H_2_, as measured by ^31^P{^1^H} SSNMR. Ethane is also formed (gas-phase ^1^H NMR spectroscopy).

#### 2.1.3 Propane

The addition of H_2_ to the propene complex [Rh(Cy_2_PCH_2_CH_2_PCy_2_)(η^2^_π_:η^2^_C–H_-H_2_C=CHCH_3_)][BAr^F^_4_] (**[1-propene][BAr**^**F**^_**4**_**]**)^25,39^ was studied on selected single crystals *in situ* on the I19 Beamline. H_2_ was added while the sample was being cooled from 298 to 150 K. This allowed for rapid initial reaction with H_2_ and also slowed decomposition of the alkane complex, once formed. The resulting complex, [Rh(Cy_2_PCH_2_CH_2_PCy_2_)(H_3_CCH_2_CH_3_)][BAr^F^_4_] (**[1-propane][BAr**^**F**^_**4**_**]**), is stable enough when it is formed under these conditions to allow for a structural analysis at 150 K. In the bulk **[1-propane][BAr**^**F**^_**4**_**]** decomposes rapidly (30 min) at 298 K to give **[1-BAr**^**F**^_**4**_**]**, as measured by SSNMR (section 2.2)^44^ and shown visually by a color change from plum red to pale yellow. *Ex situ* hydrogenation strategies thus result in decomposition.

Figure 1A shows the solid-state structure of **[1-propane][BAr**^**F**^_**4**_**]**. The resulting structural refinement gives a satisfactory solution. While there is no disorder evident that would signal the presence of unreacted **[1-propene][BAr**^**F**^_**4**_**]**, we cannot rule out the presence of a small amount of this still being present in the unit cell, as indicated in the bulk by SSNMR (section 2.2). The formation of **[1-propane][BAr**^**F**^_**4**_**]** involves three consecutive SC-SC transformations on bulk materials (40–50 mg) starting from **[1-NBD][BAr**^**F**^_**4**_**]**(25,26) (eqs 1–3). This is reflected in the relatively high residual (*R*(2σ) = 10.5%) observed—a consequence of the falloff in high-angle data.
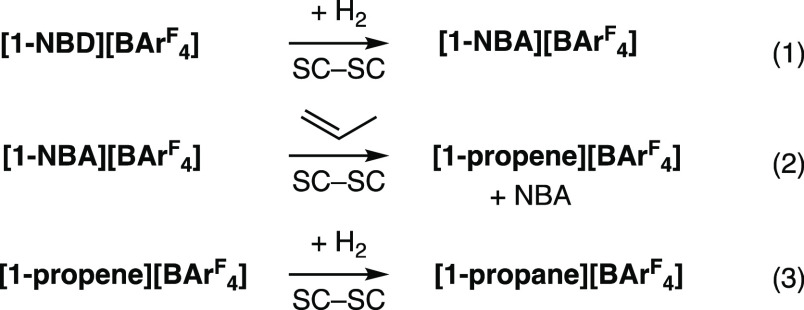
2

The generation of a σ-alkane complex in the single-crystalline sample is signaled by a change in the binding mode of the hydrocarbon: from propene (π-face/C–H agostic interaction) to one where the ligand now lies in the square plane of the Rh(I) center, ligated to the metal through two Rh···H–C σ-interactions. The C–C distances in the hydrocarbon are consistent with single bonds, and the Rh···C distances are ∼0.2 Å longer than in the starting propene complex (Table 1).^39^ The propane binds in a 1,3-motif: Rh···C 2.46(2), 2.45(2) Å (calculated 2.50 and 2.51 Å, section 2.3), and the central carbon (C2) is considerably farther away, being nonbonding (Rh···C2 2.99(3) Å), further signaling a change from the π-bound propene complex. Given the quality of the data, we cannot rule out that the propane binds slightly asymmetrically, as suggested by the DFT-calculated distances. With this caveat, the Rh···C1 and Rh···C3 distances sit in the range of those measured for other σ-alkane complexes (Table S2). For example, they are longer than in **[1-NBA][BAr**^**F**^_**4**_**]** (2.389(3) and 2.400(3) Å^26^) but shorter than in **[1-cyclohexane][BAr**^**F**^_**4**_**]** (2.62(2) and 2.53(3) Å^31^), which have 1,2- and 1,3-alkane binding motifs, respectively. They are considerably shorter than for d^6^-propane weakly bound to an open Fe site in the MOF Fe_2_(dobdc) (∼3 Å), as analyzed by powder neutron diffraction at 4 K.^41^ The 1,3-motif is similar to that proposed for propane coordination to a PdO(101) surface, albeit spanning two Pd sites.^45^ The propane σ-complex Mn(η^5^-C_5_H_5_)(CO)_2_(propane) has been characterized in solution using *in situ* NMR spectroscopy at low temperature (133 K).^16^ Given the quality of the refinement, hydrogen atoms were not located, and so the hapticity of the Rh···H–C interaction in **[1-propane][BAr**^**F**^_**4**_**]** (e.g., η^1^ or η^2^) was interrogated using computational techniques (section 2.3).

The ∼*O*_*h*_ environment of [BAr^F^_4_]^−^ anions, which is encoded^36^ in the propene starting complex,^25^ is retained in **[1-propane][BAr**^**F**^_**4**_**]** (Figure S49) and is very similar to that observed in **[1-NBA][BAr**^**F**^_**4**_**]** and **[1-cyclohexane][BAr**^**F**^_**4**_**]**. However, these two alkane complexes are considerably more stable than the propane congener with respect to the formation of **[1-BAr**^**F**^_**4**_**]**. **[1-NBA][BAr**^**F**^_**4**_**]** is indefinitely stable at 298 K, while **[1-cyclohexane][BAr**^**F**^_**4**_**]** undergoes acceptorless dehydrogenation over 16 h to give **[1-cyclohexadiene][BAr**^**F**^_**4**_**]**. This change in stability, despite similar Rh···C distances, likely reflects differences in the weak, multiple, stabilizing dispersive interactions between the alkane and the surrounding microenvironment provided in the solid state. These interactions are explored in more detail in section 2.3.

#### 2.1.4 Butane

Addition of H_2_ to the previously reported^25^ butadiene complex **[1-butadiene][BAr**^**F**^_**4**_**]** as a bulk single-crystalline material resulted in decomposition at 298 K to form **[1-BAr**^**F**^_**4**_**]**, as measured by ^31^P{^1^H} SSNMR spectroscopy.^44^ While the *in situ* addition to a selected crystal of **[1-butadiene][BAr**^**F**^_**4**_**]** on the I19 Beamline with simultaneous cooling to 150 K allowed for a structural refinement of the product, this was not of sufficient quality to unambiguously confirm whether a σ-alkane complex, or a partially hydrogenated Rh(III) metallocyclopentane intermediate, is formed. As found for **[1-propane][BAr**^**F**^_**4**_**]**, the relative stability of the targeted **[1-butane][BAr**^**F**^_**4**_**]** is considerably lower in comparison with other σ-alkane complexes with the same {Rh(L_2_)}^+^ fragment. This, again,^30,36^ hints at the importance of the stabilizing noncovalent interactions between the alkane and the secondary microenvironment, which is modified by changing the shape and size of the alkane ligand. This hypothesis is strengthened by noting that the previously reported *branched* isomer **[1-isobutane][BAr**^**F**^_**4**_**]**(31) (Scheme 1B) is stable at 298 K toward decomposition. Instead, this alkane complex undergoes acceptorless dehydrogenation over 4 h in an SC-SC transformation. M(η^5^-C_5_H_5_)(CO)_2_(butane) (M = Mn, Re) complexes have been generated by *in situ* NMR photochemical techniques in liquid butane at 136 K and have lifetimes of minutes at this temperature.^16^

#### 2.1.5 2-Methylbutane

Addition of H_2_ for 25 min to single crystals of the precursor diene complex **[1-isoprene][BAr**^**F**^_**4**_**]** forms [Rh(Cy_2_PCH_2_CH_2_PCy_2_){H_3_CCH(CH_3_)CH_2_CH_3_}][BAr^F^_4_] (**[1-(2-methylbutane)][BAr**^**F**^_**4**_**]**) in a SC–SC transformation (Figure 1B). While this complex is stable toward decomposition at 298 K in the solid state, it slowly loses H_2_ (6 h) in an SC-SC acceptorless dehydrogenation under an Ar-flow, similar to the closely related **[1-isobutane][BAr**^**F**^_**4**_**]**(31) (section 2.4).

The molecular structure of **[1-(2-methylbutane)][BAr**^**F**^_**4**_**]** demonstrates that the branched C5-alkane binds to the metal center in a 1,2-motif, via methyl (C1) and methine (C2) Rh···H–C interactions. All of the C–C bonds in the hydrocarbon fragment are in the range associated with C–C single bonds (Table 1 and Figure S51). The two Rh···C distances, 2.348(9) and 2.39(1) Å (calculated 2.38 and 2.48 Å, section 2.3), sit at the shorter end of the range observed with these Rh systems: e.g., **[1-NBA][BAr**^**F**^_**4**_**]**(26) (2.389(3) and 2.400(3) Å) and **[1-isobutane][BAr**^**F**^_**4**_**]**(31) (2.362(14) and 2.442(7) Å) (Table S2). The C–C angles around C2 sum to 328.6°, supporting the formation of an alkane ligand (sp^3^ hybridization) on hydrogenation. While the residual of *R*(2σ) = 9.6% may reflect a small amount of superpositionally disordered alkene in the unit cell, we were unable to sensibly model a secondary alkene fragment being present (Figure 5 shows the corresponding alkene structure that arises from acceptorless dehydrogenation of **[1-(2-methylbutane)][BAr**^**F**^_**4**_**]**). Solution trapping experiments (CD_2_Cl_2_) on the bulk sample immediately after hydrogenation recover 2-methylbutane with no evidence for residual alkene. However, we cannot discount the presence of a small amount of alkene complex in the unit cell of the analyzed sample that may contribute to these apparently shorter Rh···C distances.^46^ The hydrogen atoms were placed at calculated positions, and a full discussion of the bonding with the metal center is provided in section 2.3.

**Scheme 3:**
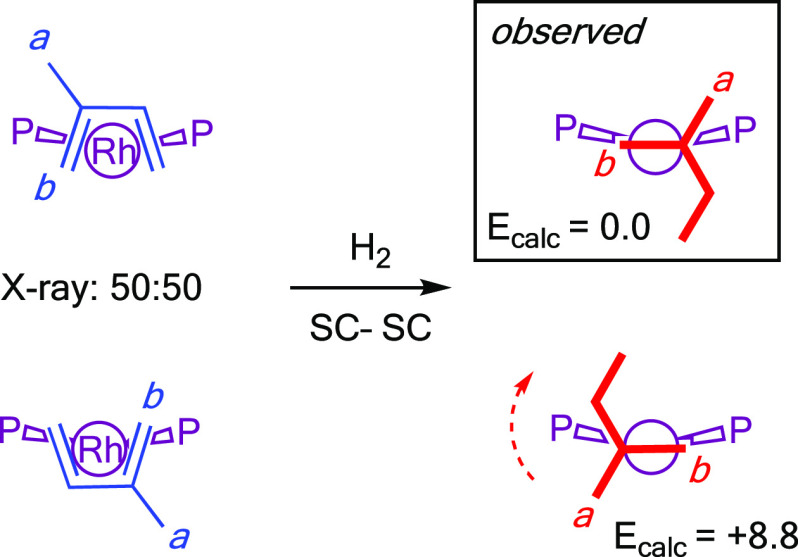
Suggested SC-SC Reorganization Process for [1-(2-methylbutane)][BAr^F^_4_] on Hydrogenation of **[1-isoprene][BAr^F^_4_]** Computed relative energies for the two rotamers in the solid state are given in kcal/mol.

The 2-methylbutane ligand is not disordered, which is in contrast with the precursor diene complex **[1-isoprene][BAr**^**F**^_**4**_**]**, which exists in the solid state as a 50:50 mixture of superpositionality-imposed orientations of the diene that are related by a noncrystallographically imposed *C*_2_ rotation (Scheme 3 and Figure S52). ^31^P{^1^H} SSNMR spectroscopy of **[1-(2-methylbutane)][BAr**^**F**^_**4**_**]** also confirms that a single isomer is formed in the bulk sample (section 2.2). As hydrogenation might be expected to initially form two different orientations of the bound 2-methylbutane ligand, we suggest that a relatively low energy reorganization of the alkane ligand is accessible to give the thermodynamically preferred orientation, which is both observed and computed in the solid state. This is likely a simple rotation. Low-energy fluxional processes for related σ-alkane complexes in the solid state have reported for NBA,^34^ pentane,^47^ and cyclohexane ligands.^31^ We cannot discount alternative mechanisms in which the stepwise hydrogenation accesses intermediates that result in a single isomer being favored.

**[1-(2-methylbutane)][BAr**^**F**^_**4**_**]** (and the closely related **[1-isobutane][BAr**^**F**^_**4**_**]**(31)) is stable toward alkane loss and formation of **[1-BAr**^**F**^_**4**_**]**, this being very different from the case for proposed **[1-butane][BAr**^**F**^_**4**_**]**. Again, we suggest that noncovalent interactions, which are provided by the ∼*O*_*h*_ microenvironment of [BAr^F^_4_]^−^ anions, play a significant part in this. When this influence of the secondary coordination environment is removed, the relative stabilities of related σ-complexes become leveled. For example Mn(η^5^-C_5_H_5_)(CO)_2_(butane)^16^ and Mn(η^5^-C_5_H_5_)(CO)_2_(isobutane)^19^ have very similar lifetimes of ∼5–6 min at ∼135 K in liquid alkane solvent.

#### 2.1.6 Hexane

The precursor to a σ-complex of hexane is the 2,4-hexadiene complex [Rh(Cy_2_PCH_2_CH_2_PCy_2_)(η^2^η^2^-H_3_CCH=CHCH=CHCH_3_)][BAr^F^_4_] (**[1-hexadiene][BAr**^**F**^_**4**_**]**) (Figure 1C and Figure S53). This complex is isolated in single-crystal form as the symmetric 2,4-isomer but in solution coexists in slow equilibrium with the 1,3-isomer (see the Supporting Information). This likely occurs through successive 1,3-hydride shifts^48^ via an allyl hydride intermediate. Addition of H_2_ to single crystals of **[1-hexadiene][BAr**^**F**^_**4**_**]** results in hydrogenation of the diene in a SC-SC transformation to form [Rh(Cy_2_PCH_2_CH_2_PCy_2_)(H_3_C(CH_2_)_4_CH_3_)][BAr^F^_4_] (**[1-hexane][BAr**^**F**^_**4**_**]**) (*R*(2σ) = 4.1%). Figure 1C shows the resulting structure determined from a single-crystal X-ray diffraction study. The hexane ligand binds in a 2,4-motif (i.e., the Rh···H–C interactions are separated by a methylene group as in [**1-propane][BAr**^**F**^_**4**_**]**), and all C–C distances in the hydrocarbon ligand are consistent with single bonds (C–C range 1.460(8)–1.55(2) Å). The Rh····C distances (2.527(3)/2.549(4) Å; calculated 2.54/2.62 Å, section 2.3) are within the range observed for σ-alkane complexes with {Rh(L_2_)}^+^ fragments (see Table S2). They are similar to those reported for **[1-pentane][BAr**^**F**^_**4**_**]**, 2.514(4) and 2.522(5) Å, which also binds in a 2,4-motif.^47^ The quality of the data was sufficient to locate and refine the hydrogen atoms associated with the Rh···H–C interactions (*R*(2σ) = 4.1%). These data suggest that both methylene C–H groups on each carbon are interacting with the metal center, although to differing degrees: i.e., Rh–H4B = 2.14(4) Å versus Rh–H4A = 2.46(A) Å. These interactions are analyzed in the computational section (section 2.3).

**Figure 2:**
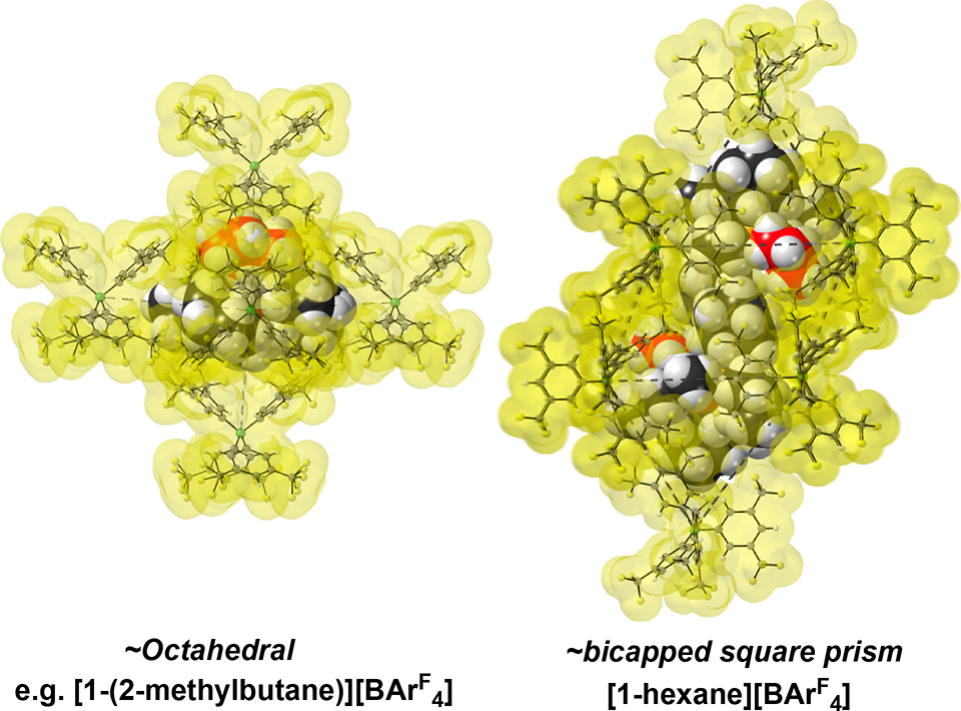
Packing diagrams of **[1-(2-methylbutane)][BAr**^**F**^_**4**_**]** and **[1-hexane][BAr**^**F**^_**4**_**]** (van der Waals radii) showing the different arrangements of the [BAr^F^_4_]^−^ anions.

In the solid state the [BAr^F^_4_]^−^ anions do not form an ∼*O*_h_ arrangement, this being different from the other complexes discussed here: e.g., **[1-(2-methylbutane)][BAr**^**F**^_**4**_**]**. Instead, a bicapped square prism (BCSP) of anions accommodates two [Rh]^+^ cations (Figure 2). This arrangement of anions has been observed before for **[1-pentane][BAr**^**F**^_**4**_**]**(47) and is encoded in the starting diene precursor (Figure S54).

While a hexane σ-complex has not been directly characterized in solution using NMR techniques, a related heptane complex, W(CO)_5_(heptane), has been observed using time-resolved XAFS.^12^ This allowed for the W···C distance to be modeled at 3.07(6) Å for the σ-interaction. This is considerably longer than in **[1-hexane][BAr**^**F**^_**4**_**]**, even given the difference in covalent radii between W and Rh (0.2 Å).^49^

#### 2.1.7 3-Methylpentane

The branched-hexane σ-alkane complex **[1-(3-methylpentane)][BAr**^**F**^_**4**_**]** is formed from a mixture of precursor isomeric alkene complexes **[1-(3-methyl-1,3-pentadiene)][BAr**^**F**^_**4**_**]** and **[1-(2-ethylbutadiene)][BAr**^**F**^_**4**_**]** (Figure 1D and Figure S55). In the solid state these cocrystallize as a superpositionally disordered 50:50 mixture, with an *O*_*h*_ arrangement of [BAr^F^_4_]^−^ anions. Addition of H_2_ to these single crystals results in an SC–SC transformation and the formation of a single isomer of the σ-complex **[1-(3-methylpentane)][BAr**^**F**^_**4**_**]** (Figure 1E). The resulting structural refinement was of good quality (*R*(2σ) = 4.8%) and shows the alkane to be interacting with the Rh center through methyl (Rh···C1 2.430(4) Å) and methylene (Rh···C4 2.788(6) Å) groups, in a 1,4-motif (calculated 2.46 and 2.89 Å, section 2.3). On the basis of these distances, the former is likely a η^2^ interaction, while the latter is considerably longer, suggesting η^1^ bonding.^33,50^ Hydrogen atoms were placed in calculated positions. The fine details of the bonding mode of the Rh···H–C interactions are discussed in section 2.3. The single isomer observed in **[1-(3-methylpentane)][BAr**^**F**^_**4**_**]** in the solid state suggests that a reorganization occurs on hydrogenation of the two isomeric dienes present in the precursors. As for **[1-(2-methylbutane)][BAr**^**F**^_**4**_**]**, this can be explained by a simple rotation of the bound alkane to form the thermodynamically preferred isomer (Δ*E*_calc_ = +2.5 kcal/mol). The stability of **[1-(3-methylpentane)][BAr**^**F**^_**4**_**]** is discussed in section 2.2.

#### 2.1.8 Octane

The precursor to a potential octane σ-alkane complex, **[1-(octa-2,4-diene)][BAr**^**F**^_**4**_**]**, was synthesized and structurally characterized (Figure S57). This structural analysis also showed that the [BAr^F^_4_]^−^ anions adopt the same ∼*O*_h_ arrangement as for many of the other precursors discussed here. However, addition of H_2_ resulted in an immediate loss of diffraction and the formation of pale yellow **[1-BAr**^**F**^_**4**_**]**, suggesting that octane is too large to support an SC-SC transformation in this [BAr^F^_4_]^−^ cavity. Thus, ethane and octane define lower and upper bounds, respectively, for currently accessible single-crystalline examples of σ-alkane complexes generated by SMOM techniques using this combination of a metal fragment and anion.

### 2.2 Relative Stabilities in the Solid State as Measured by Solid-State NMR Spectroscopy

**Scheme 4:**
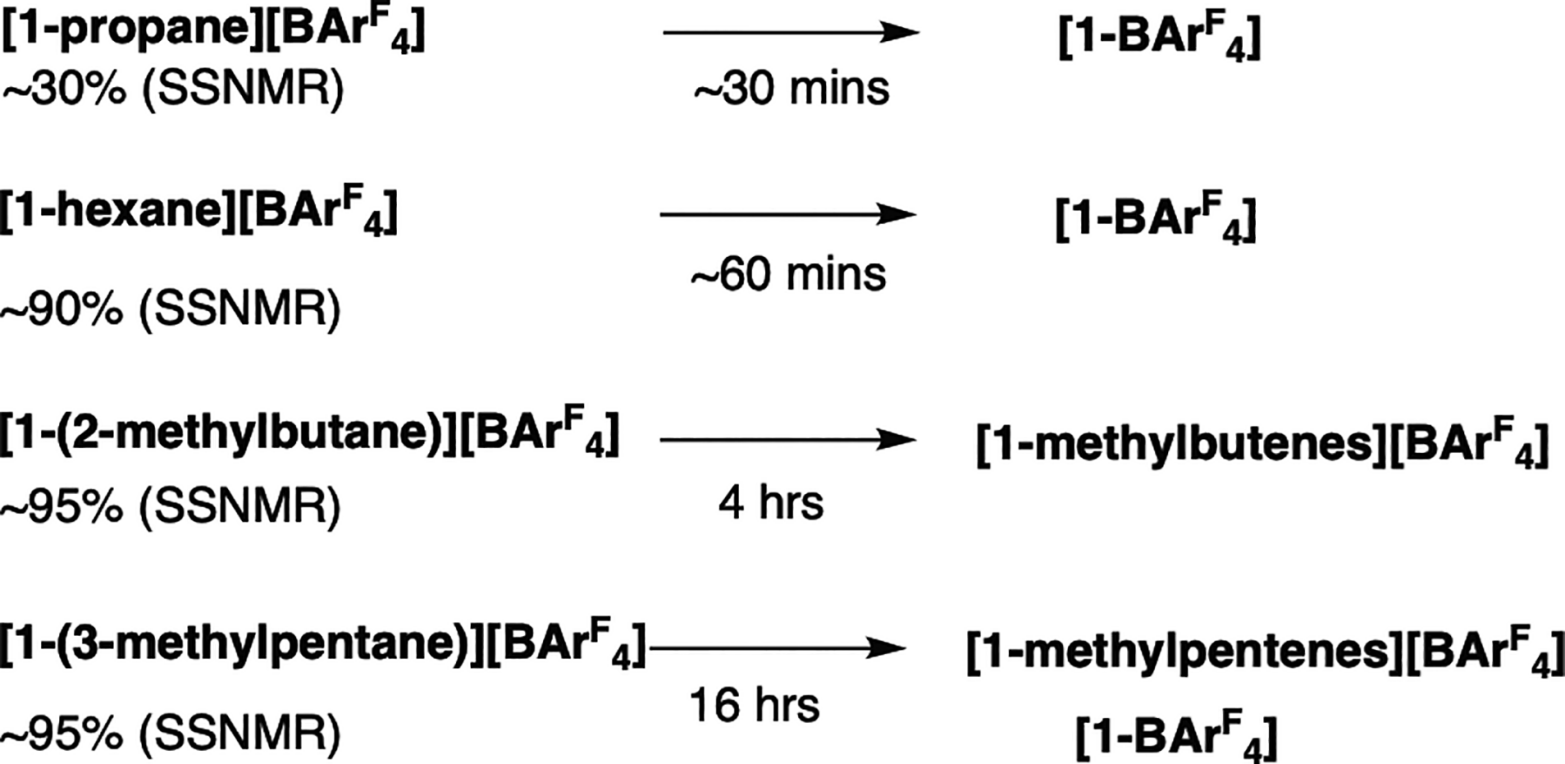
Relative Stabilities of the σ-Alkane Complexes as Bulk Solids (under Ambient Conditions)

In addition to characterization of the new σ-alkane complexes using single-crystal X-ray diffraction, ^31^P{^1^H} and ^13^C{^1^H} SSNMR spectroscopy was used to characterize the reaction in the bulk and assess their relative stabilities (Scheme 4). For all of the systems reported here a diagnostic downfield shift is observed in the ^31^P{^1^H} SSNMR spectrum on formation of the σ-alkane complex (δ 102–110) from the alkene precursor (δ 74–90). There is an increase in the *J*(RhP) coupling constant on forming the σ-alkane complex, consistent with a more weakly bound *trans* alkane ligand, i.e. 152–182 to 188–236 Hz, respectively. In the ^13^C{^1^H} SSNMR spectrum signals due to the coordinated alkene in the precursor (100–50 ppm) disappear on hydrogenation. The decomposition product **[1-BAr**^**F**^_**4**_**]** is observed as a very broad signal, indicating loss of crystallinity, at δ ∼88.

The propane σ-complex is so unstable at room temperature that on hydrogenation of the bulk precursor in situ only ∼30% of the target alkane complexes is initially observed at 294 K. In addition to **[1-propane][BAr**^**F**^_**4**_**]** a small amount of unreacted **[1-propene][BAr**^**F**^_**4**_**]** is observed (∼10%), and the remainder is **[1-BAr**^**F**^_**4**_**]**. After 30 min at 294 K there is no **[1-propane][BAr**^**F**^_**4**_**]** observed. Thus, characterization rests on the *in situ* analysis of a single crystal on the I19 beamline. **[1-hexane][BAr**^**F**^_**4**_**]** is relatively more stable toward decomposition, being formed in ∼90% spectroscopic yield and decomposing over 1 h to form **[1-BAr**^**F**^_**4**_**]**. This allows for good ^31^P{^1^H} and ^13^C{^1^H} SSNMR NMR spectral data to be recorded (Figures S28 and S29). **[1-pentane][BAr**^**F**^_**4**_**]** decomposes over a comparable time scale (∼4 h).^47^

Once synthesized, **[1-(2-methylbutane)][BAr**^**F**^_**4**_**]** is stable toward decomposition to form amorphous **[1-BAr**^**F**^_**4**_**]**, although a small amount (∼10–20%) is formed during the synthesis that arises from over-hydrogenation, as we have commented on before.^31^ Instead, an acceptorless SC-SC dehydrogenation occurs to form **[1-(methylbutenes)][BAr**^**F**^_**4**_**]** that is described in more detail in section 2.4. This means that a small amount (∼10%) of **[1-(methylbutenes)][BAr**^**F**^_**4**_**]** is also observed in the first NMR spectrum that is taken after 10 min post H_2_ addition. In contrast, **[1-(3-methylpentane)][BAr**^**F**^_**4**_**]** is not as stable in the solid state at 298 K. Although a σ-alkane complex is initially formed on hydrogenation (Figure 1D and Figure S38 and S39), this changes over 16 h (Figure S40) to form a mixture of dehydrogenated methylpentene complexes and **[1-BAr**^**F**^_**4**_**]** (section 2.4). Despite this, in comparison to **[1-hexane][BAr**^**F**^_**4**_**]**, **1-(3-methylpentane)][BAr**^**F**^_**4**_**]** is considerably more stable—likely a consequence of the branched alkane structure that modifies interactions with the anion microenvironment, and the different motifs of anions (Figure 2).

The stability of any particular alkane σ-complex toward decomposition is likely to be strongly influenced by a combination of the primary coordination sphere interactions (i.e., the strength of the Rh···H–C bonds), stabilizing or destabilizing interactions from the secondary microenvironment, and differences in the tertiary, periodic, crystal structure. To probe both the intimate interactions of the alkane with the Rh(I) centers and the influence of the wider secondary microenvironment, we turned to a computational analysis of these new systems, as well as a comparison with those previously reported. We initially discuss the primary coordination sphere around the metal centers, which provides a baseline for the subsequent analysis of the influence of the wider environment.

### 2.3 Computational Studies on the Primary and Secondary Coordination Spheres

Further insights into the structure and stability of the Rh σ-alkane complexes were provided by periodic DFT calculations and electronic structure analyses. The latter were based on the fully optimized solid-state structures rather than the crystallographic data, and this choice was prompted by the experimental uncertainties in some of the alkane atom positions, notably in **[1-propane][BAr**^**F**^_**4**_**]**. For the other complexes the observed and fully optimized structures provided very similar data, and both sets of results are compared in the Supporting Information. In the following discussion we first assess the intramolecular Rh···H–C σ-interactions, before probing the effect of the extended solid-state environment on the stability of both the Rh σ-alkane complexes reported here and related complexes from previous studies.

#### 2.3.1 Computational Characterization of σ-Alkane Hapticities

**Figure 3:**
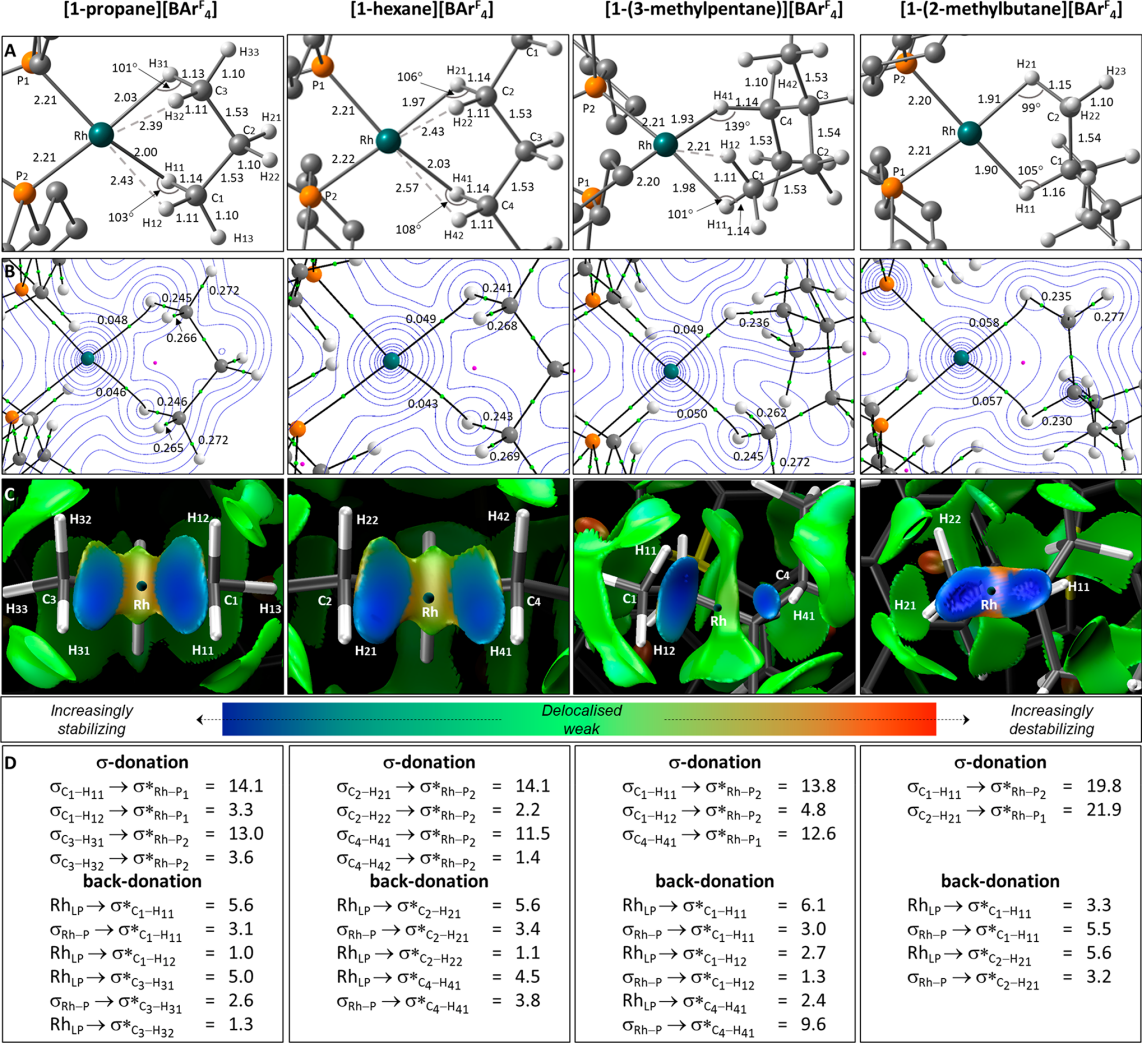
(A) Computed structure of the **[1-alkane]**^**+**^ cations highlighting key distances (Å) and angles to H atoms. Non-alkane H atoms are omitted for clarity. (B) QTAIM molecular graphs with bond critical bonds (BCPs) in green, ring critical points (RCPs) in pink, and selected BCP electron densities, ρ(*r*), in au. Contour plots are in the plane containing Rh and the two H atoms involved in the σ-interactions. (C) Detail of the NCI plots viewed from the Rh center looking down an axis passing through the center of the alkane moiety interacting with Rh. Isosurfaces are generated for σ = 0.3 au and −0.07 < ρ < 0.07 au, and a key showing the color scheme employed is also provided. (D) Major donor–acceptor interactions derived from a second-order perturbation NBO analysis (kcal/mol). Values are the sum of each type of donor–acceptor interaction (e.g., there are up to four Rh_LP_→σ*_C–H_ and two σ_Rh–P_→σ*_C–H_ donations; see the Supporting Information for full details).

The alkane σ-complexes characterized here present three different binding motifs: 1,3-binding (**[1-propane][BAr**^**F**^_**4**_**]**) and its equivalent 2,4-binding (**[1-hexane][BAr**^**F**^_**4**_**]**), 1,4-binding (**[1-(3-methylpentane)][BAr**^**F**^_**4**_**]**), and 1,2-binding (**[1-(2-methylbutane)][BAr**^**F**^_**4**_**]**). The computed structures of the **[1-propane]**^**+**^, **[1-hexane]**^**+**^, **[1-(3-methylpentane)]**^+^ and **[1-(2-methylbutane)]**^**+**^ cations are shown in Figure 3A along with the results of the quantum theory of atoms in molecules (QTAIM), noncovalent interaction (NCI) and natural bond orbital (NBO) analyses in Figure 3B–D, respectively.

The computed structure of **[1-propane]**^**+**^ shows two short Rh···H contacts (Rh···H_11_ 2.03 Å; Rh···H_31_ 2.00 Å) and slightly elongated C_1_–H_11_ and C_3_–H_31_ bonds (1.13/1.14 Å) that are indicative of two Rh→H–C σ-interactions. These are confirmed by the presence of Rh···H_11_ and Rh···H_31_ bond paths in the QTAIM analysis that feature bond critical point (BCP) electron densities, ρ(*r*), of 0.046 and 0.048 au, respectively. The C_1_–H_11_/C_3_–H_31_ BCPs also exhibit reduced ρ(*r*) values of ca. 0.246 au, consistent with σ-donation to Rh (cf. the spectator C_1_–H_13_/C_3_–H_33_ bonds 1.10 Å; ρ(*r*) = 0.272 au). We have previously found NCI plots to be a good indicator of C–H bond hapticity.^33^ In this case the stabilizing blue features between the Rh center and the alkane that span both the C_1_–H_11_ and C_3_–H_31_ bonds suggest an η^2^_C–H_ binding mode. This is confirmed by NBO calculations that quantify σ-donation from the C_1_–H_11_ and C_3_–H_31_ bonds at 14.1 and 13.0 kcal/mol, respectively. This σ-donation is supported by total back-donations of 8.7 and 7.6 kcal/mol, respectively. An inspection of the Rh_LP_→σ*_C–H_ back-donation confirms that this is dominated by π-character (Figures S64 and S66). An η^2^_C–H_ binding mode is also consistent with Rh–H–C angles of ca. 102°:^33,50^ i.e., a “closed” M···H–C interaction.^51^

In addition to these η^2^_C–H_ interactions, some contribution from the geminal C_1_–H_12_ and C_3_–H_32_ bonds is also evident. Rh···H_12_ and Rh···H_32_ contacts of 2.39 and 2.43 Å are computed, as well as C–H distances and BCP ρ(*r*) values that are intermediate between those of the C_1_–H_11_/C_3_–H_31_ and C_1_–H_13_/C_3_–H_33_ pairs. Although QTAIM does not identify Rh···H_12_ or Rh···H_32_ bond paths, the NCI plot does show extension of the stabilizing blue features over the C_1_–H_12_ and C_3_–H_32_ bonds.^52^ An NBO analysis also identifies σ-donation from each bond (C_1_–H_12_→Rh = 3.3 kcal/mol; C_3_–H_32_→Rh = 3.6 kcal/mol) supported by weak back-donation in each case of ca. 1.0 kcal/mol. Overall, these data suggest that the dominant η^2^_C1–H11_ and η^2^_C3–H31_ σ-interactions are supported by additional stabilization from the geminal C_1_–H_12_ and C_3_–H_32_ bonds. Thus, propane binds to Rh through two {CH_2_}→Rh interactions that lie along the η^2^_C–H_ to η^2^:η^2^_C–H_ continuum.^13,50,53^ An analysis of the **[1-hexane]**^**+**^ cation indicates that a very similar situation pertains, although the supporting geminal interactions are now somewhat weaker.

The C_1_–H_11_→Rh σ-interactions in the **[1-(3-methylpentane)]**^**+**^ cation can be interpreted in a similar way. An NBO analysis suggests comparable σ-donation (13.8 kcal/mol), but unusually the degree of back-donation (9.1 kcal/mol) now approaches that of the σ-donation. The major Rh_LP_→σ*_C–H_ components exhibit π-character (Table S20), and this relatively strong back-donation may be a feature of the wide bite angle of the 1,4-alkane binding mode that is observed here for the first time as the thermodynamically preferred structure.^47^ σ-Donation from the geminal C_1_–H_12_ bonds is also somewhat larger in **[1-(3-methylpentane)]**^**+**^ (4.8 kcal/mol) than in **[1-propane]**^**+**^ (ca. 3.5 kcal/mol). In contrast, the C_4_–H_41_→Rh interaction in **[1-(3-methylpentane)]**^**+**^ is markedly different and exhibits an η^1^_C–H_ binding mode. This is most evident in the NCI plot, which shows a localized blue disk along the Rh···H_41_ vector. The degree of σ-donation is close to that of the η^2^_C1–H11_ interaction (12.6 kcal/mol), and a similar degree of back-donation is also found (12.0 kcal/mol). However, in this case back-donation is dominated by σ-donation from the occupied σ_Rh–P_ orbitals into σ*_C–H_ and this reflects a more end-on approach of the C_4_–H_41_ bond to the Rh center. The different η^2^_C–H_ and η^1^_C–H_ binding modes are reflected in RhC_1_H_11_ and RhC_4_H_41_ angles of 101 and 139°, respectively.

The 1,2-bound alkane ligand in **[1-(2-methylbutane)][BAr**^**F**^_**4**_**]** exhibits two chemically distinct C–H→Rh σ-interactions involving 1° and 3° C–H bonds. Both exhibit an η^2^_C–H_ hapticity with NBO indicating that the 1° C_2_–H_21_→Rh interaction is marginally the stronger of the two. In this case the orientation of the C_2_–H_22_ bond rules out any additional geminal stabilization and this is reflected in the NCI plot, where the blue stabilizing region runs parallel to the C_2_–H_21_ bond without extending toward H_22_.

#### 2.3.2 Comparison with Related σ-Alkane Complexes

Previously, we have reported the structures of **[1-isobutane][BAr**^**F**^_**4**_**]**,^31^**[1-NBA][BAr**^**F**^_**4**_**]**,^26^**[1-pentane][BAr**^**F**^_**4**_**]**,^47^ and **[1-cyclohexane][BAr**^**F**^_**4**_**]**.^31^ The alkane ligands in the **[1-isobutane]**^**+**^ and **[1-NBA]**^**+**^ cations both exhibit a 1,2-binding mode that closely resembles that of the 2-methylbutane ligand in **[1-(2-methylbutane)]**^**+**^. Moreover the 2,4-binding mode of pentane in **[1-pentane]**^**+**^ has features similar to those of the alkane ligands in **[1-propane]**^**+**^ and **[1-hexane]**^**+**^. These last three linear alkanes all lie parallel to the {RhP_2_} coordination plane. In contrast, the cyclohexane ligand in **[1-cyclohexane]**^**+**^ sits perpendicular to this plane and this results in a binding mode that is best described as intermediate between η^2^_C–H_ and η^1^_C–H_. The different orientation of the cyclohexane also rules out any stabilization from the geminal C–H bonds that was a feature of the linear alkanes (see Figure S97).

#### 2.3.3 Stability of the σ-Alkane Complexes

Scheme 4 summarizes the room-temperature stabilities of the σ-alkane complexes reported here. In this context “stability” refers to the lifetime of the σ-alkane complex before either (i) loss of the alkane to give the **[1-BAr**^**F**^_**4**_**]** zwitterion and/or (ii) dehydrogenation to an alkene complex. **[1-propane][BAr**^**F**^_**4**_**]** and **[1-hexane][BAr**^**F**^_**4**_**]** fall into the first category, forming the zwitterion in 30–60 min. In contrast, **[1-(2-methylbutane)][BAr**^**F**^_**4**_**]** and **[1-(3-methylpentane)][BAr**^**F**^_**4**_**]** undergo acceptorless dehydrogenation, implying a greater stability toward alkane loss—although zwitterion formation is a competitive process with **[1-(3-methylpentane)][BAr**^**F**^_**4**_**]**.

**Scheme 5:**
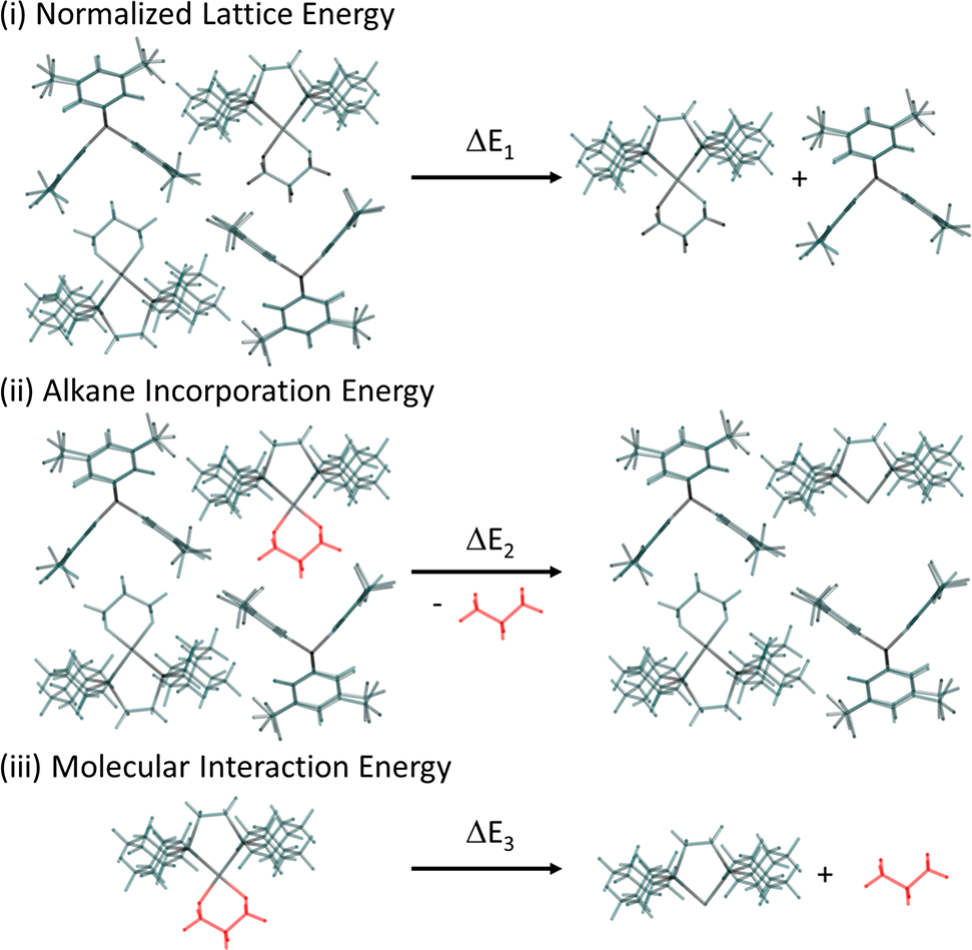
Energy Interaction Terms Computed to Assess the Stability of the σ-Alkane Complexes

To probe these differing, empirically determined behaviors, we have computed Δ*E*_1_, the normalized lattice energy (i.e., taking into account the number of formula units per unit cell), and Δ*E*_2_, the energy required to remove one alkane from the unit cell. These provide a direct measure of the stability of the crystal lattice and of the strength of alkane binding within that lattice, respectively. Δ*E*_3_ quantifies the interaction energy between the alkane ligand and [Rh(Cy_2_PCH_2_CH_2_PCy_2_)]^+^ in the isolated cation. Scheme 5 illustrates these terms for **[1-propane][BAr**^**F**^_**4**_**]**.

For each energy term all geometries were fixed at those found in the fully optimized structures. In addition to the four σ-complexes characterized here, **[1-cyclohexane][BAr**^**F**^_**4**_**]**, **[1-isobutane][BAr**^**F**^_**4**_**]**, **[1-pentane][BAr**^**F**^_**4**_**]**, and **[1-NBA][BAr**^**F**^_**4**_**]** have been added to the analysis. Like its hexane congener, the pentane complex loses the alkane to form the zwitterion, whereas the cyclohexane and isobutane complexes undergo room-temperature dehydrogenation. In contrast to all the other σ-alkane complexes **[1-NBA][BAr**^**F**^_**4**_**]** is essentially indefinitely stable when it is maintained under an inert atmosphere. Thus, taken collectively, these σ-alkane complexes provide a good basis to compare the underlying factors that might influence stability in the solid state.

Computed data are presented in Table 2 and are organized by alkane binding mode and anion environment to bring together the most directly comparable structures with the same tertiary, periodic structure of anions. Some evidence for increased stability with a larger alkane can be seen in the higher values of Δ*E*_1_ and Δ*E*_2_ computed for **[1-cyclohexane][BAr**^**F**^_**4**_**]** vs **[1-propane][BAr**^**F**^_**4**_**]** (both 1,3-binding motifs). These differences arise from a greater interaction not only within the cation (Δ*E*_3_) but also, more significantly, with the surrounding microenvironment, the latter being quantified by Δ*E*_4_ (=Δ*E*_2_ – Δ*E*_3_). Although, as discussed, there are some subtle variations in the C–H→Rh σ-interactions, an additional factor is likely to be the presence of stabilizing dispersive interactions between the alkane ligand and both the cyclohexyl substituents of the chelating phosphine and the surrounding anionic framework. *Intramolecular* dispersive effects have been highlighted as playing a key role in σ-complex stability;^54^ however, our study clearly highlights the role of the solid-state environment in providing additional stabilization and the fact that this factor can be substantial. For example the molecular binding energy of propane (Δ*E*_3_ = 25.7 kcal/mol) is enhanced by almost 33% through *intermolecular* stabilization (Δ*E*_4_ = 8.3 kcal/mol). Incorporating such environmental effects (be these due to the solid state or solvent) is therefore essential in order to provide a full picture of the factors affecting σ-complex stability.

**Table 2:** Computed Normalized Lattice Energy (Δ*E*_1_), Energy Required to Remove on Alkane from the Unit Cell (Δ*E*_2_), and Interaction Energy between Alkane and Metal Fragment (Δ*E*_3_)^a^

complex/microenvironment	Δ*E*_1_	Δ*E*_2_	Δ*E*_3_	Δ*E*_4_^b^
**[1-propane]**^**+**^/*O*_*h*_	110.4	34.0	25.7	8.3
**[1-cyclohexane]**^**+**^/*O*_*h*_	118.0	40.6	28.2	12.5
**[1-(3-methylpentane)]**^**+**^/*O*_*h*_	119.1	45.4	31.7	13.6
**[1-isobutane]**^**+**^/*O*_*h*_	120.0	39.2	27.8	11.4
**[1-(2-methylbutane)]**^**+**^/*O*_*h*_	118.9	c	30.7	c
**[1-NBA]**^**+**^/*O*_*h*_	119.7	47.1	33.1	14.0
**[1-pentane]**^**+**^/BCSA^d^	121.5	45.6	30.7	14.9
**[1-hexane]**^**+**^/BCSA	119.4	48.3	31.4	16.8

a All energies are in kcal/mol. [BAr^F^_4_] anions are not shown in the formula.

b Δ*E*_4_ = Δ*E*_2_–Δ*E*_3_;

c The SCF energy of the apo-alkane unit cell did not converge.

d BCSA denotes a bicapped square antiprism.

Similar trends are seen on comparison of **[1-isobutane][BAr**^**F**^_**4**_**]** and **[1-NBA][BAr**^**F**^_**4**_**]** (both 1,2-binding motifs) and **[1-pentane][BAr**^**F**^_**4**_**]** vs **[1-hexane][BAr**^**F**^_**4**_**]** (2,4-binding motifs within a bicapped square antiprism of anions). In both cases larger values of Δ*E*_2_ are computed for the larger alkane, reflecting increased values of both Δ*E*_3_ and Δ*E*_4_. In **[1-hexane][BAr**^**F**^_**4**_**]** the intermolecular stabilization now rises to above 50% of the intramolecular binding energy. However, these factors do not now translate into a larger lattice energy. More generally, although **[1-propane][BAr**^**F**^_**4**_**]** has the lowest values of Δ*E*_1_ and Δ*E*_2_ and this seemingly correlates with its susceptibility to alkane loss and zwitterion formation, for the larger alkanes no such relation is seen. Instead, these show remarkably little variation in Δ*E*_1_ (120 ± 2 kcal/mol), while the Δ*E*_2_ values are comparable for **[1-hexane][BAr**^**F**^_**4**_**]** and **[1-NBA][BAr**^**F**^_**4**_**]** despite the much greater stability of the latter. This lack of correlation reflects the difficulties in comparing structures with different anion arrangements in the lattice. However, it may also point to the possibility that differential σ-alkane complex stabilities are kinetic in origin rather than thermodynamic.

#### 2.3.4 Anion Microenvironment Effects

**Figure 4:**
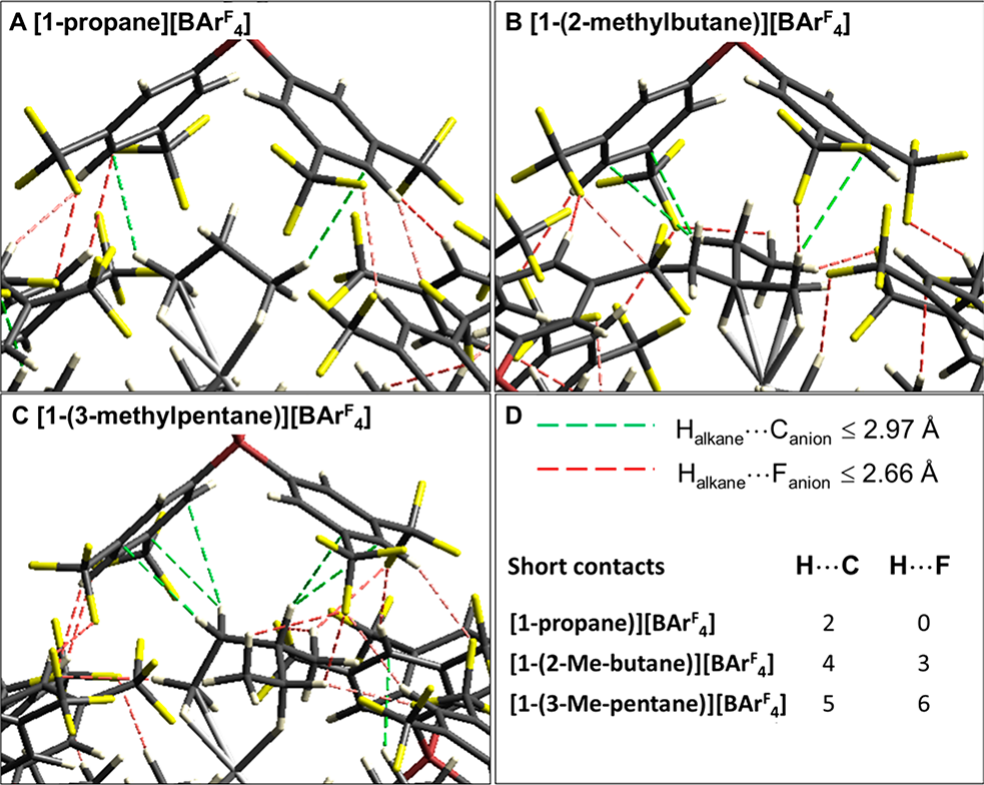
Details of the alkane binding pocket in (A) **[1-propane][BAr**^**F**^_**4**_**]**, (B) **[1-(2-methylbutane)][BAr**^**F**^_**4**_**]**, and (C) **[1-(3-methylpentane)][BAr**^**F**^_**4**_**]** highlighting alkane···anion C–H···C and C–H···F contacts below the sum of the van der Waals radii.^55^ (D) Key and the total number of each short contacts for each species.

We have previously commented on the role of nonclassical C–H^δ+^···F^δ−^–C H-bonds in stabilizing σ-alkane complexes in the solid state.^26,36^Figure 4 highlights short contacts (at or below the sum of the van der Waals radii^55^) of this type, as well as C–H···C contacts between the alkane H atoms and the surrounding anions in the computed structures. For **[1-propane][BAr**^**F**^_**4**_**]** only two C–H···C contacts are present. In **[1-(2-methylbutane)][BAr**^**F**^_**4**_**]** four C–H···C and three C–H···F contacts are seen and these increase in number to five and six, respectively, in **[1-(3-methylpentane)][BAr**^**F**^_**4**_**]**. The C–H···F contacts are also apparent in NCI plots of the proximal ion pairs (Supporting Information). Although it is difficult to quantitively compare these noncovalent interactions, the paucity of such contacts in **[1-propane][BAr**^**F**^_**4**_**]** does correlate with the instability of this system. However, the presence of several C–H^δ+^···F^δ−^–C contacts is not a sufficient condition for stability; thus, **[1-(2-methylbutane)][BAr**^**F**^_**4**_**]** is stable to decomposition and undergoes acceptorless dehydrogenation, whereas **[1-(3-methylpentane)][BAr**^**F**^_**4**_**]** (with twice the number of C–H···F contacts) is susceptible to decomposition.

Overall, several factors appear to be at play in controlling σ-alkane complex stability in the solid state: the strength of the intramolecular Rh···H–C interactions, the extent of intermolecular interactions in the microenvironment, and the fit of the alkane to the binding pocket. In addition, the kinetics of alkane displacement by the incoming [BAr^F^_4_]^−^ anion may also be a factor and this will itself also be related to the microenvironment and the periodic, tertiary structure of the anion framework.

### 2.4 Acceptorless SC-SC Dehydrogenation in the Solid State of 2-Methylbutane and 3-Methylpentane

**Scheme 6:**
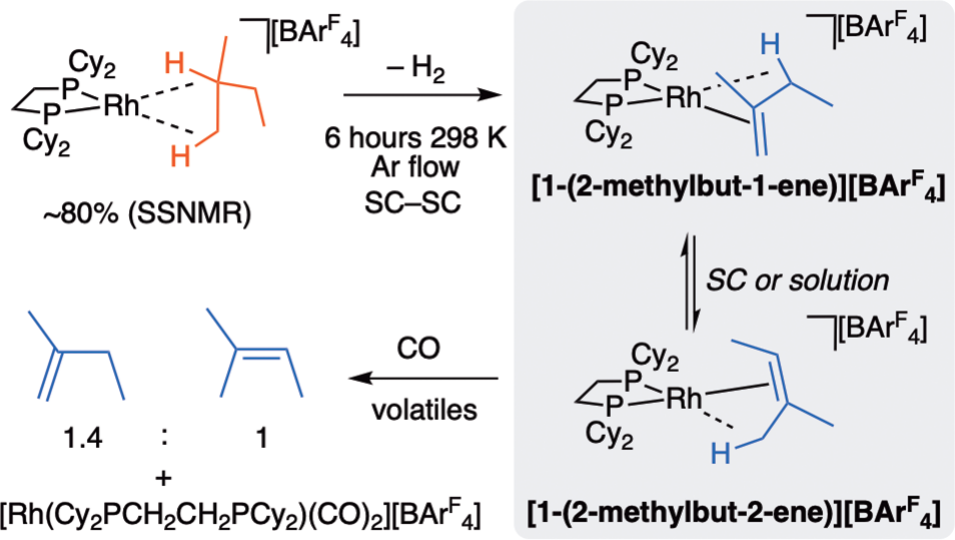
Acceptorless Dehydrogenation of **[1-(2-methylbutane)][BAr^F^_4_]** and Trapping of Alkene Products

In the solid state **[1-(2-methylbutane)][BAr**^**F**^_**4**_**]** undergoes an SC-SC acceptorless dehydrogenation to form a mixture of the two monoalkene isomers **[1-(2-methylbut-1-ene)][BAr**^**F**^_**4**_**]** and **[1-(2-methylbut-2-ene)][BAr**^**F**^_**4**_**]** (Scheme 6). This occurs under an Ar flow (6 h, unoptimized) or vacuum (2 × 10^–2^ mbar, 4 h) to remove H_2_. Addition of CO at 298 K to the resulting single crystals liberates the bound alkenes from the metal center, and an analysis by ^1^H NMR spectroscopy shows that 2-methylbut-1-ene and 2-methylbut-2-ene are formed in a 1.4:1 ratio under these conditions.

**Figure 5:**
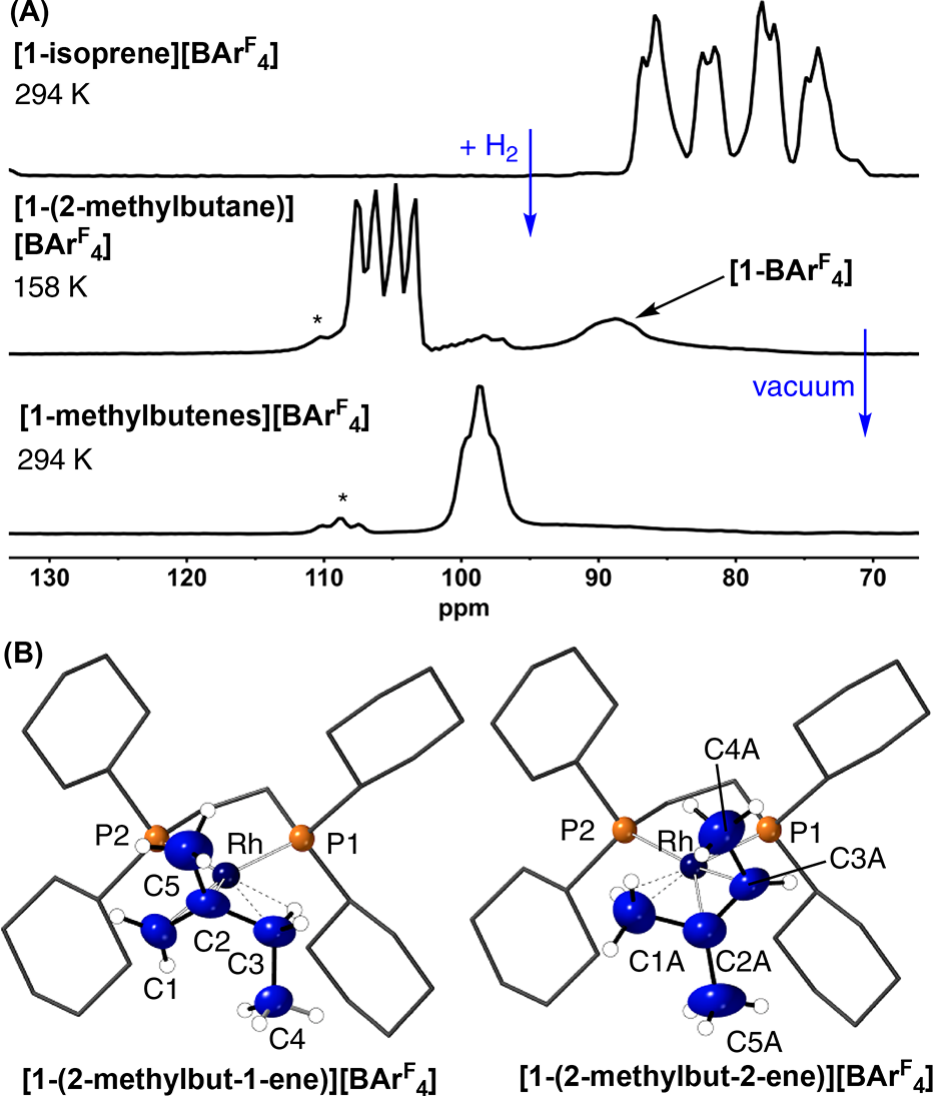
(A) ^31^P{^1^H} SSNMR spectra of the sequential SC–SC transformation of **[1-isoprene][BAr^F^_4_]** to **[1-(2-methylbutane)][BAr^F^_4_]** and **[1-(2-methylbut-1-ene)][BAr**^**F**^_**4**_**]**/**[1-(2-methylbut-2-ene)][BAr**^**F**^_**4**_**]**. Asterisks indicate an unidentified product. (B) Solid-state molecular structures of the dehydrogenation products. Both 50:50 disordered components are shown, with 30% displacement ellipsoids. Selected bond distances (Å) and angle (deg) for [**1-(2-methylbut-1-ene)][BAr**^**F**^_**4**_**]**: Rh–C1, 2.23(2); Rh–C2, 2.12(2); Rh–C3, 2.36(2); C1–C2, 1.39(3); C2–C5, 1.53(3); C2–C3, 1.52(3); ∑ angles around C2, 359.9. Selected bond distances (Å) and angle (deg) for **1-(2-methylbut-2-ene)][BAr**^**F**^_**4**_**]**: Rh–C2A, 2.11(2); Rh–C3A, 2.3(2); Rh–C1A, 2.41(3); C1A-C2A, 1.54(3); C2A-C5A, 1.55(4); C2A-C3A, 1.37(3); ∑ angles around C2A, 359.9°.

Following the overall process from the initial formation of **[1-(2-methylbutane)][BAr**^**F**^_**4**_**]** to the products of dehydrogenation using ^31^P{^1^H} SSNMR spectroscopy shows that the reaction is remarkably clean (Figure 5A). At room temperature the ^13^C{^1^H} SSNMR spectrum of the dehydrogenation products is featureless in the alkene region (100–50 ppm). However, cooling to 158 K reveals three (1 + 1 + 2) alkene environments. This suggests that in the solid state there is a low-energy isomerization process occurring, as described for **[1-propene][BAr**^**F**^_**4**_**]**, likely operating via an allyl/hydride intermediate.^25,36^

This dehydrogenation is an SC-SC process, and the resulting structural refinement of the alkene products (*R*(2σ) = 8.6%) is of sufficient quality to unambiguously determine the formation of a 50:50 mixture of superpositional isomers of **[1-(2-methylbut-1-ene)][BAr**^**F**^_**4**_**]** and **[1-(methylbut-2-ene)][BAr**^**F**^_**4**_**]** (Figure 5B) at 150 K. The formation of an alkene ligand is signposted by coordination of a π-face of the ligand, sp^2^ geometries of the salient carbon atoms, and a corresponding short C–C distance in each isomer. Each monoene ligand also engages in a supporting Rh···H–C agostic interaction (Rh···C3, 2.36(2) Å; Rh···C1A, 2.41(3) Å). These are revealed in the 183 K ^1^H NMR spectrum in CD_2_Cl_2_ solution by the observation of resonances in the alkene region and signals diagnostic of agostic interactions at δ −0.25 and −1.23 (two overlapping signals); the latter are assigned to the diastereotopic agostic interactions from methylene C3 that results in two different agostomers.^56^ The ^31^P{^1^H} NMR spectrum shows resonances due to three Rh(I) complexes, each with inequivalent phosphines. At 298 K this becomes a single environment with coupling to ^103^Rh, and alkene/agostic signals in the ^1^H NMR spectrum are lost, indicating a fluxional process at this temperature, likely a rapid alkene isomerization, as proposed in the solid state.

**Figure 6:**
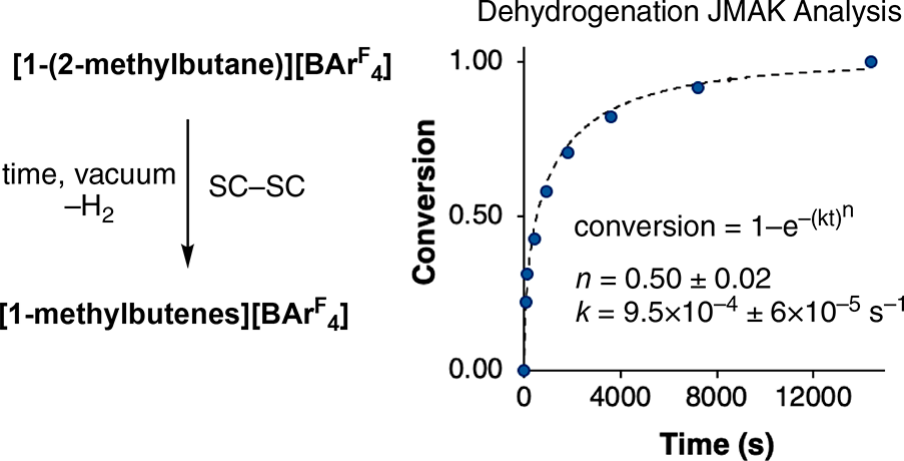
Modified JMAK plot of conversion versus time for the dehydrogenation of *in situ* prepared **[1-(2-methylbutane)][BAr^F^_4_]** to **[1-methylbutenes][BAr^F^_4_]**: (···) fit; (●) experimental data. *k* denotes the growth rate constant and *n* the Avrami exponent.

Kinetic data for this dehydrogenation process were collected by running individual reactions using batches of finely ground **[1-(2-methylbutane)][BAr**^**F**^_**4**_**]** and quenching by dissolving them in CD_2_Cl_2_. The relative ratio of **[1-BAr**^**F**^_**4**_**]** (from decomposition of **[1-(2-methylbutane)][BAr**^**F**^_**4**_**]**) versus **[1-(2-methylbut-1-ene)][BAr**^**F**^_**4**_**]/[1-(2-methylbut-2-ene)][BAr**^**F**^_**4**_**]** was measured using ^31^P{^1^H} NMR spectroscopy (with an internal reference) (Figure 6).^58^ These data were modeled using modified Johnson–Mehl–Avrami–Kologoromov (JMAK) kinetics. This approach describes reaction progress in the solid state in terms of a nucleation and growth model, where *k* is the growth rate constant and *n* is the Avrami exponent.^59^ JMAK analysis has been used to describe SC-SC photoreactions in the solid state.^59−61^ For the process here *k* = 9.5 × 10^–4^ (±6 × 10^–5^) s^–1^ and *n* = 0.50 ± 0.02. Avrami exponents close to *n* = 4, 3, and 2 are suggestive of 3-D, 2-D, and 1-D growth, respectively, while *n* = 1 is indicative of a noncooperative transformation that occurs throughout the crystal. It has been suggested that noninteger Avrami constants, such as those observed here, point to the kinetics being diffusion controlled.^62^ This could be related to a reaction front (i.e., H_2_ loss) that moves through the crystal from outside to inside. A JMAK analysis of the dehydrogenation of **[1-isobutane][BAr**^**F**^_**4**_**]** also has *n* ≈ 0.5,^31^ suggesting that this may be a more general observation for this type of reactivity in the single crystal. Avrami exponents of *n* ≈ 0.5 have been measured for other SC-SC processes.^57^

**Scheme 7:**
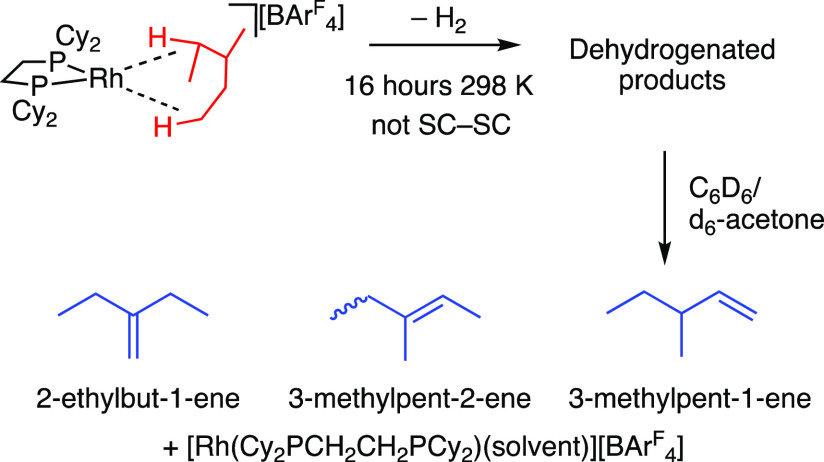
Acceptorless Dehydrogenation of **[1-(3-methylpentane)][BAr^F^_4_]** and Trapping of Alkene Products

A similar dehydrogenation process occurs for **[1-(3-methylpentane)][BAr**^**F**^_**4**_**]** in the single crystal over the course of 16 h under a dynamic vacuum (10^–2^ mbar) to form a mixture of methylpentene isomers bound to Rh(I): **[1-(methylpentenes)][BAr**^**F**^_**4**_**]**. However, this is not an SC-SC process; the crystallinity is lost, and signals due to the decomposition product **[1-BAr**^**F**^_**4**_**]** grow in considerably (∼35%). For this reason a reaction progress analysis using the JMAK approach was not appropriate. The presence of **[1-BAr**^**F**^_**4**_**]** and other decomposition products also meant that the solution characterization was not unambiguous. Dissolution of the solid in C_6_D_6_/*d*_6_-acetone liberated the bound alkenes from the metal center by forming the corresponding benzene adduct of Rh(I).^31^^1^H NMR spectroscopy and ESI-MS data show that these alkenes are a mixture of isomers of methylpentene (Scheme 7)—confirming that an acceptorless dehydrogenation in [**1-(3-methylpentane)][BAr**^**F**^_**4**_**]** has occurred.

The acceptorless dehydrogenation of alkanes to alkenes is an important industrial process that requires high temperatures and a heterogeneous catalyst, as it is an endothermic process.^63^ Using molecular organometallic systems it can be driven catalytically by removing H_2_^64^ or working in the solid phase under continuous-flow conditions.^38,65^ We have recently demonstrated that spontaneous, albeit stoichiometric, dehydrogenation and H_2_ loss occur in the well-defined σ-alkane complexes **[1-isobutane][BAr**^**F**^_**4**_**]** and **[1-cyclohexane][BAr**^**F**^_**4**_**]** to form isobutene and cyclohexadiene complexes, respectively.^31^ This demonstrates that, if pre-equilibria for alkane binding at a metal center is biased to the σ-alkane complex, dehydrogenation is kinetically a rather straightforward process. While the coordination of the alkene product makes these processes more thermodynamically favorable than for the free alkane/alkene, calculations show that they are still slightly endergonic.^31^ The same concept operates here but is now extended to methylbutane and methylpentane alkane ligands. The mechanism for dehydrogenation (which is also the microscopic reverse of hydrogenation), as described in detail for **[1-cyclohexane][BAr**^**F**^_**4**_**]**,^31^ likely proceeds via initial C–H oxidative cleavage followed by β-H elimination and loss of H_2_, closely related to solution-based dehydrogenation systems.^2^

## Conclusions

By using the {Rh(Cy_2_PCH_2_CH_2_PCy_2_)}^+^ metal fragment **1** with a supporting anionic framework of [BAr^F^_4_]^−^ anions, we have prepared a series of C_3_–C_6_ linear and branched σ-alkane complexes using single-crystal to single-crystal solid/gas transformations from the corresponding alkene precursors. In combination with our previous studies using the same metal/ligand/anion combination, this provides structurally characterized σ-alkane complexes of propane, isobutane,^31^ pentane,^47^ 2-methylbutane, hexane, cyclohexane,^31^ 3-methylpentane, and norbornane.^26,34^ These complexes display a wide variety of Rh(I)···H–C binding motifs at the metal coordination site: 1,2-η^2^:η^2^, 1,3-η^2^:η^2^, 1,4-η^1^:η^2^, and intermediate η^1^/η^2^. In addition methyl, methylene, and methine C–H groups are all shown to be involved in bonding to Rh. Detailed DFT, QTAIM, NCI, and NBO computational studies on the isolated cations reveal additional subtleties of the Rh···H–C binding modes, including the contribution of weaker geminal C–H···Rh interactions with the linear alkanes.

The new alkane complexes show a range of stabilities with respect to alkane loss in the solid state. Thus, **[1-propane][BAr**^**F**^_**4**_**]** and **[1-hexane][BAr**^**F**^_**4**_**]** both lose the alkane within 30–60 min to form the zwitterionic complex **[1-BAr**^**F**^_**4**_**]**. In contrast, **[1-(2-methylbutane)][BAr**^**F**^_**4**_**]** is stable toward alkane loss and instead undergoes acceptorless alkane dehydrogenation to form a bound alkene complex, while for **[1-(3-methylpentane)][BAr**^**F**^_**4**_**]** this dehydrogenation dominates with zwitterion formation being a competing process. Computed alkane bonding energies show that the interaction between the alkane ligand and the {Rh(Cy_2_PCH_2_CH_2_PCy_2_)}^+^ fragment within the isolated cation (Δ*E*_3_) does not by itself reflect the empirically observed relative stabilities toward loss of the alkane in the solid state. For example, **[1-propane][BAr**^**F**^_**4**_**]** and **[1-cyclohexane][BAr**^**F**^_**4**_**]** show dramatically different stabilities but rather similar values of Δ*E*_3_. Thus, while an analysis that is restricted to the primary coordination sphere provides detailed insight into the bonding at the metal center, it does not capture the stability of these σ-alkane complexes in the solid state. The secondary coordination sphere due to the microenvironment provided by the anionic framework of [BAr^F^_4_]^−^ counterions must also be taken into account. This is shown to provide considerable additional stabilization and hence significantly increases the alkane binding energy within the lattice (Δ*E*_2_). A somewhat better correlation between the energy associated with microenvironment stabilization (Δ*E*_4_) and empirical stability is seen. This arises from both dispersive interactions and more localized C–H···H–X (X = C, F) contacts, which are maximized with larger, and branched, alkane ligands. However, there is also an upper limit. Too large an alkane also results in decomposition of the complex, despite a numerical increase in C–H···H–X contacts. This suggests that there are ideal conditions for the fit of the alkane into the anion binding pocket. Highlighting this, **[1-NBA][BAr**^**F**^_**4**_**]**, **[1-isobutane][BAr**^**F**^_**4**_**]**, and **[1-(2-methylbutane)][BAr**^**F**^_**4**_**]** are remarkably stable, and these alkanes clearly find a good fit in the binding pocket *and* receive significant stabilization from the microenvironment, with Δ*E*_4_ contributing up to an additional 40% to the total alkane binding energies in these cases. In some respects this is related to the optimal guest volume to host volume ratio for supramolecular systems in solution,^66^ where noncovalent interactions are crucial in determining structure and reactivity.^67^

In addition, kinetic factors, which are even more challenging to deconvolute in molecular solid-state systems, will no doubt affect the stability. While more speculative, a comparison between ∼*O*_*h*_ and bicapped-square-prism (BCSP) arrangements of anions is instructive here. For example, **[1-cyclohexane][BAr**^**F**^_**4**_**]** and **[1-hexane][BAr**^**F**^_**4**_**]** both enjoy significant stabilization from the microenvironment, but the latter has a BCSP tertiary structure of anions and is significantly less stable with regard to decomposition.

When the balance of these factors is just right (the “Goldilocks” conditions), room-temperature stable σ-alkane complexes are generated that then undergo remarkable reactivity, such as the SC-SC acceptorless dehydrogenation for **[1-(2-methylbutane)][BAr**^**F**^_**4**_**]** described here or elsewhere for **[1-isobutane][BAr**^**F**^_**4**_**]**,^31^ and selective H/D exchange in **[1-NBA][BAr**^**F**^_**4**_**]**.^34^ This combination of primary active site (alkane bonding), secondary structure (anion microenvironment), and the tertiary, periodic, motif of the anions has parallels with enzymatic systems, where equivalent environments and structures work holistically to control the reactivity, selectivity, and stability.^68,69^ It will be interesting to see whether fine control of these elements in SMOM systems, by a judicious choice of metal/ligand fragment, anion structure, and packing motifs, encourages the binding of even more weakly binding alkanes, such as ethane and methane.

## Supporting Information Available

Full details of experimental methods, characterization data, details of computational methods and experiments, and single-crystal X-ray diffraction collection and refinement data (PDF)
Computed Cartesian coordinates (XYZ)

## Supplementary Material

ja1c00738_si_001.pdf

ja1c00738_si_002.xyz

## Accession Codes

CCDC 2056857–2056860 and 2056862–2056866 contain the supplementary crystallographic data for this paper. These data can be obtained free of charge via www.ccdc.cam.ac.uk/data_request/cif, or by emailing data_request@ccdc.cam.ac.uk, or by contacting The Cambridge Crystallographic Data Centre, 12 Union Road, Cambridge CB2 1EZ, UK; fax: +44 1223 336033.
