# Supplementary material for: A Series of Crystallographically Characterized Linear and Branched σ-Alkane Complexes of Rhodium: From Propane to 3-Methylpentane
Source: J Am Chem Soc. 2021 Mar 26;143(13):5106–20. doi: 10.1021/jacs.1c00738 (PMC8154534; doi:10.1021/jacs.1c00738)
Supplement: Supplementary file 1 — ja1c00738_si_001.pdf [file ja1c00738_si_001.pdf]

Electronic Supporting Information

A Series of Crystallographically Characterized Linear and Branched σ-Alkane Complexes of Rhodium: From Propane to 3-Methylpentane

Alexander J. Bukvic,[a,b] Arron L. Burnage,[c] Graham J. Tizzard,[d] Antonio J. Martínez-Martínez,[b] Alasdair I. McKay,[b] Nicholas H. Rees,[b] Bengt E. Tegner,[c] Tobias Krämer,[c] Heather Fish,[a] Mark Warren,[e] Simon J. Coles,[d] Stuart A. Macgregor[c]* and Andrew S. Weller.[a]*

[a] Department of Chemistry, University of York, Heslington, York, YO10 5DD, UK. Email: andrew.weller@york.ac.uk

[b] Department of Chemistry, Chemistry Research Laboratories, University of Oxford, Oxford, OX1 3TA, UK.

[c] Institute of Chemical Sciences, Heriot-Watt University, Edinburgh, EH14 4AS, UK. Email: S.A.Macgregor@hw.ac.uk

[d] UK National Crystallography Service, University of Southampton, Highfield, Southampton, SO17 1BJ, UK.

[e] Diamond Light Source Ltd., Diamond House, Harwell Science and Innovation Campus, Didcot, OX11 0DE, UK.

TABLE OF CONTENTS

S.1. EXPERIMENTAL DETAILS 4

S.1.1. General methods 4

S.2. SYNTHETIC PROCEDURES 6

S.2.1. [Rh($Cy_2PCH_2CH_2PCy_2$)(C_3H_8)][BAr^F_4], [1-propane][BAr^F_4] 6

S.2.1.1. Synthesis of a precursor propene complex, [1-propene][BAr^F_4] 6

S.2.1.2. Synthesis of a propane complex, [1-propane][BAr^F_4] 6

S.2.1.3. Charaterisation data and spectra for [1-propane][BAr^F_4] 7

S.2.1.4. Stability of [1-propane][BAr^F_4] at 294 K 10

S.2.2. [Rh($Cy_2PCH_2CH_2PCy_2$)(C_5H_{12})][BAr^F_4], [1-(2-methylbutane)][BAr^F_4] 11

S.2.2.1. Synthesis of a precursor 2-methylbutane complex, [1-isoprene][BAr^F_4] 11

S.2.2.2. Charaterisation data and spectra for [1-isoprene][BAr^F_4] 11

S.2.2.3. Synthesis of a 2-methylbutane complex, [1-(2-methylbutane)][BAr^F_4] 16

S.2.2.4. Charaterisation data and spectra for [1-(2-methylbutane)][BAr^F_4] 16

S.2.3. [Rh($Cy_2PCH_2CH_2PCy_2$)(C_5H_{10})][BAr^F_4], [1-methylbutenes][BAr^F_4] 18

S.2.3.1. Synthesis of a methylbutenes complex, [1-methylbutenes][BAr^F_4] 18

S.2.3.2. Charaterisation data and spectra for [1-methylbutenes][BAr^F_4] 19

S.2.3.3. Liberation of bound methylbutenes from [1-methylbutenes][BAr^F_4] 26

S.2.3.4. Reaction kinetics of the dehydrogenation of [1-(2-methylbutane)][BAr^F_4] 27

S.2.4. [Rh($Cy_2PCH_2CH_2PCy_2$)(C_6H_{14})][BAr^F_4], [1-hexane][BAr^F_4] 29

S.2.4.1. Synthesis of a precursor hexane complex, [1-hexadiene][BAr^F_4] 29

S.2.4.2. Charaterisation data and spectra for [1-hexadiene][BAr^F_4] 30

S.2.4.3. Synthesis of a hexane complex, [1-hexane][BAr^F_4] 34

S.2.4.4. Charaterisation data and spectra for [1-hexane][BAr^F_4] 34

S.2.4.5. Stability of [1-hexane][BAr^F_4] at 294 K 36

S.2.5. [Rh($Cy_2PCH_2CH_2PCy_2$)(C_6H_{14})][BAr^F_4], [1-(3-methypentane)][BAr^F_4] 37

S.2.5.1. Synthesis of a precursor 3-methylpentane complex, [1-(C_6H_{10})][BAr^F_4] (C_6H_{10} = 3-methyl-1,3-pentadiene or 2-ethylbutadiene) 37

S.2.5.2. Charaterisation data and spectra for [1-(C_6H_{10})][BAr^F_4] 38

S.2.5.3. Liberation of bound C_6-isomers from [1-(C_6H_{10})][BAr^F_4] (C_6H_{10} = 3-methyl-1,3-pentadiene or 2-ethylbutadiene) 42

S.2.5.4. Synthesis of a 3-methylpentane complex, [1-(3-methylpentane)][BAr^F_4] 44

S.2.5.5. Charaterisation data and spectra for [1-(3-methylpentane)][BAr^F_4] 44

S.2.5.6. Stability of [1-(3-methylpentane)][BAr^F_4] at 294 K 46

S.2.6. The partial dehydrogenation of [1-(3-methylpentane)][BAr^F_4] 47

S.2.6.1. Proposed methylpentenes complex 47

S.2.6.2. Charaterisation data and spectra ... 48
S.2.6.3. Liberation of bound methylpentenes ... 50
S.2.7. [Rh($Cy_2PCH_2CH_2PCy_2$)(C_8H_{14})][BAr^F_4], [1-octadiene][BAr^F_4] ... 51
S.2.7.1. Synthesis of an octodiene complex, [1-octadiene][BAr^F_4] ... 51
S.2.7.2. Charaterisation data and spectra for [1-octadiene][BAr^F_4] ... 52
S.2.7.3. Attempted synthesis of ethane and octane complexes ... 54
S.3 CRYSTALLOGRAPHIC AND REFINEMENT DATA ... 55
S.3.1. Crystal structure determinations ... 55
S.3.1.1. Diamond Light Source ... 55
S.3.1.2. University of Oxford ... 58
S.3.1.3. National Crystallography Service, Southampton, UK ... 58
S.3.2. Additional comments on solved structures ... 58
S.3.3. Refinement tables ... 59
S.3.4. Structural images from single crystal X-ray diffraction experiments ... 62
S.3.5. Comparison of Rh···C distances from selected σ-alkane complexes ... 71
S.4. COMPUTATIONAL METHODS ... 72
S.4.1. Solid State Calculations ... 72
S.4.2. Molecular Calculations ... 73
S.5. ELECTRONIC STRUCTURE ANALYSIS ... 76
S.5.1. [1-propane][BAr^F_4] ... 76
S.5.2. [1-pentane][BAr^F_4] ... 84
S.5.3. [1-(2-methylbutane)][BAr^F_4] ... 90
S.5.4. [1-hexane][BAr^F_4] ... 95
S.5.5. [1-(3-methylpentane)][BAr^F_4] ... 101
S.5.6. [1-cyclohexane][BAr^F_4] ... 107
S.5.7. [1-NBA][BAr^F_4] ... 113
S.5.8. [1-isobutane][BAr^F_4] ... 118
S.6. REFERENCES ... 123

S.1. Experimental details

S.1.1. General methods

All manipulations (unless otherwise stated) were performed under an atmosphere of argon, using standard Schlenk techniques on a dual vacuum/inlet manifold or by employment of an MBraun glovebox. Glassware was dried in an oven at 130 °C overnight prior to use. Pentane, benzene and dichloromethane (abbreviated as CH_2Cl_2) were dried using an MBraun SPS-800 solvent purification system and degassed by three freeze-pump-thaw cycles. 1,2-difluorobenzene (abbreviated as $F_2C_6H_4$) and 1,2-dichloroethene were prepared by stirring over Al_2O_3 for two hours then over CaH_2 overnight before being vacuum distilled and subsequently degassed by three freeze-pump-thaw cycles. Dichloromethane-*d*$_2$ (abbreviated to CD_2Cl_2), acetonitrile-*d*$_3$ (abbreviated to MeCN-*d*$_3$) and acetone-*d*$_6$ were dried by stirring over CaH_2 overnight before being vacuum distilled onto 3 Å molecular sieves and subsequently degassed by three freeze-pump-thaw cycles. Benzene-*d*$_6$ (abbreviated as C_6D_6) was stirred over Na pieces overnight before being vacuum distilled onto 3 Å molecular sieves and subsequently degassed by three freeze-pump-thaw cycles. Propene gas was purchased from CK Gases and used as received. All other chemicals were purchased from commercial vendors and used as received.

$[Rh(Cy_2PCH_2CH_2PCy_2)(1,2\text{-}F_2C_6H_4)][BAr^F_4]$ **[1-F$_2$C$_6$H$_4$][BAr$^F_4$]**[S1], $[Rh(Cy_2PCH_2CH_2PCy_2)(NBD)][BAr^F_4]$ (NBD = norbornadiene) **[1-NBD][BAr$^F_4$]**[S2] and $[Rh(Cy_2PCH_2CH_2PCy_2)(NBA)][BAr^F_4]$ (NBA = norbornane) **[1-NBA][BAr$^F_4$]**[S2] were prepared by the literature procedures.

Solution NMR data were collected on either a Bruker AVD 500 MHz or a Bruker Ascend 400 MHz spectrometer at room temperature unless otherwise started. Non-deuterated solvents were locked to standard external CD_2Cl_2 solutions. Residual protio solvent resonances were used as a reference for ^{1}H NMR spectra. ^{31}P{^{1}H} NMR spectra were referenced externally to 85 % H_3PO_4 (D_2O). All chemical shifts (δ) are quoted in ppm and coupling constants in Hz.

Solid state NMR (SSNMR) samples were prepared packing powdered microcrystalline sample into either a 3.2 mm or 4 mm zirconia solid state rotor inside an argon filled glove box. SSNMR spectra were obtained on a Bruker AVANCE III HD spectrometer equipped with a 9.4 Tesla magnet operating at 100.6 MHz for ^{13}C and 162 MHz for ^{31}P respectively, with a magic-angle spinning (MAS) rate of 10 kHz. Relaxation time for ^{1}H and contact time for ^{31}P{^{1}H} CP/MAS and ^{13}C{^{1}H} CP/MAS NMR experiments were optimised for each compound as appropriate. All ^{13}C{^{1}H} CP/MAS spectra were referenced to adamantane (up

field methine resonance, δ 29.5)[S3] on a scale where δ (TMS) = 0 as a secondary reference. The temperature for Variable Temperature (VT) NMR experiments at low temperatures was externally calibrated using lead nitrate ($PbNO_3$).

Electrospray ionization mass spectrometry (ESI-MS) was carried out using a Bruker MicrOTOF instrument directly connected to a modified Innovative Technology glovebox.[S4] Typical acquisition parameters were used (sample flow rate: 4 μL min^{-1}, nebulizer gas pressure: 0.4 bar, drying gas: Nitrogen at 333 K flowing at 4 L min^{-1}, capillary voltage: 4.5 kV, exit voltage: 60 V). The spectrometer was calibrated using a mixture of tetraalkyl ammonium bromides $[N(C_nH_{2n+1})_4]Br$ (n = 2-8, 12, 16 and 18). Samples were diluted to a concentration of 1 × 10^{-6} M in the appropriate solvent before sampling by ESI-MS.

Elemental analyses were conducted by Mr. Stephan Boyer at London Metropolitan University.

S.2. Synthetic Procedures

S.2.1. [Rh($Cy_2PCH_2CH_2PCy_2$)(C_3H_8)][BAr^F_4], [1-propane][BAr^F_4]

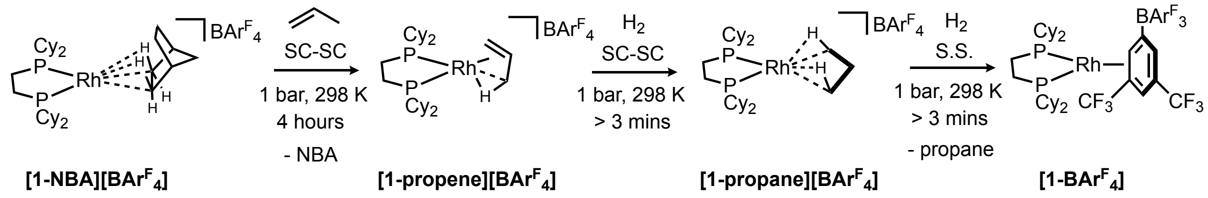

S.2.1.1. Synthesis of a precursor propene complex, [1-propene][BAr^F_4]

Note: **[1-propene][BAr^F_4]** has been previously reported[S5] and the synthesis is repeated below. There has been no change in the synthetic route compared to that previously reported.

A J. Young flask (~50 mL) was loaded with crystals of **[1-NBA][BAr^F_4]** (750 mgs), evacuated (< 2 × 10^{-2} mbar) and re-charged with propene gas (1 bar, 298 K). The flask was left to stand for 4 hours. The excess propene and liberated norbornane could be removed under vacuum (< 2 × 10^{-2} mbar, 18 hours) to quantitively yield [Rh($Cy_2PCH_2CH_2PCy_2$)(C_3H_6)][BAr^F_4], **[1-propene][BAr^F_4]**. The crystalline material is stored in an argon filled glove box in a freezer operating at –25 °C.

S.2.1.2. Synthesis of a propane complex, [1-propane][BAr^F_4]

For Single-Crystal X-Ray Diffraction: Conducted at Diamond Light Source synchrotron facility,[S6] a single orange coloured crystal of **[1-propene][BAr^F_4]** was affixed upon a 50 μm MiTeGen Micromount using epoxy resin. The mount was inserted and affixed inside a 100 μm quartz capillary. This in turn was supported into a stainless-steel gas-line adapter fitted with Swagelok adapters, forming the gas cell. The above steps were performed inside an argon filled glove box. Each gas cell could be transferred and cycled onto the specially adapted gas-rig connected directly to the diffractometer upon Beamline I19 at Diamond Light Source. A unit-cell check was performed on each crystal to ensure the correct starting material had been mounted. Further details on this procedure and equipment is shown in Section S.3.1.1.

The crystal of **[1-propene][BAr^F_4]** was then treated with H_2 (1 bar, 293 K) as the cryostream was set to cool to 150 K at a rate of 360 K / hour. This gave an approximate time of ~3 minutes between 293 K and 275 K. The colour of the crystals slowly turned from orange to

dark red in this time and no reactivity with H_2 was observed below this temperature. When the cryostream reached 150 K, an X-ray diffraction study was undertaken and the structure of $[Rh(Cy_2PCH_2CH_2PCy_2)(C_3H_8)][BAr^F_4]$ **[1-propane][BAr$^F_4$]** was refined. Further details of this structural refinement are shown in Section S.3.

For Solid-State NMR Spectroscopy: A powdered microcrystalline sample of **[1-propene][BAr$^F_4$]** (45 mgs) was packed in a 4.0 mm SSNMR rotor inside an argon filled glove box. The rotor was then placed in a custom-built glass J. Young flask[S5] and the flask was placed under a vacuum (< 2 × 10^{-2} mbar) and then placed under an atmosphere of H_2 (1 bar, 298 K). The rotor's cap was then immediately fitted under a flush of H_2. The sample was rapidly transferred into the bore of a SSNMR spectrometer at 298 K and cooled to 158 K (~10 minutes) before being analysed by $^{31}P\{^1H\}$ and $^{13}C\{^1H\}$ solid state NMR spectroscopy. At 158 K no reactivity with H_2 is observed.

Note: This procedure was optimised. The time period at 298 K (~10 minutes) was found to be the experimentally shortest time possible, however this still lead to a significant amount of decomposition to $[Rh(Cy_2PCH_2CH_2PCy_2)\{(\eta^6\text{-}C_6H_3(CF_3)_2)BAr^F_3\}]$ **[1-BAr$^F_4$]** ($^{31}P\{^1H\}$ SSNMR: δ 91)[S2] under an H_2 atmosphere, see Figure S2. Minor signals assigned to **[1-propene][BAr$^F_4$]** ($^{31}P\{^1H\}$ SSNMR: δ 99, 93)[S5] are also observed as a likely result of incomplete hydrogenation of the sample.

S.2.1.3. Charaterisation data and spectra for [1-propane][BAr$^F_4$]

$^{31}P\{^1H\}$ SSNMR (162 MHz, 158 K, 10 kHz spin rate): δ 106.8 (br s) (~30 % yield).
$^{13}C\{^1H\}$ SSNMR (101 MHz, 158 K, 10 kHz spin rate): δ 163.4 (ArC), 133.8 (ArC), 129.3 (ArC), 124.5 (ArC), 116.5 (ArC), 34.6, 29.2, 25.1, 19.2, 15.5 (multiple aliphatic resonances).

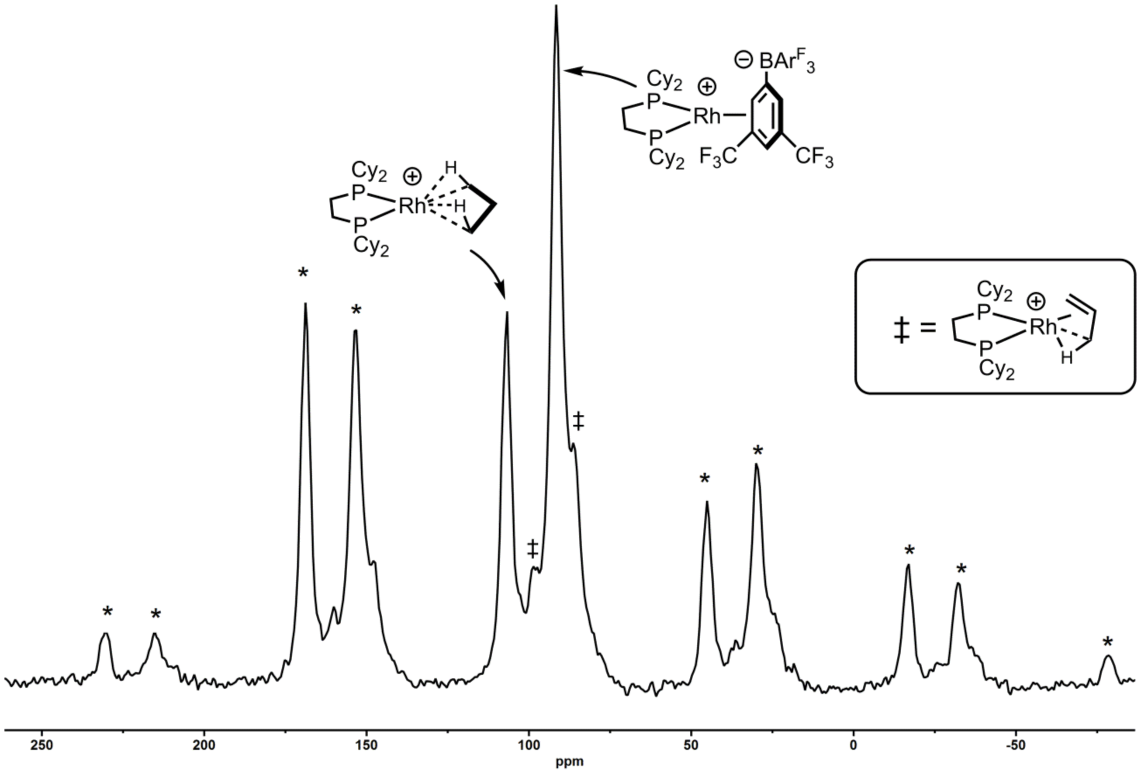

Figure S1: The $^{31}P\{^{1}H\}$ SSNMR spectrum (162 MHz, 158 K, 10 kHz spin rate) of **[1-propane][BAr$^{F}_{4}$]** prepared *in situ* after 3 minutes under H_2 at 298 K. The resonances marked * are due to spinning sidebands and ‡ from **[1-propene][BAr$^{F}_{4}$]**.[S5]

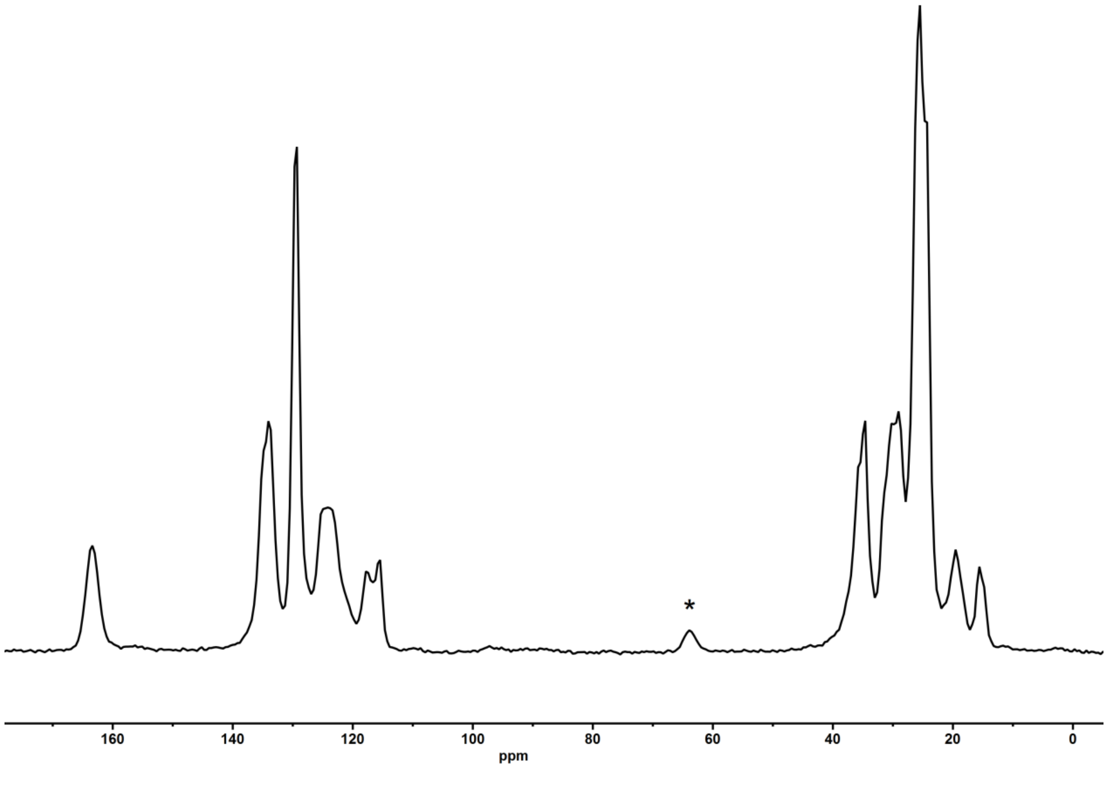

Figure S2: The $^{13}C\{^{1}H\}$ SSNMR spectrum (100 MHz, 158 K, under H_2, 10 kHz spin rate) of **[1-propane][BAr$^{F}_{4}$]** prepared *in situ*. The resonance marked * is due to a spinning sideband.

In Figure S1, we suggest that the multiple species present are due to the unique hydrogenation kinetics present during *in situ* hydrogenations within the solid-state rotor. This is not the case compared to *in situ* single crystal experiments, as only evidence for **[1-propane][BAr^F$_4$]** could be refined. The densely packed nature of the material within the SSNMR rotor limits the movement of H_2 throughout the rotor within the time and so, longer hydrogenation times than necessary are required to fully hydrogenate the sample. However, these longer hydrogenation times also lead to an increased concentration of decomposition product **[1-BAr^F$_4$]**.

The procedure has been optimised for **[1-propane][BAr^F$_4$]** and the signals relating to **[1-propene][BAr^F$_4$]** in Figure S1 are most likely from incomplete hydrogenation of the sample within this time and are from sample most likely located at the bottom of the rotor. This effect is not present in single-crystal X-ray diffraction studies as H_2 gas can access the crystal from multiple points, visualised in Figure S3.

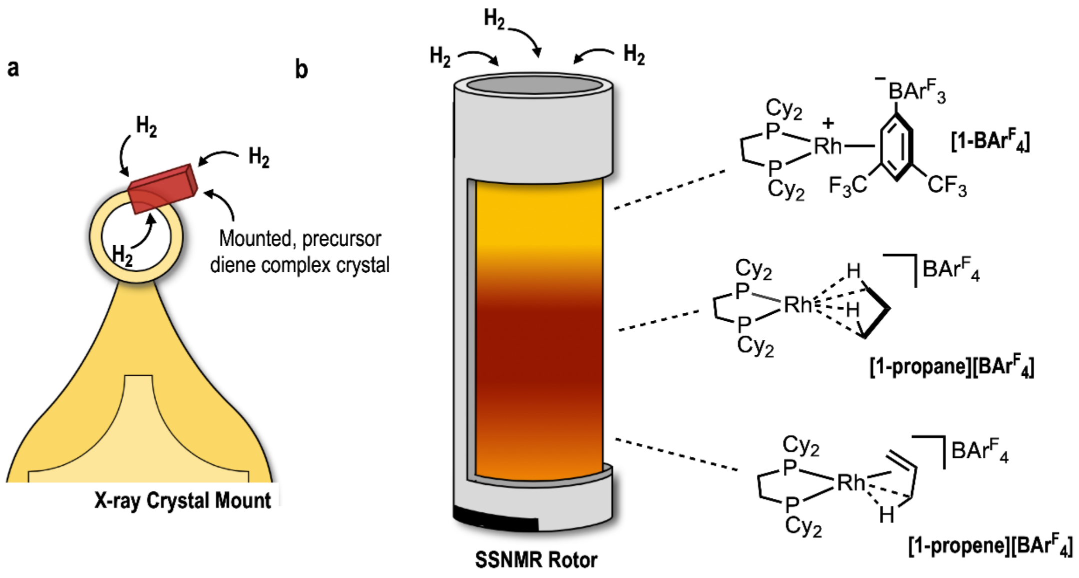

Figure S3: Hydrogen gas accessibility in **a**) single-crystal X-ray diffraction experiments and **b**) SSNMR spectroscopy.

S.2.1.4. Stability of [1-propane][BAr$^F_4$] at 294 K

On the same sample as presented in Figures S1, the solid-state NMR spectrometer probe was warmed to 294 K (from 158 K) and $^{31}P\{^1H\}$ SSNMR spectra collected until complete decomposition of **[1-propane][BAr$^F_4$]** was observed. During this time, the solid-state NMR rotor was under a flushing N_2 atmosphere.

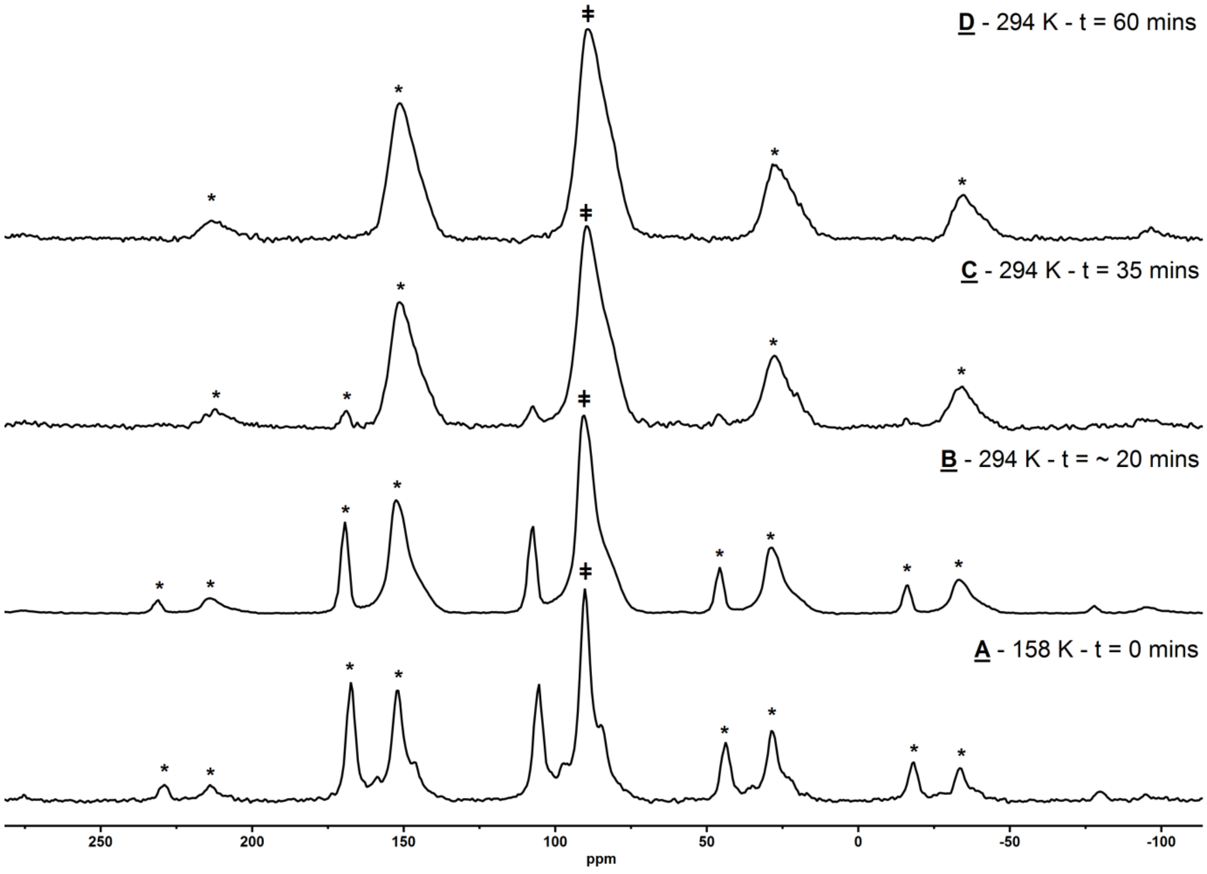

Figure S4: The $^{31}P\{^1H\}$ SSNMR spectra (162 MHz, 10 kHz spin rate) of **A**: **[1-propane][BAr$^F_4$]**, as prepared at 158 K. **B**: Spectrum collected upon warming to 294 K. Total time at 294 K including instrument warming, stabilisation and experimental set-up / collection is **20 minutes**. **C**: Total time at 294 K including instrument warming, stabilisation and experimental set-up / collection is **35 minutes**. **D**: Total time at 294 K including instrument warming, stabilisation and experimental set-up / collection is **60 minutes**. Peaks marked * are spinning sidebands and ǂ are assigned to **[1-BAr$^F_4$]** in all parts.

S.2.2. [Rh($Cy_2PCH_2CH_2PCy_2$)(C_5H_{12})][BAr^F_4], [1-(2-methylbutane)][BAr^F_4]

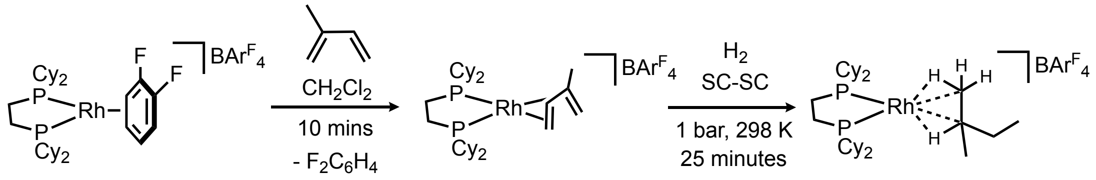

[1-$F_2C_6H_4$][BAr^F_4] **[1-isoprene][BAr^F_4]** **[1-(2-methylbutane)][BAr^F_4]**

S.2.2.1. Synthesis of a precursor 2-methylbutane complex, [1-isoprene][BAr^F_4]

A pale-yellow solution of **[1-$F_2C_6H_4$][BAr^F_4]** (600 mgs, 0.40 mmol) in CH_2Cl_2 (20 mL) was treated with neat 2-methyl-1,3-butadiene (50 µL, 0.5 mmol) at ambient temperature. The resultant deep red solution was stirred at ambient temperature for 30 minutes. Pentane (100 mL) was then added with vigorous stirring which resulted in the formation of a red precipitate. The solid was isolated by filtration and washed with pentane (2 × 20 mL). The solid was then dissolved in CH_2Cl_2 (~8 mL) and filtered into a J. Youngs crystallisation flask and layered with pentane. Deep red crystals were obtained after storage for 1 week at room temperature of [Rh($Cy_2PCH_2CH_2PCy_2$)(*iso*-C_5H_8)][BAr^F_4], **[1-isoprene][BAr^F_4]** (Yield: 551 mgs, 95 %).

S.2.2.2. Charaterisation data and spectra for [1-isoprene][BAr^F_4]

^{1}H NMR (CD_2Cl_2, 298 K, 400 MHz): δ 7.73 (s, 8H, *ortho*-ArH), 7.57 (s, 4H, *para*-ArH), 5.16 (d. of d. of d., 1H, alkene-H, J = 13.8, 8.1, 1.6 Hz), 4.38 (d, 1H, alkene-H, J = 2.8 Hz), 4.32 (d. of d. of d., 1H, alkene-H, J = 7.8, 4.1, 1.3 Hz), 2.74 (d. of m., 1H, alkene-H, J = 14.1, 1.3 Hz), 2.70 (br. m., 1H, alkene-H), 2.23-1.52 (br m, 31H, multiple overlapping aliphatic CH), 1.42-0.84 (br m, 20H, multiple overlapping aliphatic CH).

^{31}P{^{1}H} NMR (CD_2Cl_2, 298 K, 162 MHz): δ 84.3 (d, J_{RhP} = 163 Hz, J_{PP} = 22 Hz), δ 79.5 (d, J_{RhP} = 169 Hz, J_{PP} = 22 Hz).

^{19}F{^{1}H} NMR (CD_2Cl_2, 298 K, 377 MHz): δ -62.9 (s).

^{13}C{^{1}H} NMR (CD_2Cl_2, 298 K, 126 MHz): δ 162.4 (q, J_{CB} = 50 Hz, *ipso*-ArC), 135.1 (s, *ortho*-ArC), 129.3 (q, J_{CF} = 32 Hz, meta-ArC), 125.1 (q, J_{CF} = 272 Hz, -CF_3), 120.0 (apparent triplet, C=C, J = 4.3 Hz), 117.8 (s, *para*-ArC), 101.2 (apparent triplet, C=C, J = 4.3 Hz), 63.7 (d. of d. of d., C=C, J = 14.4, 5.3, 1.5 Hz), 62.8 (d. of d. of d., C=C, J = 10.9, 7.6, 1.7 Hz), 38.4 – 22.6 (m, overlapping cyclohexyl-CH_2).

The solid-state structure of **[1-isoprene][BAr$^F_4$]** shows two crystallographically distinct orientations of the bound isopentadiene ligand (see Section S.3.). These two disorder components can be identified in the solid-state NMR spectra:

$^{31}P\{^1H\}$ SSNMR (162 MHz, 294 K, 10 kHz spin rate): Orientation 1**:** δ 86.2 (d, J_{RhP} = 152 Hz), 75.5 (d, J_{RhP} = 165 Hz). Orientation 2**:** δ 81.9 (d, J_{RhP} = 147 Hz), 74.1 (d, J_{RhP} = 150 Hz).

$^{13}C\{^1H\}$ SSNMR (101 MHz, 294 K, 10 kHz spin rate): δ 164.7 (*ipso*-ArC), 134.5 (*ortho*-ArC), 129.7 (*meta*-ArC), 124.4 (br, -CF_3), 120.5 (C=C), 117.3 (*para*-ArC), 116.1 (*para*-ArC), 104.3 (C=C), 103.0 (C=C), 96.4 (C=C), 94.3 (C=C), 91.6 (C=C), 88.0 (C=C), 60.4 (C=C), 38.7 – 18.7 (m, overlapping cyclohexyl-CH_2).

Note: Peaks that are assigned (C=C) as they disappear upon hydrogenation of the sample (see Figure S10).

ESI-MS (calc. for $[Rh(Cy_2PCH_2CH_2PCy_2)(C_5H_8)]^+$): *m/z* 593.2912 (593.2907).
Elemental Analysis (calc. for $C_{63}H_{68}BF_{24}P_2Rh$): C 52.06 (51.94), H 4.50 (4.70).

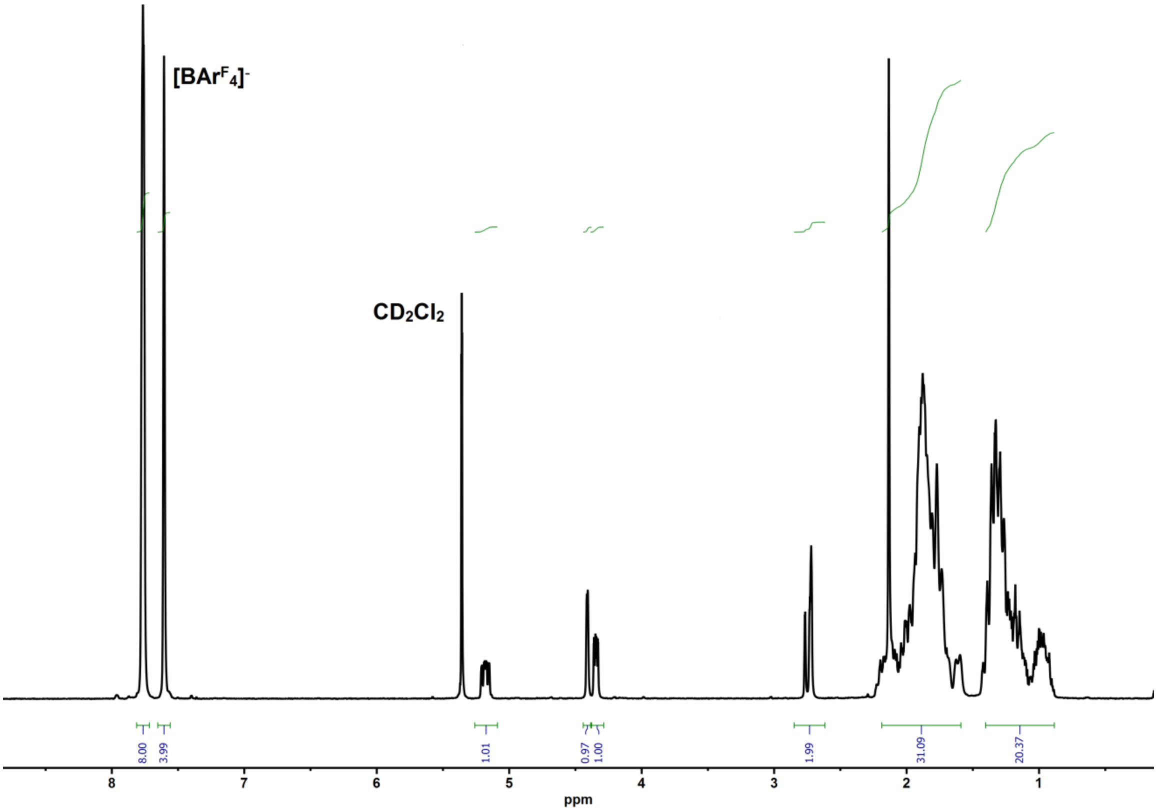

Figure S5: The solution ^{1}H NMR spectrum (CD_2Cl_2, 298 K, 400 MHz) of **[1-isoprene][BAr$^F_4$]**.

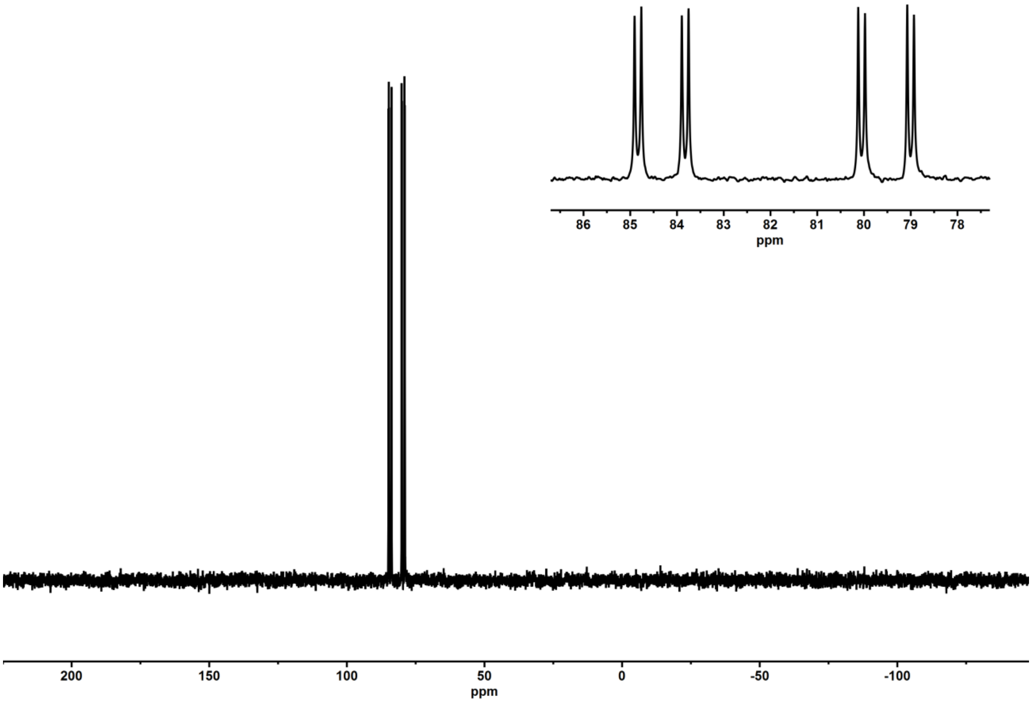

Figure S6: The solution $^{31}P\{^1H\}$ NMR spectrum (CD_2Cl_2, 298 K, 162 MHz) of **[1-isoprene][BAr$^F_4$]**. The inset is an enlargement of the resonances between δ 85 and 78.

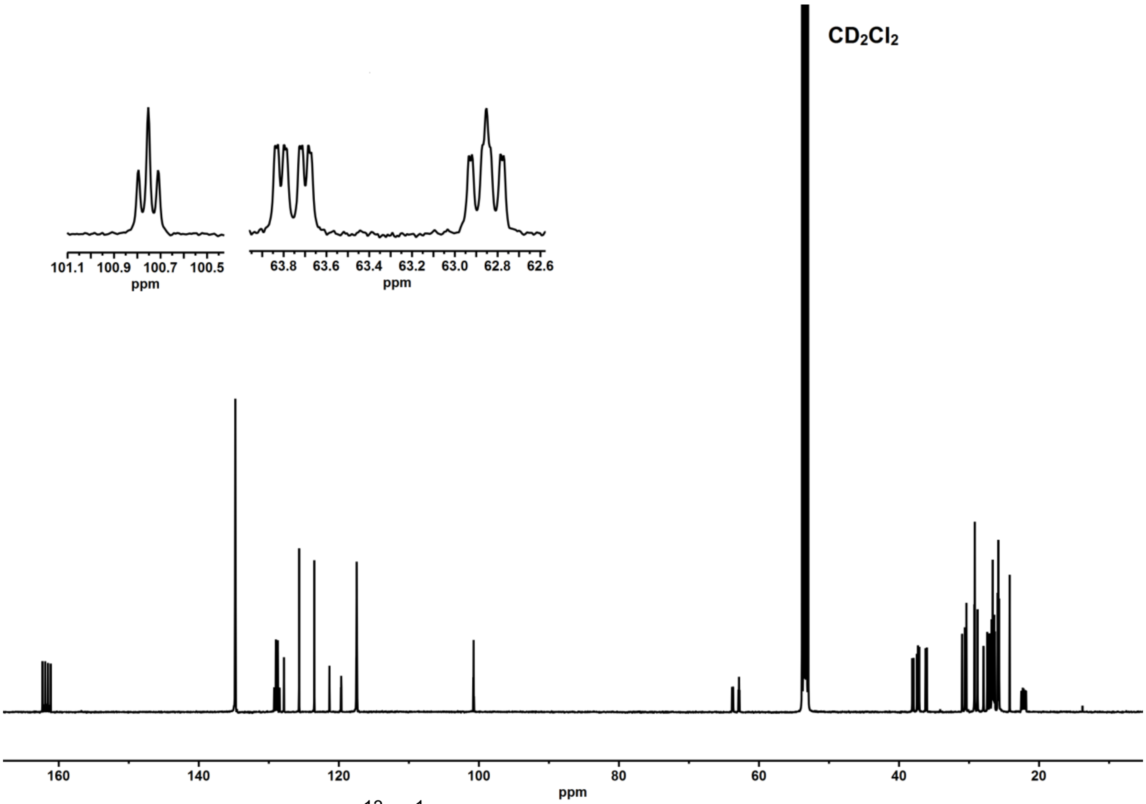

Figure S7: The solution $^{13}C\{^1H\}$ NMR spectrum (CD_2Cl_2, 298 K, 126 MHz) of **[1-isoprene][BAr^F$_4$]**. The inset is an enlargement of the resonances between δ 101 and 62.

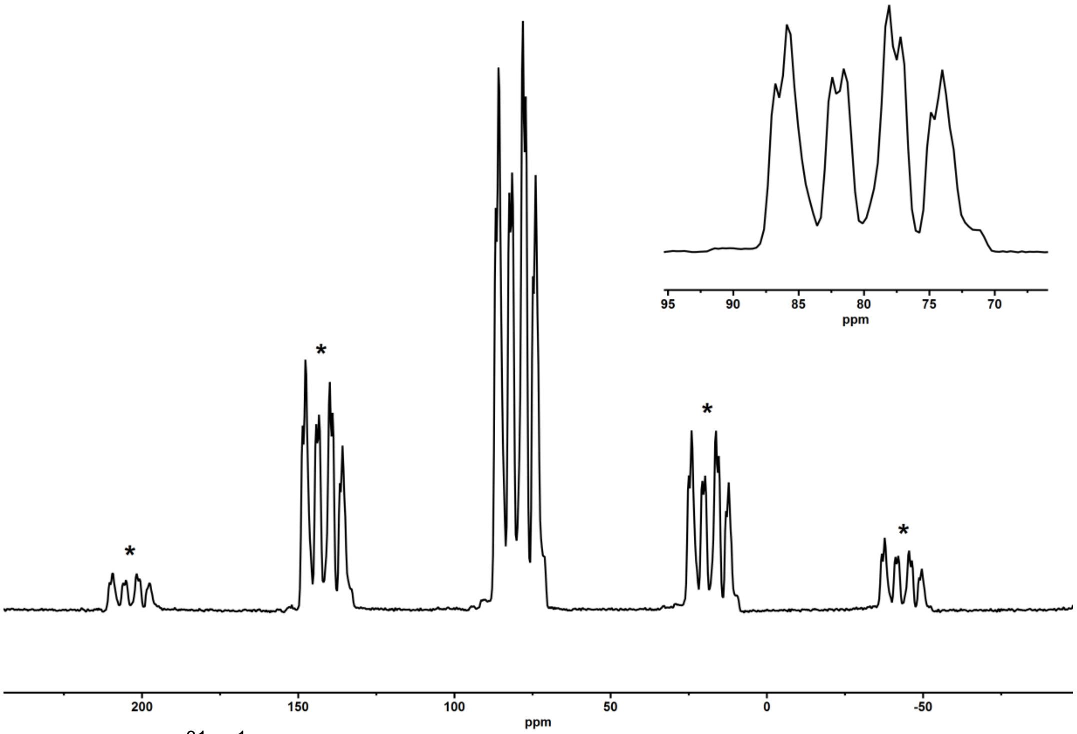

Figure S8: The $^{31}P\{^1H\}$ SSNMR spectrum (162 MHz, 294 K, 10 kHz spin rate) of **[1-isoprene][BAr^F$_4$]**. The resonances marked * are due to spinning sidebands. The inset is an enlargement of the resonances between δ 101 and 62.

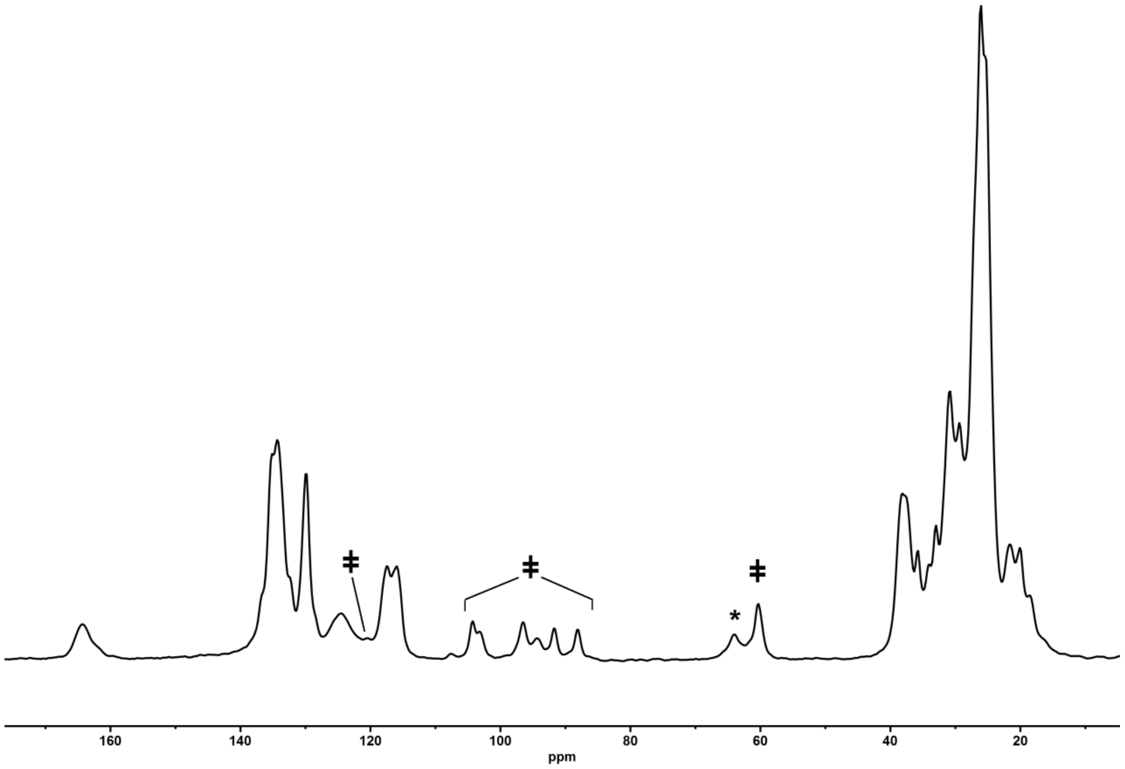

Figure S9: The $^{13}C\{^{1}H\}$ SSNMR spectrum (101 MHz, 298 K, 10 kHz spin rate) of **[1-isoprene][BAr$^{F}_{4}$]**. The resonances marked * are due to spinning sidebands. The alkene resonances have been assigned ‡.

S.2.2.3. Synthesis of a 2-methylbutane complex, [1-(2-methylbutane)][BAr$^F{}_4$]

For Single-Crystal X-Ray Diffraction: Ruby red crystals of **[1-isoprene][BAr$^F{}_4$]** (1.5 mgs) were placed in a J. Young NMR tube and treated with H_2 (1 bar, 298 K, 25 minutes). The colour of the crystals slowly turned a dark red over this time. The crystalline material was then removed from the tube and coated with Fomblin® Y oil under an argon flush. A suitable crystal was then selected and transferred to the cryostream of a diffractometer (150 K). A X-ray diffraction study was undertaken and the structure of [Rh($Cy_2PCH_2CH_2PCy_2$)(C_5H_{12})][BAr$^F{}_4$] **[1-(2-methylbutane)][BAr$^F{}_4$]** was refined. For further details of structural refinement please refer to Section S.3.

For Solid-State NMR Spectroscopy: A powdered microcrystalline sample of **[1-isoprene][BAr$^F{}_4$]** (45 mgs) was packed in a 4.0 mm SSNMR rotor, inside an argon filled glove box. The rotor was then placed in a custom-built glass J. Young flask[S5] and the sample was then exposed to H_2 (1 bar, 298 K). After 20 minutes, the rotor cap was fitted under a flush of H_2. The sample was rapidly transferred to the bore of a SSNMR spectrometer, cooled to 158 K (~10 minutes) and analysed by $^{31}P\{^1H\}$ and $^{13}C\{^1H\}$ solid-state NMR spectroscopy.

Note: These conditions were optimised to form **[1-(2-methylbutane)][BAr$^F{}_4$]**. At 158 K, no onward reactivity with H_2 is observed. When H_2 is replaced by argon at 298 K, as so the rotor is packed under an argon atmosphere, rapid dehydrogenation to form a mixture of **[1-methylbutenes][BAr$^F{}_4$]** isomers are observed before cooling to 158 K, see Section S.2.4. Further exposure to H_2 (90 minutes) at 298 K results in complete decomposition to **[1-BAr$^F{}_4$]**.[S2]

S.2.2.4. Charaterisation data and spectra for [1-(2-methylbutane)][BAr$^F{}_4$]

$^{31}P\{^1H\}$ SSNMR (162 MHz, 158 K, 10 kHz spin rate): δ 106.9 (d, J_{RhP} = 204 Hz), 104.1 (d, J_{RhP} = 220 Hz).

$^{13}C\{^1H\}$ SSNMR (101 MHz, 158 K, 10 kHz spin rate): δ 163.3 (*ipso*-ArC), 134.1 (*ortho*-ArC), 128.9 (*meta*-ArC), 123.7 (br, -CF_3), 116.6 (*para*-ArC), 39.9, 36.6, 34.8, 31.1, 30.1, 28.3, 25.0, 20.2, 17.3, 13.3, 10.1, 8.42 (multiple aliphatic resonances).

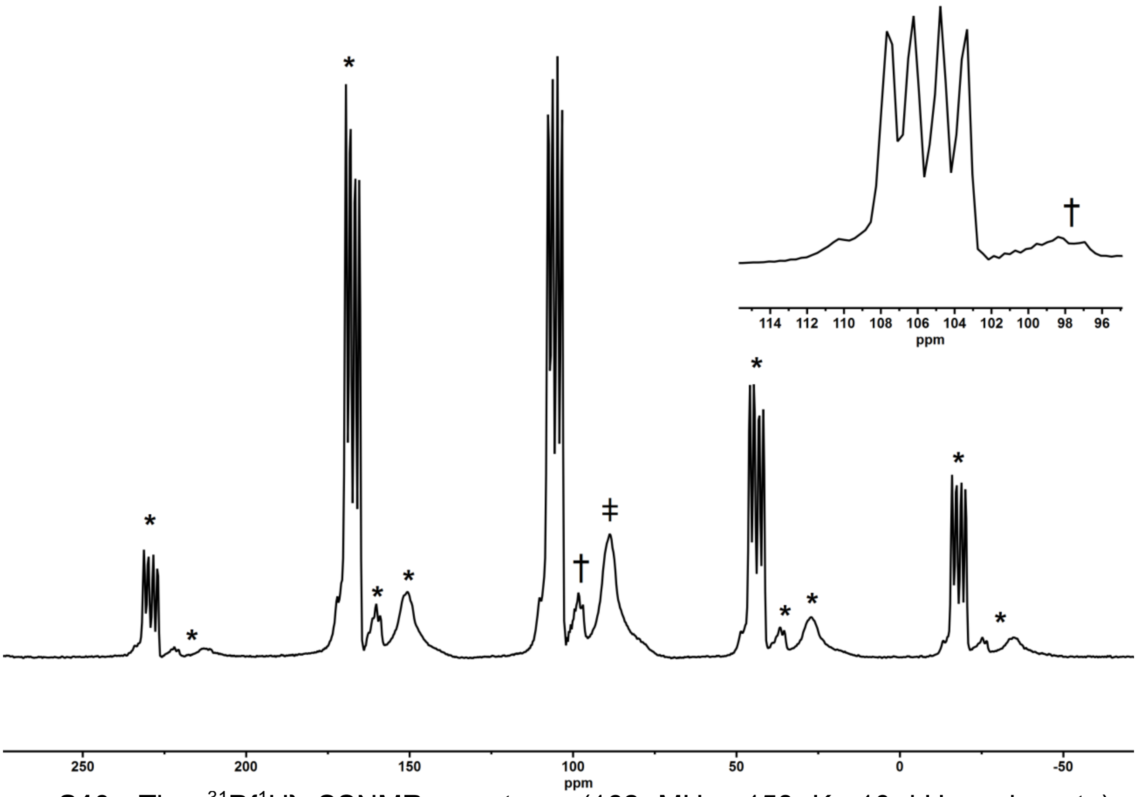

Figure S10: The $^{31}P\{^{1}H\}$ SSNMR spectrum (162 MHz, 158 K, 10 kHz spin rate) of **[1-(2-methylbutane)][BAr$^F_4$]**. Peaks marked * are spinning sidebands, ‡ are assigned to **[1-BAr$^F_4$]**[S2] and † to **[1-methylbutenes][BAr$^F_4$]**. The inset is an enlargement of the resonances between δ 114 and 96.

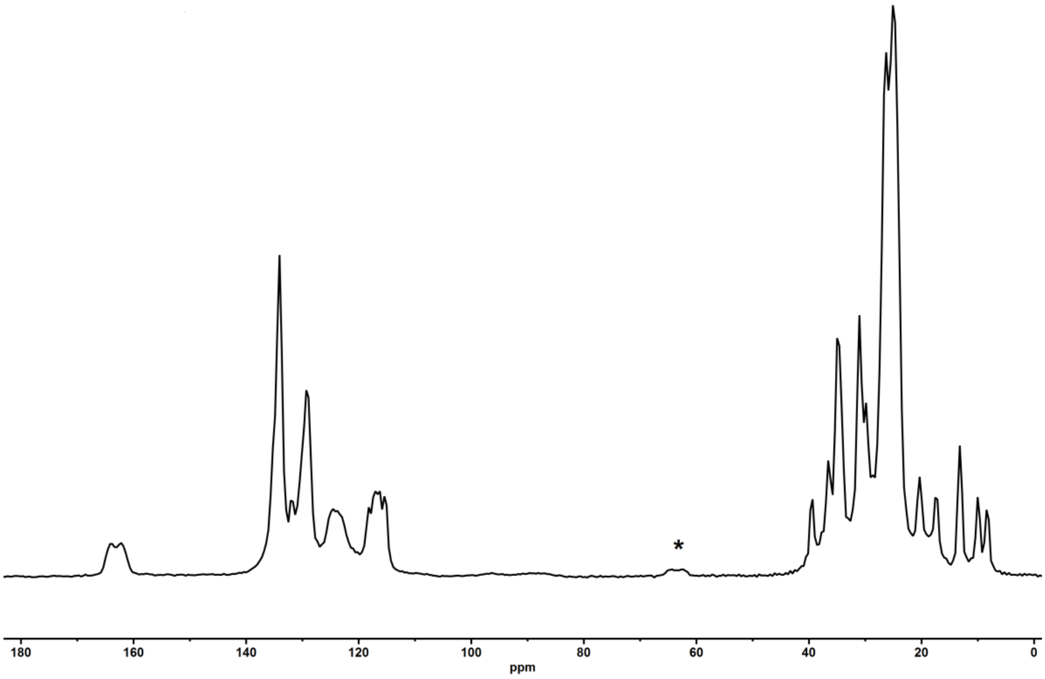

Figure S11: The $^{13}C\{^{1}H\}$ SSNMR spectrum (101 MHz, 158 K, 10 kHz spin rate) of **[1-(2-methylbutane)][BAr$^F_4$]**. The resonances marked * are due to spinning sidebands.

S.2.3. $[Rh(Cy_2PCH_2CH_2PCy_2)(C_5H_{10})][BAr^F{}_4]$, **[1-methylbutenes][BAr$^F{}_4$]**

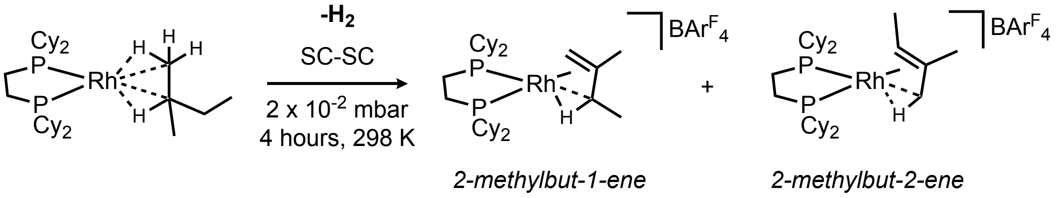

[1-(2-methylbutane)][BAr$^F{}_4$] **[1-methylbutenes][BAr$^F{}_4$]**

S.2.3.1. Synthesis of a methylbutenes complex, [1-methylbutenes][BAr$^F{}_4$]

For Solid-State NMR Spectroscopy and Single-Crystal X-Ray Diffraction: A J. Young flask (~50 mL) was charged with ruby red crystals of **[1-isoprene][BAr$^F{}_4$]** (100 mgs) and placed under an atmosphere of H_2 (1 bar, 298 K, 25 minutes). The colour of this material slowly turned dark red within this time. The powder was then exposed to a dynamic Schlenk line vacuum (< 2 × 10^{-2} mbar, optimised at 4 hours) to yield $[Rh(Cy_2PCH_2CH_2PCy_2)(C_5H_{10})][BAr^F{}_4]$, **[1-methylbutenes][BAr$^F{}_4$]**. The now yellow-orange powder was then taken into an argon filled glove box and stored at –25 °C.

Note: This dehydrogenation reaction was also found to occur under a flushing argon flow and was complete within 6 hours (unoptimised).

A single-crystal X-ray diffraction experiment was conducted on a sample of these crystals and for further details of the structural refinement please refer to Section S.3. A sample of this material (45 mgs) was also packed in a 4.0 mm SSNMR rotor and analysed by $^{31}P\{^1H\}$ and $^{13}C\{^1H\}$ solid-state NMR spectroscopy at 294 and 158 K.

S.2.3.2. Charaterisation data and spectra for [1-methylbutenes][BAr$^F_4$]

^{1}H NMR (CD_2Cl_2, 298 K, 400 MHz): δ 7.76 (s, 8H, *ortho*-ArH), 7.61 (s, 4H, *para*-ArH), 2.05-1.68 (br m, overlapping aliphatic CH), 1.61-0.85 (br m, aliphatic CH).

^{1}H NMR (CD_2Cl_2, 183 K, 400 MHz): δ 7.77 (s, 8H, *ortho*-ArH), 7.58 (s, 4H, *para*-ArH), 5.08 (s, 2H, C=C**H**CH_3 from 2-methylbut-2-ene isomer), 4.33 (s, 1H, C=C**H_2** from 2-methylbut-1-ene isomer), 3.29 (s, 1H, C=C**H_2** from 2-methylbut-1-ene isomer), 2.14-0.78 (br m, overlapping aliphatic CH), -0.25, (s, 3H, agostic from 2-methylbut-2-ene isomer), –1.23 (s, 1H, agostic from 2-methylbut-1-ene isomer).

Note: The low temperature limit was not reached at 183 K in the ^{1}H NMR spectrum, so integrated values are approximate. The assignments for the above data have been made from ^{1}H / ^{1}H COSY analysis upon the same sample, as well as comparison to the similar, previously reported isobutene[S7] and 2-butene[S5] coordinated complexes. NMR samples were prepared under argon and immediately run after preparation.

The ratio of coordinated 2-methylbut-1-ene: 2-methylbut-1-ene isomer is 1: 1.34 (Figure S16).

^{31}P{^{1}H} NMR (CD_2Cl_2, 298 K, 162 MHz): δ 94.4 (d, J_{RhP} 181.7 Hz).
^{31}P{^{1}H} NMR (CD_2Cl_2, 183 K, 162 MHz): 2-methylbut-2-ene Isomer: δ 96.6 (d of d, J_{RhP} = 199.6 Hz, J_{PP} = 25 Hz), 93.9 (d of d, J_{RhP} = 155.9 Hz, J_{PP} = 25 Hz). 2-methylbut-1-ene Isomer*:* δ 95.2 (d of d, J_{RhP} = 152.6 Hz, J_{PP} = 26 Hz), 93.9 (d of d, J_{RhP} = 212.0 Hz, J_{PP} = 26 Hz).

^{19}F{^{1}H} NMR (CD_2Cl_2, 298 K, 377 MHz): δ -62.9 (s).

^{13}C{^{1}H} NMR (CD_2Cl_2, 183 K, 126 MHz): δ 161.6 (q, J_{CB} = 50 Hz, *ipso*-ArC), 134.6 (s, *ortho*-ArC), 128.6 (q, J_{CF} = 32 Hz, *meta*-ArC), 124.5 (q, J_{CF} = 272 Hz, -CF_3), 119.1 (s, alkene-CH), 117.6 (s, *para*-ArC), 104.8 (s, alkene-CH), 86.4 (s, alkene-CH), 72 (br., alkene-CH), 37.4 to 19.8 (multiple aliphatic resonances from both isomers), 17.4, 17.2, 14.5, 13.7, 12.4, 11.7.

^{31}P{^{1}H} SSNMR (162 MHz, 294 K, 10 kHz spin rate): δ 95.4 (br, apparent triplet).
^{31}P{^{1}H} SSNMR (162 MHz, 158 K, 10 kHz spin rate): δ 94.6 (br, apparent triplet).

$^{13}C\{^{1}H\}$ SSNMR (101 MHz, 294 K, 10 kHz spin rate): δ 163.8 (*ipso*-ArC), 134.5 (*ortho*-ArC), 130.1 (*meta*-ArC), 124.6 (br, CF_3), 118.2 (*para*-ArC), 115.5 (*para*-ArC), 38.4 (CH), 35.9 (CH), 30.6 (CH_2), 21.7 (CH_2), 13.1 (CH_2).

$^{13}C\{^{1}H\}$ SSNMR (101 MHz, 158 K, 10 kHz spin rate): δ 163.9 (*ipso*-ArC), 133.9 (*ortho*-ArC), 129.4 (*meta*-ArC), 124.2 (br, CF3), 117.7 (*para*-ArC), 114.9 (*para*-ArC), 106.5 (C=C), 87.6 (C=C), 69.5 (C=C, 1+1 coincidence), 38.8 (CH), 36.6 (CH), 30.8 (CH_2), 26.6 (CH_2), 24.7 (CH_2), 19.2 (CH_2), 16.8 (CH_2), 12.6 (CH_2).

ESI-MS: Not stable under the conditions during mass spectrometry (20 eV, 333 K). Species with appropriate isotopic distributions at *m/z* found = 581.240, calculated to $[(Cy_2PCH_2CH_2PCy_2)Rh(N_2)_2]^+$ (581.242). There is no evidence for the nitrogen compound in bulk samples so it is assumed to form *via* an *in situ* ESI-MS process.

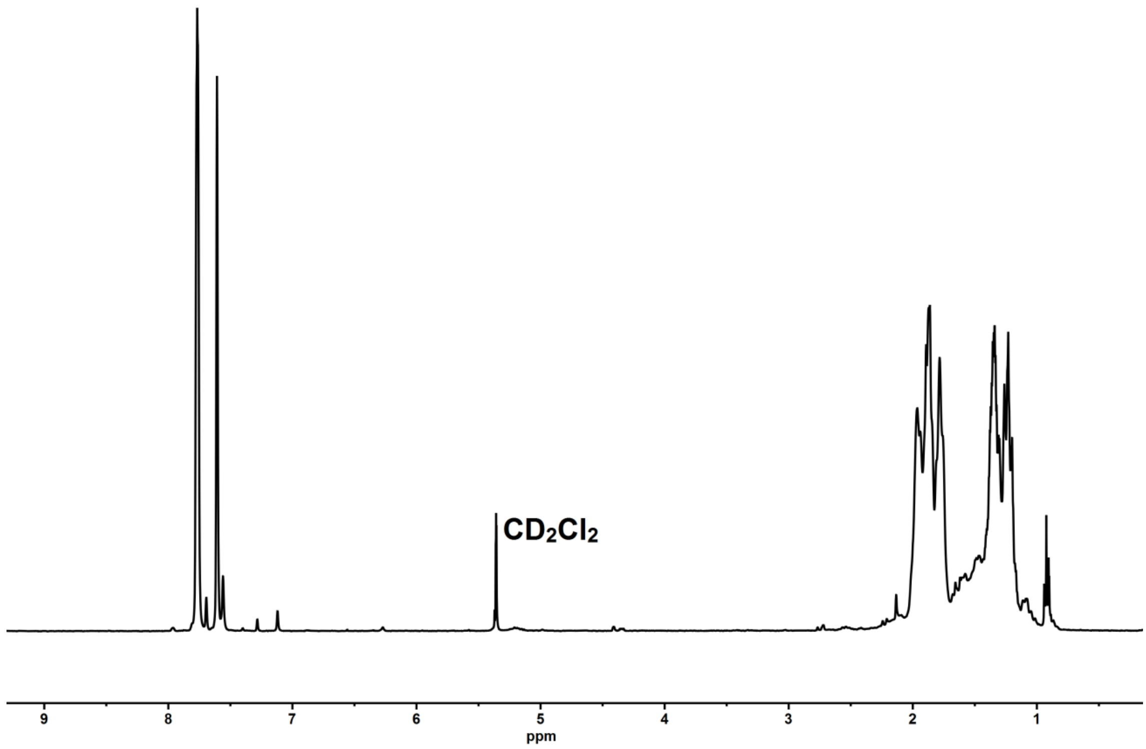

Figure S12: The solution ^{1}H NMR spectrum (CD_2Cl_2, 298 K, 400 MHz) of **[1-methylbutenes][BAr^F$_4$]**.

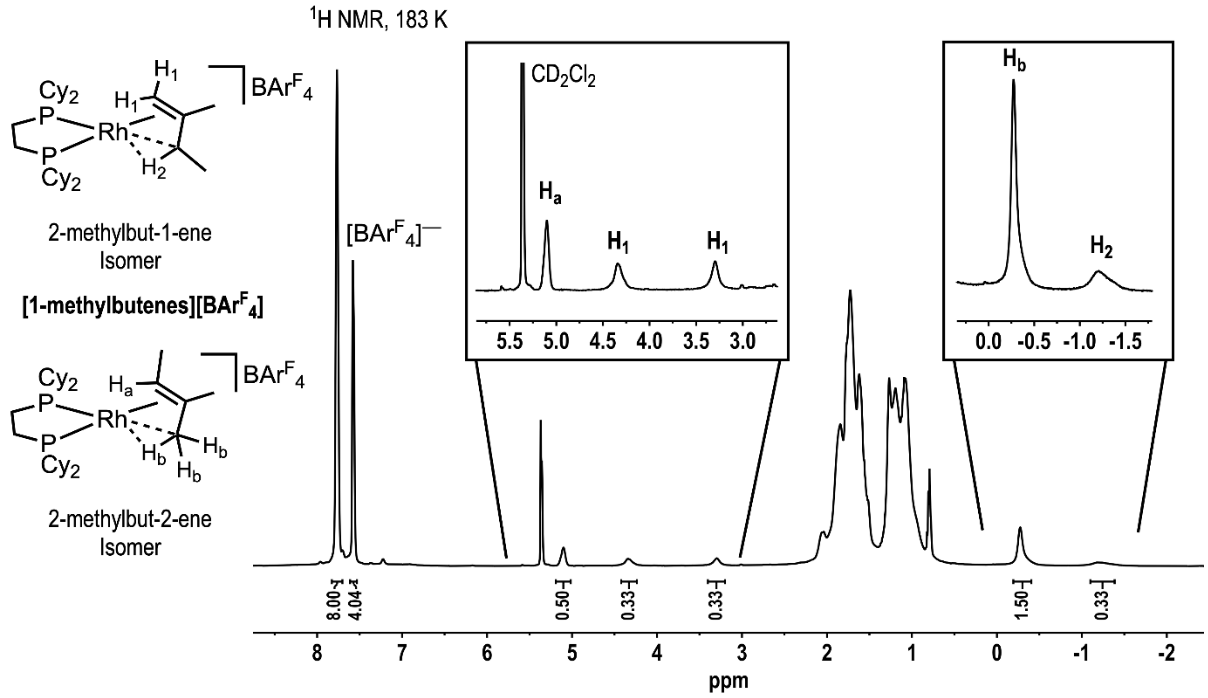

Figure S13: The solution ^{1}H NMR spectrum (CD_2Cl_2, 183 K, 400 MHz) of **[1-methylbutenes][BAr^F$_4$]**. The inserts show enlargements of the peaks between δ 5.5 and 3.0 and 0.00 to -1.23. The ratio of 2-methylbut-1-ene: 2-methylbut-2-ene isomers is 1: 1.34, from the integration of the signals of H_1:H_a.

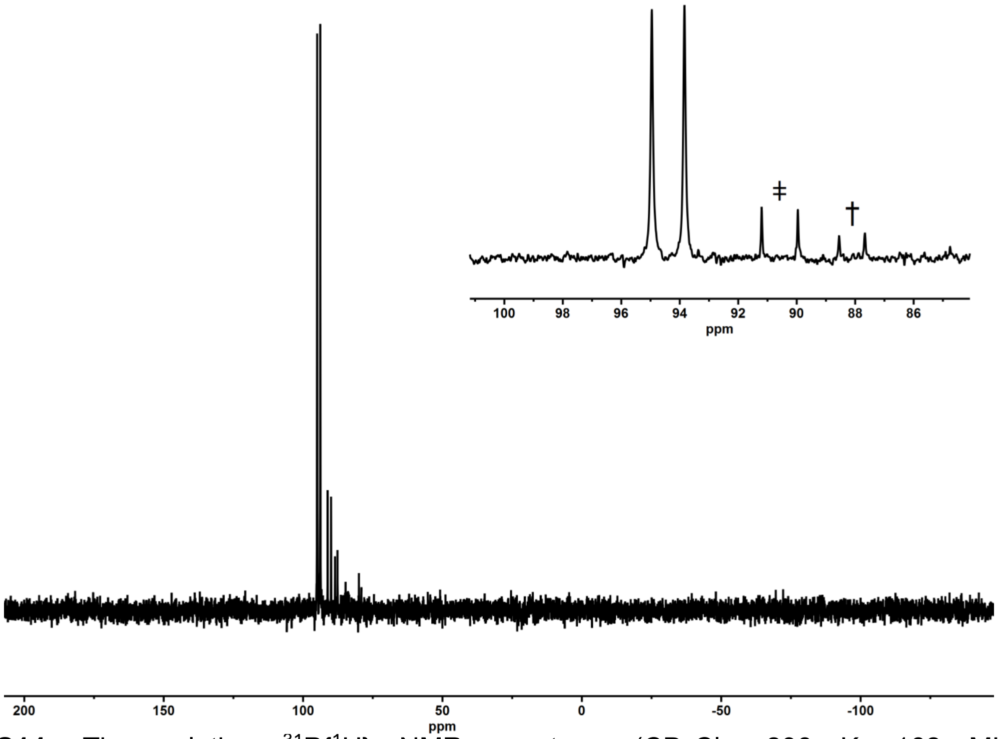

Figure S14: The solution $^{31}P\{^{1}H\}$ NMR spectrum (CD_2Cl_2, 298 K, 162 MHz) of **[1-methylbutenes][BAr$^F_4$]**. The inset is an enlargement of the resonances between δ 100 and 86. ‡ is of **[1-BAr$^F_4$]**,[S2] formed as a minor product during the initial hydrogenation reaction. The signal marked † is from a further decomposition product of **[1-BAr$^F_4$]** in solution.[S2]

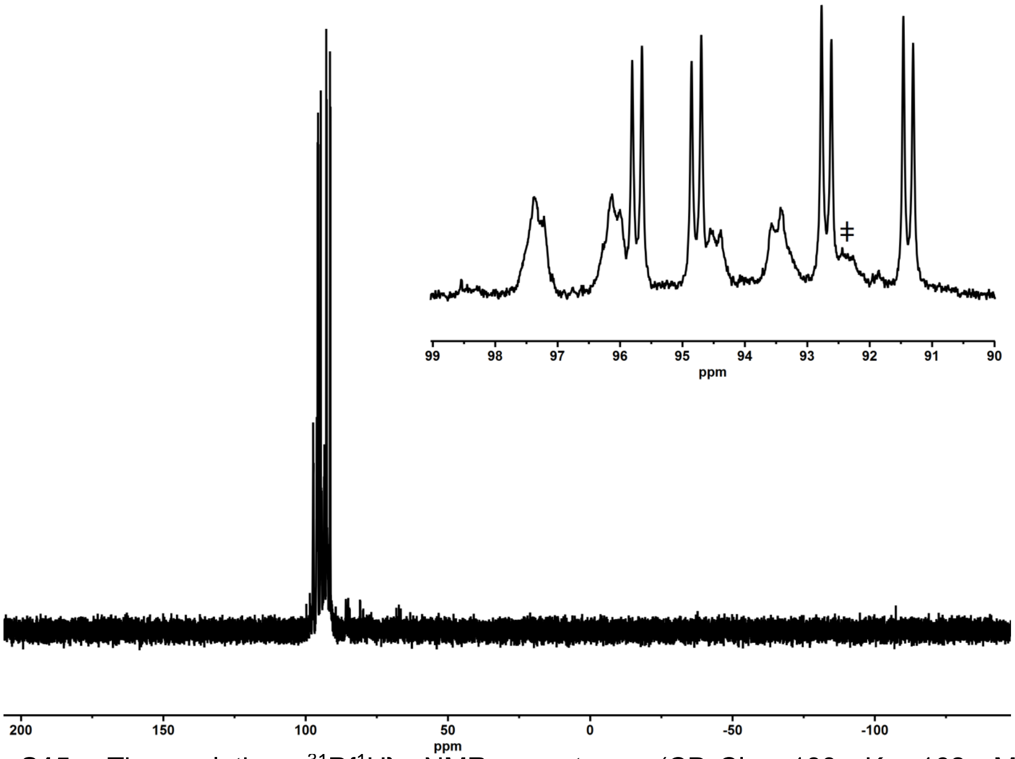

Figure S15: The solution $^{31}P\{^{1}H\}$ NMR spectrum (CD_2Cl_2, 183 K, 162 MHz) of **[1-methylbutenes][BAr$^F_4$]**. The inset is an enlargement of the resonances between δ 99 and 90. The signal marked ‡ is from a further decomposition product of **[1-BAr$^F_4$]** in solution.[S2]

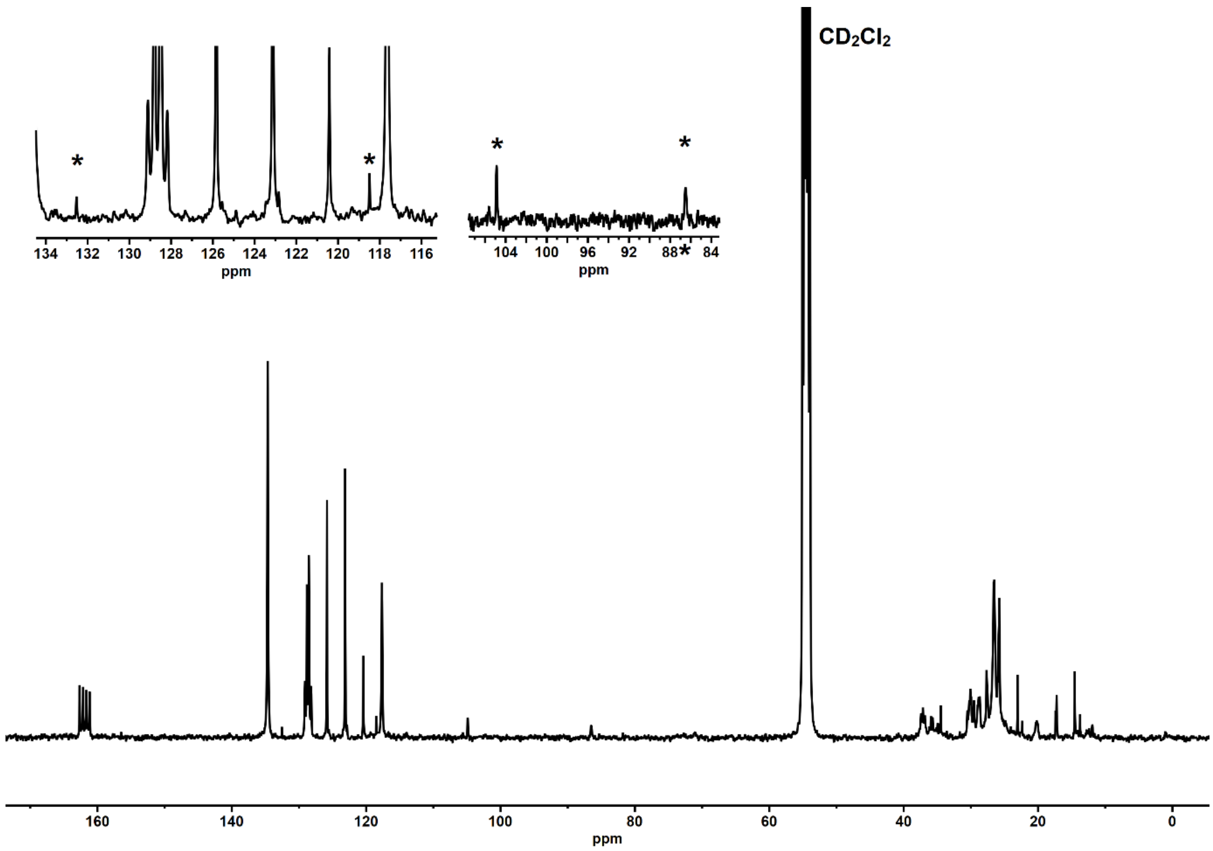

Figure S16: The solution $^{13}C\{^1H\}$ NMR spectrum (CD_2Cl_2, 183 K, 126 MHz) of **[1-methylbutenes][BAr$^F_4$]**. The inset is an enlargement of the alkene resonances between δ 134 to 116 and 104 to 84. Signals assigned to alkene carbons labelled *.

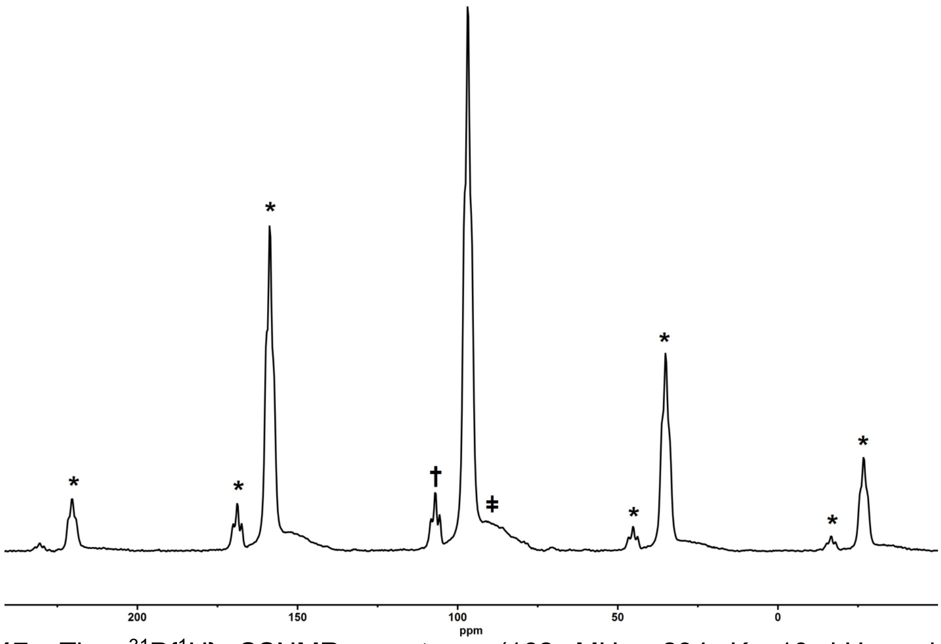

Figure S17: The $^{31}P\{^{1}H\}$ SSNMR spectrum (162 MHz, 294 K, 10 kHz spin rate) of **[1-methylbutenes][BAr^F$_4$]** isomers. Peaks marked * are spinning sidebands, ‡ are assigned to **[1-BAr^F$_4$]**[S2] and † to an unidentified, previously noted, decomposition product under an hydrogen atmosphere.[S7]

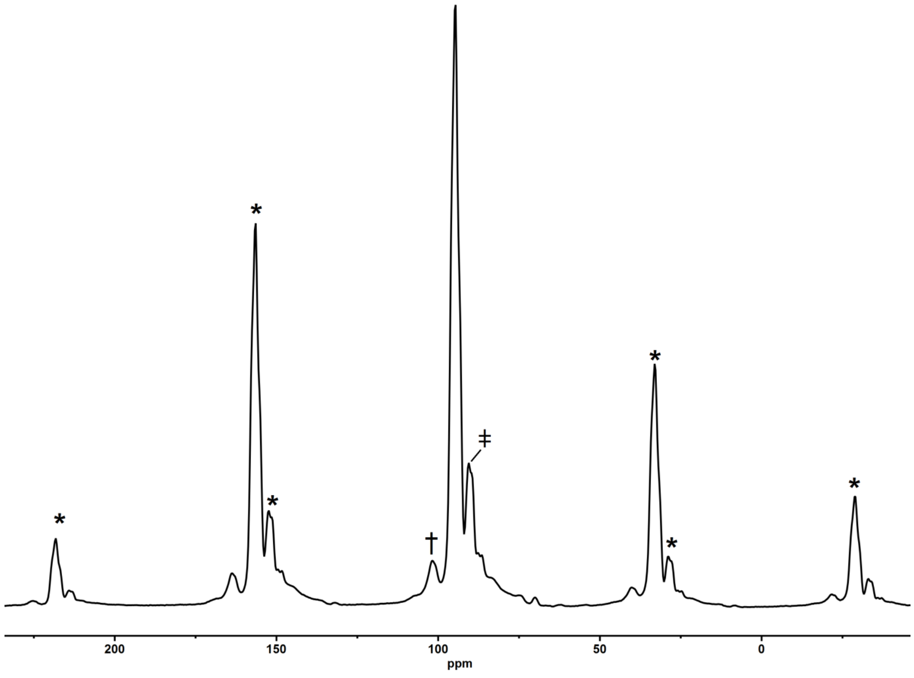

Figure S18: The $^{31}P\{^{1}H\}$ SSNMR spectrum (162 MHz, 158 K, 10 kHz spin rate) of **[1-methylbutenes][BAr^F$_4$]** isomers. Peaks marked * are spinning sidebands, ‡ is assigned to **[1-BAr^F$_4$]**,[S2] but we cannot rule out this from a static **[1-methylbutenes][BAr^F$_4$]** isomer. † is assigned to an unidentified, previously noted, decomposition product under a hydrogen atmosphere.[S7] Note: The spectrum in Figures S20 to S21 were obtained from a different sample to that of Figures S13 and S14 resulting in a different degree of decomposition to **[1-BAr^F$_4$]**.

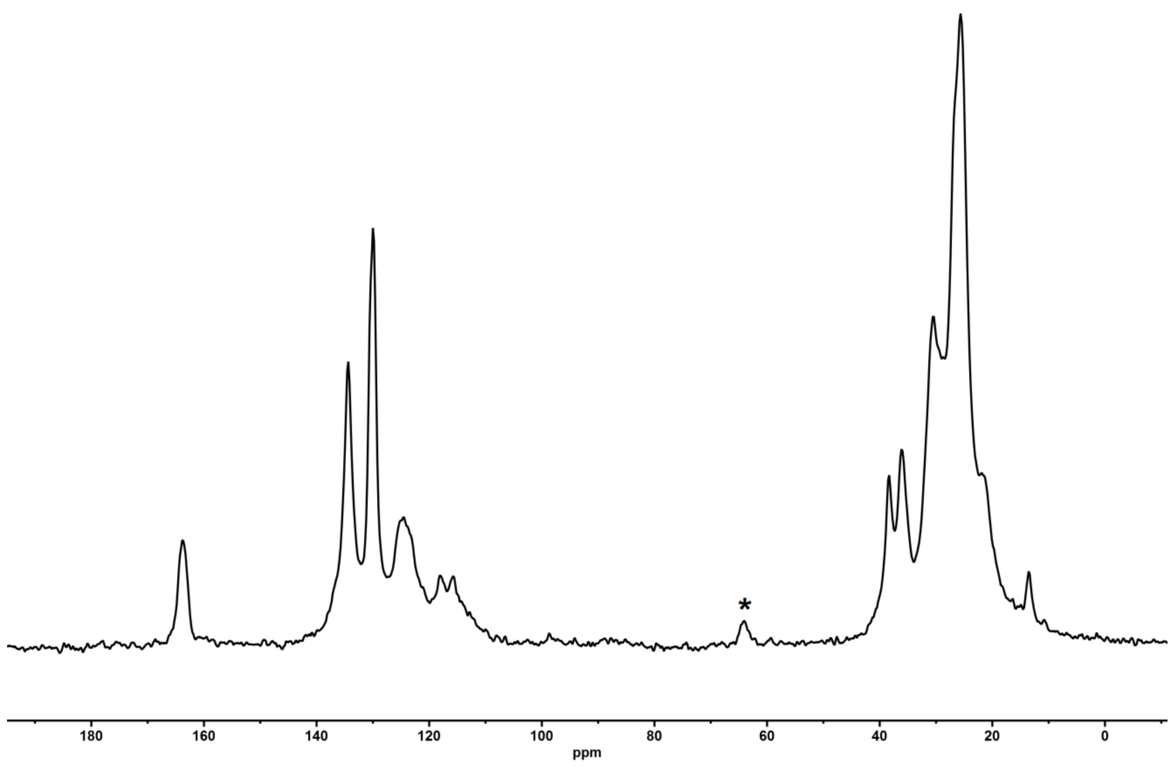

Figure S19: The $^{13}C\{^1H\}$ SSNMR spectrum (101 MHz, 294 K, 10 kHz spin rate) of **[1-methylbutenes][BAr$^F_4$]**. The resonances marked * are due to spinning sidebands.

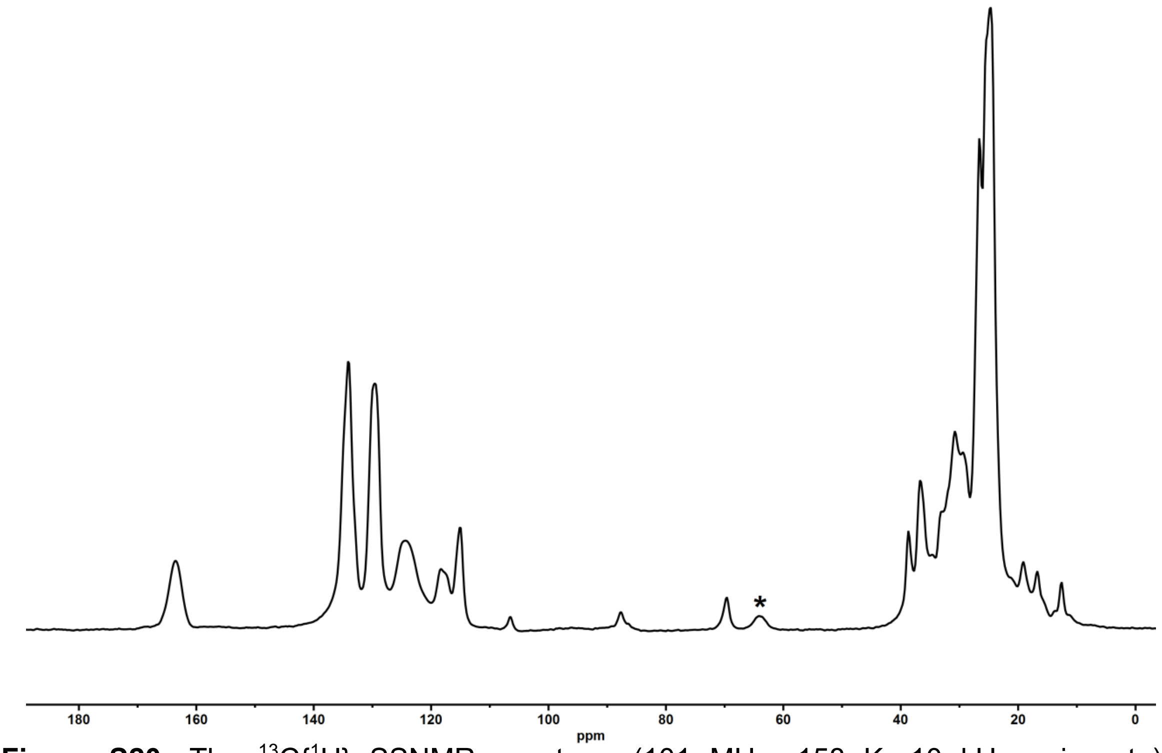

Figure S20: The $^{13}C\{^1H\}$ SSNMR spectrum (101 MHz, 158 K, 10 kHz spin rate) of **[1-methylbutenes][BAr$^F_4$]**. The resonances marked * are due to spinning sidebands.

S.2.3.3. Liberation of bound methylbutenes from [1-methylbutenes][BAr$^F_4$]

A J. Young flask was charged with a sample of **[1-methylbutenes][BAr$^F_4$]** (30 mgs), as prepared from the method in Section S.2.4.1. The flask was placed under vacuum (< 2 × 10^{-2} mbar) and backfilled with CO (1 bar, 298 K). After 18 hours, the orange crystalline material had turned a pale-yellow colour. The resultant solid was confirmed to be [Rh($Cy_2PCH_2CH_2PCy_2$)$(CO)_2$][BAr$^F_4$] by solution $^{31}P\{^1H\}$ NMR spectroscopy (δ 85.22, d, J_{RhP} = 116 Hz).[S5] The volatile component was then isolated by trap-to-trap distillation into CD_2Cl_2 and analysed by solution ^1H NMR spectroscopy.

2-methylbut-1-ene: **^{1}H NMR ($CDCl_3$, 298 K, 400 MHz):** δ 4.66 (s., 2H), 2.02 (quartet, 2H, J_{HH} = 7.5 Hz), 1.73 (s., 3H), 1.03 (triplet, 3H, J_{HH} = 7.5 Hz).
2-methylbut-2-ene: **^{1}H NMR (CH_2Cl_2, 298 K, 500 MHz):** δ 5.18 (m., 1H), 1.67 (s., 3H), 1.60 (s., 3H), 1.55 (d., 3H, J_{HH} = 6.7 Hz).

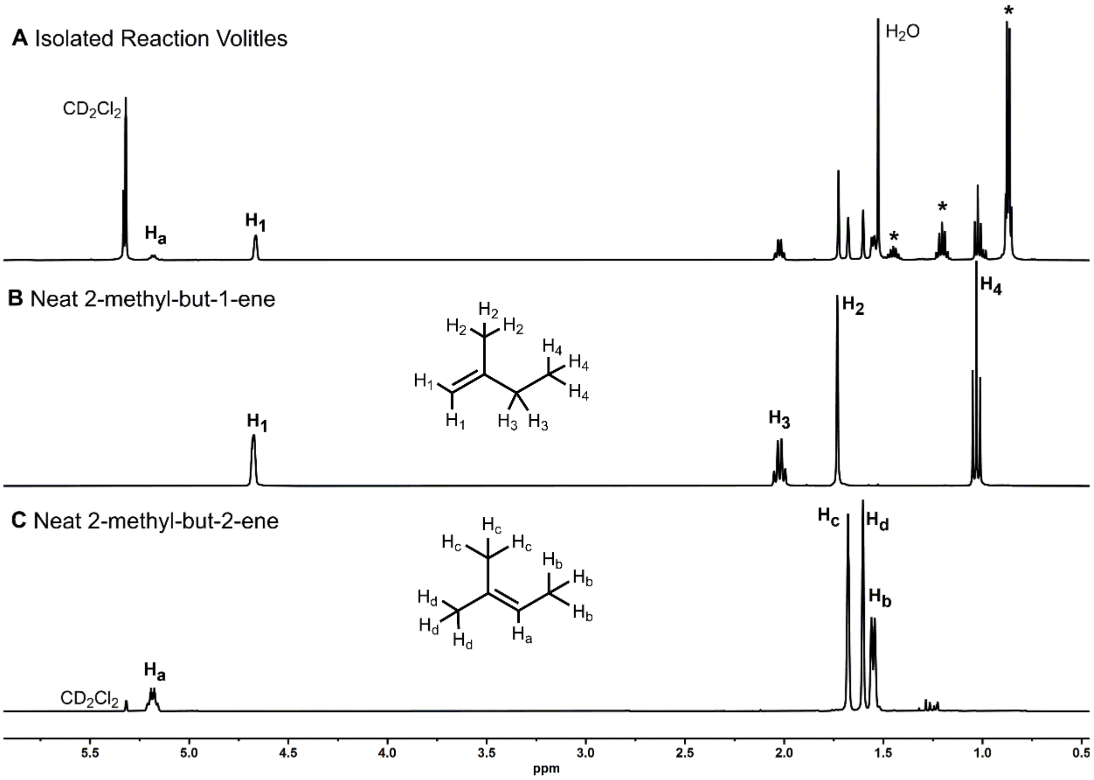

Figure S21: The solution ^{1}H NMR spectra of **A**: (CD_2Cl_2, 298 K, 500 MHz) liberated reaction volatiles showing mix of methylbutene isomers. Peaks marked * are from 2-methylbutane from alkane liberation from sample decomposition during the initial hydrogenation. **B**: ($CDCl_3$, 298 K, 400 MHz) Neat 2-methyl-but-1-ene. **C**: (CD_2Cl_2, 298 K, 400 MHz) Neat 2-methylbut-2-ene. Note: The integration of the H_1: H_a resonances (from spectrum A) is **2.79: 1**. This suggests a **1.4: 1** ratio of liberated 2-methylbut-1-ene: 2-methylbut-2-ene.

S.2.3.4. Reaction kinetics of the dehydrogenation of [1-(2-methylbutane)][BAr$^F_4$]

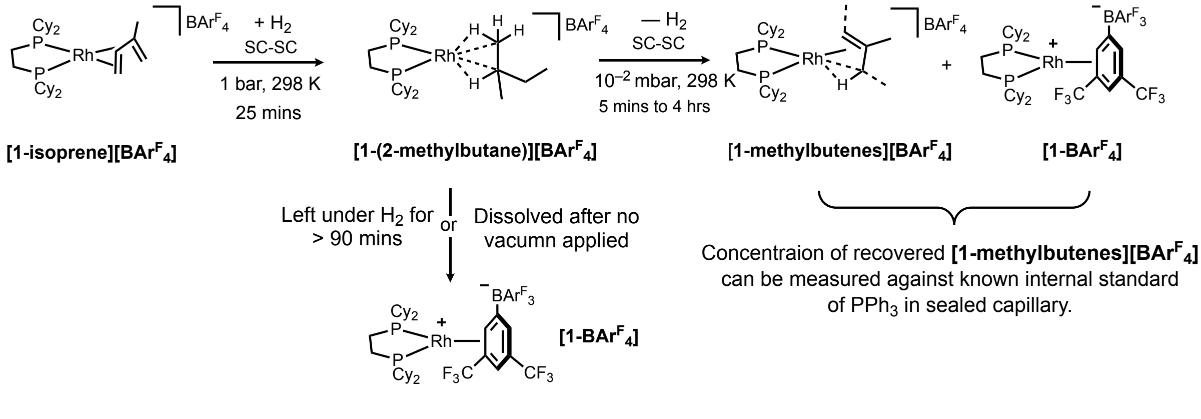

Procedure: Nine J. Young NMR tubes (~2.1 mL) were individually charged with 12.5 mgs (8.59 μmol) of powdered crystalline **[1-isoprene][BAr$^F_4$]** and a separate sealed capillary containing PPh_3 in acetone. The tubes were evacuated (< 2 × 10^{-2} mbar) and H_2 (1 bar, 298 K) was added for 25 minutes, to yield the dark red powder of **[1-(2-methylbutane)][BAr$^F_4$]**. These samples were placed under vacuum (< 2 × 10^{-2} mbar) for varying times between 0 seconds and 4 hours at room temperature, then dissolved in CH_2Cl_2 (0.35 mL) and analysed by solution $^{31}P\{^1H\}$ NMR spectroscopy.

When time = 0 seconds, no signals relating to **[1-isoprene][BAr$^F_4$]** could be identified, confirming the complete hydrogenation of the sample. When left under vacuum for greater than 4 hours (with longest time point of 7 days), no signals relating to **[1-isoprene][BAr$^F_4$]** were identified confirming only the selective dehydrogenation to **[1-methylbutenes][BAr$^F_4$]**.

After dissolution in CH_2Cl_2, two major signals were observed in the $^{31}P\{^1H\}$ NMR spectra at 298 K, of the dehydrogenation product **[1-methylbutenes][BAr$^F_4$]** (δ 94, J_{RhP} = 179 Hz) and decomposition product **[1-BAr$^F_4$]** (δ 91, J_{RhP} = 201 Hz). The concentration of recovered **[1-methylbutenes][BAr$^F_4$]** could be accurately measured against the integration of the PPh_3 signal.

No more than 82 % recovered **[1-methylbutenes][BAr$^F_4$]** could be achieved relative to the initial **[1-isoprene][BAr$^F_4$]** loading. This suggests a consistent ~20 % decomposition to **[1-BAr$^F_4$]** during the initial 25 minutes hydrogenation time. This ~20 % decomposition is also observed in the $^{31}P\{^1H\}$ SSNMR spectrum of **[1-(2-methylbutane)][BAr$^F_4$]** (Figure S13).

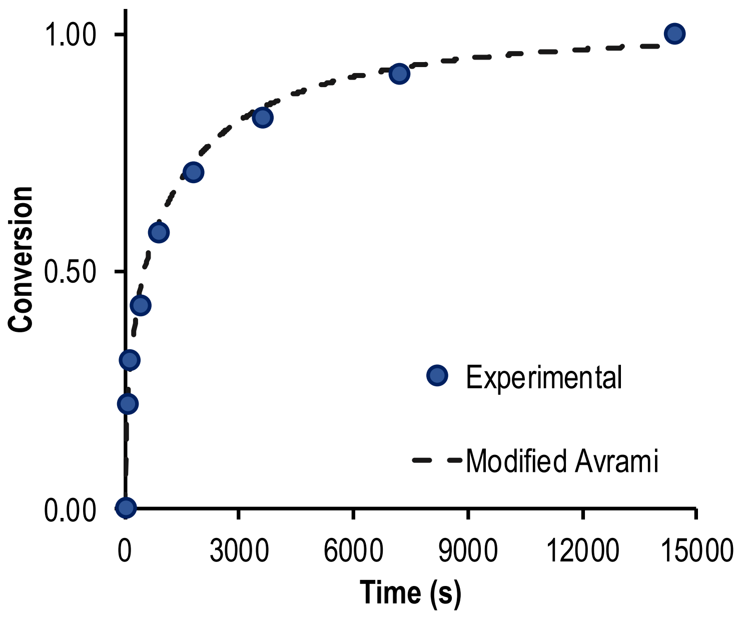

Figure S22: Temporal profile for the dehydrogenation of **[1-(2-methylbutane)][BAr^F$_4$]**. Each data point represents an individual dehydrogenation experiments and are shown by blue markers. These plots have been modelled to take into account the 20 % systematic decomposition of **[1-(2-methylbutane)][BAr^F$_4$]** observed during the initial hydrogenation (See Section S.2.3.3). The maximum concentration formed of **[1-methylbutenes][BAr^F$_4$]** is 20.16 mM.

Overlaid curve represents the modified JAMK plot of conversion[S8] (f) = ($1–e^{(–kt)^n}$) versus time for the dehydrogenation of **[1-(2-methylbutane)][BAr^F$_4$]** to **[1-methylbutenes][BAr^F$_4$]** in the solid-state. Where k = growth rate constant, 9.5×10^{-4} ($\pm$ 6×10^{-5}) s^{-1}, and n = Avrami exponent (0.50 ± 0.02).

S.2.4. [Rh($Cy_2PCH_2CH_2PCy_2$)(C_6H_{14})][BAr^F_4], [1-hexane][BAr^F_4]

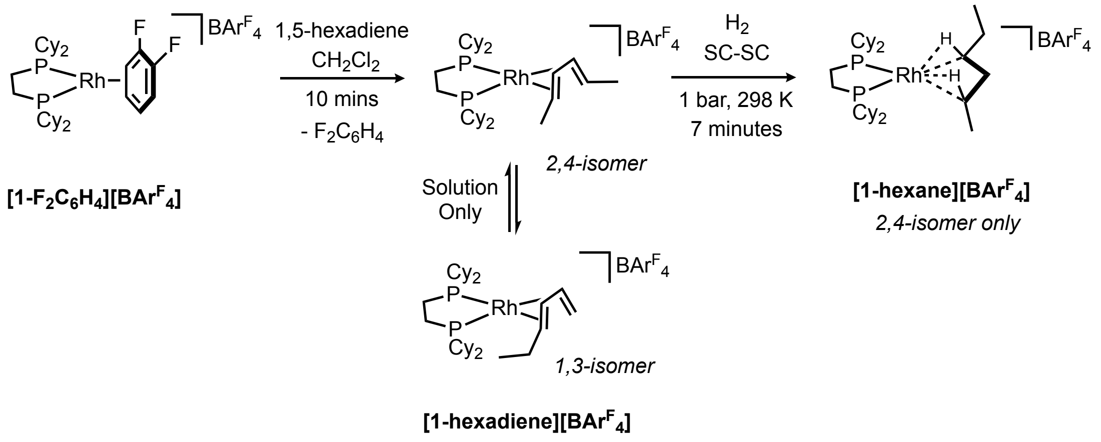

S.2.4.1. Synthesis of a precursor hexane complex, [1-hexadiene][BAr^F_4]

A pale-yellow solution of **[1-$F_2C_6H_4$][BAr^F_4]** (600 mgs, 0.40 mmol) in CH_2Cl_2 (20 mL) was treated with neat 1,5-hexadiene (0.1 mL, 0.48 mmol) at ambient temperature. The resultant deep red solution was stirred at ambient temperature for 30 minutes. Pentane (100 mL) was then added with vigorous stirring which resulted in the formation of a red precipitate. The solid was isolated by filtration and washed with pentane (2 × 20 mL). The solid was then dissolved in 1,2-dichloroethane (~12 mL) and filtered into a J. Young crystallisation flask and layered with pentane. Ruby red crystals were obtained after storage for 1 week at room temperature of [Rh($Cy_2PCH_2CH_2PCy_2$)(C_6H_{10})][BAr^F_4], **[1-hexadiene][BAr^F_4]** (Yield: 404 mgs, 69 %).

Note: The 2,4-isomer was found to be the only isomer present in the solid-state. Taking single-crystals of the 2,4-isomer and re-dissolving in CH_2Cl_2 initially showed only the 2,4-isomer, where the mixture of isomers was observed to grow in within 2 hours.

S.2.4.2. Charaterisation data and spectra for [1-hexadiene][BAr^F_4]

^{1}H NMR (CD_2Cl_2, 298 K, 400 MHz): δ 7.82 (s, 8H, *ortho*-ArH), 7.67 (s, 4H, *para*-ArH), 5.68 (d. of d., alkene-H, J = 14.6, 3.8 Hz), 5.49 (d. of m., alkene-H, J = 12.5 Hz), 5.34 (m., alkene-H, J = 1.1 Hz), 4.25 (m., alkene-H), 4.12 (m., alkene-H), 2.88 (d., alkene-H, J = 14.4 Hz), 2.16 -1.70 (br m, overlapping aliphatic CH), 1.52-0.98 (br m, aliphatic CH).

^{31}P{^{1}H} NMR (CD_2Cl_2, 298 K, 162 MHz): 2,4-isomer: δ 80.6 (d, J_{RhP} = 179 Hz). The 1,3-isomer: δ 84.5 (d.d., J_{RhP} = 174 Hz, J_{PP} = 22 Hz), δ 77.3 (d.d., J_{RhP} = 171 Hz, J_{PP} = 22 Hz).

^{19}F{^{1}H} NMR (CD_2Cl_2, 298 K, 377 MHz): δ -62.9 (s).

^{13}C{^{1}H} NMR (CD_2Cl_2, 298 K, 126 MHz): δ 162.1 (q, J_{CB} = 50 Hz, *ipso*-ArC), 135.1 (s, *ortho*-ArC), 129.2 (q, J_{CF} = 32 Hz, meta-ArC), 125.0 (q, J_{CF} = 272 Hz, -CF_3), 117.7 (s, *para*-ArC), 108.2, 109.2, 104.6, 101.0, 98.6, 97.5, 88.8, 61.8 (m, multiple alkene resonances from 2,4-isomer and 1,3-isomers), 38.8, 38.5, 37.8, 37.0, 36.2, 35.5, 32.0 - 19.9 (m, overlapping cyclohexyl-CH_2 from isomers A and B).

ESI-MS (calc. for $[Rh(Cy_2PCH_2CH_2PCy_2)(C_6H_{10})]^+$): *m/z* 607.3086 (607.3063).
Elemental Analysis (calc. for $C_{64}H_{70}BF_{24}P_2Rh$): C 52.17 (52.26), H 4.83 (4.80).

^{31}P{^{1}H} SSNMR (162 MHz, 298 K, 10 kHz spin rate): δ 82.5 (d, J_{RhP} = 189 Hz), 74.5. (d, J_{RhP} = 182 Hz).

^{13}C{^{1}H} SSNMR (101 MHz, 298 K, 10 kHz spin rate): δ 164.7 (ArC), 161.3 (ArC), 134.8 (ArC), 129.0 (ArC), 124.4 (ArC), 117.7 (ArC), 115.1 (ArC), 99.3 (C=C), 98.6 (C=C), 88.6 C=C), 85.3 (C=C), 39.6, 38.1, 34.9, 32.5, 30.6, 29.2, 27.0, 26.0, 24.6, 22.6, 19.9, 17.0 (multiple aliphatic resonances).

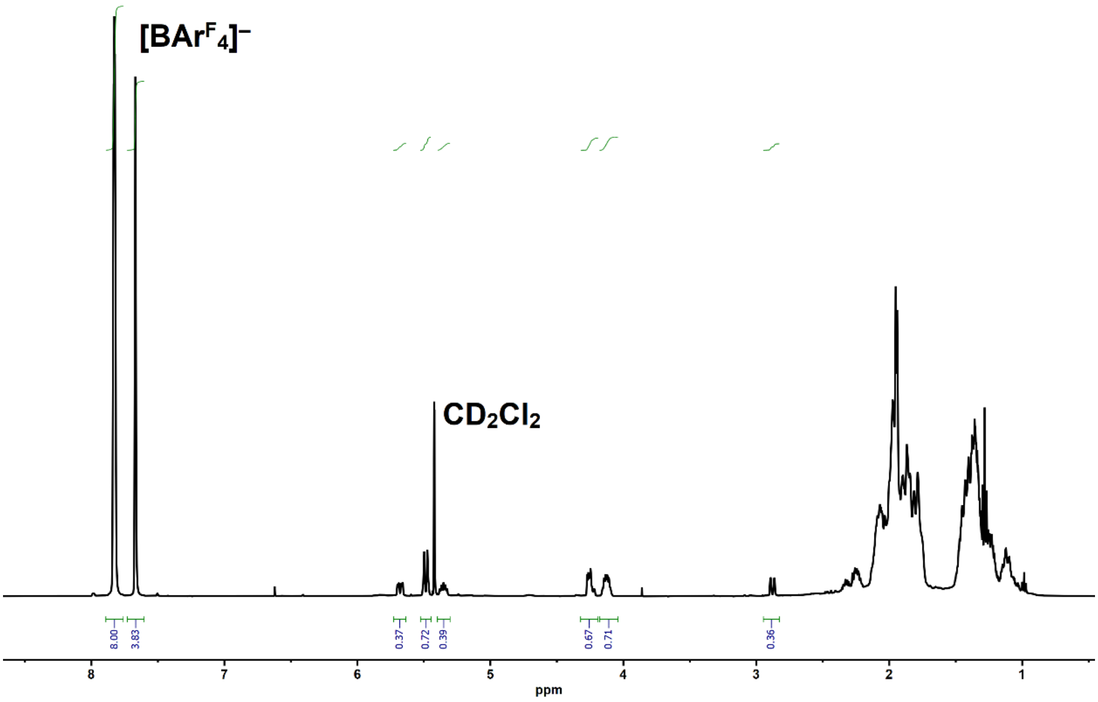

Figure S23: The solution ^{1}H NMR spectrum (CD_2Cl_2, 298 K, 400 MHz) of **[1-hexadiene][BAr$^F{}_4$]**.

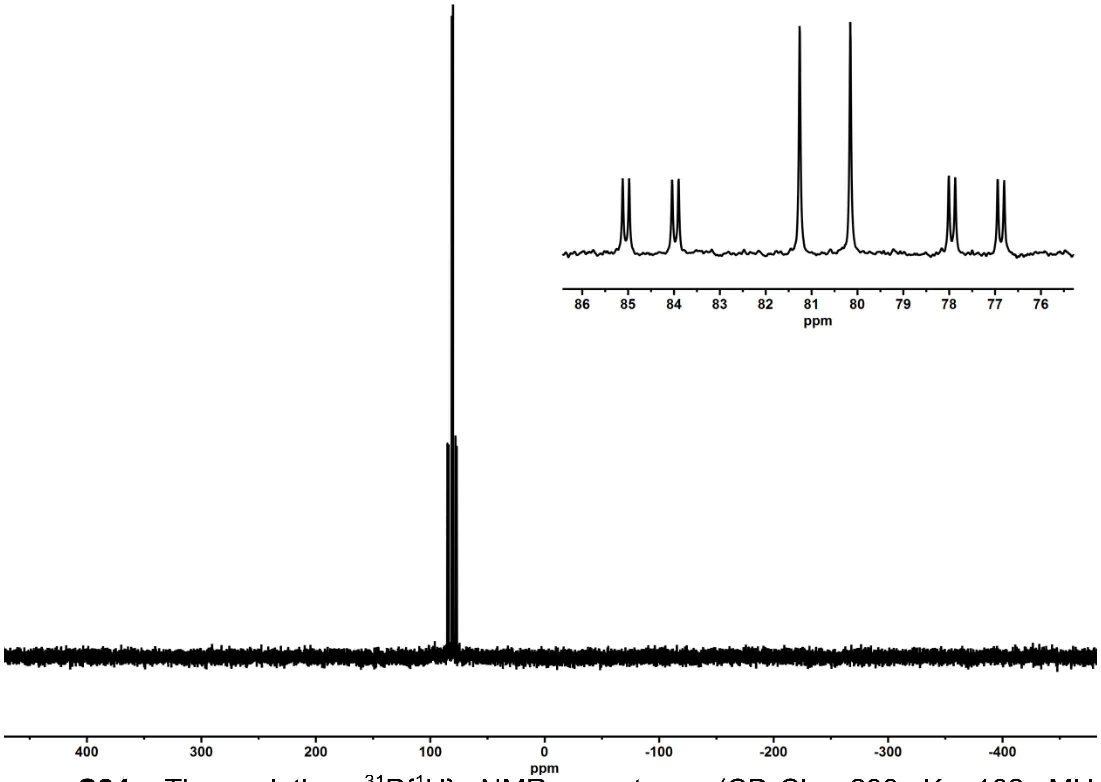

Figure S24: The solution $^{31}P\{^1H\}$ NMR spectrum (CD_2Cl_2, 298 K, 162 MHz) of **[1-hexadiene][BAr$^F{}_4$]**. The inset is an enlargement of the resonances between δ 86 and 76.

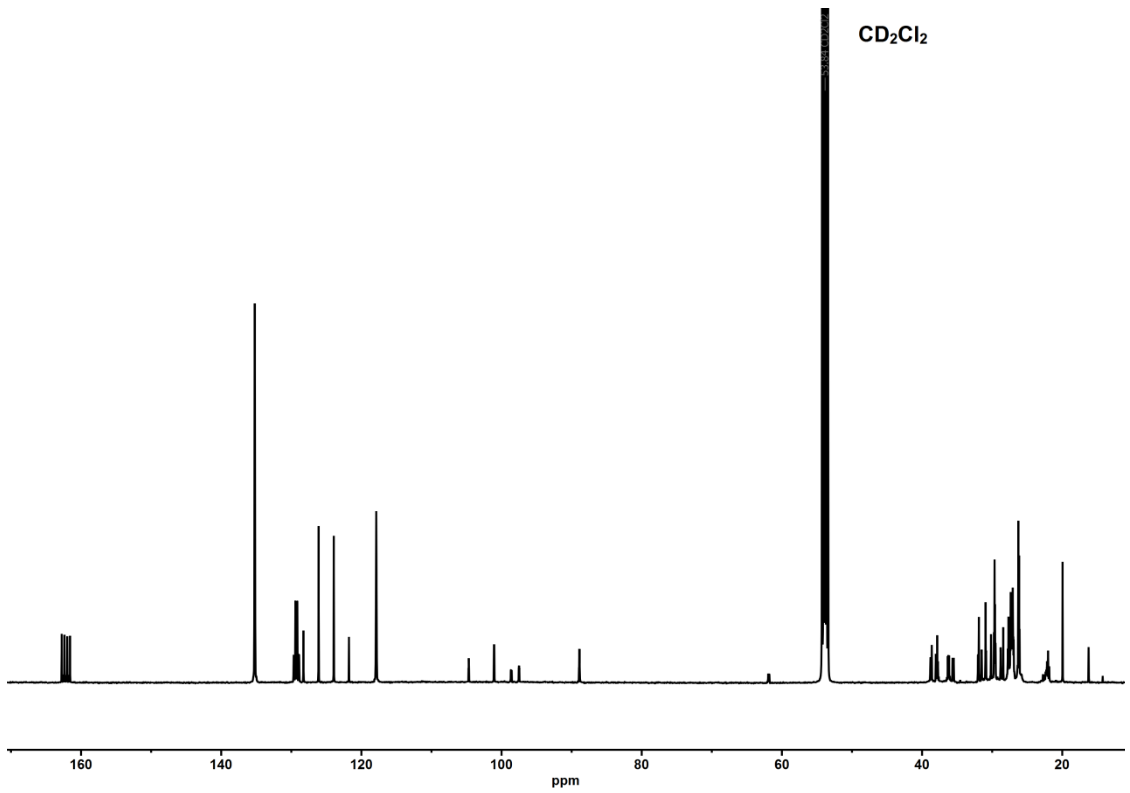

Figure S25: The solution $^{13}C\{^{1}H\}$ NMR spectrum (CD_2Cl_2, 298 K, 126 MHz) of **[1-hexadiene][BAr^F$_4$]**.

.

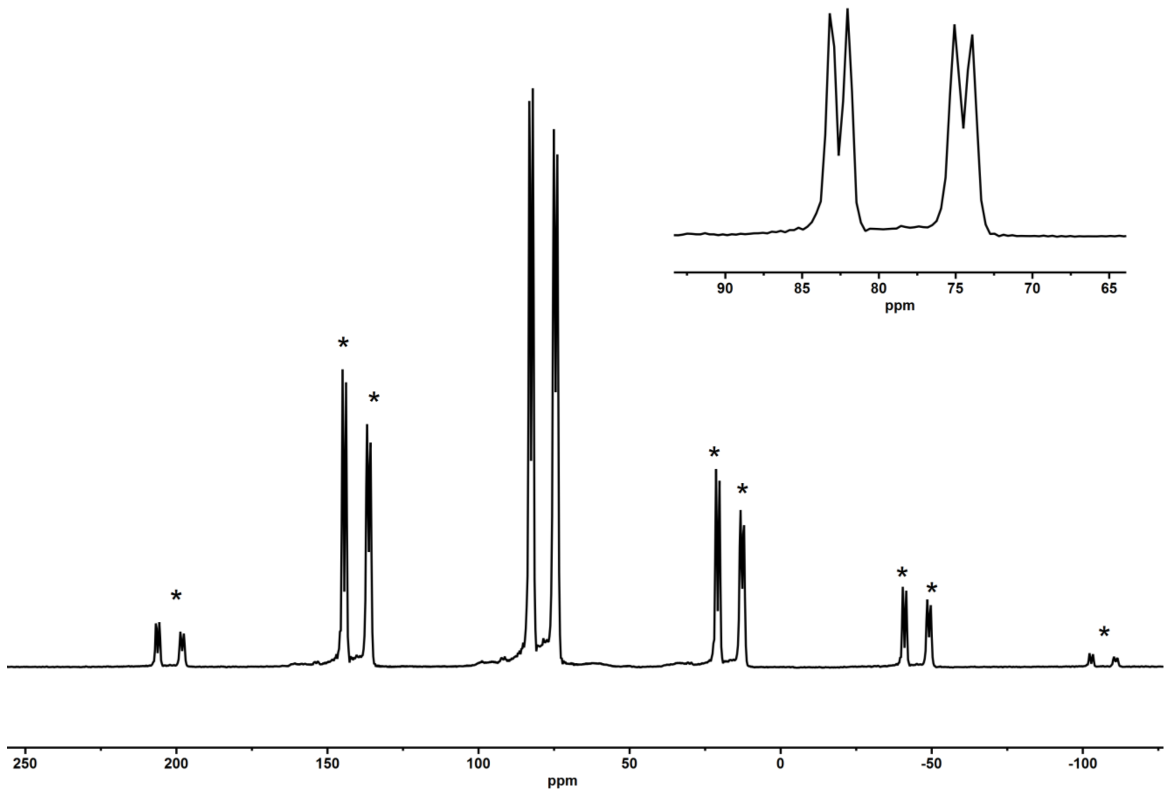

Figure S26: The $^{31}P\{^{1}H\}$ SSNMR spectrum (162 MHz, 294 K, 10 kHz spin rate) of **[1-hexadiene][BAr$^F{}_4$]**.The resonances marked * are due to spinning sidebands

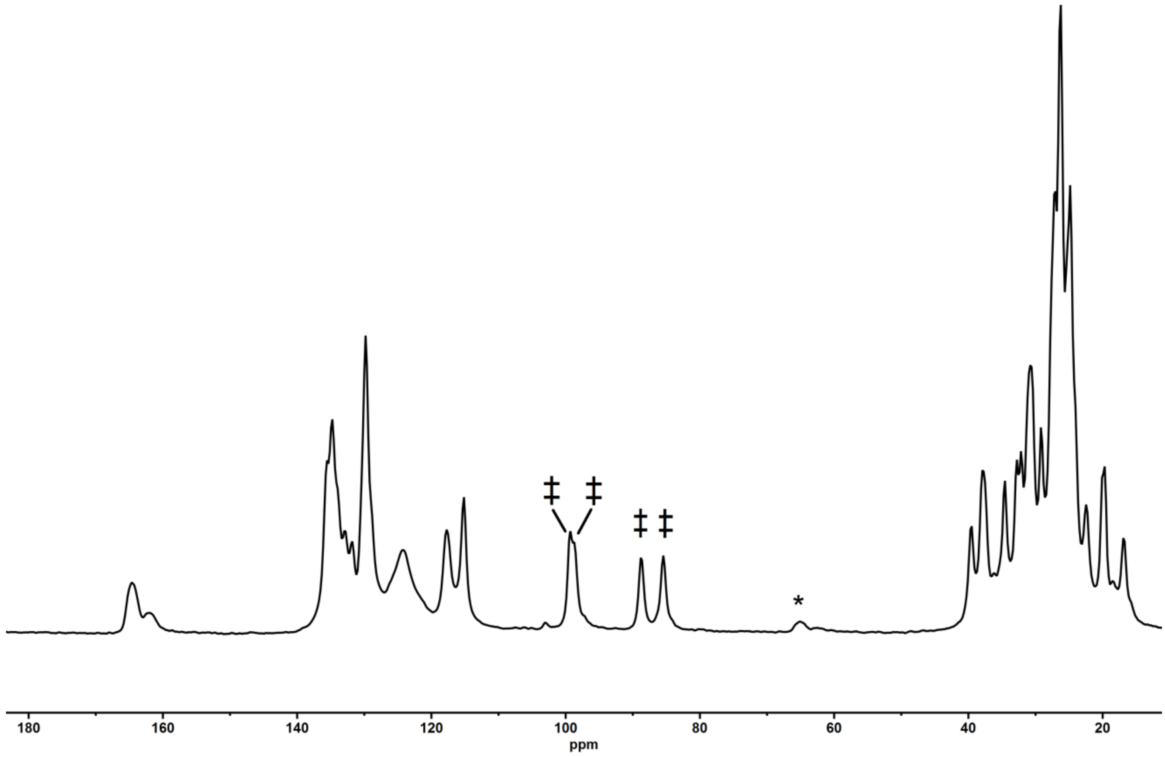

Figure S27: The $^{13}C\{^{1}H\}$ SSNMR spectrum (101 MHz, 294 K, 10 kHz spin rate) of **[1-hexadiene][BAr$^F{}_4$]**. The resonances marked * are due to spinning sidebands. The alkene resonances have been assigned ‡.

S.2.4.3. Synthesis of a hexane complex, [1-hexane][BAr^F_4]

For Single-Crystal X-Ray Diffraction: Ruby red crystals of **[1-hexadiene][BAr^F_4]** (1.5 mgs) were placed in a J. Young NMR tube and treated with H_2 (1 bar, 298 K, 7 minutes). The colour of the crystals slowly turned a dark red over this time. The crystalline material was then removed from the tube and coated with Fomblin® Y oil under an argon flush. A suitable crystal was then selected and transferred to the cryostream of a diffractometer (150 K). An X-ray diffraction study was undertaken and the structure of $[Rh(Cy_2PCH_2CH_2PCy_2)(C_6H_{14})][BAr^F_4]$ **[1-hexane][BAr^F_4]** was refined. For further details of structural refinement please refer to Section S.3.

For Solid-State NMR Spectroscopy: A powdered microcrystalline sample of **[1-hexadiene][BAr^F_4]** (45 mgs) was packed in a 4.0 mm SSNMR rotor, inside an argon filled glove box. The rotor was then placed in a custom-built glass J. Young flask[S5] and the sample was then exposed to H_2 (1 bar, 298 K). After 7 minutes, the rotor cap was fitted under a flush of H_2. The sample was rapidly transferred to the bore of a SSNMR spectrometer, cooled to 158 K (~10 minutes) and analysed by $^{31}P\{^1H\}$ and $^{13}C\{^1H\}$ solid-state NMR spectroscopy.

Note: These conditions were optimised to form **[1-hexane][BAr^F_4]**. At 158K, no onward reactivity with H_2 is observed. Further exposure to H_2 (30 minutes) at 298 K results in complete decomposition to **[1-BAr^F_4]**.[S2]

S.2.4.4. Charaterisation data and spectra for [1-hexane][BAr^F_4]

$^{31}P\{^1H\}$ SSNMR (162 MHz, 158 K, 10 kHz spin rate): δ 106.0 (d, J_{RhP} = 226 Hz), 102.5. (d, J_{RhP} = 216 Hz).

$^{13}C\{^1H\}$ SSNMR (101 MHz, 158 K, 10 kHz spin rate): δ 162.7 (ArC), 134.0 (ArC), 129.1 (ArC), 124.0 (ArC), 115.7 (ArC), 37.2, 34.9, 34.6, 32.5, 31.5, 30.7 , 28.1, 24.8, 22.6, 20.1, 18.1, 13.9, 13.1 (multiple aliphatic resonances).

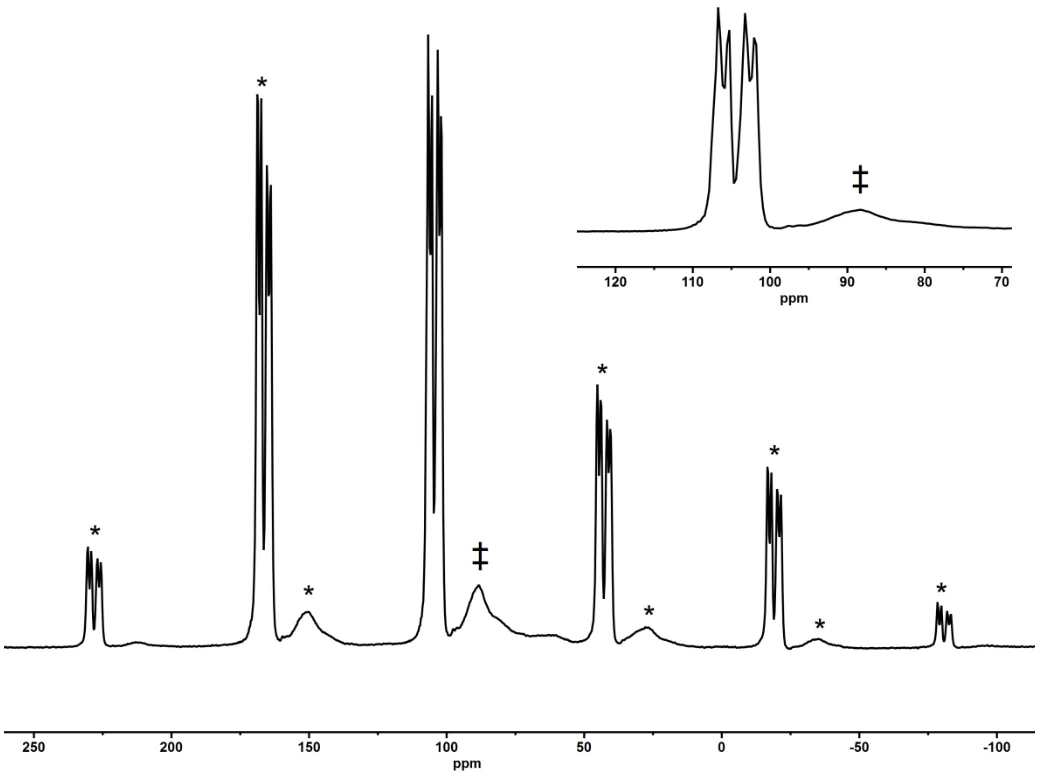

Figure S28: The $^{31}P\{^{1}H\}$ SSNMR spectrum (162 MHz, 158 K, 10 kHz spin rate) **[1-hexane][BAr$^F_4$]** prepared *in situ* after 15 minutes under H_2 at 298K. The resonances marked are assigned to an **[1-BAr$^F_4$]**[S2] decomposition product under H_2 at 298K. The resonances marked * are due to spinning sidebands. The inset is an enlargement of the central resonances.

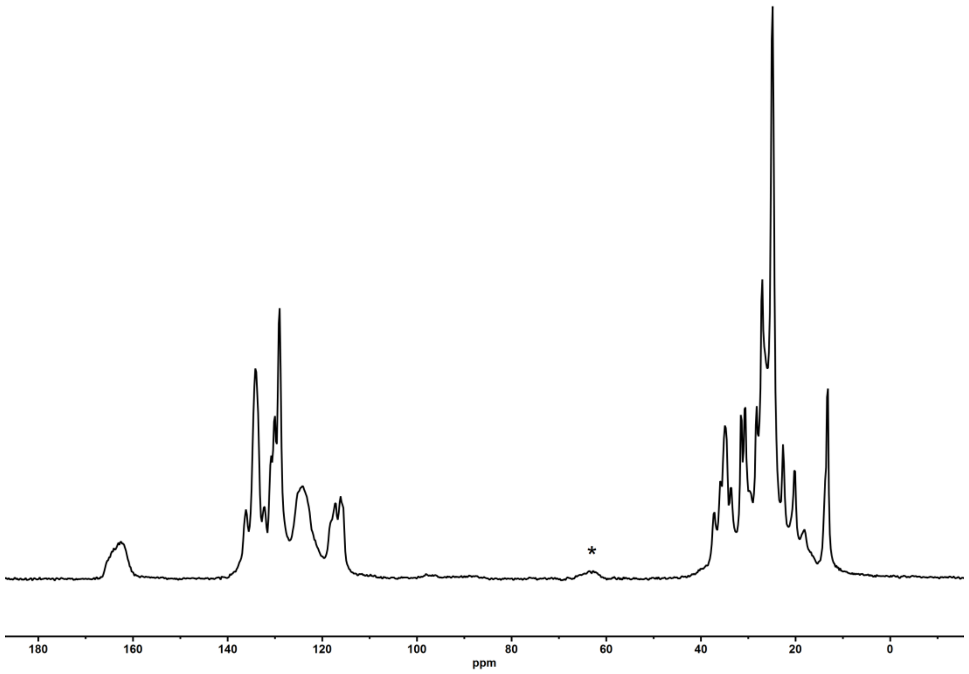

Figure S29: The $^{13}C\{^{1}H\}$ SSNMR spectrum (100 MHz, 158 K, under H_2, 10 kHz spin rate) of **[1-hexane][BAr$^F_4$]** prepared *in situ*. The resonance marked * is due to a spinning sideband.

S.2.4.5. Stability of [1-hexane][BAr^F$_4$] at 294 K

On the same sample as presented in Figures S31 to S32, the solid-state NMR spectrometer was warmed to 294 K and $^{31}P\{^1H\}$ SSNMR spectra collected until complete decomposition of **[1-hexane][BAr^F$_4$]** was observed. During this time, the solid-state rotor was under a flushing N_2 atmosphere.

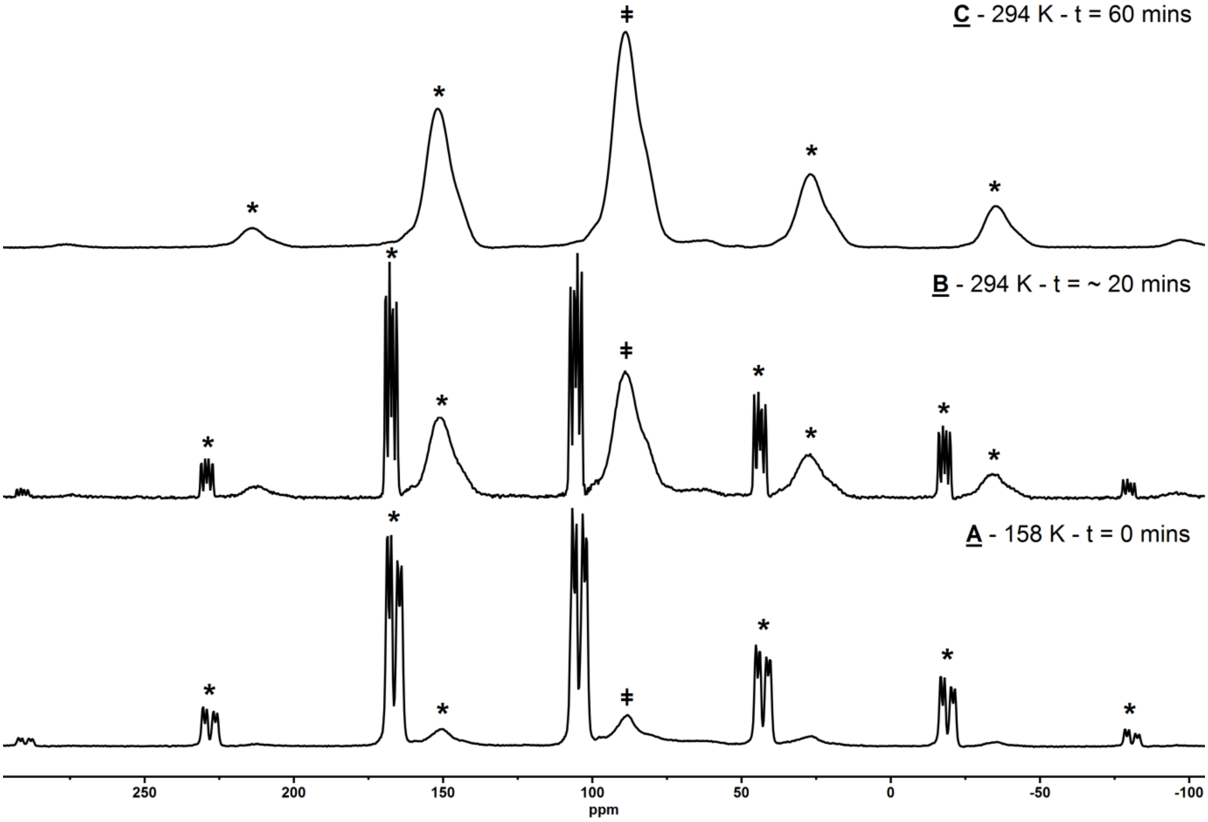

Figure S30: The $^{31}P\{^1H\}$ SSNMR (162 MHz, 10 kHz spin rate) spectra of **A**: **[1-hexane][BAr^F$_4$]** as prepared at 158 K. **B**: Spectrum collected upon warming to 294 K. Total time at 294 K including instrument warming, stabilisation and experimental set-up / collection is **20 minutes**. **C**: Total time at 294 K including instrument warming, stabilisation and experimental set-up / collection is **60 minutes**. Peaks marked * are spinning sidebands and ‡ are assigned to **[1-BAr^F$_4$]**[S2] in all parts.

S.2.5. [Rh($Cy_2PCH_2CH_2PCy_2$)(C_6H_{14})][$BAr^F{}_4$], [1-(3-methypentane)][$BAr^F{}_4$]

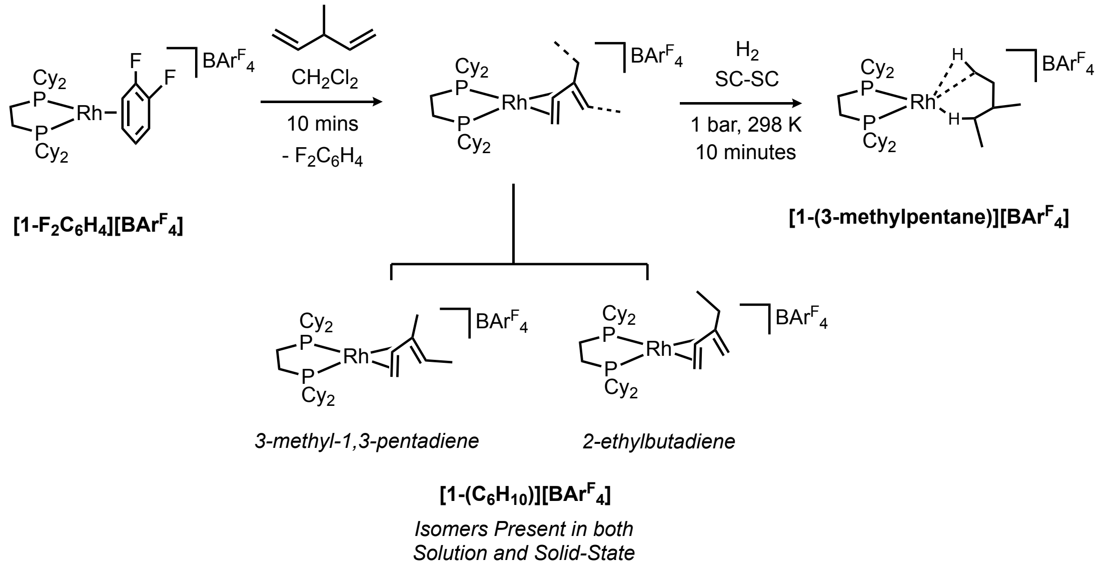

S.2.5.1. Synthesis of a precursor 3-methylpentane complex, [1-(C_6H_{10})][$BAr^F{}_4$] (C_6H_{10} = 3-methyl-1,3-pentadiene or 2-ethylbutadiene)

A pale-yellow solution of **[1-$F_2C_6H_4$][$BAr^F{}_4$]** (600 mgs, 0.40 mmol) in CH_2Cl_2 (20 mL) was treated with neat 3-methyl-1,4-pentadiene (0.1 mL, 0.78 mmol) at ambient temperature. The resultant deep red solution was stirred at ambient temperature for 30 minutes. Pentane (100 mL) was then added with vigorous stirring which resulted in the formation of a red precipitate. The solid was isolated by filtration and washed with pentane (2 × 20 mL). The solid was then dissolved in CH_2Cl_2 (~8 mL) and filtered into a J. Young crystallisation flask and layered with pentane. Deep red crystals were obtained after storage for 1 week at room temperature of [Rh($Cy_2PCH_2CH_2PCy_2$)(C_6H_{10})][$BAr^F{}_4$], **[1-(C_6H_{10})][$BAr^F{}_4$]** (C_6H_{10} = 3-methyl-1,3-pentadiene or 2-ethylbutadiene) (Yield: 515 mgs, 88 %).

S.2.5.2. Charaterisation data and spectra for [1-(C_6H_{10})][BAr$^F{}_4$]

^{1}H NMR (CD_2Cl_2, 298 K, 400 MHz): δ 7.72 (s, 8H, *ortho*-ArH), 7.56 (s, 4H, *para*-ArH), 5.16 (d. of d. of d., alkene-H, J = 13.9, 8.6, 2.2 Hz), 5.01 (d. of d. of d., alkene-H, J = 13.7, 8.1, 2.1 Hz), 4.37-4.33 (broad m., alkene-H), 4.04 (d. of d. of d., alkene-H, J = 8.2, 4.3, 1.4 Hz), 3.90 (apparent quartet, alkene-H, J = 6.3 Hz), 2.31 -1.51 (br m, overlapping aliphatic CH), 1.44-0.82 (br m, aliphatic CH).

^{31}P{^{1}H} NMR (CD_2Cl_2, 298 K, 162 MHz): 3-methyl-1,3-pentadiene Isomer: δ 85.6 (d.d., J_{RhP} = 171 Hz, J_{PP} = 24 Hz), 79.2 (d.d., J_{RhP} = 169 Hz, J_{PP} = 24 Hz). 2-ethylbutadiene Isomer: δ 84.0 (d.d., J_{RhP} = 172 Hz, J_{PP} = 23 Hz), 75.3 (d.d., J_{RhP} = 163 Hz, J_{PP} = 23 Hz).

^{19}F{^{1}H} NMR (CD_2Cl_2, 298 K, 377 MHz): δ -62.9 (s).

^{13}C{^{1}H} NMR (CD_2Cl_2, 298 K, 126 MHz): δ 162.7 (q, J_{CB} = 50 Hz, *ipso*-ArC), 134.7 (s, *ortho*-ArC), 128.8 (q, J_{CF} = 32 Hz, meta-ArC), 125.1 (apparent triplet, C=C, J = 4.3 Hz), 124.6 (q, J_{CF} = 272 Hz, -CF_3), 119.3 (apparent triplet, C=C, J = 4.2 Hz), 117.4 (s, *para*-ArC), 99.6 (apparent triplet, C=C, J = 5.5 Hz), 98.4 (apparent triplet, C=C, J = 5.7 Hz), 86.0 (d. of d., C=C, J = 12.8, 3.3 Hz), 63.4 (d. of d. of d., C=C, J = 10.5, 7.8, 1.5 Hz), 62.8 (d. of d. of d., C=C, J = 14.2, 5.4, 1.6 Hz), 59.2 (d. of d. of d., C=C, J = 11.8, 8.2, 2.1 Hz), 38.3 – 21.1 (m, overlapping cyclohexyl-CH_2 from both isomers), 19.4, 15.9, 14.5, 13.8 (s, -ethyl/-methyl groups from co-ordinated isomers).

^{31}P{^{1}H} SSNMR (162 MHz, 294 K, 10 kHz spin rate): δ 84.8 (from two, coincident ^{31}P environments), 79.6, 74.9.

^{13}C{^{1}H} SSNMR (101 MHz, 294 K, 10 kHz spin rate): δ 163.3 (*ipso*-ArC), 134.7 (*ortho*-ArC), 129.5 (*meta*-ArC), 124.4 (br, CF_3), 117.2 (*para*-ArC), 101.8 (C=C), 99.8 (C=C), 84.3 (C=C), 63.5 (C=C), 38.7 (CH), 36.0 (CH), 30.8 (CH_2), 26.2 (CH_2), 25.3 (CH_2), 21.2 (CH_2), 17.5 (CH_2), 14.5 (CH_2).

ESI-MS (calc. for [Rh($Cy_2PCH_2CH_2PCy_2$)(C_6H_{10})]$^+$): *m/z* 607.3097 (607.3063).
Elemental Analysis (calc. for $C_{64}H_{70}BF_{24}P_2Rh$): C 52.38 (52.26), H 4.57 (4.80).

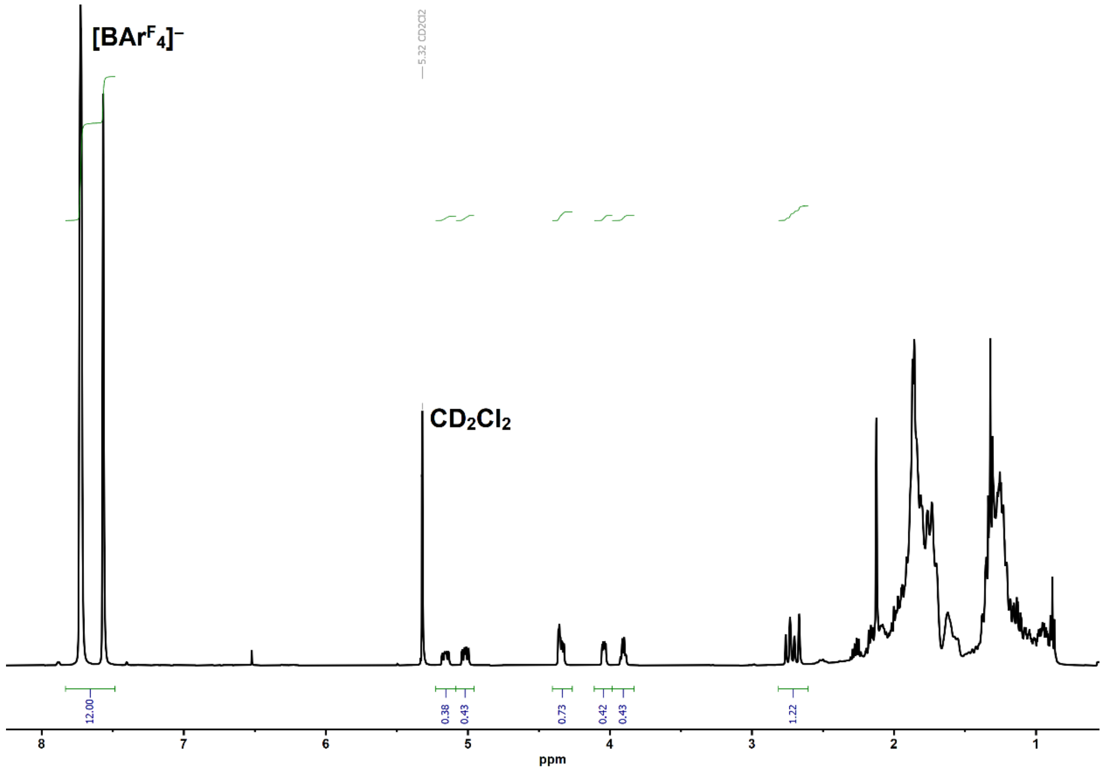

Figure S31: The solution ^{1}H NMR spectrum (CD_2Cl_2, 298 K, 400 MHz) of **[1-(C_6H_{10})][BAr^F_4]** (C_6H_{10} = 3-methyl-1,3-pentadiene or 2-ethylbutadiene).

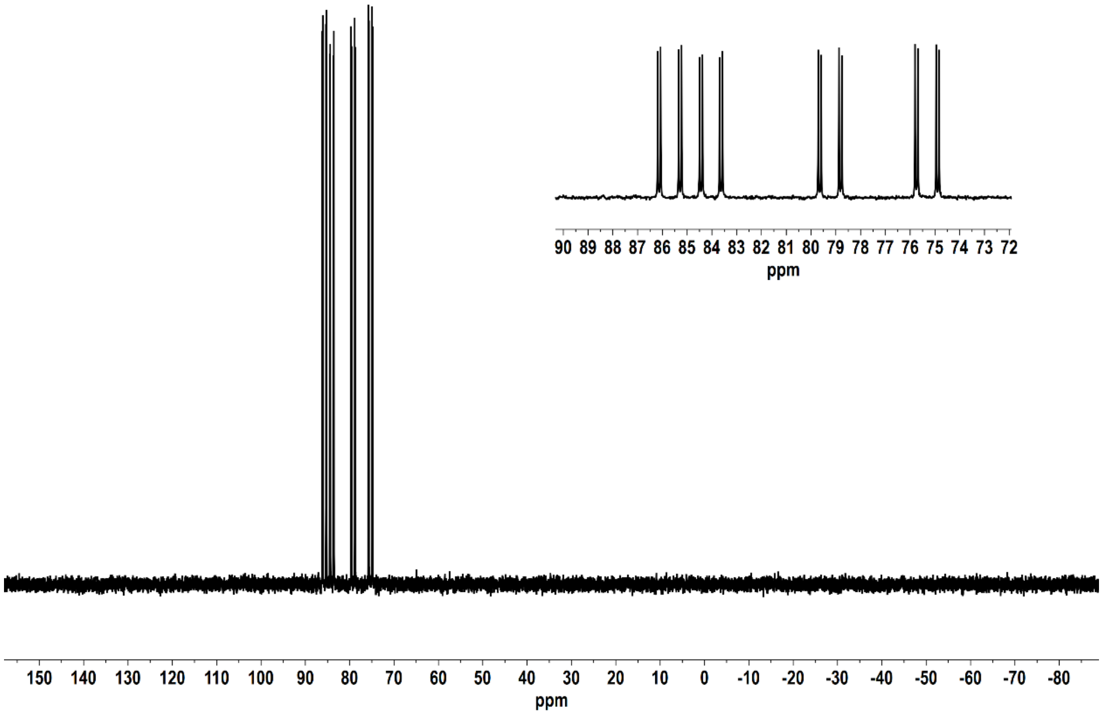

Figure S32: The solution $^{31}P\{^1H\}$ NMR spectrum (CD_2Cl_2, 298 K, 162 MHz) of **[1-(C_6H_{10})][BAr^F_4]** (C_6H_{10} = 3-methyl-1,3-pentadiene or 2-ethylbutadiene). The inset is an enlargement of the resonances between δ 90 and 72.

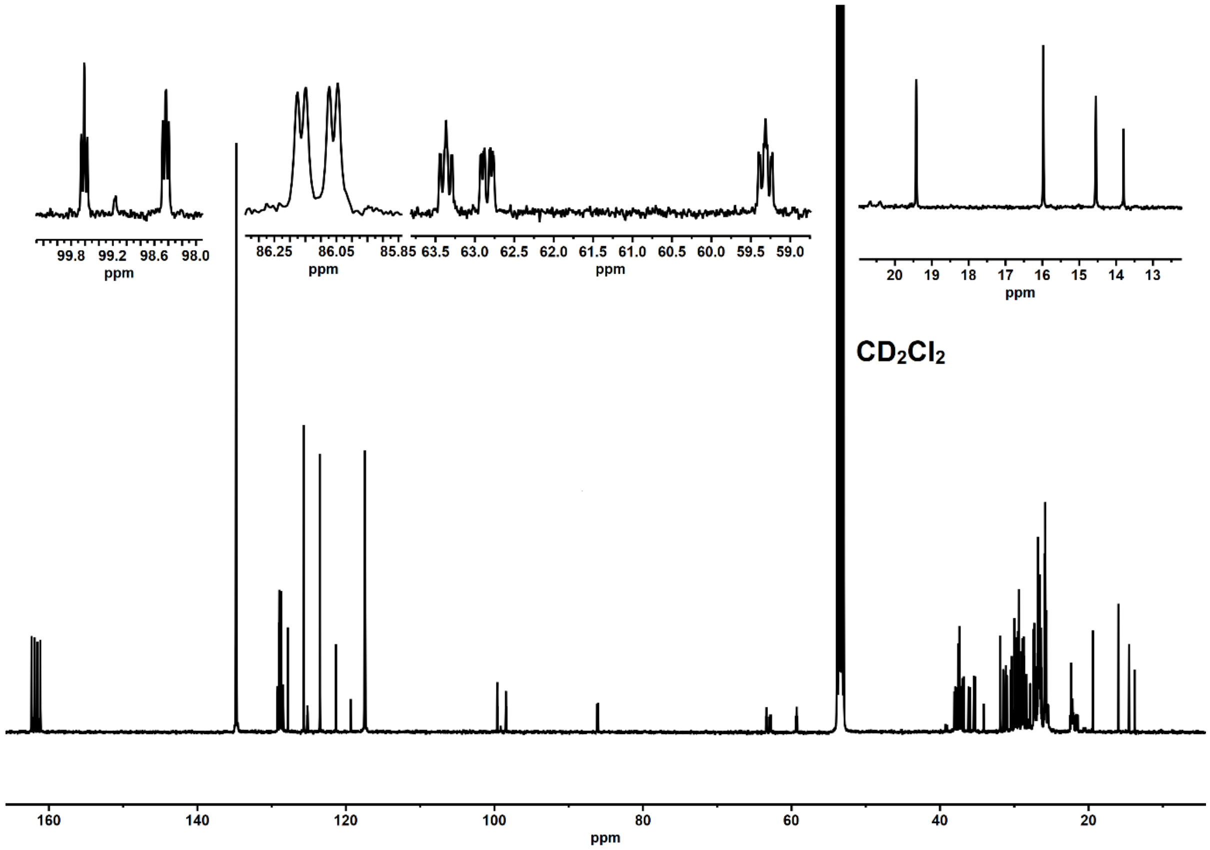

Figure S33: The solution $^{13}C\{^{1}H\}$ NMR spectrum (CD_2Cl_2, 298 K, 126 MHz) of **[1-(C_6H_{10})][BAr$^F_4$]** (C_6H_{10} = 3-methyl-1,3-pentadiene or 2-ethylbutadiene). The insets are enlargements of the resonances of alkene or ethyl/-methyl groups from co-ordinated the C_6-isomers.

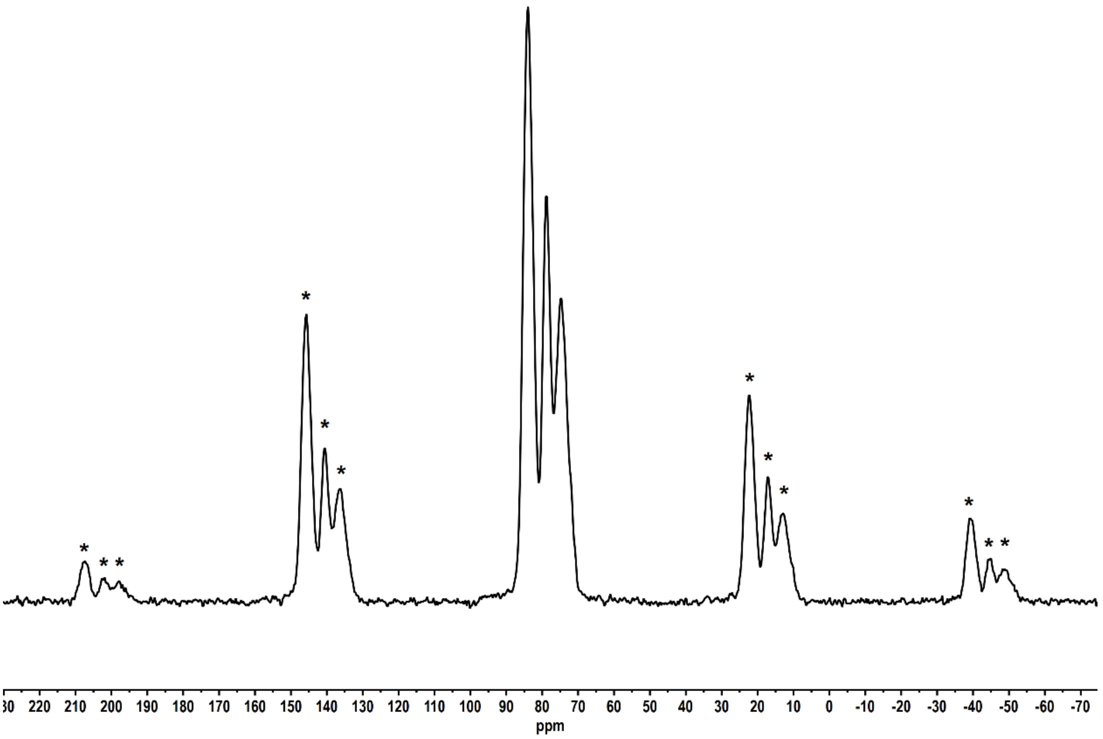

Figure S34: The ^{31}P{^{1}H} SSNMR spectrum (162 MHz, 294 K, 10 kHz spin rate) of **[1-(C_6H_{10})][BAr$^F{}_4$]** (C_6H_{10} = 3-methyl-1,3-pentadiene or 2-ethylbutadiene). The resonances marked * are due to spinning sidebands.

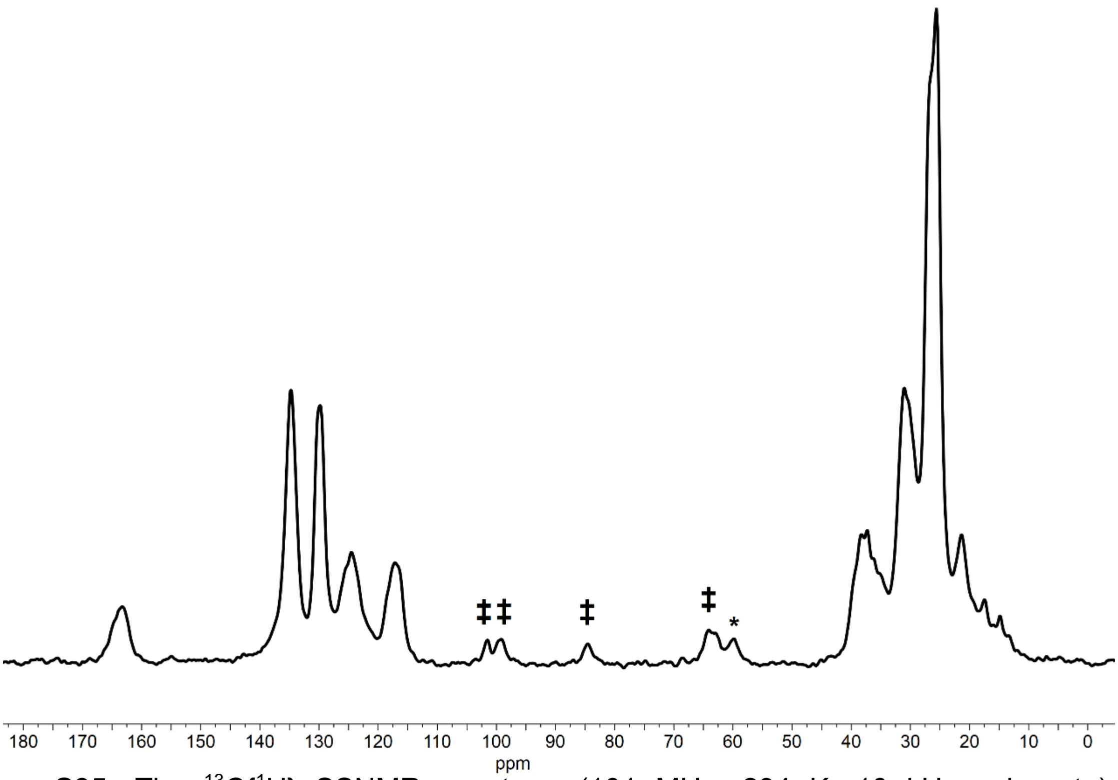

Figure S35: The ^{13}C{^{1}H} SSNMR spectrum (101 MHz, 294 K, 10 kHz spin rate) of **[1-(C_6H_{10})][BAr$^F{}_4$]**. The resonances marked * are due to spinning sidebands. The alkene resonances have been assigned ‡.

S.2.5.3. Liberation of bound C_6-isomers from **[1-(C_6H_{10})][BAr$^F{}_4$]** (C_6H_{10} = 3-methyl-1,3-pentadiene or 2-ethylbutadiene)

A J. Young NMR tube was charged with crystalline sample of **[1-(C_6H_{10})][BAr$^F{}_4$]** (30 mgs) dissolved in CD_2Cl_2 (0.5 mL, dark red solution) and MeCN-d_3 (0.05 mL) added to liberate the bound diene ligands. After 2 minutes, the solution had turned a pale-yellow colour which was confirmed to be [Rh($Cy_2PCH_2CH_2PCy_2$)($MeCN)_2$][BAr$^F{}_4$] by solution $^{31}P\{^1H\}$ NMR spectroscopy (δ 91, d, J_{RhP} = 175 Hz).[S5] The volatile components were then isolated by trap-to-trap distillation and analysed by solution ^1H NMR spectroscopy (Figure S39). A second batch was prepared in a similar fashion, however using neat MeCN, and analysed by solution $^{13}C\{^1H\}$ NMR spectroscopy (Figure S38).

$^{13}C\{^1H\}$ NMR (CD_2Cl_2, 298 K, 126 MHz): δ 148.2, 141.5, 139.0, 134.9, 133.4, 133.0, 127.3, 125.2, 114.4, 122.9, 122.8, 109.9, 23.8, 19.0, 13.0, 12.1, 11.2, 10.5.

^1H NMR (CD_2Cl_2, 298 K, 400 MHz): δ 6.84 (d. of d., J = 17.28, 10.55 Hz), 6.41 (d. of t., J = 18.18, 10.70 Hz), 5.60 (broad quartet, J = 6.46 Hz, H_4), 5.49 (broad quartet, J = 6.43 Hz, H_4), 5.26 (d., J = 18.6 Hz, H_4), 5.21 (d., J = 17.6 Hz), 5.08 (multiple overlapping signals), 5.04 (m.), 5.01 (m.), 4.92 (d., J = 11.85 Hz), 2.26 (quartet, J = 7.70 Hz, H_5), 1.82 (m.), 1.75 (m.), 1.12 (t., J = 7.70 Hz).

Literature Values: 2-ethylbutadiene[S9]

$^{13}C\{^1H\}$ NMR (Neat, 298 K, 10 MHz): δ 112.9 [1], 148.3 [2], 139.6 [3], 114.7 [4], 24.6 [5], 12.6 [6].

Literature Values: (*E*)-3-methyl-1,3-pentadiene[S10]

$^{13}C\{^1H\}$ NMR ($CDCl_3$, 298 K, 15.1 MHz): δ 133.5 [1], 133.2 [2], 125.0 [3], 113.0 [4], 19.71 [5], 12.9 [6].

Literature Values: (*Z*)-3-methyl-1,3-pentadiene[S11]

$^{13}C\{^1H\}$ NMR ($CDCl_3$, 298 K, 15.1 MHz): δ 141.6 [1], 135.0 [2], 127.0 [3], 110.0 [4], 13.7 [5], 11.3 [6].

Note: The solid-state structure of **[1-(C_6H_{10})][BAr$^F{}_4$]** (see Figure S60) was modelled to include both 2-ethylbutadiene and (*Z*)-3-methyl-1,3-pentadiene. (E)-3-methyl-1,3-pentadiene could not be modelled in the solid-state yet is observed in the ^1H NMR spectrum of the isolated dienes. We suggest a fast isomerisation event during the liberation of the bound dienes.

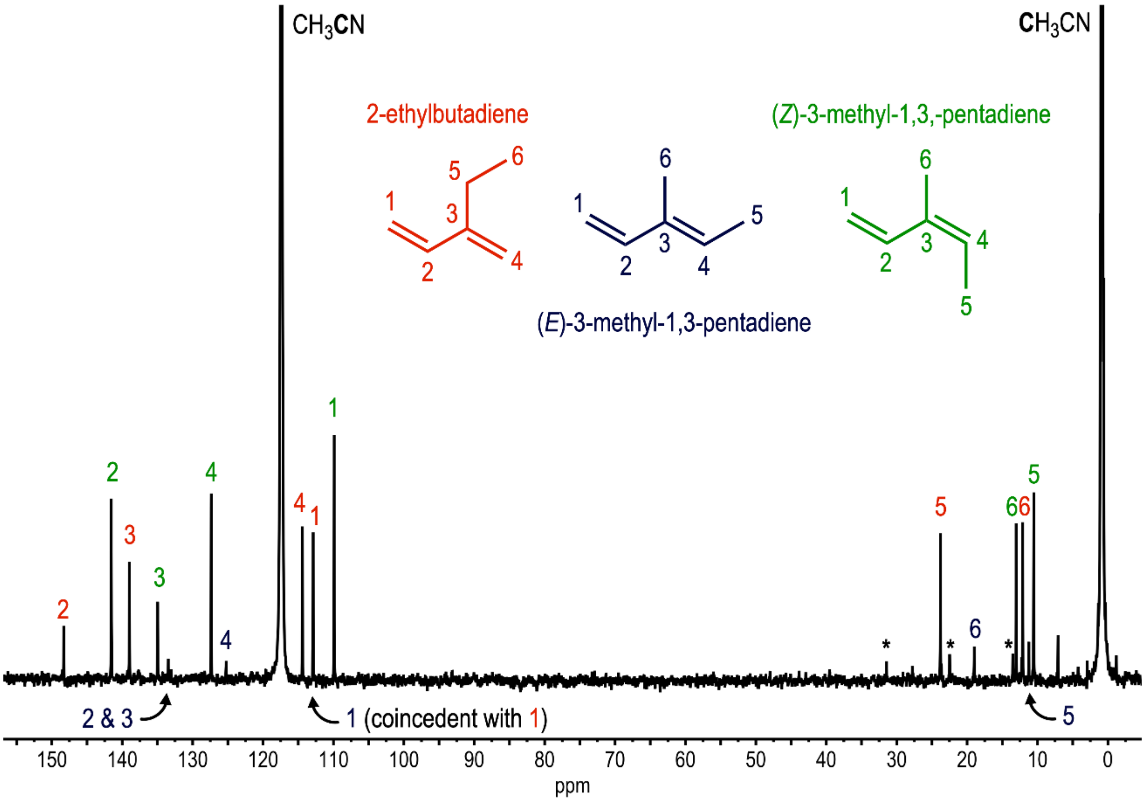

Figure S36: The solution ^{13}C{^{1}H} NMR (MeCN, 298 K, 126 MHz) spectrum of C_6H_{10} volatiles. * = *n*-hexane impurity.

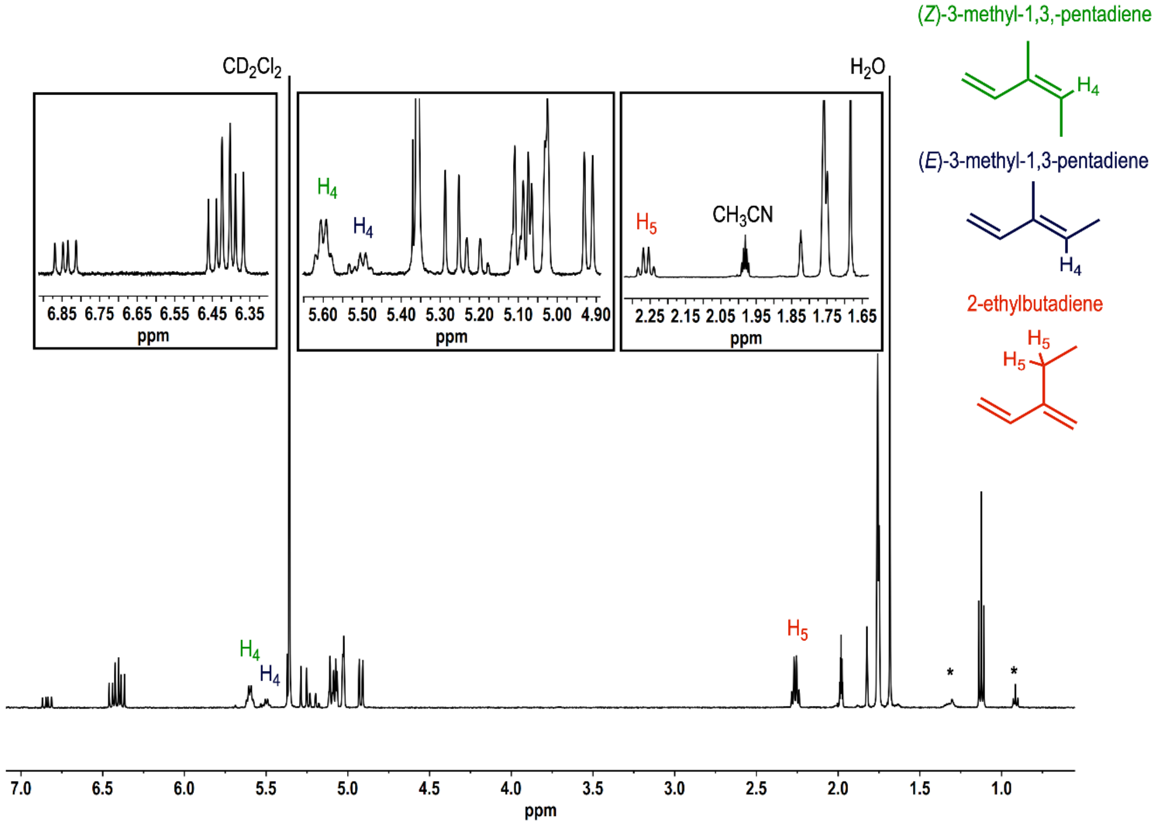

Figure S37: The solution ^{1}H NMR spectrum (CD_2Cl_2, 298 K, 500 MHz) of isolated C_6H_{10} volatiles. * = *n*-hexane impurity. The integration of the H_4: H_4: H_5 resonances is 0.70: 0.26 :1. This suggests an ~1: 1 ratio of coordinated (*Z/E*)-3-methyl-1,3-pentadiene and 2-ethylbutadiene.

S.2.5.4. Synthesis of a 3-methylpentane complex, [1-(3-methylpentane)][BAr$^F_4$]

For Single-Crystal X-Ray Diffraction: Ruby red crystals of **[1-(C_6H_{10})][BAr$^F_4$]** (C_6H_{10} = 3-methyl-1,3-pentadiene or 2-ethylbutadiene) (1.5 mgs) were placed in a J. Young NMR tube and treated with H_2 (1 bar, 298 K, 7 minutes). The colour of the crystals slowly turned a dark red over this time. The crystalline material was then removed from the tube and coated with Fomblin® Y oil under an argon flush. A suitable crystal was then selected and transferred to the cryostream of a diffractometer (150 K). An X-ray diffraction study was undertaken and the structure of [Rh($Cy_2PCH_2CH_2PCy_2$)(C_6H_{14})][BAr$^F_4$] **[1-(3-methylpentane)][BAr$^F_4$]** was refined. For further details of structural refinement please refer to Section S.3.

For Solid-State NMR Spectroscopy: A powdered microcrystalline sample of **[1-(C_6H_{10})][BAr$^F_4$]** (C_6H_{10} = 3-methyl-1,3-pentadiene or 2-ethylbutadiene) (45 mgs) was packed in a 4.0 mm SSNMR rotor, inside an argon filled glove box. The rotor was then placed in a custom-built glass J. Young flask[S5] and the sample was then exposed to H_2 (1 bar, 298 K). After 20 minutes, the rotor cap was fitted under a flush of H_2. The sample was rapidly transferred to the bore of a SSNMR spectrometer (~10 minutes) and analysed by $^{31}P\{^1H\}$ and $^{13}C\{^1H\}$ solid-state NMR spectroscopy.

S.2.5.5. Charaterisation data and spectra for [1-(3-methylpentane)][BAr$^F_4$]

$^{31}P\{^1H\}$ SSNMR (162 MHz, 285 K, 10 kHz spin rate): δ 107.9 (d, J_{RhP} = 188 Hz), 105.2 (d, J_{RhP} = 236 Hz) (~70 % yield).

$^{13}C\{^1H\}$ SSNMR (101 MHz, 285 K, 10 kHz spin rate): δ 164.6 (ArC), 135.9 (ArC), 131.5 (ArC), 130.6 (ArC), 125.5 (ArC), 119.3 (ArC), 118.6 (ArC), 117.1 (ArC), 39.1, 37.8, 36.9, 34.5, 33.7, 32.2, 31.4, 30.8, 30.2, 28.2, 26.8, 21.9, 19.2, 11.9, 10.5, 7.63 (multiple aliphatic resonances).

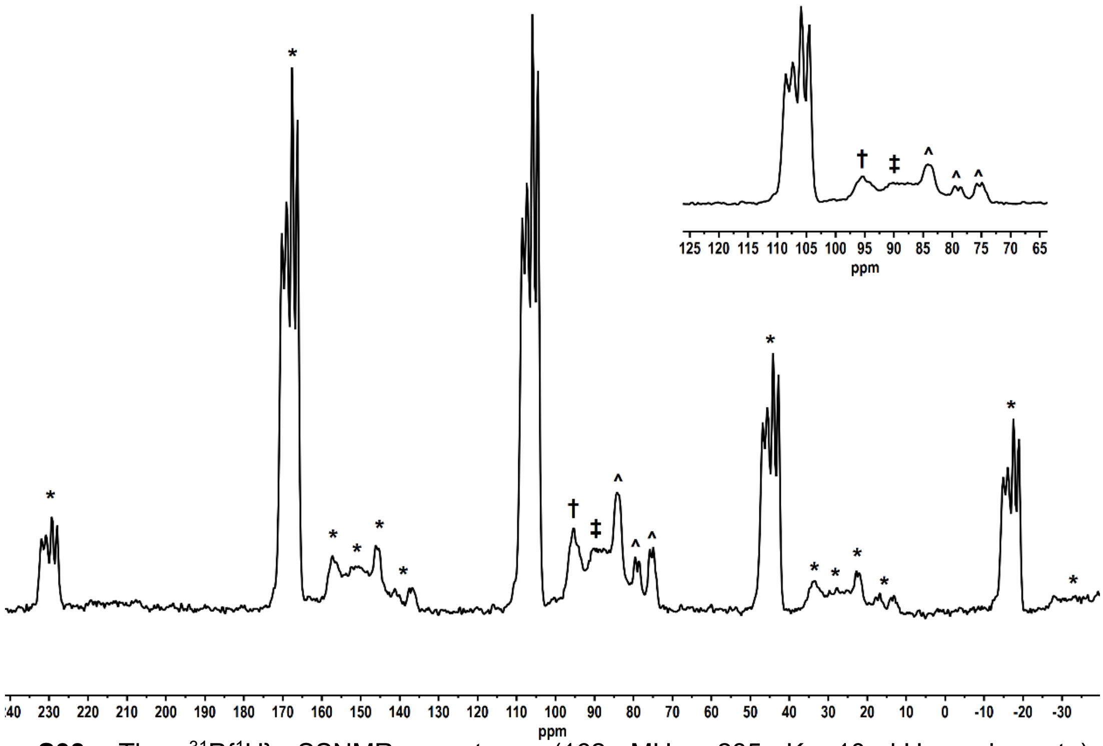

Figure S38: The $^{31}P\{^{1}H\}$ SSNMR spectrum (162 MHz, 285 K, 10 kHz spin rate) of **[1-(3-methylpentane)][BAr$^{F}_{4}$]**. Peaks marked * are assigned to spinning sidebands, ‡ to **[1-BAr$^{F}_{4}$]**[S2] and ^ to unreacted **[1-(C_6H_{10})][BAr$^{F}_{4}$]** (C_6H_{10} = 3-methyl-1,3-pentadiene or 2-ethylbutadiene) (see Fig. S36). † is assigned to the dehydrogenation product from **[1-(3-methylpentane)][BAr$^{F}_{4}$]**. The inset is an enlargement of the resonances between δ 125 and 65.

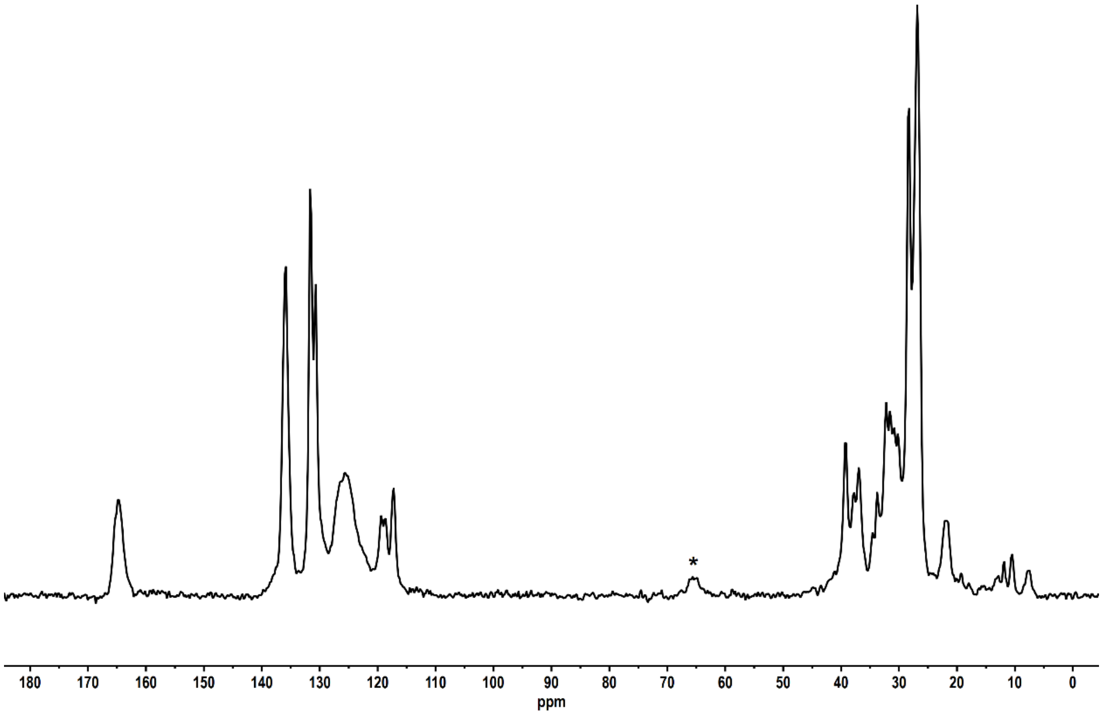

Figure S39: The $^{13}C\{^{1}H\}$ SSNMR spectrum (101 MHz, 285 K, 10 kHz spin rate) of **[1-(3-methylpentane)][BAr$^{F}_{4}$]**. The resonances marked * are due to spinning sidebands.

S.2.5.6. Stability of [1-(3-methylpentane)][BAr$^F_4$] at 294 K

On the same sample as presented in Figure S41 and S42, $^{31}P\{^1H\}$ SSNMR spectra were collected until the loss of any signals relating to **[1-(3-methylpentane)][BAr$^F_4$]** was observed.

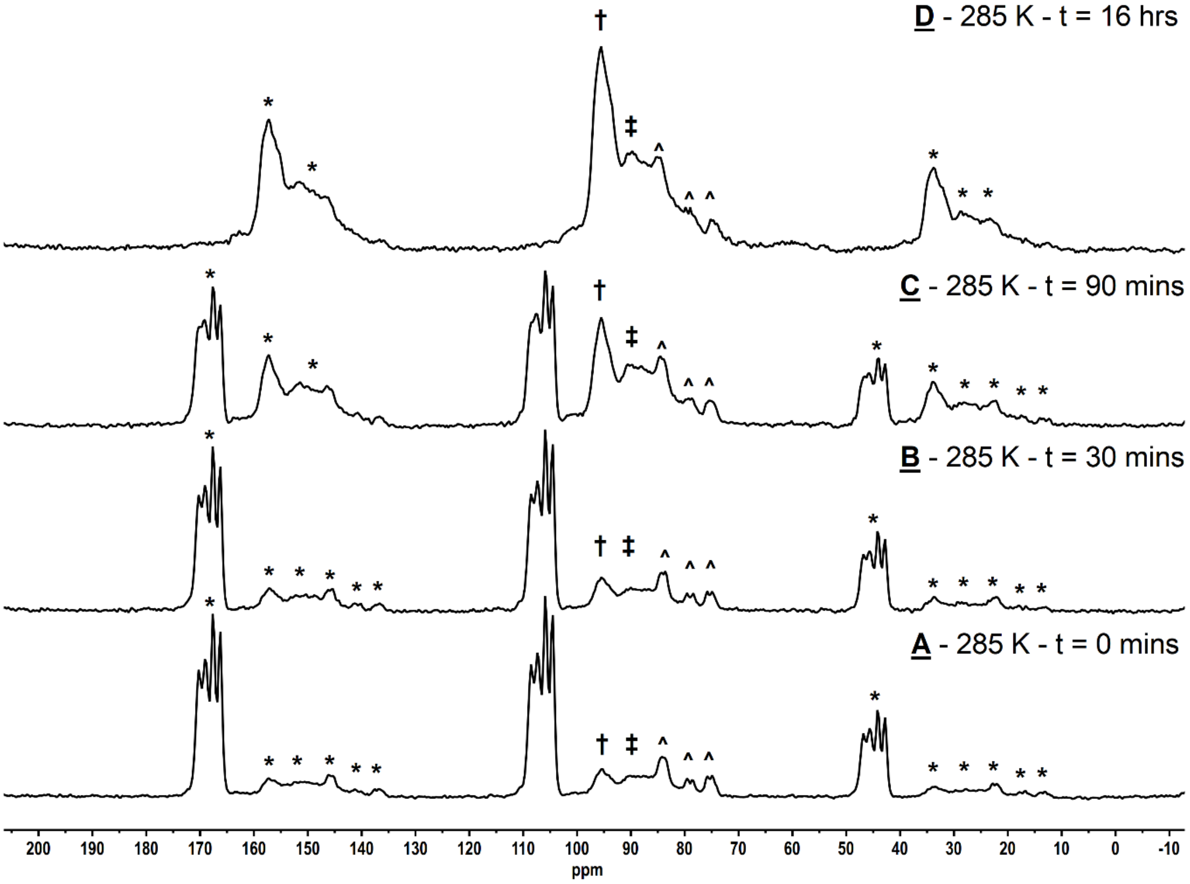

Figure S40: The $^{31}P\{^1H\}$ SSNMR spectra (162 MHz, 285 K, 10 kHz spin rate) of **A**: **[1-(3-methylpentane)][BAr$^F_4$]**, as prepared at 285 K. **B**: Spectrum collected after **20 minutes**. **C**: Spectrum collected after **90 minutes**. **D**: Spectrum collected after **16 hours**; where the rotor has been left under a flushing N_2 atmosphere during each timepoint. Peaks marked * are assigned to spinning sidebands, ‡ to **[1-BAr$^F_4$]**[S2] and ^ to unreacted **[1-(C_6H_{10})][BAr$^F_4$]** (C_6H_{10} = 3-methyl-1,3-pentadiene or 2-ethylbutadiene, see Figure S36). † is assigned to the dehydrogenation product from **[1-(3-methylpentane)][BAr$^F_4$]**, (See Section S.2.7.).

S.2.6. The partial dehydrogenation of [1-(3-methylpentane)][BAr^F_4]

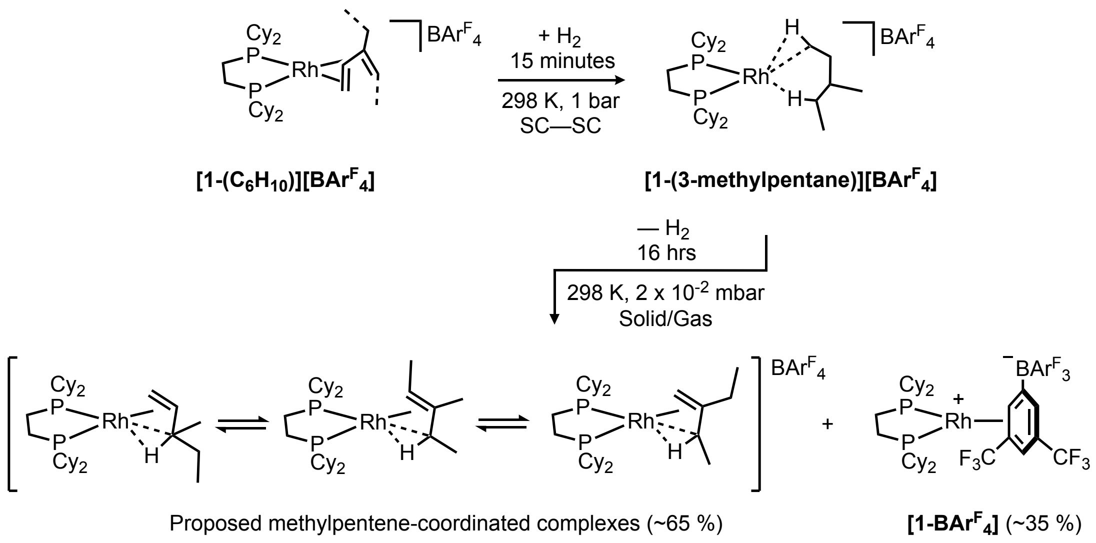

S.2.6.1. Proposed methylpentenes complex

As shown in Figure S42D, upon leaving **[1-(3-methylpentane)][BAr^F_4]** under a flushing N_2 atmosphere for 16 hours (unoptimised) at ambient temperature, a new signal in the $^{31}P\{^1H\}$ SSNMR spectra (δ 95.1) is observed growing in. This is assigned to a potential methylpentene-coordinated complex.

The solution $^{31}P\{^1H\}$ NMR spectra after this time showed a set of signals at [δ 93.4, d, J_{RhP} 180 Hz], consistent to that of an isobutene[S7] and 2-butene[S5] coordinated complex, as well as **[1-methylbutenes][BAr^F_4].** The doublet nature at 298 K suggests a low energy fluxional process, averaging the two ^{31}P environments, as stated for **[1-methylbutenes][BAr^F_4]** (Section S.2.4.).

This complex could not be isolated from the **[1-BAr^F_4]** decomposition product, inhibiting the full characterisation of this dehydrogenation product. The limited significant data gathered upon this complex is shown herein.

S.2.6.2. Charaterisation data and spectra

^{1}H NMR (CD_2Cl_2, 298 K, 400 MHz): δ 7.72 (s, 8H, *ortho*-ArH), 7.66 (s, 4H, *para*-ArH), 2.19-1.52 (br m, overlapping aliphatic CH), 1.44-0.80 (br m, aliphatic CH).

^{31}P{^{1}H} NMR (CD_2Cl_2, 298 K, 162 MHz): δ 92.9 (d, J_{RhP} = 179.9 Hz).
Note: Both **[1-methylbutenes][BAr^F$_4$]** (Section S.2.4.) and a previously reported isobutene coordinated complex,[S7] show analogues doublets in their ^{31}P{^{1}H} NMR spectra of [δ 94.4, d, J_{RhP} = 181 Hz] and [δ 95.3, d, J_{RhP} = 179 Hz] respectively.

^{31}P{^{1}H} SSNMR (162 MHz, 285 K, 10 kHz spin rate): δ 95.4 (br, apparent triplet).

Note: Both **[1-methylbutenes][BAr^F$_4$]** (Section S.2.4.) and a previously reported isobutene coordinated complex,[S7] show analogues resonances in the ^{31}P{^{1}H} SSNMR spectra at δ 94.6 and 94.8 respectively.

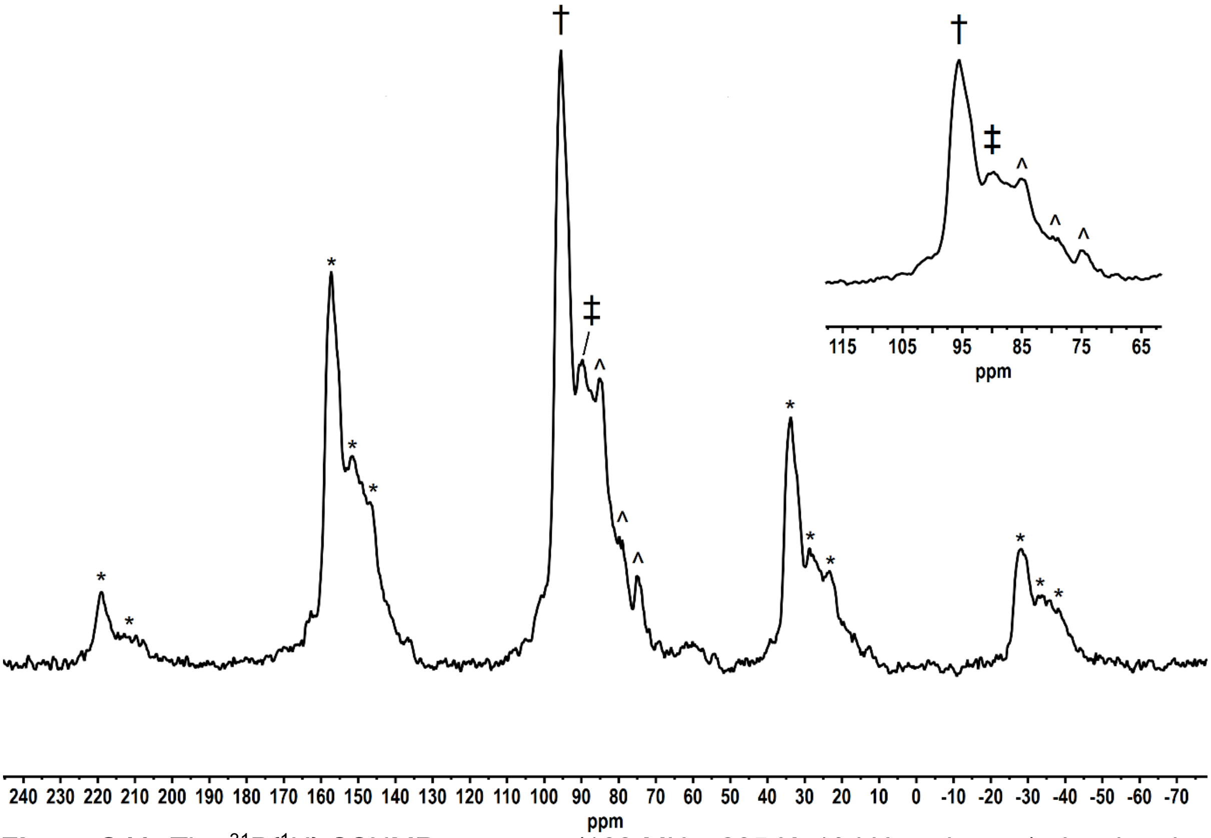

Figure S41: The ^{31}P{^{1}H} SSNMR spectrum (162 MHz, 285 K, 10 kHz spin rate) showing the possible methylpentenes-bound complex, labelled †. Peaks marked ‡ are assigned to **[1-BAr^F$_4$]**[S2] and ^ to **[1-(C_6H_{10})][BAr^F$_4$]** (C_6H_{10} = 3-methyl-1,3-pentadiene or 2-ethylbutadiene, see Figure S36) from incomplete hydrogenation of the sample. * are assigned to spinning sidebands.

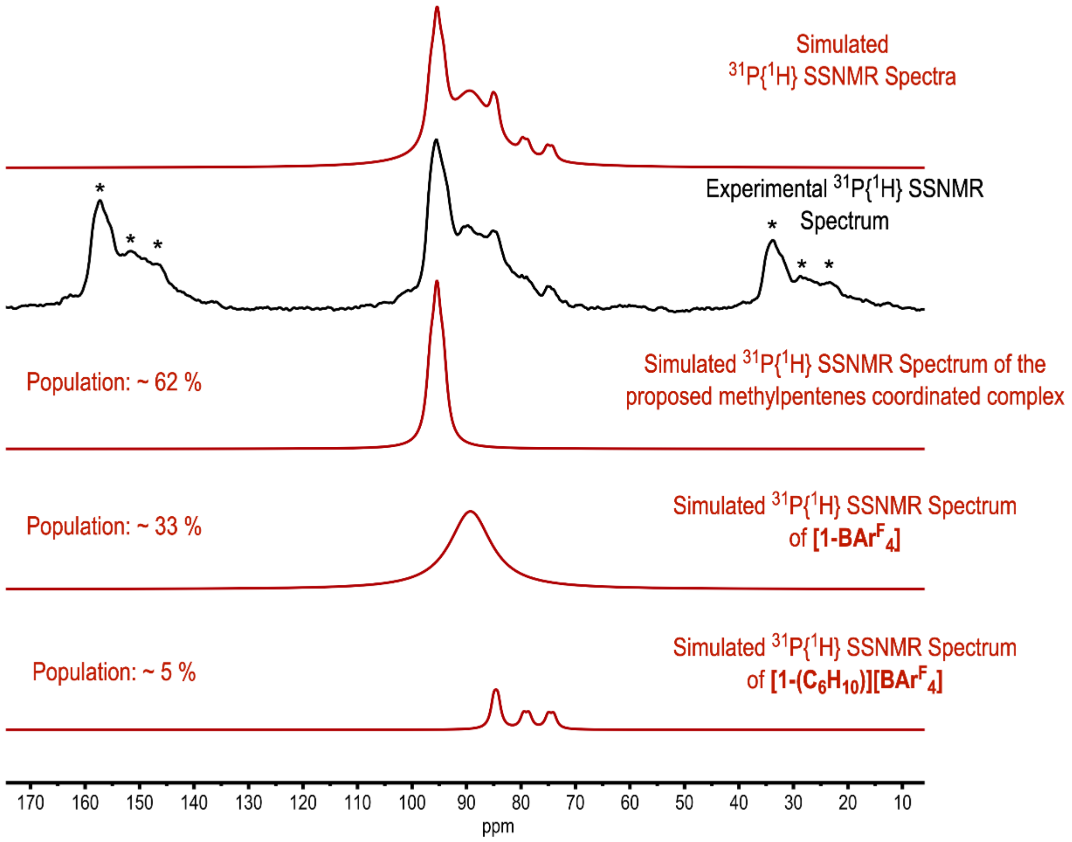

Figure S42: Simulated and experiment $^{31}P\{^1H\}$ SSNMR spectra of a potential methylpentene-complex convoluted with known signals from **[1-BAr$^F_4$]** and **[1-(C_6H_{10})][BAr$^F_4$]** (C_6H_{10} = 3-methyl-1,3-pentadiene or 2-ethylbutadiene). Peaks marked * are assigned to spinning sidebands. The simulated $^{31}P\{^1H\}$ SSNMR spectra, suggests an approx. 65: 35 ratios of methylpentene-complex and **[1-BAr$^F_4$]**.

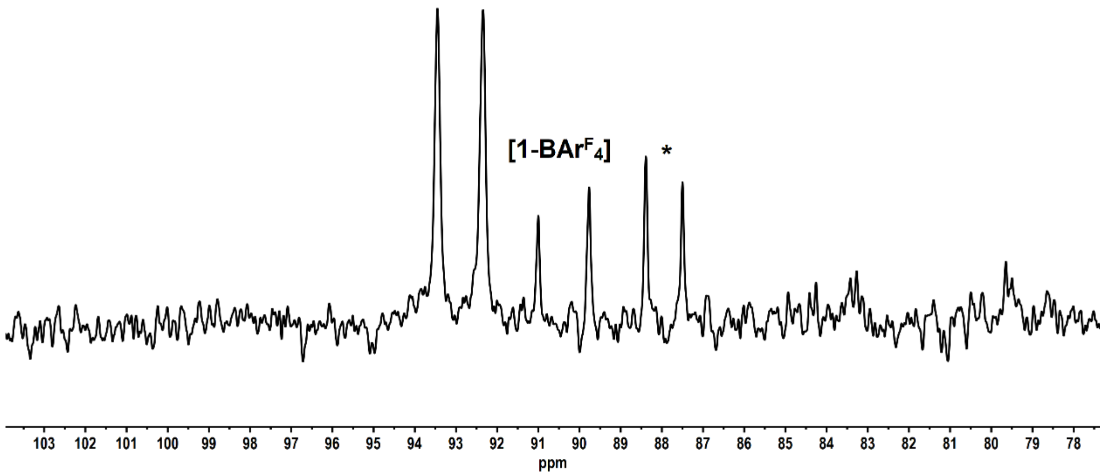

Figure S43: The solution $^{31}P\{^1H\}$ NMR spectrum (CD_2Cl_2, 298 K, 162 MHz) showing the possible methylpentene-bound complex (δ 93), **[1-BAr$^F_4$]** and * is assigned to a further decomposition product of **[1-BAr$^F_4$]** in CD_2Cl_2.[S5]

S.2.6.3. Liberation of bound methylpentenes

A J. Young flask was charged with a sample of the proposed methylpentene-complex mixture, as prepared from the same method described in Section S.2.4.3. Dissolution of the solid material in a C_6D_6/Acetone-d_6 mixture liberated the bound methylpentenes. The resultant organometallic complex was found to be the benzene-coordinated organometallic complex by $^{31}P\{^1H\}$ NMR spectroscopy [δ 99.7, d, J_{RhP} = 201 Hz].[S7]

The reaction volatiles could be isolated by trap-to-trap distillation and the ^1H NMR spectrum is shown in Figure S47, with assigned alkene-isomers highlighted. EI-MS of these volatiles confirmed the formation of C_6H_{12} [*m/z* found 84.0935 (calc. 84.0933)].

Although the exact ratios of methylpentene-isomers in the solid-state was not found, this solution trapping method suggests (E/Z)-3-methyl-2-pentene is the most populous isomer.

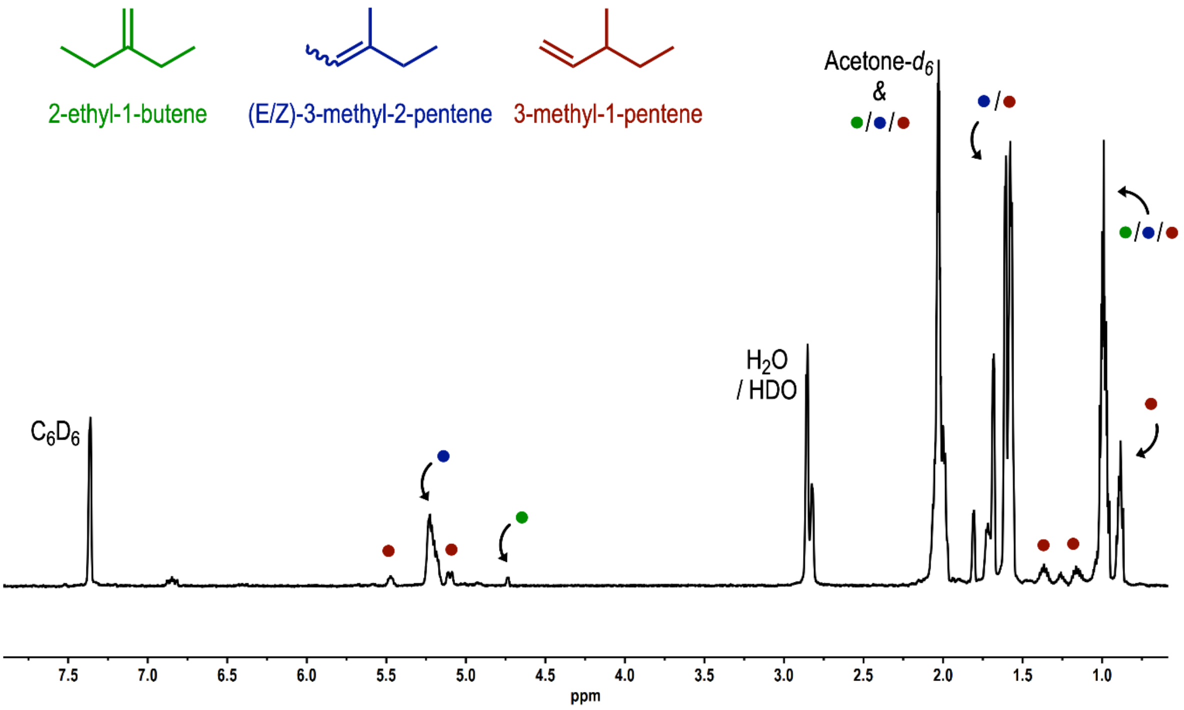

Figure S44: The solution ^1H NMR spectrum (CD_2Cl_2, 298 K, 500 MHz) of isolated alkene volatiles. Signals from the mixture of isomers of 2-ethyl-1-butene,[S12] (E/Z)-3-methyl-2-pentene[S13] and 3-methyl-1-pentene[S14] are marked respectively.

S.2.7. $[Rh(Cy_2PCH_2CH_2PCy_2)(C_8H_{14})][BAr^F_4]$, [1-octadiene][$BAr^F_4$]

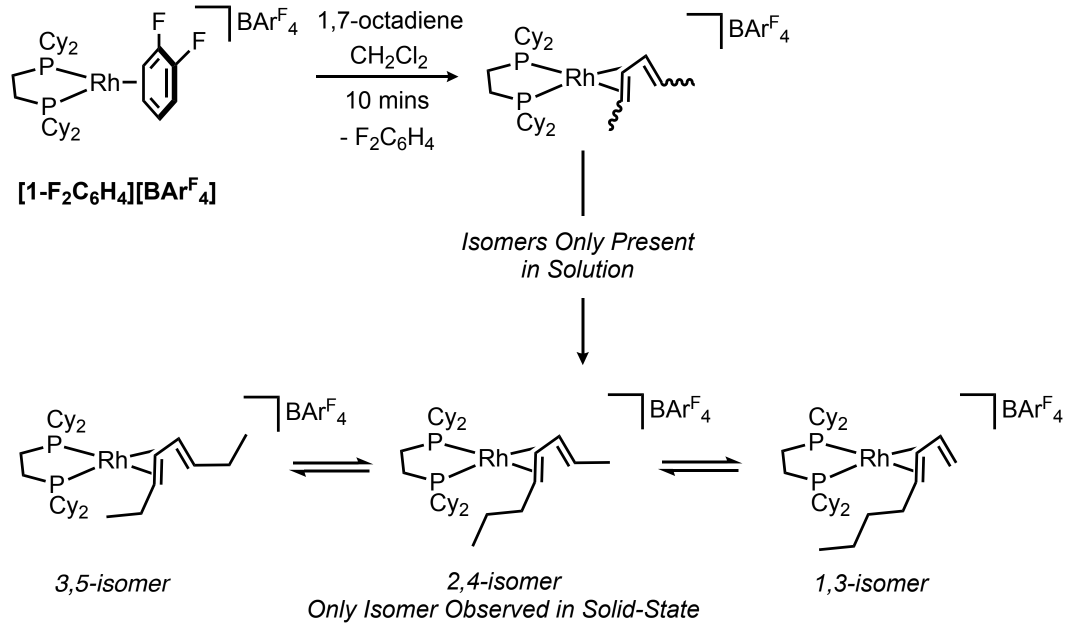

[1-octadiene][BAr^F_4]

S.2.7.1. Synthesis of an octodiene complex, [1-octadiene][BAr^F_4]

A pale-yellow solution of **[1-$F_2C_6H_4$][BAr^F_4]** (600 mgs, 0.40 mmol) in CH_2Cl_2 (20 mL) was treated with neat 1,7-octadiene (50 μL) at ambient temperature. The resultant deep red solution was stirred at ambient temperature for 15 minutes. Pentane (100 mL) was then added with vigorous stirring which resulted in the formation of a red precipitate. The solid was isolated by filtration and washed with pentane (2 × 20 mL). The solid was then dissolved in 1,2-dichloroethane (~12 mL) and filtered into a J. Young crystallisation flask and layered with pentane. Deep red crystals were obtained after storage for 1 week at room temperature of $[Rh(Cy_2PCH_2CH_2PCy_2)(C_8H_{14})][BAr^F_4]$, **[1-octadiene][$BAr^F_4$]** (Yield: 466 mgs, 78 %).

Note: The 2,4-isomer was found to be the only isomer present in the solid-state. Taking single-crystals of the 2,4-isomer and re-dissolving in CH_2Cl_2 initially showed only 2,4-isomer, before the mixture of isomers growing in within 5 minutes.

S.2.7.2. Charaterisation data and spectra for [1-octadiene][BAr$^F{}_4$]

^{1}H NMR (CD_2Cl_2, 298 K, 400 MHz): δ 7.72 (s, 8H, *ortho*-ArH), 7.57 (s, 4H, *para*-ArH), 6.65, 6.30, 5.56, 5.36, 5.25, 4.16, 4.02, 2.78 (m, multiple alkene resonances from all isomers), 2.47-0.98 (br m, overlapping aliphatic CH).

^{31}P{^{1}H} NMR (CD_2Cl_2, 298 K, 162 MHz): 3,5-isomer: δ 80.7 (d, J_{RhP} = 178 Hz). 2,4-isomer: δ 80.9 (d, J_{RhP} = 178 Hz, J_{PP} = 24 Hz), δ 80.6 (d, J_{RhP} = 178 Hz, J_{PP} = 24 Hz). 1,3-isomer: δ 84.5 (d, J_{RhP} = 175 Hz, J_{PP} = 22 Hz), δ 77.3 (d, J_{RhP} = 173 Hz, J_{PP} = 22 Hz).

^{19}F{^{1}H} NMR (CD_2Cl_2, 298 K, 377 MHz): δ -62.9 (s).

ESI-MS (calc. for $[Rh(Cy_2PCH_2CH_2PCy_2)(C_8H_{14})]^+$): *m/z* 635.3329 (635.3376).
Elemental Analysis (calc. for $C_{66}H_{74}BF_{24}P_2Rh$): C 52.73 (52.88), H 4.87 (4.98).

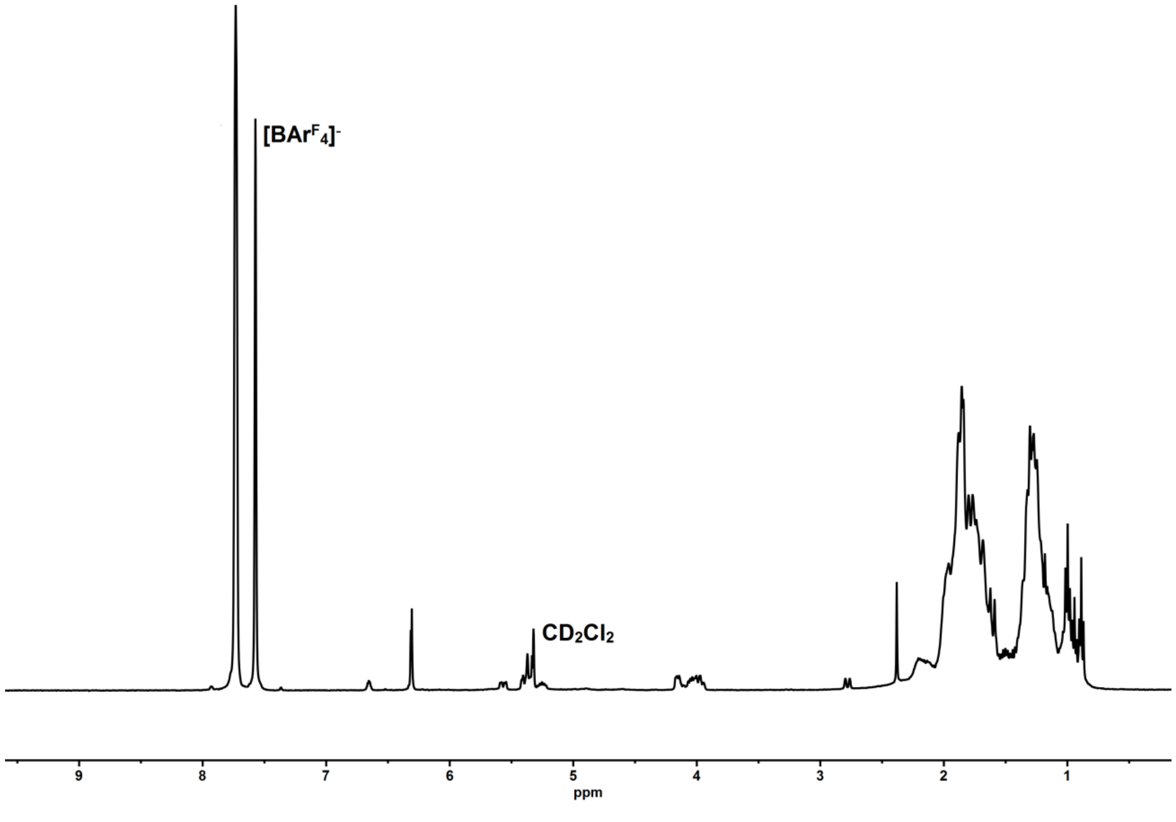

Figure S45: The solution ^{1}H NMR spectrum (CD_2Cl_2, 298 K, 400 MHz) of **[1-octadiene][BAr$^F{}_4$]**.

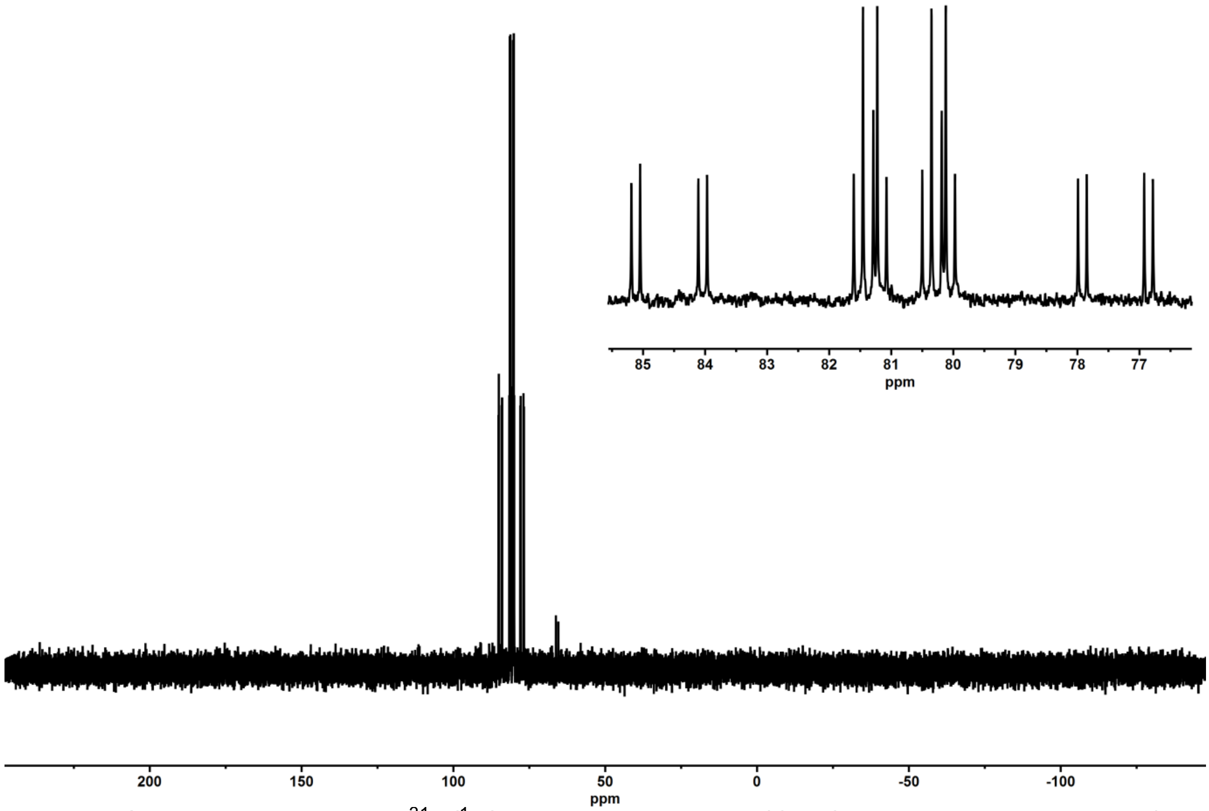

Figure S46: The solution $^{31}P\{^{1}H\}$ NMR spectrum (CD_2Cl_2, 298 K, 162 MHz) of **[1-octadiene][BAr$^{F}_{4}$]**. The inset is an enlargement of the resonances between δ 85 and 77.

S.2.7.3. Attempted synthesis of ethane and octane complexes

Following the previously stated procedures in Sections S.2.3.3 and S.2.4.3., addition of H_2 (1 bar, 298 K, 10 minutes) to ruby red crystals the previously reported bis-ethene complex,[S5] $[Rh(Cy_2PCH_2CH_2PCy_2)(C_2H_4)_2][BAr^F_4]$ **$[1\text{-(ethene)}_2][BAr^F_4]$** or **$[1\text{-octadiene}][BAr^F_4]$** resulted in the crystals changing colour change to light yellow. It was found in both cases these yellow crystals did not diffract in single-crystal X-ray diffraction experiments.

Dissolution of this product in CD_2Cl_2 shows the complete hydrogenation of **$[1\text{-(ethene)}_2][BAr^F_4]$** and **$[1\text{-octadiene}][BAr^F_4]$**, as no signals relating to **$[1\text{-(ethene)}_2][BAr^F_4]$** or **$[1\text{-octadiene}][BAr^F_4]$** were observed in the respective solution $^{31}P\{^1H\}$ NMR spectra. Trap-to-trap distillation of the volatiles from these solutions and analysis by ^1H NMR spectroscopy confirmed the formation of ethane and octane respectively.

Additionally for **$[1\text{-(ethene)}_2][BAr^F_4]$**, SSNMR spectroscopy was attempted following the same procedure used in Section S.2.1.2. for **$[1\text{-propane}][BAr^F_4]$**. Only signals relating to the decomposition product of **$[1\text{-}BAr^F_4]$** were observed ($^{31}P\{^1H\}$ SSNMR: δ 91).[S2]

For **$[1\text{-octadiene}][BAr^F_4]$**, any reduction in the time of hydrogenation (< 10 minutes) resulted in a mix of **$[1\text{-octadiene}][BAr^F_4]$** starting material (see Section S.2.8.2.) and **$[1\text{-}BAr^F_4]$** (δ 91, d, J_{RhP} = 175 Hz)[S5] product by solution $^{31}P\{^1H\}$ NMR spectroscopy.

S.3 Crystallographic and Refinement Data

S.3.1. Crystal structure determinations

Regardless of collection method (described below), all raw frame data were reduced using CrysAlisPro.[S15] The structures were solved using SHELXT[S16] and refined using full-matrix least squares refinement on all F^2 data using the SHELXL-18[S17] using the interface OLEX2.[S18] In all structures, disorder of the -CF_3 groups was treated by introducing a split site model and restraining geometries and displacement parameters. Distances and angles were calculated using the full covariance matrix.

S.3.1.1. Diamond Light Source

Gas cell: The I19 1mm quartz capillary gas cell comprises of a quartz capillary (Hampton research HR6-148, OD 1 mm, ID 0.98 mm, L 25 mm) which is glued into a brass specimen pin (Hampton research HR4-661) using silver epoxy glue and the open end cut to length using a capillary cutting stone (Hampton research HR4-334). The other half of the cell is a 1/8 to 1/16 inch reducing union with 1/16 inch stainless steel capillary tubing (Swagelok SS-T1-S-014-6ME) to a miniature quick connector (Swagelok SS-QM2-D-100).

Due to the air sensitivity of the systems under investigation the mounting procedure occurs under an inert atmosphere within a glove box. Without the use of manipulation oil, a crystal is selected and mounted, using a minimal amount of epoxy glued, to the tip of a sample mount (mitogen M2-L25SP-30). The mount is then placed into the capillary and fixed in place with a small amount of glue. The two halves of the gas cell assembled creating a gas tight environment for the sample ready to be mounted on the diffractometer and connected to gas rig.

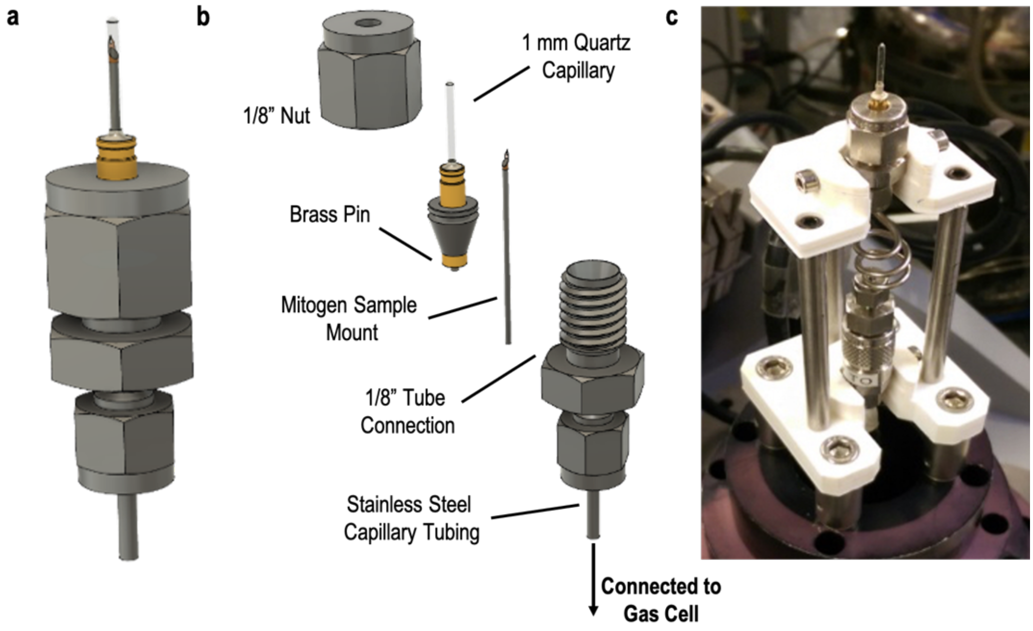

Figure S47: I19 Beamline 1 mm quartz capillary gas cell equipment. **a**) Technical drawing of the head of the cell with **b**) showing the exploded view. **c**) Image of the diffractometer mounted cell. Image adapted with kind permission from Dr Mark Warren, Diamond Light Source.

Gas Rig: The I19 gas-cell control panel allows the users to operate the gas flow, gas pressure, gas mixture and vacuum remotely. The main rig has several pneumatic valves (V1-6), for connections to samples (S1), sample return (SR1), gases (G1-3), vacuum (Vac1), pressure transducers (PR1-2) and exhaust (EX1) (Figure S51).

The vacuum pump is a scroll pump with a turbo attachment capable of reducing the pressure to 10^{-6} mbar at the pump transducer and 10^{-4} mbar at the sample position. Gas pressure, flows and mixtures can be controlled by the mass flow controllers (Alicat MC-200SCCM-D) and pneumatic valves (V1-3).

The gas-cell on the diffractometer is attached to the control apparatus *via* 1/16 inch tubing with quick release connectors. The quick release connectors allow users to rapidly and easily attach and remove the cell whilst maintaining the pressure in the cell. The setup of this apparatus allows for the gas-cell to be subjected to vacuum, single gases or gas mixtures either under flow of the gas or with gas pressures up to 200 bar in-situ with the diffraction experiment. The alicat mass flow controllers, pneumatic valves and transducers can be controlled and read through the supporting control software.

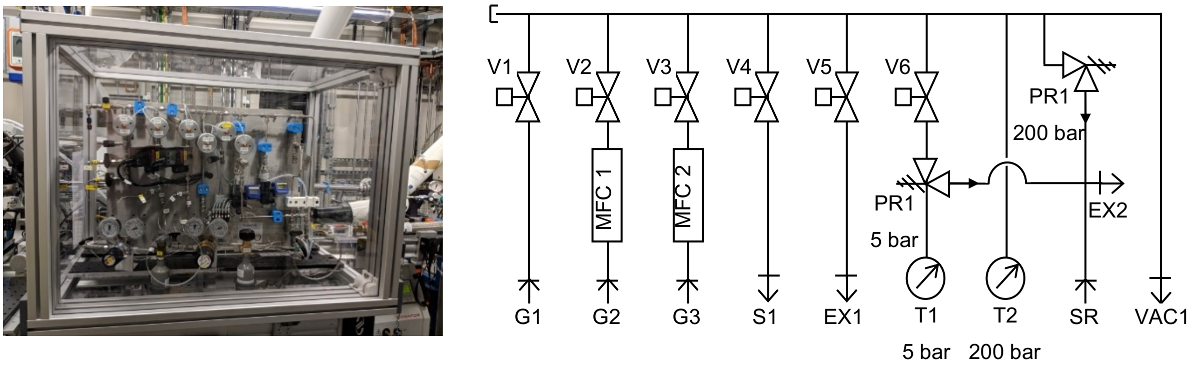

Figure S48: Gas-cell control apparatus schematic. Key: V1-6 pneumatic valves, MFC1-2 alicat mass flow controllers, G1-3 gas inlets, S1 sample connection, EX1-2 exhaust connection, T1-2 pressure transducers, PR1-2 pressure relief valves and VAC1 vacuum connection.

Using the above set-up, the structure of **[1-propane][BAr$^F_4$]** (150 K) was collected on a Pilatus 300K hybrid pixel diffractometer with synchrotron source Zr K-edge (17.998 keV which equates to λ = 0.6889 Å) on Station I19 at the Diamond Light Source (DLS).[S6]

S.3.1.2. University of Oxford

The structures of **[1-isoprene][BAr$^F_4$]**, **[1-(2-methylbutane)][BAr$^F_4$]**, **[1-hexadiene][BAr$^F_4$]**, **[1-hexane][BAr$^F_4$]**, **[1-(C_6H_{10})][BAr$^F_4$]**, **[1-(3-methylpentane)][BAr$^F_4$]** and **[1-octadiene][BAr$^F_4$]** (all at 150 K) were collected at the Oxford Chemical Crystallography Service from the University of Oxford, with an Agilent SuperNova diffractometer (Cu K_α radiation, λ = 1.54180 Å). Single crystal X-ray diffraction data for all samples were collected as follows: a selected crystal was mounted on a MiTeGen Micromounts using perfluoropolyether oil and cooled rapidly to the collection temperature in a stream of nitrogen gas using an Oxford Cryosystems Cryostream unit.[S19]

S.3.1.3. National Crystallography Service, Southampton, UK

[1-methylbutenes][BAr$^F_4$] (150 K) was collected at the National Crystallography Service, University of Southampton, Southampton, UK, using a Rigaku 007HF diffractometer equipped with Varimax confocal mirrors (Cu K_α radiation, λ = 1.54184 Å), an AFC11 goniometer, and HyPix 6000 detector.

S.3.2. Additional comments on solved structures

The structures of **[1-(2-methylbutane)][BAr$^F_4$]** and **[1-methylbutenes][BAr$^F_4$]** were refined as a 2-component twins; rotated by -179.9945° around 0.71 0.71 -0.00 (reciprocal space) or 0.73 0.69 0.05 (direct space) with BASF refined to 0.4997(13) for **[1-(2-methylbutane)][BAr$^F_4$]** and rotated by -179.9799° around 0.71 0.71 -0.00 (reciprocal space) or 0.72 0.69 0.03 (direct space) with BASF refined to 0.899(2) for **[1-methylbutenes][BAr$^F_4$]**.

S.3.3. Refinement tables

Table S1: Selected crystallographic and refinement data.

	**[1-propane] [BAr$^F_4$]**	**[1-isoprene] [BAr$^F_4$]**	**[1-methylbutenes] [BAr$^F_4$]**	**[1-(2-methylbutane)] [BAr$^F_4$]**
Chemical formula	$C_{61}H_{68}BF_{24}P_2Rh$	$C_{63}H_{68}BF_{24}P_2Rh$	$C_{63}H_{70}BF_{24}P_2Rh$	$C_{63}H_{72}BF_{24}P_2Rh$
Formula weight	1432.81	1456.83	1458.85	1460.86
Temperature (K)	150	150	100	150
Crystal system	triclinic	triclinic	triclinic	triclinic
Space group	P-1	P-1	P-1	P-1
a (Å)	12.9862(8)	12.7425(3)	12.7343(2)	12.7559(6)
b (Å)	13.0857(8)	12.9552(3)	13.0256(2)	13.1978(5)
c (Å)	19.9409(11)	20.0752(4)	19.8332(4)	19.7970(5)
α (deg)	91.206(5)	92.435(2)	92.6533(16)	93.734(2)
β (deg)	90.367(5)	91.227(2)	91.1282(16)	92.418(3)
γ (deg)	96.291(5)	97.363(2)	96.6525(15)	95.556(3)
V (Å^3)	3367.3(4)	3282.54(13)	3263.06(11)	3306.2(2)
Z	2	2	2	2
ρ(calcd) (g cm^{-3})	1.413	1.474	1.485	1.467
μ (mm^{-1})	0.374	3.519	3.540	3.494
Reflections collected	16866	51527	13689	26345
Unique reflections	6569	13739	13689	26345
Restraints / Parameters	1212/1028	399/999	508/956	571/975
R_{int}	0.0532	0.0512		
R_1 [$I > 2\sigma(I)$]	0.1073	0.0575	0.0861	0.0930
wR_2 [all data]	0.2812	0.1679	0.2666	0.2622
GooF	1.100	1.019	1.145	0.997
Residual electron density (e Å^{-3})	2.25/-0.78	1.32/-0.71	0.93/-1.74	3.40/-1.02
CCDC no.	2056862	2056864	2056859	2056863

	[1-hexadiene] [BAr$^F_4$]	[1-hexane] [BAr$^F_4$]	[1-(C_6H_{10})] [BAr$^F_4$]^a	[1-(3-methylpentane)] [BAr$^F_4$]
Chemical formula	$C_{64}H_{70}BF_{24}P_2Rh$	$C_{64}H_{74}BF_{24}P_2Rh$	$C_{64}H_{70}BF_{24}P_2Rh$	$C_{64}H_{74}BF_{24}P_2Rh$
Formula weight	1470.86	1474.89	1470.86	1474.89
Temperature (K)	150	150	150	150
Crystal system	triclinic	triclinic	triclinic	triclinic
Space group	P-1	P-1	P-1	P-1
a (Å)	12.8807(3)	13.1078(2)	12.7317(7)	12.6885(2)
b (Å)	13.3277(4)	13.6868(3)	13.1412(6)	13.2056(2)
c (Å)	20.1295(6)	19.1309(3)	20.1179(7)	20.0728(3)
α (deg)	101.803(2)	99.0834(15)	94.535(5)	94.5410(10)
β (deg)	97.376(2)	95.1697(14)	92.043(4)	91.6930(10)
γ (deg)	100.681(2)	99.1623(15)	96.288(4)	95.6740(10)
V (Å^3)	3273.92(16)	3322.42(10)	3331.9(3)	3334.03(9)
Z	2	2	2	2
ρ(calcd) (g cm^{-3})	1.492	1.474	1.466	1.469
μ (mm^{-1})	3.543	3.483	3.473	3.471
Reflections collected	35413	36508	28618	62097
Unique reflections	13553	13707	13907	13909
Restraints / Parameters	571/1010	597/1003	1491/1112	710/983
R_{int}	0.0356	0.0320	0.0376	0.0337
R_1 [$I > 2\sigma(I)$]	0.0402	0.0408	0.0726	0.0483
wR_2 [all data]	0.1064	0.1108	0.2144	0.1295
GooF	1.030	1.021	1.047	1.048
Residual electron density (e Å^{-3})	1.42/-0.95	0.92/-0.91	1.49/-1.33	1.29/-1.05
CCDC no.	2056866	2056857	2056860	2056858

a = C_6H_{10} = 3-methyl-1,3-pentadiene or 2-ethylbutadiene.

	**[1-octadiene] [BAr$^F_4$]**
Chemical formula	$C_{66}H_{74}BF_{24}P_2Rh$
Formula weight	1498.91
Temperature (K)	150
Crystal system	monoclinic
Space group	$P2_1/c$
a (Å)	22.8949(2)
b (Å)	13.1174(1)
c (Å)	25.4290(10)
α (deg)	90
β (deg)	114.565(1)
γ (deg)	90
V (Å^3)	6945.67(13)
Z	4
ρ(calcd) (g cm^{-3})	1.433
μ (mm^{-1})	3.342
Reflections collected	84898
Unique reflections	14458
Restraints / Parameters	601/1016
R_{int}	0.0357
R_1 [$I > 2\sigma(I)$]	0.0477
wR_2 [all data]	0.1387
GooF	1.018
Residual electron density (e Å^{-3})	1.38/-0.70
CCDC no.	2056865

S.3.4. Structural images from single crystal X-ray diffraction experiments

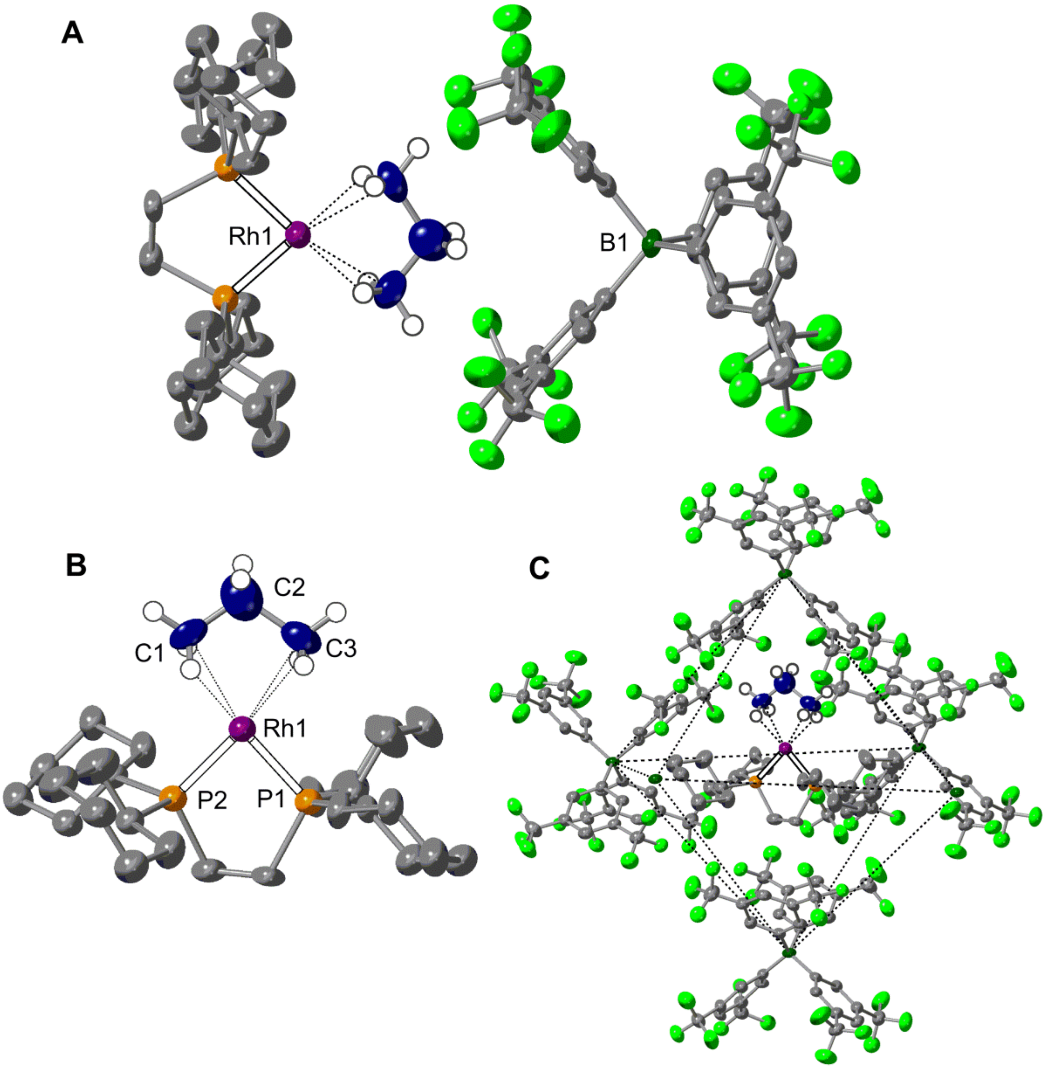

Figure S49: (A) Molecular structure of **[1-propane][BAr^F$_4$]**. **(B)** Cationic fragment of **[1-propane][BAr^F$_4$]** showing atom labelling. **(C)** Structure displaying pseudo-O_h anion network of the [BAr^F$_4$]$^-$ ion, with cationic fragment sat within the cavity; –(Ar^F$_4$) removed from 2 boron atoms for clarity. Displacement ellipsoids set at 50 % and hydrogen from phosphine ligand and [BAr^F$_4$]$^-$ anion removed for clarity in all parts.

Selected bond lengths (Å): Rh1-P1 2.206(4), Rh1-P2 2.226(6), Rh1-C1 2.459(19), Rh1-C3 2.453(19), C1-C2 1.54(2), C2-C3 1.52(2).

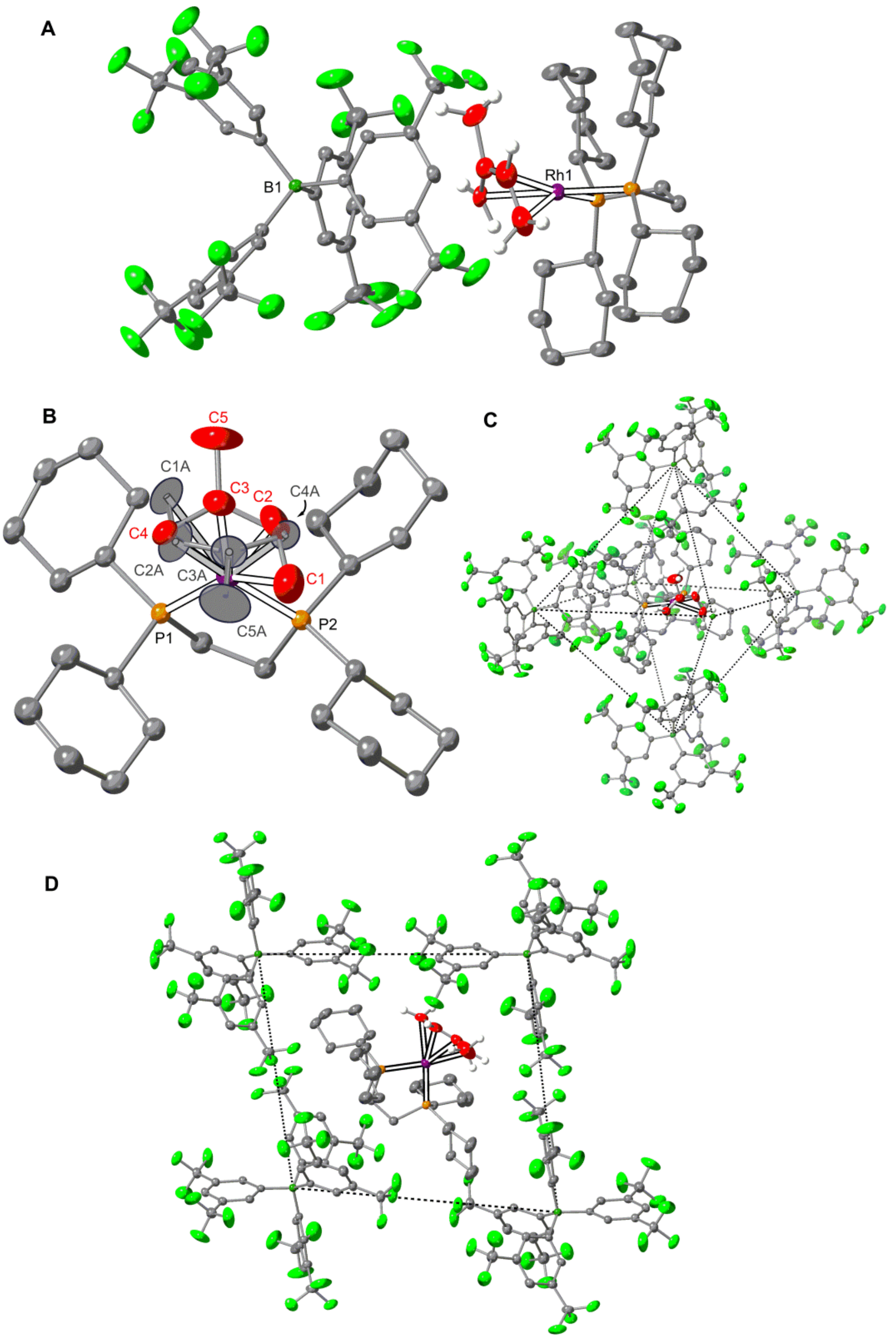

Figure S50: (A) Molecular structure of **[1-isoprene][BAr^F$_4$]**. **(B)** Cationic fragment of **[1-isoprene][BAr^F$_4$]** showing major disorder components, where the ratio of non-crystallographically imposed disorder components is 0.51: 0.49 for major: minor. **(C)** Structure displaying pseudo-O_h anion network of the [BAr^F$_4$]$^-$ ions, with cationic fragments sat within the cavity; –(Ar^F$_4$) removed from 1 boron atom for clarity. **(D)** Top view of pseudo-O_h anion-cage, showing linear cation/anion orientation. Displacement ellipsoids set at 50 % and hydrogen from phosphine ligand and **[BAr^F$_4$]$^-$** anion removed for clarity in all parts.

Selected bond lengths (Å): Rh1-P1 2.2815(9), Rh1-P2 2.2708(9), Rh1-C1 2.279(16), Rh1-C2 2.167(8), Rh1-C3 2.117(19), Rh1-C4 2.313(9), C1-C2 1.34(2), C3-C4 1.31(2).

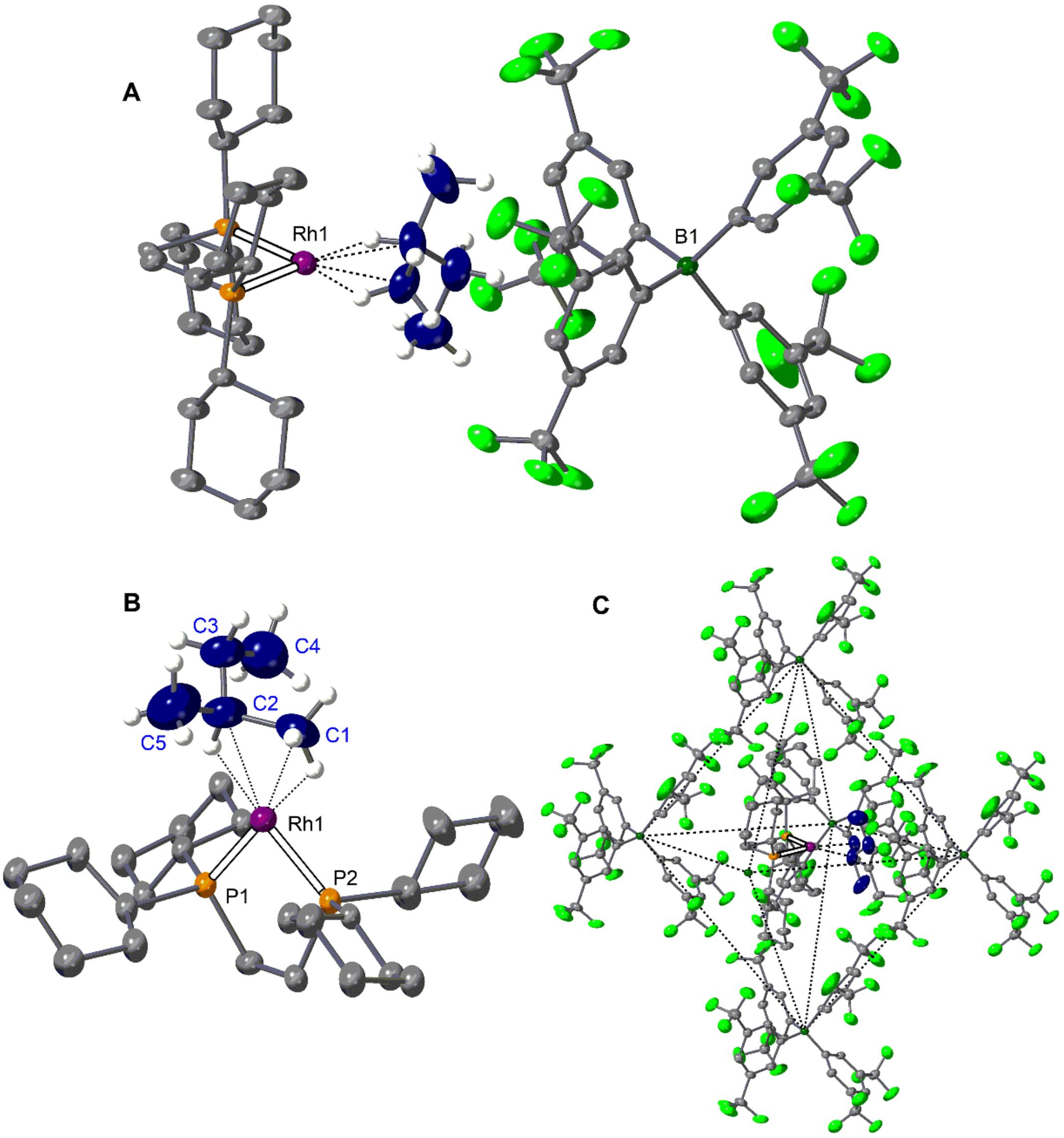

Figure S51: (A) Molecular structure of **[1-(2-methylbutane)][BAr$^F{}_4$]**. **(B)** Cationic fragment of **[1-(2-methylbutane)][BAr$^F{}_4$]** showing expanded cation view and atom labelling. **(C)** Structure displaying pseudo-O_h network of the [BAr$^F{}_4$]$^-$ ions, with cationic fragments sat within the cavity; –(Ar$^F{}_4$) removed from 4 boron atoms for clarity. Displacement ellipsoids set at 50 % and hydrogen from phosphine ligand and [BAr$^F{}_4$]$^-$ anion removed for clarity in all parts.

Selected bond lengths (Å): Rh1-P1 2.1869(16), Rh1-P2 2.1848(15), Rh1-C1 2.348(9), Rh1-C2 2.391(10), C1-C2 1.599(13), C2-C3 1.456(14), C2-C5 1.551(15), C3-C4 1.512(16).

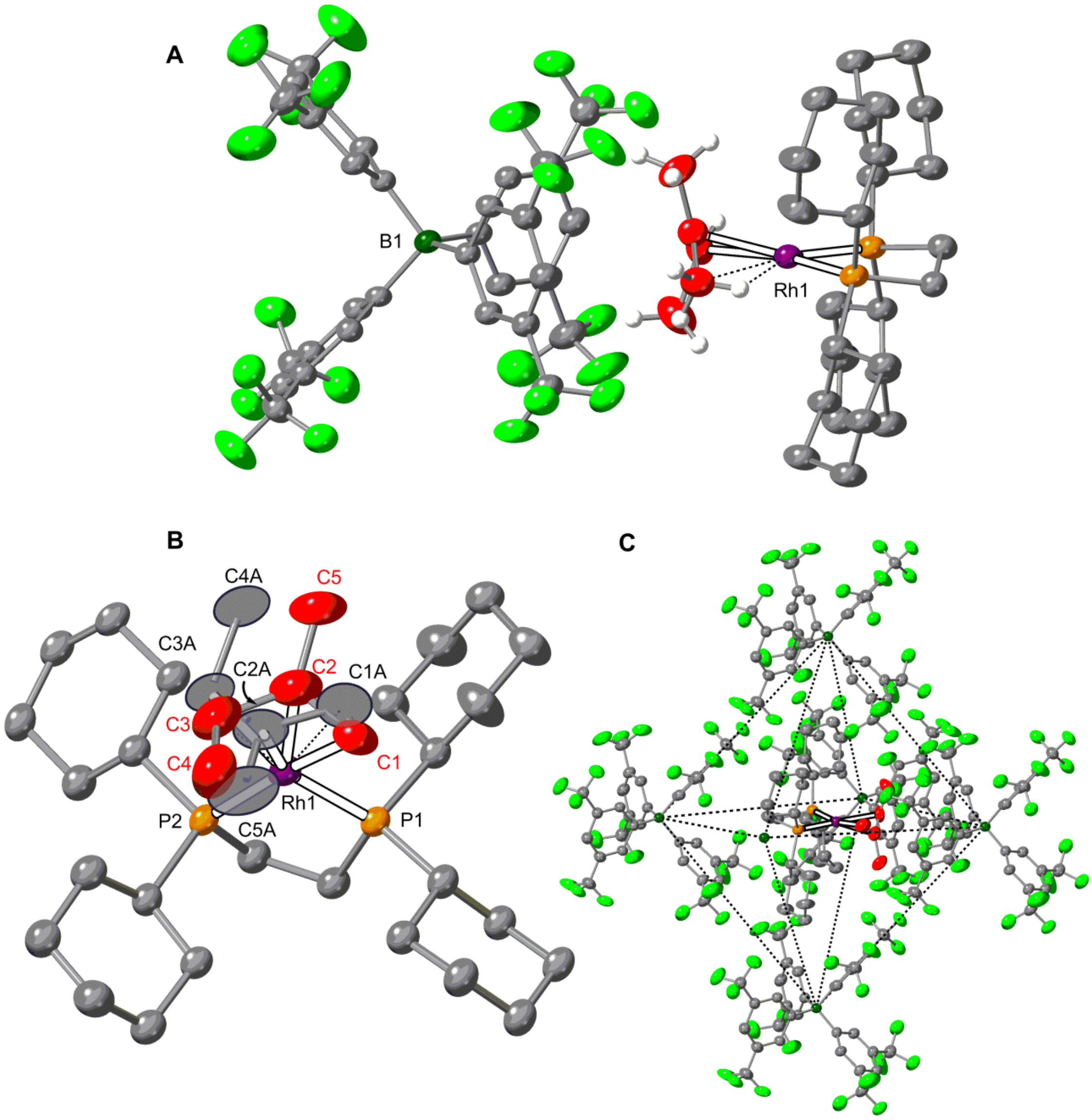

Figure S52: (A) Molecular structure of **[1-methylbutenes][BAr^F$_4$]**. **(B)** Cationic fragment of **[1-methylbutenes][BAr^F$_4$]** showing two disorder components in 50: 50 ratio. One component has been solved as 2-methylbut-1-ene and the other 2-methylbut-2-ene. There was no significant difference in final R-value between solving either disorder component as either methylbutene isomer. **(C)** Structure displaying pseudo-*O*$_h$ anion network of the [BAr^F$_4$]$^-$ ions, with cationic fragments sat within the cavity; –(Ar^F$_4$) removed from 1 boron atom for clarity. Displacement ellipsoids set at 50 % and hydrogen from phosphine ligand and [BAr^F$_4$]$^-$ anion removed for clarity in all parts.

Selected bond lengths (Å): Rh1-P1 2.2370(15), Rh1-P2 2.2314(16), Rh1-C1 2.23(2), Rh1-C2 2.120(19), Rh1-C3 2.360(18), Rh1-C1A 2.41(3), Rh1-C2A 2.11(2), Rh1-C3A 2.27(16), C1-C2 1.39(3), C2-C3 1.52(3), C1A-C2A 1.54(3), C2A-C3A 1.37(3).

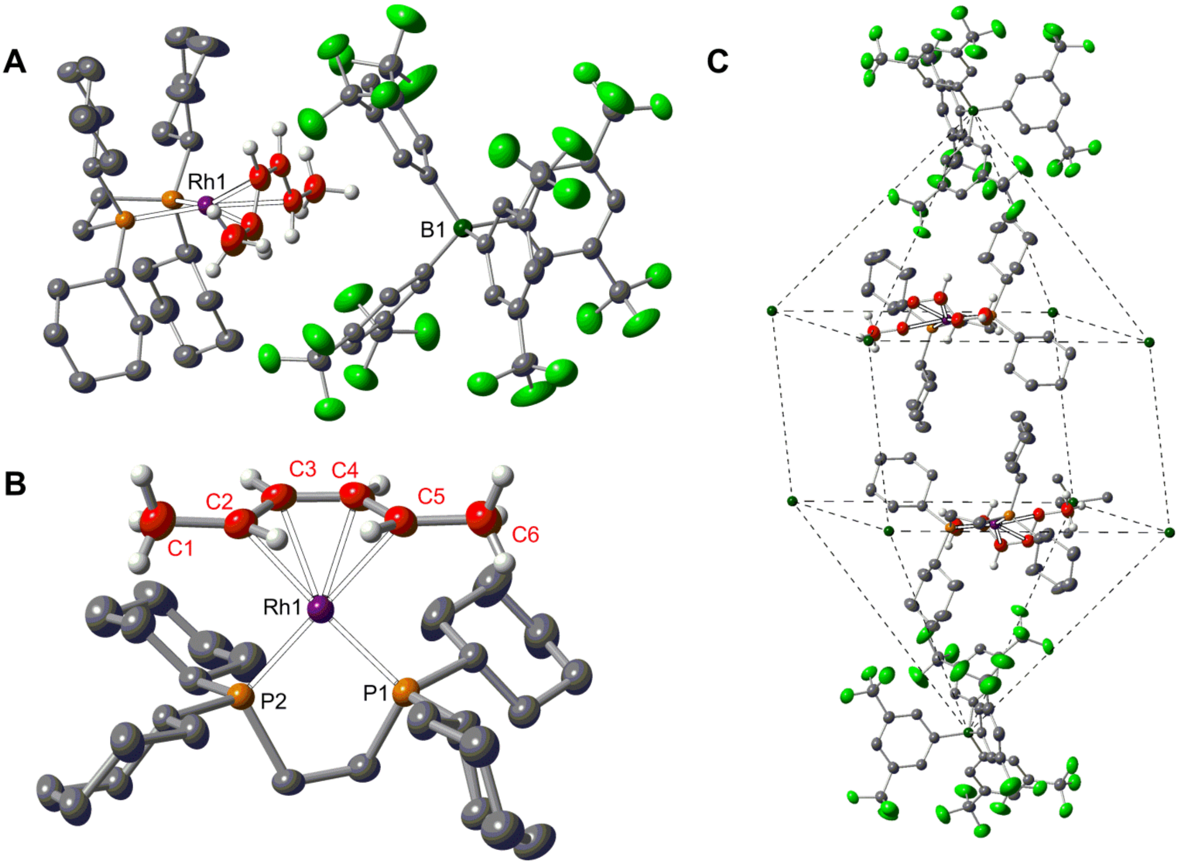

Figure S53: (A) Molecular structure of **[1-hexadiene][BAr^F$_4$]**. **(B)** Cationic fragment of **[1-hexadiene][BAr^F$_4$]** showing atom labelling. **(C)** Structure displaying bi-capped pyramidal anion network of the [BAr^F$_4$]$^-$ ions, with cationic fragments sat within the cavity; –(Ar^F$_4$) removed from 4 boron atoms for clarity. Displacement ellipsoids set at 50 % and hydrogen from phosphine ligand and [BAr^F$_4$]$^-$ anion removed for clarity in all parts.

Selected bond lengths (Å): Rh1-P1 2.2755(9), Rh1-P2 2.2689(6), Rh1-C2 2.328(3), Rh1-C3 2.184(3), Rh1-C4 2.197(3), Rh1-C5 2.337(3), C2-C3 1.357(4), C4-C5 1.352(5).

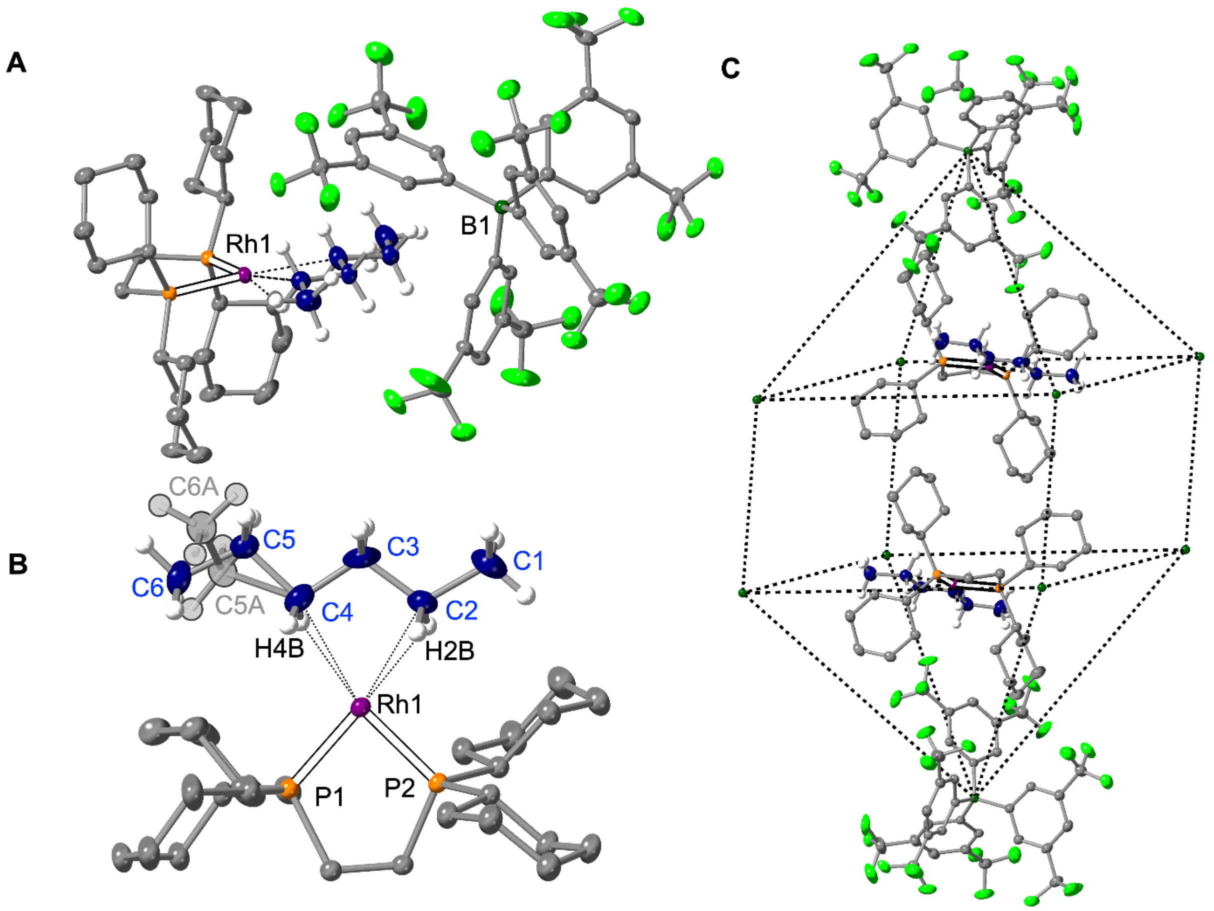

Figure S54: (A) Molecular structure of **[1-hexane][BAr$^F{}_4$]**. **(B)** Cationic fragment of **[1-hexane][BAr$^F{}_4$]** showing major disorder components, where the ratio of disorder components is 0.65: 0.35 for major: minor. **(C)** Structure displaying bi-capped pyramidal anion network of the [BAr$^F{}_4$]$^-$ ions, with cationic fragments sat within the cavity; –(Ar$^F{}_4$) removed from 4 boron atoms for clarity. Displacement ellipsoids set at 50 % and hydrogen from phosphine ligand and [BAr$^F{}_4$]$^-$ anion removed for clarity in all parts.

Selected bond lengths (Å): Rh1-P1 2.2002(6), Rh1-P2 2.1910(6), Rh1-C2 2.527(3), Rh1-C4 2.549(4), Rh1-H2B 2.11(5), Rh1-H4B 2.14(5), Rh1-H2A 2.46(5), Rh1-H4A 2.37(5), C1-C2 1.544(15), C2-C3 1.511(5), C3-C4 1.504(16), C4-C5 1.528(6), C5-C6 1.460(8).

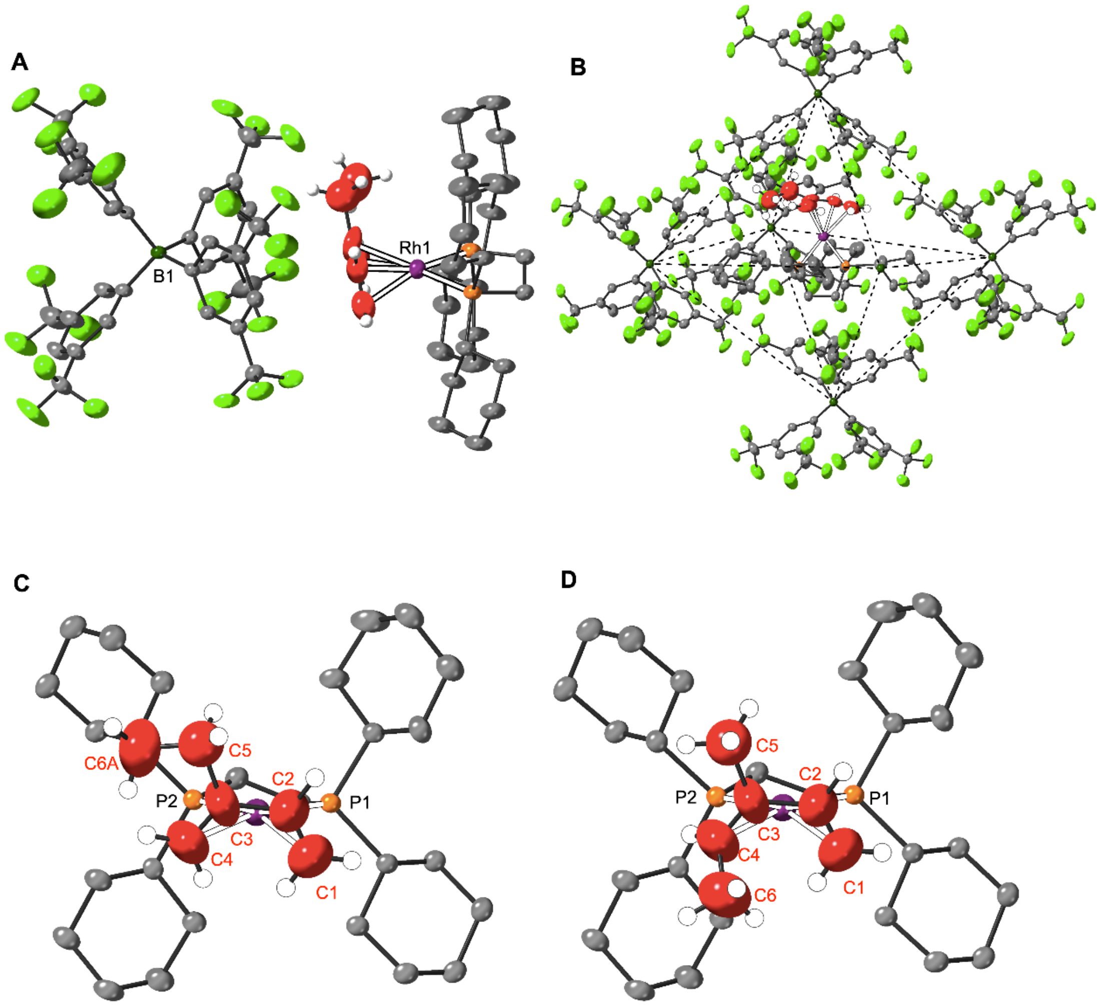

Figure S55: **(A)** Molecular structure of **$[1\text{-}(C_6H_{10})][BAr^F{}_4]$** ($C_6H_{10}$ = 3-methyl-1,3-pentadiene or 2-ethylbutadiene). **(B)** pseudo-O_h anion network of the $[BAr^F{}_4]^-$ ions, with cationic fragments sat within the cavity; –($Ar^F{}_4$) removed from 1 boron atom for clarity. **(C)** Cationic fragment of **$[1\text{-}(C_6H_{10})][BAr^F{}_4]$** showing 2-ethylbutadiene coordinated isomer, and **(D)** showing 3-methyl-1,3-pentadiene coordinated isomer. These are solved as disorder components of one another, where the ratio of disorder components is 0.49: 0.51 ratio respectively. Displacement ellipsoids set at 50 % and hydrogen from phosphine ligand and $[BAr^F{}_4]^-$ anion removed for clarity in all parts.

Selected bond lengths (Å): Rh1-P1 2.2628(12), Rh1-P2 2.2924(14), Rh1-C1 2.237(10), Rh1-C2 2.101(8), Rh1-C3 2.161(7), Rh1-C4 2.287(9), C1-C2 1.278(14), C2-C3 1.503(14), C3-C4 1.245(13), C3-C5 1.502(17), C4-C6 1.37(3), C5-C6A 1.48(3).

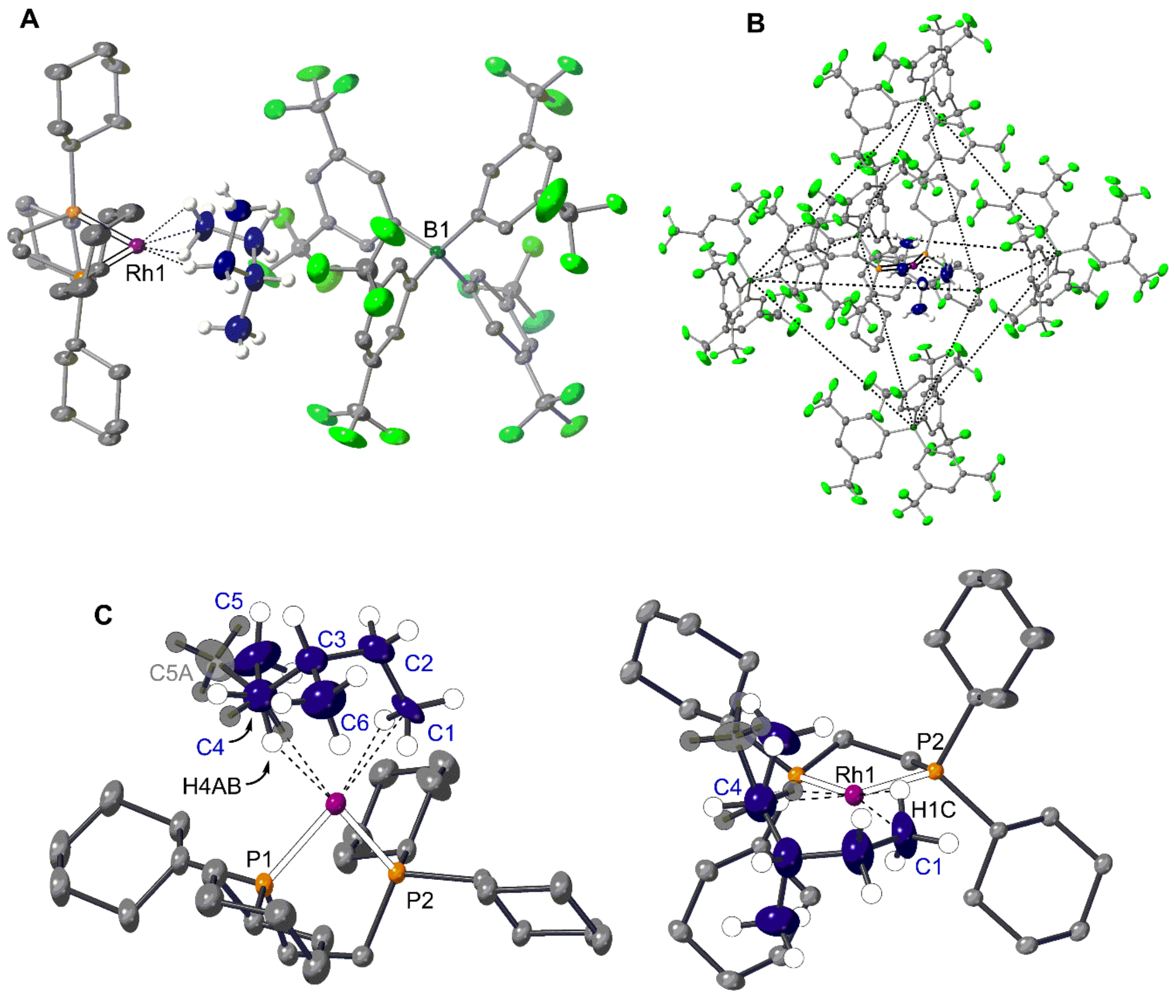

Figure S56: (A) Molecular structure of **[1-(3-methylpentane)][BAr$^{F}{}_{4}$]**. **(B)** Structure displaying pseudo-O_h anion network of the [BAr$^{F}{}_{4}$]$^{-}$ ions, with cationic fragments sat within the cavity; –(Ar$^{F}{}_{4}$) removed from 1 boron atom for clarity. **(C)** Cationic fragment of **[1-(3-methylpentane)][BAr$^{F}{}_{4}$]** showing atom labelling. showing major disorder components, where the ratio of disorder components is 0.81: 0.19 for major: minor. Displacement ellipsoids set at 50 % and hydrogen from phosphine ligand and [BAr$^{F}{}_{4}$]$^{-}$ anion removed for clarity in all parts.

Selected bond lengths (Å): Rh1-P1 2.1965(7), Rh1-P2 2.1959(7), Rh1-C1 2.430(4), Rh1-C4 2.788(6), Rh1-H4AB 1.9196(4), C1-C2 1.504(7), C3-C4 1.475(8).

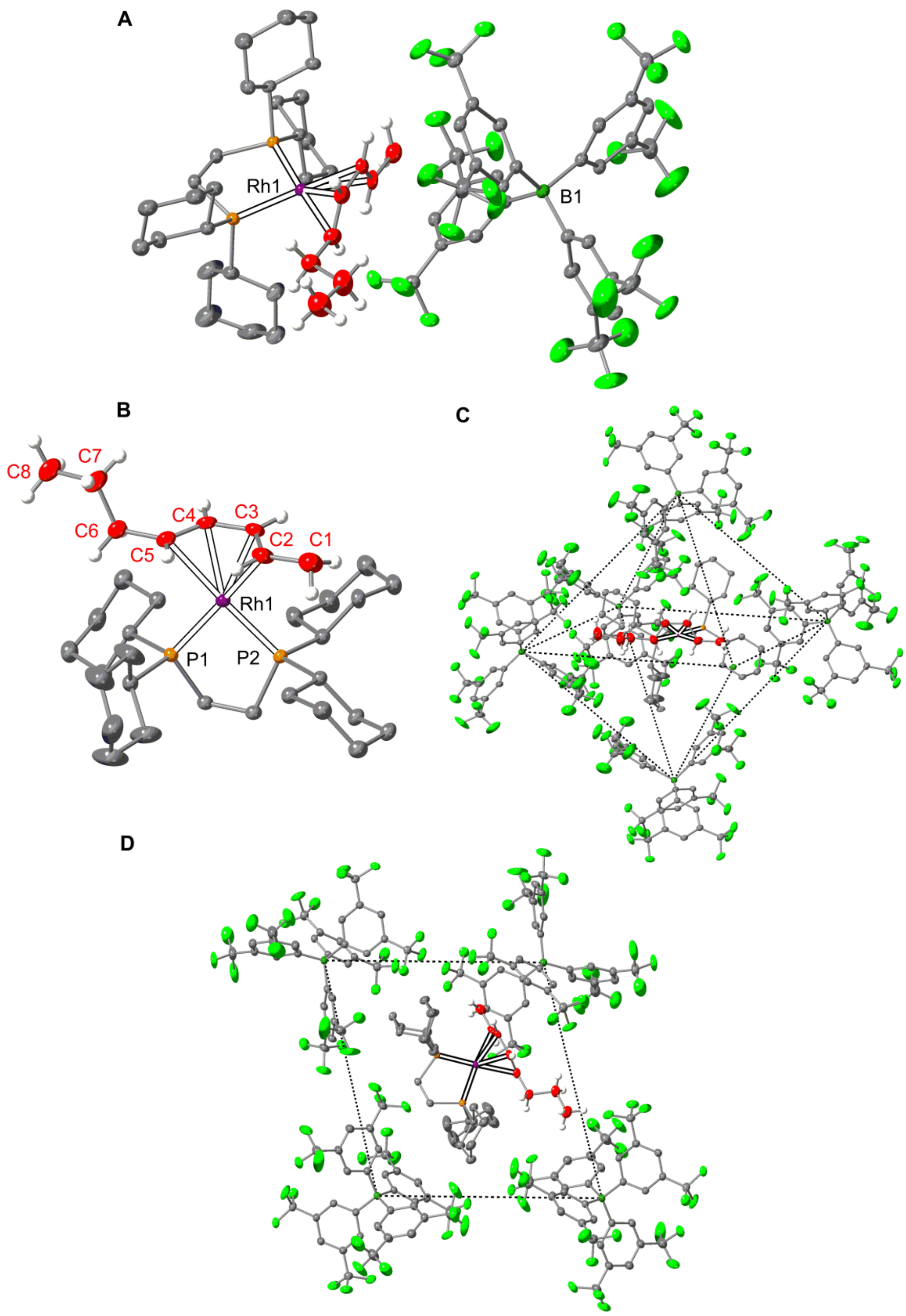

Figure S57: (A) Molecular structure of **[1-octadiene][BAr^F$_4$]**. **(B)** Cationic fragment of **[1-octadiene][BAr^F$_4$]** showing atom labelling. **(C)** Structure displaying pseudo-O_h anion network of the [BAr^F$_4$]$^-$ ions, with cationic fragments sat within the cavity; –(Ar^F$_4$) removed from 1 boron atom for clarity. **(D)** Top view of pseudo-O_h anion-cage, showing twisted cation orientation. Displacement ellipsoids set at 50 % and hydrogen from phosphine ligand and [BAr^F$_4$]$^-$ anion removed for clarity in all parts.

Selected bond lengths (Å): Rh1-P1 2.2874(6), Rh1-P2 2.2627(6), Rh1-C2 2.259(3), Rh1-C3 2.175(3), Rh1-C4 2.201(3), Rh1-C5 2.359(3), C2-C3 1.363(5), C4-C5 1.340(5).

S.3.5. Comparison of Rh···C distances from selected σ-alkane complexes

Table S2: Comparison of Rh···C distances in [Rh(L_2)(alkane)][anion], L_2 = chelating phosphine.

Complex	Rh···C	Hapacity	Ref
[Rh($^iBu_2PCH_2CH_2P^iBu_2$)(NBA)][BAr^F_4]	2.494(10), 2.480(11)	1,2–η^2,η^2	S20
[Rh($Cy_2PCH_2CH_2PCy_2$)(NBA)][BAr^F_4]	2.389(3), 2.400(3)	1,2–η^2,η^2	S2
[Rh($Cy_2PCH_2CH_2PCy_2$)(pentane)][BAr^F_4]	2.514(4), 2.522(5)	2,4–η^2,η^2	S1
[Rh($Cyp_2PCH_2CH_2PCyp_2$)(NBA)][BAr^F_4]	2.388(5), 2.392(5)	1,2–η^2,η^2	S21
[Rh($Cy_2PCH_2CH_2PCy_2$)(NBA)][BAr^{Cl}_4]	2.387(5), 2.378(5)	1,2–η^2,η^2	S22
[Rh($Cy_2PCH_2CH_2CH_2PCy_2$)(NBA)][BAr^F_4]	2.408(2), 2.402(2)	1,2–η^2,η^2	S23
[Rh($Cy_2PCH_2CH_2CH_2CH_2PCy_2$)(NBA)][$BAr^F_4$]	2.399(2), 2.396(2)	1,2–η^2,η^2	
[RhH($Cy_2PCH_2CHCH_2CH_2PCy_2$)(NBA)][BAr^F_4]	2.90(3)	η^1	
[Rh($Cy_2PCH_2CH_2PCy_2$)(cyclohexane)][BAr^F_4]	2.62(2), 2.53(2)	Between 1,3-η^2,η^2 and 1,3 –η^1,η^1	S7
[Rh($Cy_2PCH_2CH_2PCy_2$)(isobutane)][BAr^F_4]	2.362(14), 2.443(7)	1,2–η^2,η^2	
[Rh($Cy_2PCH_2CH_2PCy_2$)(propane)][BAr^F_4]	2.46(2), 2.46(2)	1,3–η^2,η^2	This work
[Rh($Cy_2PCH_2CH_2PCy_2$)(hexane)][BAr^F_4]	2.527(3), 2.549(4)	2,4–η^2,η^2	
[Rh($Cy_2PCH_2CH_2PCy_2$)(2-methylbutane)][BAr^F_4]	2.348(9), 2.39(1)	1,2–η^2,η^2	
[Rh($Cy_2PCH_2CH_2PCy_2$)(3-methylpentane)][BAr^F_4]	2.430(4), 2.788(6)	1,4–η^2,η^1	

Note: The complexes presented in Table S2 are formed with a pseudo-O_h arrangement of [BAr^F_4]$^-$ anions, with the exception of [Rh($Cy_2PCH_2CH_2PCy_2$)(pentane)][BAr^F_4] and [Rh($Cy_2PCH_2CH_2PCy_2$)(hexane)][BAr^F_4] which have a bi-capped square pyramidal arrangement, and [Rh($Cy_2PCH_2CH_2PCy_2$)(NBA)][BAr^{Cl}_4] where eight [BAr^{Cl}_4]$^-$ anions form a gyrobifastigium arrangement made of two, face-sharing, parallelepipeds.

S.4. Computational Methods

S.4.1. Solid State Calculations

All static Kohn-Sham DFT calculations were performed on periodic models of the studied rhodium complexes, employing the Gaussian Plane Wave (GPW) formalism as implemented in the QUICKSTEP[S24] module within the CP2K program suite (Version 5.0).[S25] Molecularly optimised basis sets of double-ζ quality plus polarization in their short-range variant (DZVP-MOLOPT-SR-GTH)[S26] were used on all atomic species. The interaction between the core electrons and the valence shell (Rh: 17, B: 3, C: 4, P: 5, F: 7, H: 1 electrons) was described by Goedecker-Teter-Hutter (GTH) pseudo potentials.[S27-29] The generalized gradient approximation (GGA) to the exchange-correlation functional according to Perdew-Burke-Ernzerhof (PBE)[S30] was used in combination with Grimme's D3-correction for dispersion interactions.[S31] The auxiliary plane wave basis set was truncated at a cutoff of 500 Ry. The maximum force convergence criterion was set to 10^{-4} Eh·Bohr^{-1}, whilst default values were used for the remaining criteria. The convergence criterion for the self-consistent field (SCF) accuracy was set to 10^{-7} Eh and 10^{-8} Eh for geometry optimizations and vibrational analysis, respectively.

The Brillouin zone was sampled using the Γ-point. Initial coordinates were obtained from the experimental crystallographic data, with the hydrogen positions normalised where possible with Mercury.[S32] Periodic boundary conditions (PBC) were applied throughout in combination with fixed unit cell parameters obtained from experiment. All geometries were first partially relaxed, keeping the heavy atoms (non-H, F) fixed, then fully relaxed without imposing any constraints, whilst keeping unit cell parameters constant in all cases.

All optimised stationary points were further characterized by analysis of their numerical second derivatives with a displacement of 0.01 Bohr and were found to have no imaginary eigenvalues.

Gibbs free energies for structures computed in the solid state were calculated using the TAMkin software toolkit.[S33] Numerical instability when recomputing the wavefunction of the alternative rotamer of **1-(3-methylpentane)][BAr$^F_4$]** prevented us from defining a free energy for this species and so ΔSCF energies are quoted in Sections 2.1.5 and 2.1.7 for **[1-(2-methylbutane)][BAr$^F_4$]** and **1-(3-methylpentane)][BAr$^F_4$]** respectively. In the former case, a free energy difference of +10.3 kcal/mol is computed, 1.5 kcal/mol higher than the ΔSCF energy. Based on this the ΔSCF value of 2.5 kcal/mol quoted for **1-(3-methylpentane)][BAr$^F_4$]** is likely to be underestimated by a similar amount.

The CIFs of the fully optimised systems, generated using VESTA[S34], were analysed using the Crystal Explorer package[S35], using a central cation and the six nearest neighbour anions.

The cation with its six neighbouring anions were generated with the following method:

- Open the CIF in CrystalExplorer.
- Select the cation of interest, Select Display -> Invert Selection and then Remove -> Selected Atoms.
- Select Actions - > Generate Atoms Within Radius.
- Enter an appropriate radius, (around 11 Å from the central cation in our case).
- Select the surrounding anions and the central cation and then select Remove -> “Incomplete Fragments”.
- Select all remaining atoms and then Actions-> “Complete Fragments”.
- Select the central cation then Actions -> “Generate Surface” to generate the Hirshfeld Surface.
- Modify the charges and multiplicities of all the fragments as required.

All computed structures are available as a separate file of Cartesian coordinates.

S.4.2. Molecular Calculations

Molecular calculations employed the GAUSSIAN 09 (revision D.01) program package[S36] and employed the BP86 GGA functional.[S37-38] Stuttgart-Dresden (SDD)[S39] relativistic effective core potentials (ECP) in combination with the associated basis sets were utilized to describe Rh and P, with polarization functions added for P (ζ = 0.387).[S40] The 6-31G(d,p) basis sets[S41-42] were used on remaining atoms.

Electronic structure analyses were performed on the geometries of the Rh cations extracted from the CP2K-optimised structures with the heavy atoms fixed at the experimental positions. An electron density file suitable for further analysis was generated from a single-point calculation.

The topology of the electron density was analysed by means of QTAIM (Quantum Theory of Atoms in Molecules),[S43] as implemented in the AIMALL package.[S44] Inner shell electrons on Rh and P modelled by ECPs were represented by core density functions (extended wavefunction format). All bond critical bond (BCPs) are shown in green, ring critical points (RCPs) in pink. Contours plots are in the plane containing Rh and the two H atoms involved in the σ-interactions.

NBO calculations were performed using the NBO 6.0 program[S45], using the same geometries as for the QTAIM calculations above. Donor-acceptor interactions are presented

in the main text as a sum of all contributions to a given type of pairing (e.g. all $Rh_{LP} \rightarrow \sigma *_{C–H}$ donations above the threshold of 0.05 kcal/mol). All individual donor-acceptor pairs are depicted for **[1-propane]⁺** (see Figures S68 to S75) and these are illustrative of the donor-acceptors interactions in the other systems. The latter are tabulated with additional figures presented when specifically referred to in the main text.

NCI calculations were performed using the NCIPLOT program,[S44-47] using the nearest neighbour ion-pair molecular structures extracted from CP2K optimised geometries. The promolecular electron density was employed and isosurfaces are generated for s = 0.3 au and $-0.07 \leq \rho \leq 0.07$ au.

Orbital plots were created with Chemcraft[S48] with an outer contour value of 0.07465.

Data from the partially optimised (with non-H and F atoms fixed at their crystallographically determined positions) and the fully optimised structures (used in the final analyses) are presented below side by side for comparison.

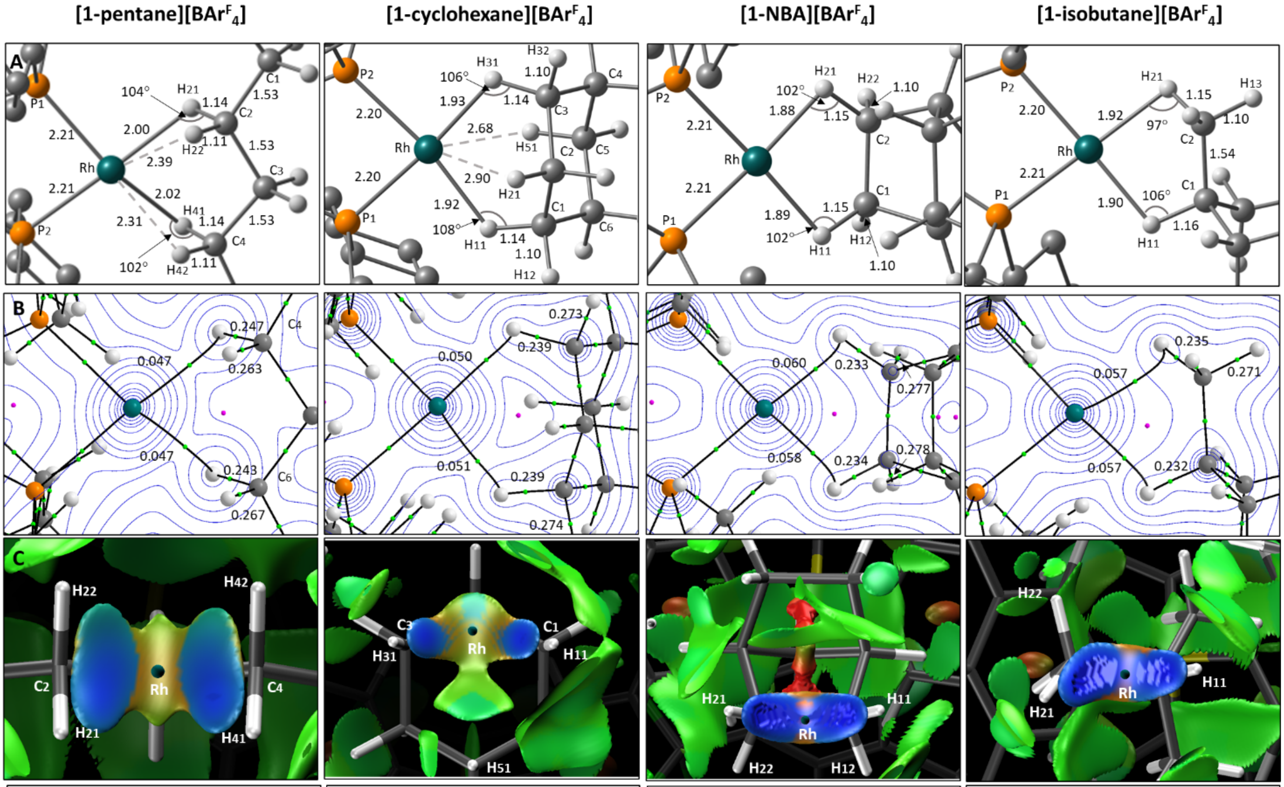

D

[1-pentane][BAr$^F_4$]

σ-donation

$\sigma_{C_2-H_{21}} \rightarrow \sigma^*_{Rh-P_2}$ = 12.0
$\sigma_{C_2-H_{22}} \rightarrow \sigma^*_{Rh-P_2}$ = 4.1
$\sigma_{C_4-H_{41}} \rightarrow \sigma^*_{Rh-P_2}$ = 13.3
$\sigma_{C_4-H_{42}} \rightarrow \sigma^*_{Rh-P_2}$ = 2.6

back-donation

$Rh_{LP} \rightarrow \sigma^*_{C_2-H_{21}}$ = 5.4
$\sigma_{Rh-P} \rightarrow \sigma^*_{C_2-H_{21}}$ = 3.0
$Rh_{LP} \rightarrow \sigma^*_{C_2-H_{22}}$ = 1.7
$\sigma_{Rh-P} \rightarrow \sigma^*_{C_2-H_{22}}$ = 1.0
$Rh_{LP} \rightarrow \sigma^*_{C_4-H_{41}}$ = 5.2
$\sigma_{Rh-P} \rightarrow \sigma^*_{C_4-H_{41}}$ = 3.3
$Rh_{LP} \rightarrow \sigma^*_{C_4-H_{42}}$ = 1.3

[1-cyclohexane][BAr$^F_4$]

σ-donation[a]

$\sigma_{C_1-H_{11}} \rightarrow \sigma^*_{Rh-P_2}$ = 16.5
$\sigma_{C_3-H_{31}} \rightarrow \sigma^*_{Rh-P_1}$ = 15.8

back-donation

$Rh_{LP} \rightarrow \sigma^*_{C_1-H_{11}}$ = 2.6
$\sigma_{Rh-P} \rightarrow \sigma^*_{C_1-H_{11}}$ = 7.8
$Rh_{LP} \rightarrow \sigma^*_{C_3-H_{31}}$ = 3.1
$\sigma_{Rh-P} \rightarrow \sigma^*_{C_1-H_{11}}$ = 7.2

[1-NBA][BAr$^F_4$]

σ-donation

$\sigma_{C_1-H_{11}} \rightarrow \sigma^*_{Rh-P_2}$ = 21.8
$\sigma_{C_2-H_{21}} \rightarrow \sigma^*_{Rh-P_1}$ = 20.5

back-donation

$Rh_{LP} \rightarrow \sigma^*_{C_1-H_{11}}$ = 4.7
$\sigma_{Rh-P} \rightarrow \sigma^*_{C_1-H_{11}}$ = 4.5
$Rh_{LP} \rightarrow \sigma^*_{C_2-H_{21}}$ = 4.3
$\sigma_{Rh-P} \rightarrow \sigma^*_{C_2-H_{21}}$ = 4.7

[1-isobutane][BAr$^F_4$]

σ-donation

$\sigma_{C_1-H_{11}} \rightarrow \sigma^*_{Rh-P_2}$ = 19.8
$\sigma_{C_2-H_{21}} \rightarrow \sigma^*_{Rh-P_1}$ = 22.6

back-donation

$Rh_{LP} \rightarrow \sigma^*_{C_1-H_{11}}$ = 3.3
$\sigma_{Rh-P} \rightarrow \sigma^*_{C_1-H_{11}}$ = 5.4
$Rh_{LP} \rightarrow \sigma^*_{C_2-H_{21}}$ = 5.4
$\sigma_{Rh-P} \rightarrow \sigma^*_{C_2-H_{21}}$ = 3.0

Figure S58: (A) Computed structure of the **[1–alkane]$^+$** cations not presented in the main paper highlighting key distances (Å) and angles to H atoms. Non-alkane H atoms are omitted for clarity **(B)** QTAIM molecular graphs **(C)** Detail of the NCI plots viewed from the Rh center looking down an axis passing through the center of the alkane moiety interacting with Rh. **(D)** Major donor–acceptor interactions derived from a 2nd order perturbation NBO analysis (kcal/mol) Values are the sum of each type of donor-acceptor interactions (see tables below for full details).

S.5. Electronic Structure Analysis

S.5.1. [1-propane][BAr^F_4]

[1-propane][BAr^F_4] (Partially Optimised)

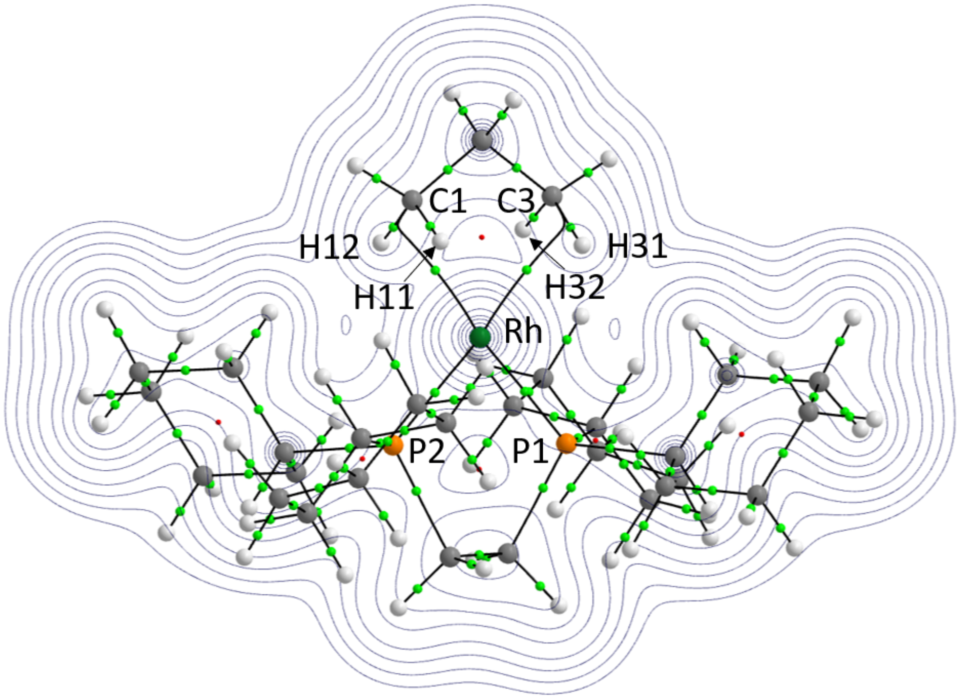

Figure S59: QTAIM molecular graph of **[1-propane][BAr^F_4]** (partially optimised) with electron density contours in the H12–Rh–H31 plane.

Table S3: QTAIM BCP data and bond lengths for key bonds.

Bond Path	Interatomic Distance (Å)	ρ (e bohr^{-3})	$\nabla^2\rho$ (e bohr^{-5})	H(r)
C1 –H12	1.128	0.2505	-0.7428	-0.2339
H12–Rh	2.074		**No BCP**	
C1–Rh	2.451	0.0447	0.1851	-0.0015
Rh–P2	2.206	0.1191	0.0727	-0.0591
C3 –H31	1.126	0.2518	-0.7541	-0.2364
C3–Rh	2.462	0.0436	0.1798	-0.0013
H31–Rh	2.089		**No BCP**	
Rh–P1	2.227	0.1151	0.0792	-0.0546

[1-propane][BAr$^F_4$] (Fully Optimised)

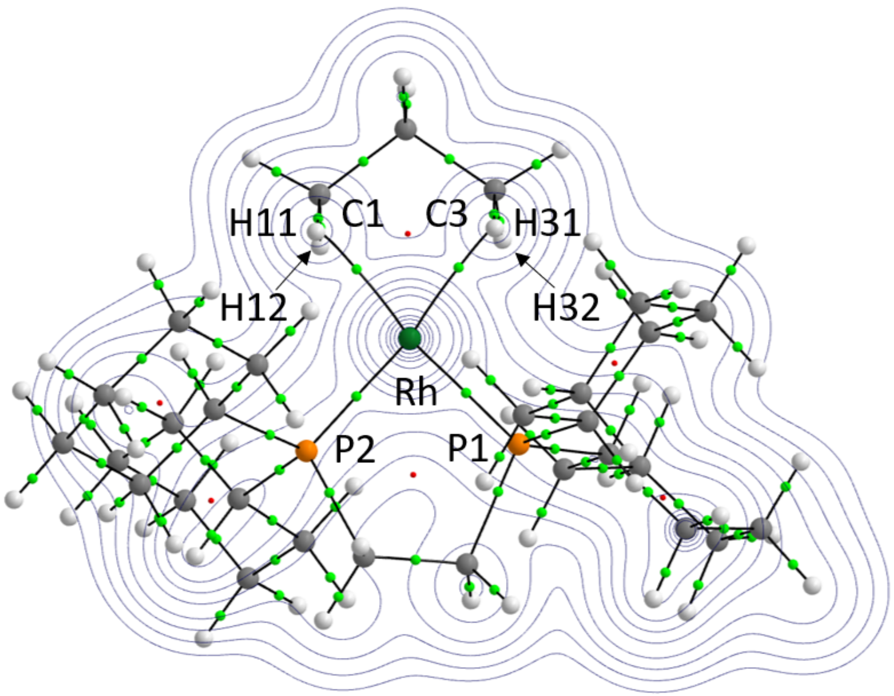

Figure S60: QTAIM molecular graph of **[1-propane][BAr$^F_4$]** (fully optimised) with electron density contours in the H11–Rh–H31 plane.

Table S4: QTAIM BCP data and bond lengths for key bonds.

Bond Path	Interatomic Distance (Å)	ρ (e bohr^{-3})	∇^{2}ρ (e bohr^{-5})	H(r)
C1 –H11	1.134	0.2463	-0.7123	-0.2262
H11–Rh	2.027	0.0457	0.1764	-0.0039
C1–Rh	2.504		**No BCP**	
Rh–P2	2.207	0.1193	0.0706	-0.0594
C3 –H31	1.136	0.2449	-0.7026	-0.2241
H31–Rh	2.001	0.0476	0.1799	-0.0049
C3–Rh	2.506		**No BCP**	
Rh–P1	2.205	0.1198	0.0688	-0.0598

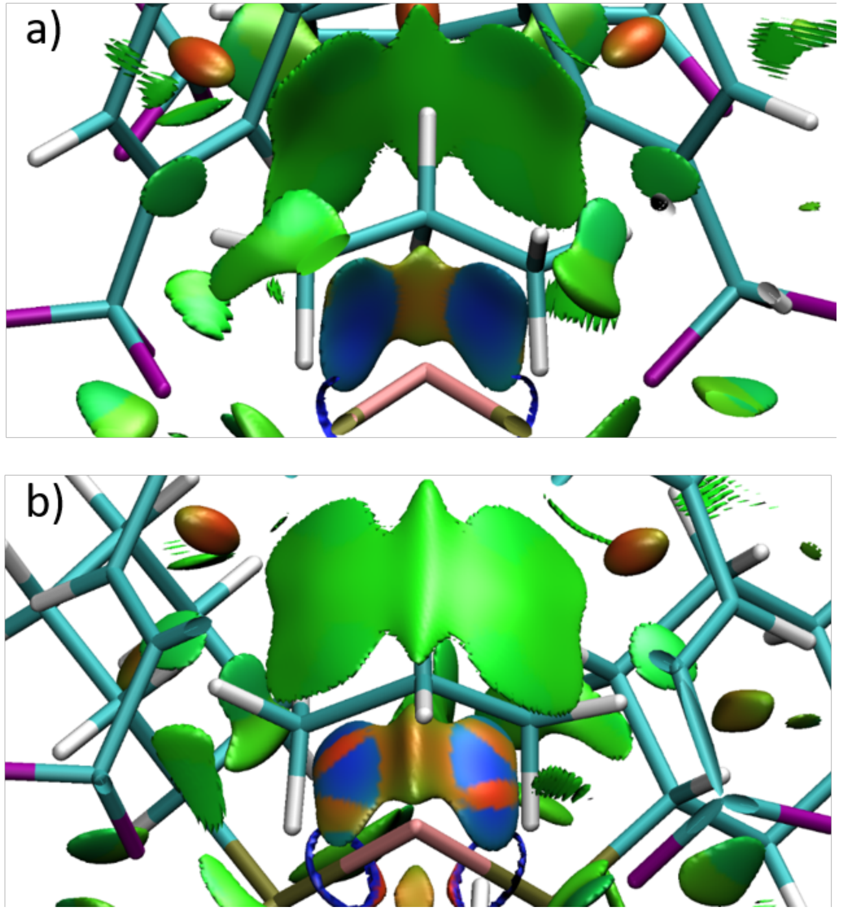

Figure S61: a) NCI Plot of **[1-propane][BAr$^F{}_4$]** (partially optimised) with isosurfaces generated for s = 0.3 au and -0.07 < ρ < 0.07 au. View from Rh looking towards the alkane. **b)** View from above the alkane looking towards Rh.

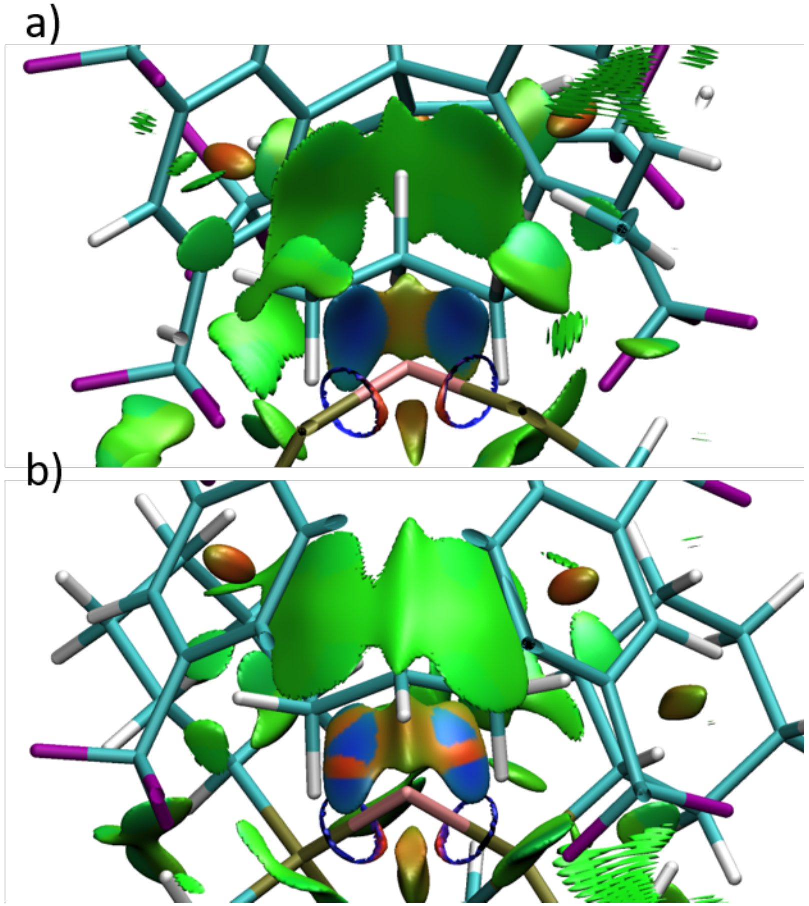

Figure S62: a) NCI Plot of **[1-propane][BAr$^F{}_4$]** (fully optimised) with isosurfaces generated for s = 0.3 au and -0.07 < ρ < 0.07 au. View from Rh looking towards the alkane. **b)** View from above the alkane looking towards Rh.

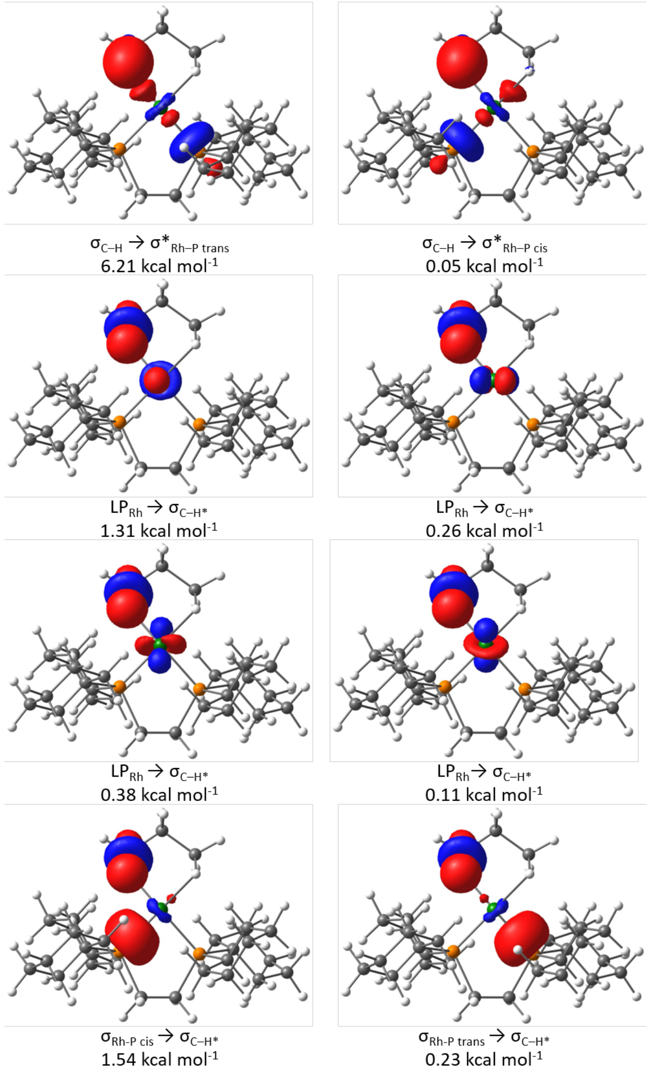

Figure S63: NBO donor acceptor orbital pairs of C3–H31 in **[1-propane][BAr^F$_4$]** (partially optimised) with their associated 2nd order perturbation theory energies.

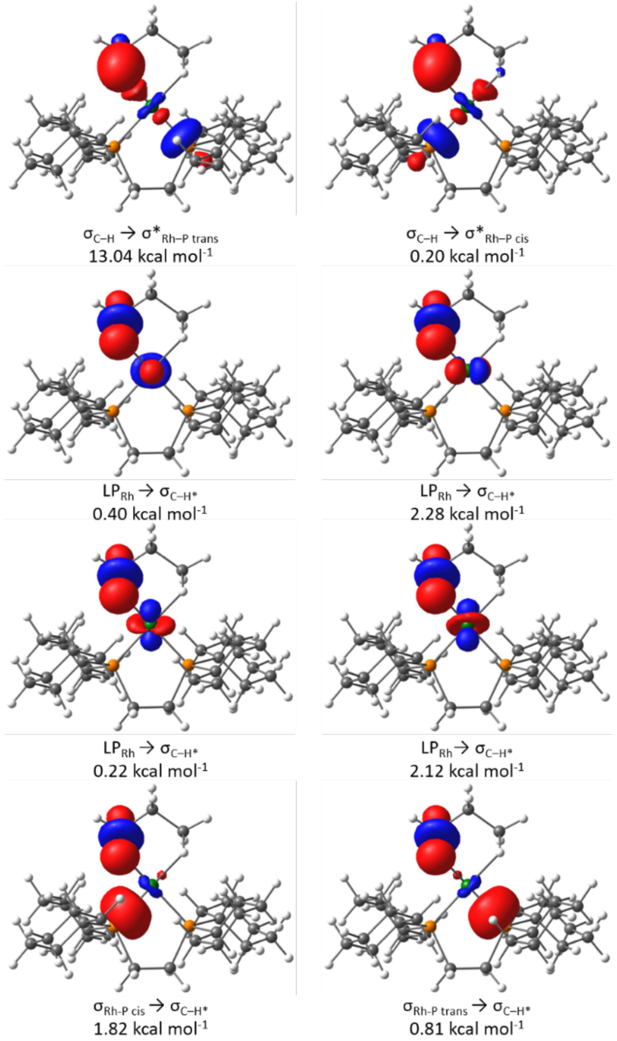

Figure S64: NBO donor acceptor orbital pairs of C3–H31 in **[1-propane][BAr^F$_4$]** (fully optimised) with their associated 2nd order perturbation theory energies.

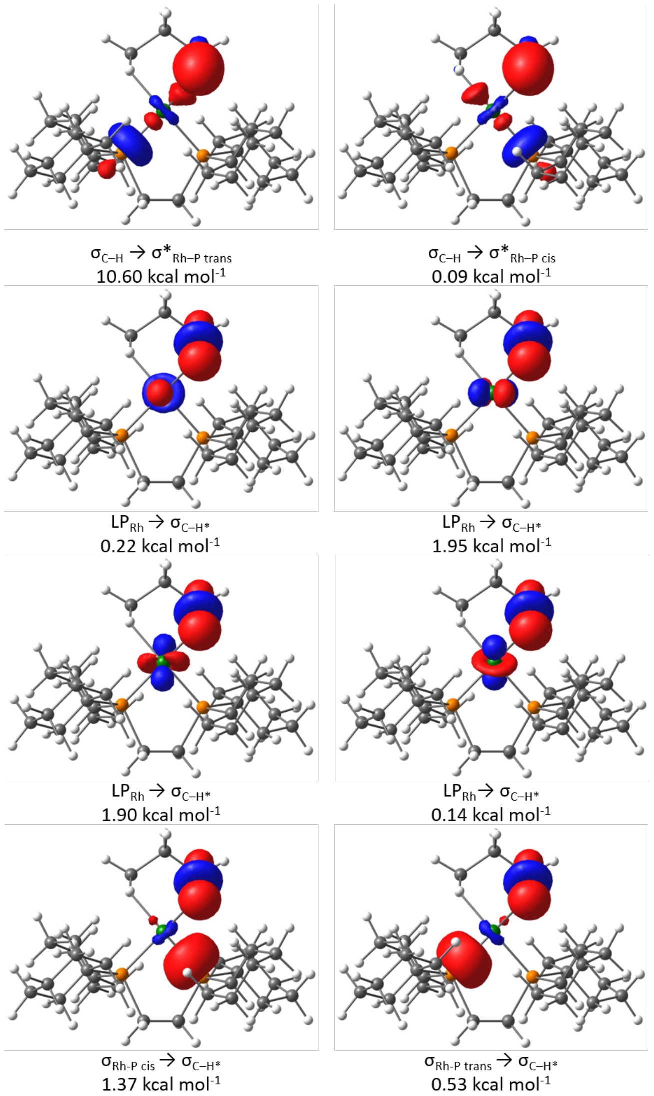

Figure S65: NBO donor acceptor orbital pairs of C1–H11 in **[1-propane][BAr$^F_4$]** (partially optimised) with their associated 2nd order perturbation theory energies

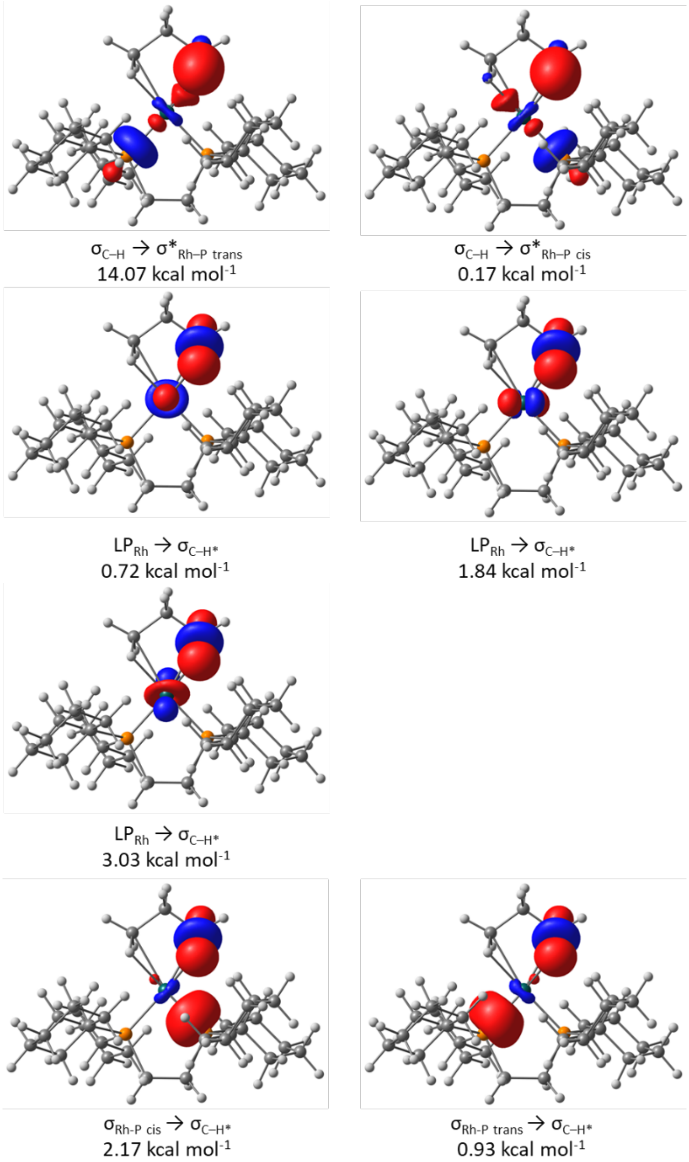

Figure S66: NBO donor acceptor orbital pairs of C1–H11 in **[1-propane][BAr$^F_4$]** (fully optimised) with their associated 2nd order perturbation theory energies

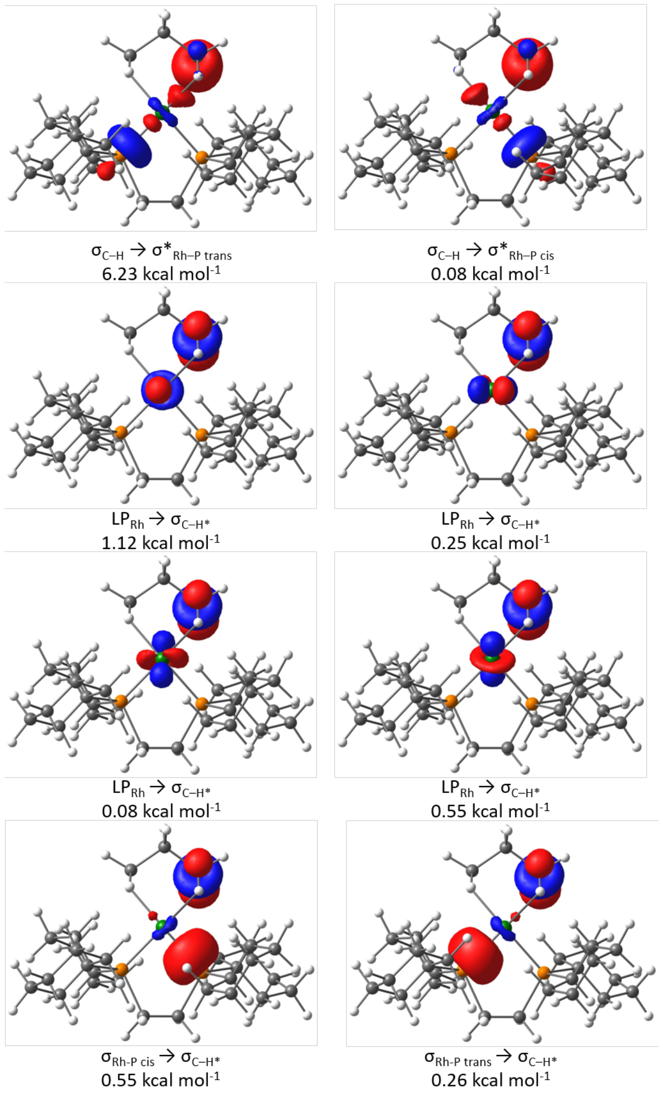

Figure S67: NBO donor acceptor orbital pairs of C1–H12 in **[1-propane][BAr^F$_4$]** (partially optimised) with their associated 2nd order perturbation theory energies.

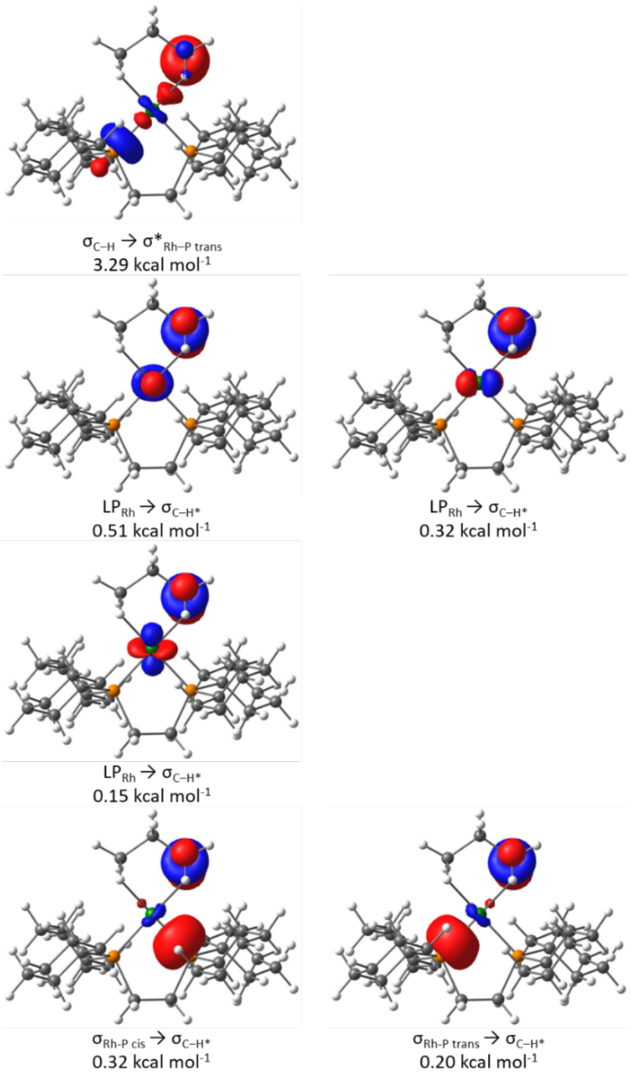

Figure S68: NBO donor acceptor orbital pairs of C1–H12 in **[1-propane][BAr^F$_4$]** (fully optimised) with their associated 2nd order perturbation theory energies.

.

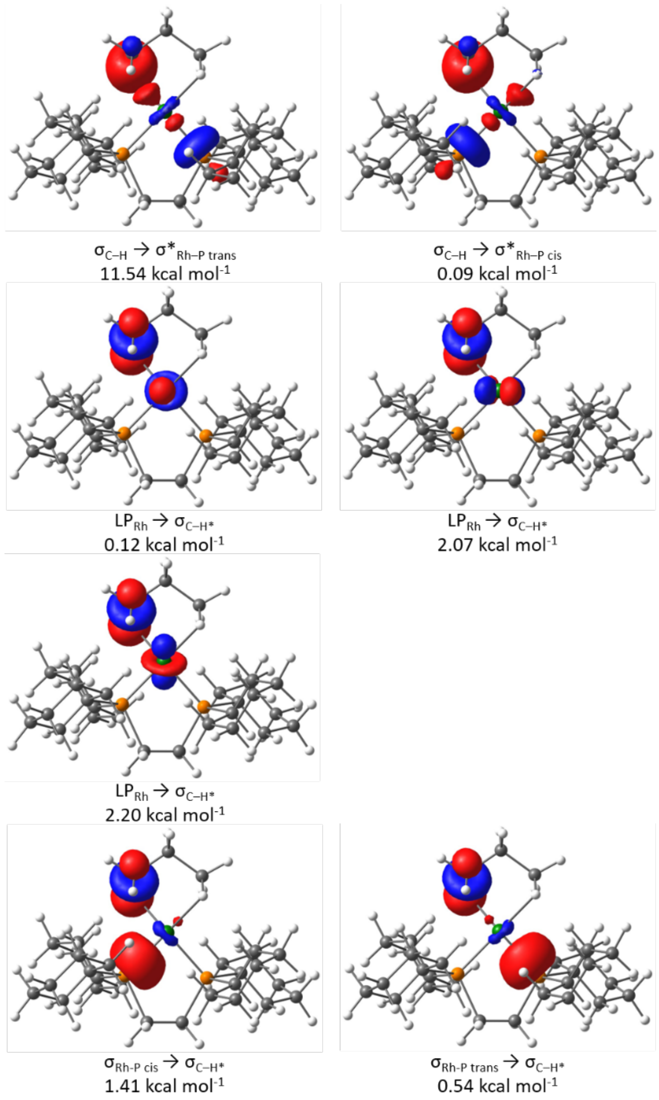

Figure S69: NBO donor acceptor orbital pairs of C3–H32 in **[1-propane][BAr$^F_4$]** (partially optimised) with their associated 2nd order perturbation theory energies.

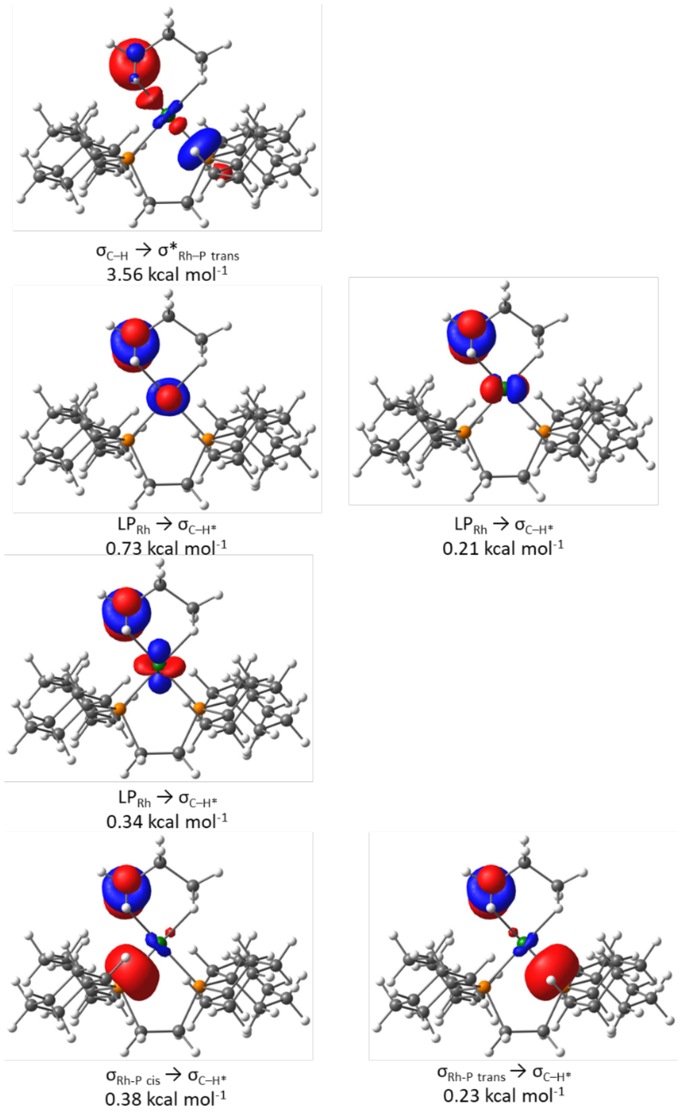

Figure S70: NBO donor acceptor orbital pairs of C3–H32 in **[1-propane][BAr$^F_4$]** (fully optimised) with their associated 2nd order perturbation theory energies.

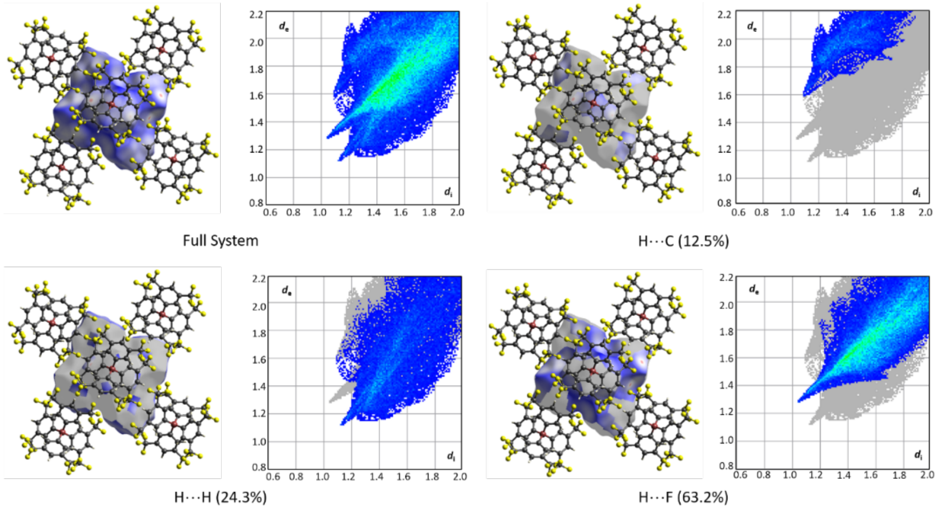

Figure S71: Hirshfeld Surfaces showing normalised distance and associated fingerprint plots in **[1-propane][BAr$^{F}_{4}$]** (partially optimised). Percentage of each type of contact is shown in brackets.

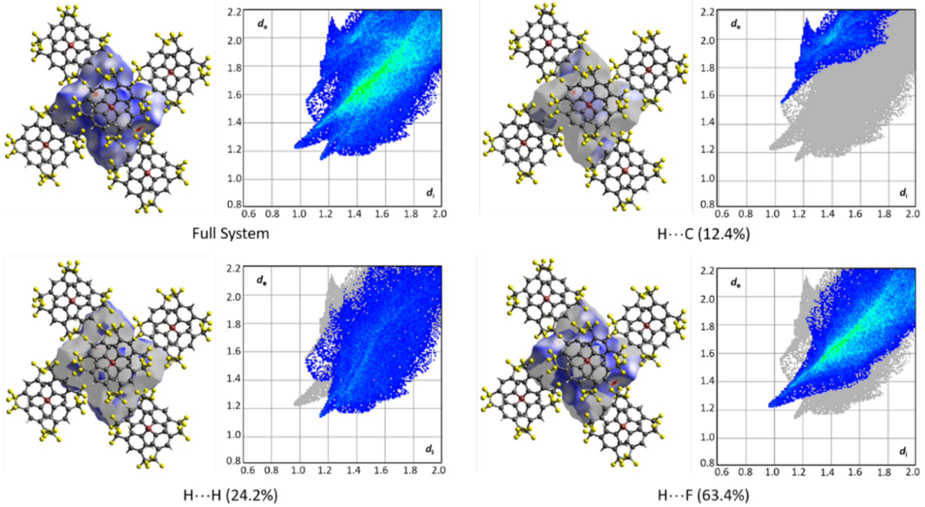

Figure S72: Hirshfeld Surfaces showing normalised distance and associated fingerprint plots in **[1-propane][BAr$^{F}_{4}$]** (fully optimised). Percentage of each type of contact is shown in brackets.

S.5.2. [1-pentane][BAr$^F_4$]

Note: The structure of [Rh($Cy_2PCH_2CH_2PCy_2$)(C_5H_{12})][BAr$^F_4$], **[1-pentane][BAr$^F_4$]**, has been previous reported.[S1] We now report results using the protocol adopted in this paper for consistency.

[1-pentane][BAr$^F_4$] (Partially Optimised)

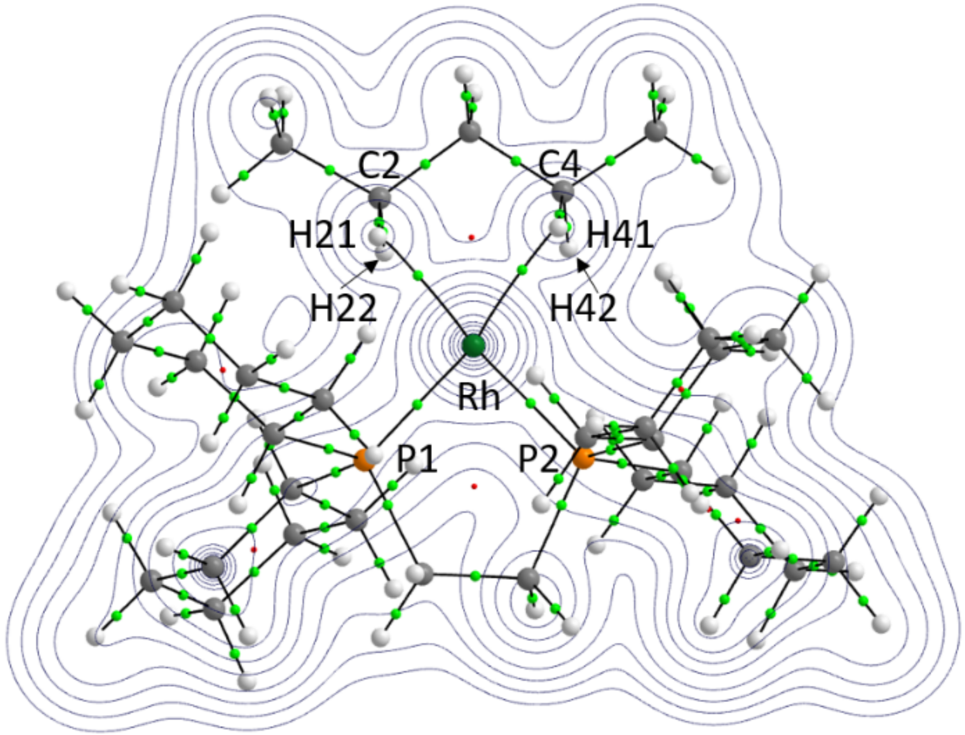

Figure S73: QTAIM molecular graph of **[1-pentane][BAr$^F_4$]** (partially optimised) with electron density contours in the H21–Rh–H41 plane.

Table S5: QTAIM BCP data and bond lengths for key bonds.

Bond Path	Interatomic Distance (Å)	ρ (e bohr^{-3})	∇^{2}ρ (e bohr^{-5})	H(r)
C2 –H21	1.140	0.2488	-0.6912	-0.2212
H21–Rh	1.998	0.0473	0.1770	-0.0052
C2–Rh	2.522		**No BCP**	
Rh–P1	2.197	0.1212	0.0668	-0.1388
C4 –H41	1.136	0.2472	-0.7156	-0.2299
H41–Rh	2.029	0.0458	0.1709	-0.0043
C4–Rh	2.513		**No BCP**	
Rh–P2	2.196	0.1216	0.0665	-0.1392

[1-pentane][BAr$^F{}_4$] (Fully Optimised)

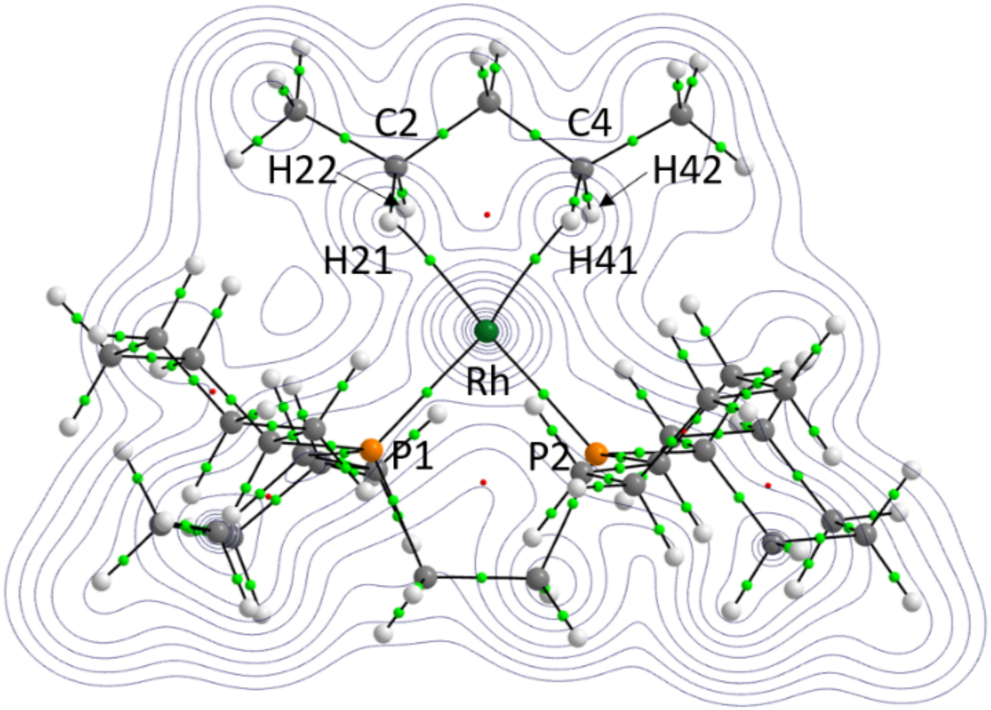

Figure S74: QTAIM molecular graph of **[1-pentane][BAr$^F{}_4$]** (fully optimised) with electron density contours in the H21–Rh–H41 plane.

Table S6: QTAIM BCP data and bond lengths for key bonds.

Bond Path	Interatomic Distance (Å)	ρ (e bohr^{-3})	∇^{2}ρ (e bohr^{-5})	H(r)
C2 –H21	1.141	0.2428	-0.6843	-0.2195
H21–Rh	1.998	0.0472	0.1742	-0.0054
C2–Rh	2.534		**No BCP**	
Rh–P1	2.207	0.1191	0.0722	-0.0591
C4–H41	1.137	0.2465	-0.7103	-0.2256
H41–Rh	2.020	0.0468	0.1721	-0.0048
C4–Rh	2.509		**No BCP**	
Rh–P2	2.207	0.1189	0.0731	-0.0588

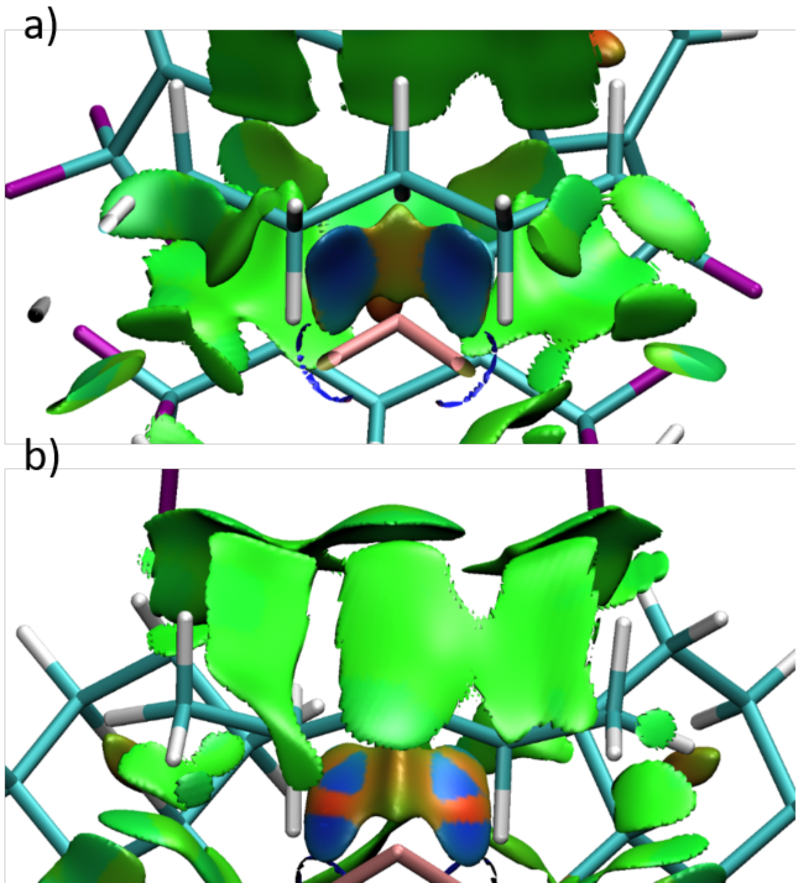

Figure S75: a) NCI Plot of **[1-pentane][BAr$^F{}_4$]** (partially optimised) with isosurfaces generated for s = 0.3 au and -0.07 < ρ < 0.07 au. View from Rh looking towards the alkane. **b)** View from above the alkane looking towards Rh.

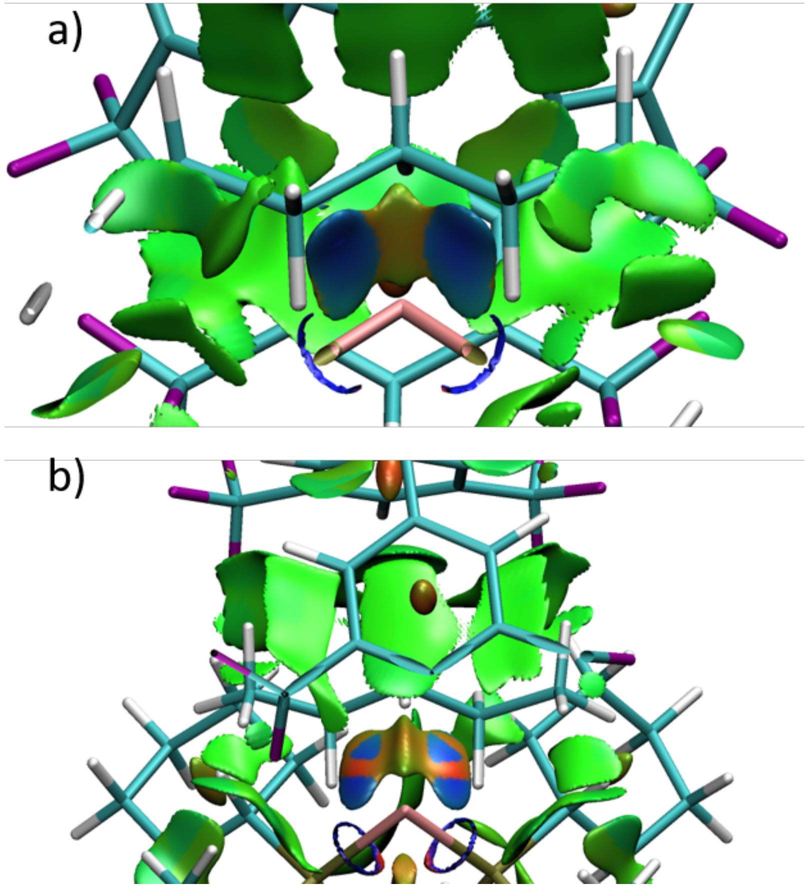

Figure S76: a) NCI Plot of **[1-pentane][BAr$^F{}_4$]** (fully optimised) with isosurfaces generated for s = 0.3 au and -0.07 < ρ < 0.07 au. View from Rh looking towards the alkane. **b)** View from above the alkane looking towards Rh.

Table S7: NBO data from **[1-pentane][BAr$^F_4$]** (partially optimised) showing the donating and accepting orbitals occupation and the 2nd order perturbation theory interaction energy.

**Donating Orbital**	**Occupation**	**Accepting Orbital**	**Occupation**	**Energy (kcal/mol)**
		C2–H21		
σ (C–H)	1.92	σ* (Rh–P_{trans})	0.25	11.63
σ (C–H)	1.92	σ* (Rh–P_{Cis})	0.26	0.14
LP (Rh)	1.96	σ* (C–H)	0.04	0.98
LP (Rh)	1.94	σ* (C–H)	0.04	1.66
LP (Rh)	1.89	σ* (C–H)	0.04	2.52
σ (Rh–P_{cis})	1.83	σ* (C–H)	0.04	1.96
σ (Rh–P_{trans})	1.83	σ* (C–H)	0.04	1.00
		C2–H22		
σ (C–H)	1.96	σ* (Rh–P_{trans})	0.25	3.61
LP (Rh)	1.96	σ* (C–H)	0.25	0.58
LP (Rh)	1.94	σ* (C–H)	0.25	0.55
LP (Rh)	1.90	σ* (C–H)	0.25	0.26
LP (Rh)	1.89	σ* (C–H)	0.25	0.14
σ (Rh–P_{cis})	1.83	σ* (C–H)	0.25	0.54
σ (Rh–P_{trans})	1.83	σ* (C–H)	0.25	0.33
		C4–H41		
σ (C–H)	1.91	σ* (Rh–P_{trans})	0.26	13.06
σ (C–H)	1.91	σ* (Rh–P_{Cis})	0.25	0.06
LP (Rh)	1.96	σ* (C–H)	0.04	0.17
LP (Rh)	1.94	σ* (C–H)	0.04	2.21
LP (Rh)	1.89	σ* (C–H)	0.04	2.86
σ (Rh–P_{cis})	1.83	σ* (C–H)	0.04	2.27
σ (Rh–P_{trans})	1.83	σ* (C–H)	0.04	0.98
		C4–H42		
σ (C–H)	1.96	σ* (Rh–P_{trans})	0.26	2.57
LP (Rh)	1.96	σ* (C–H)	0.22	0.84
LP (Rh)	1.94	σ* (C–H)	0.22	0.17
LP (Rh)	1.90	σ* (C–H)	0.22	0.15
LP (Rh)	1.89	σ* (C–H)	0.22	0.09
σ (Rh–P_{cis})	1.83	σ* (C–H)	0.22	0.45
σ (Rh–P_{trans})	1.83	σ* (C–H)	0.22	0.30

Table S8: NBO data from **[1-pentane][BAr$^F_4$]** (fully optimised) showing the donating and accepting orbitals occupation and the 2nd order perturbation theory interaction energy.

Donating Orbital	Occupation	Accepting Orbital	Occupation	Energy (kcal/mol)
		C2–H21		
σ (C–H)	1.92	σ* (Rh–P_{trans})	0.26	12.01
σ (C–H)	1.92	σ* (Rh–P_{Cis})	0.25	0.17
LP (Rh)	1.96	σ* (C–H)	0.04	1.12
LP (Rh)	1.94	σ* (C–H)	0.04	1.56
LP (Rh)	1.90	σ* (C–H)	0.04	0.12
LP (Rh)	1.89	σ* (C–H)	0.04	2.58
σ (Rh–P_{cis})	1.83	σ* (C–H)	0.04	1.96
σ (Rh–P_{trans})	1.83	σ* (C–H)	0.04	1.02
		C2–H22		
σ (C–H)	1.95	σ* (Rh–P_{trans})	0.25	4.07
LP (Rh)	1.96	σ* (C–H)	0.02	0.61
LP (Rh)	1.94	σ* (C–H)	0.02	0.64
LP (Rh)	1.90	σ* (C–H)	0.02	0.35
LP (Rh)	1.89	σ* (C–H)	0.02	0.11
σ (Rh–P_{cis})	1.83	σ* (C–H)	0.02	0.61
σ (Rh–P_{trans})	1.83	σ* (C–H)	0.02	0.36
		C4–H41		
σ (C–H)	1.91	σ* (Rh–P_{trans})	0.26	13.25
σ (C–H)	1.91	σ* (Rh–P_{Cis})	0.25	0.07
LP (Rh)	1.96	σ* (C–H)	0.04	0.12
LP (Rh)	1.94	σ* (C–H)	0.04	2.28
LP (Rh)	1.89	σ* (C–H)	0.04	2.75
σ (Rh–P_{cis})	1.83	σ* (C–H)	0.04	2.28
σ (Rh–P_{trans})	1.83	σ* (C–H)	0.04	1.01
		C4–H42		
σ (C–H)	1.96	σ* (Rh–P_{trans})	0.26	2.57
LP (Rh)	1.96	σ* (C–H)	0.02	0.89
LP (Rh)	1.94	σ* (C–H)	0.02	0.15
LP (Rh)	1.90	σ* (C–H)	0.02	0.18
LP (Rh)	1.89	σ* (C–H)	0.02	0.06
σ (Rh–P_{cis})	1.83	σ* (C–H)	0.02	0.47
σ (Rh–P_{trans})	1.83	σ* (C–H)	0.02	0.32

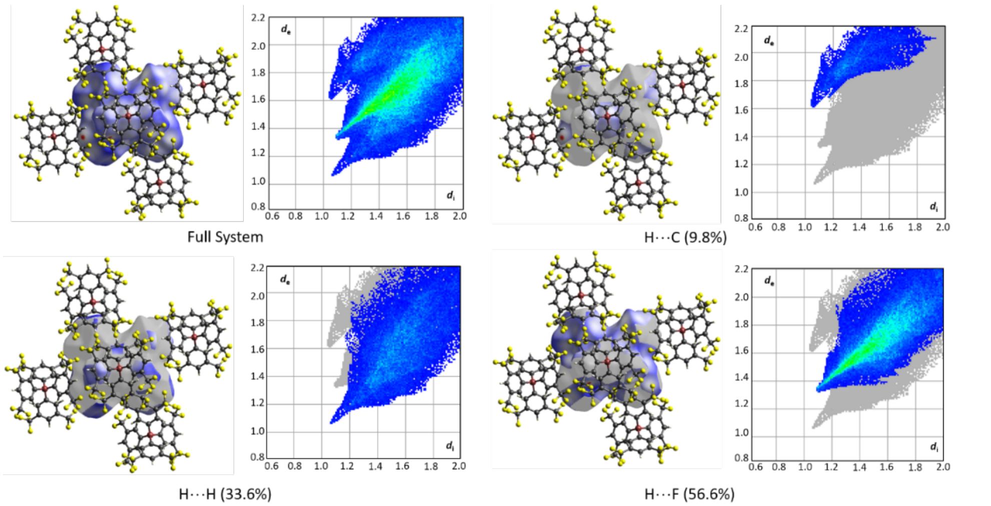

Figure S77: Hirshfeld Surfaces of **[1-pentane][BAr$^{F}_{4}$]** (partially optimised) showing normalised distance and associated fingerprint plots. Percentage of each type of contact is shown in brackets.

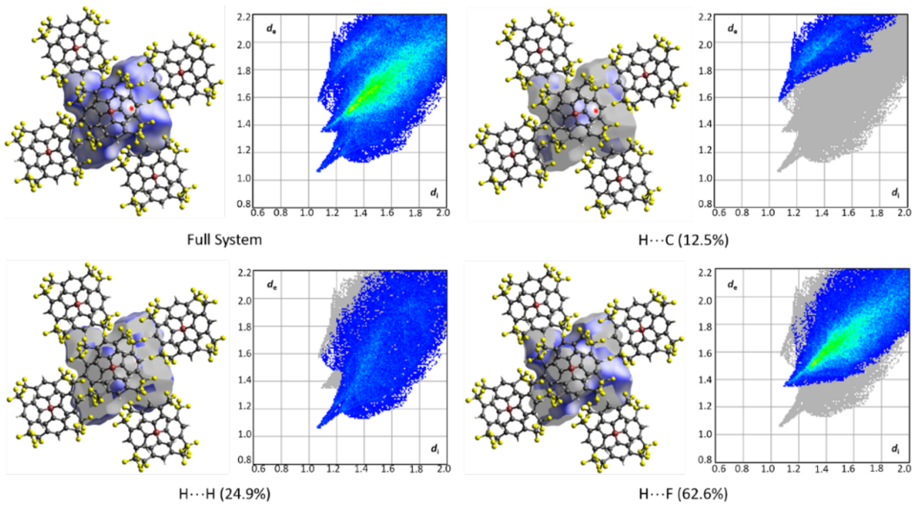

Figure S78: Hirshfeld Surfaces of **[1-pentane][BAr$^{F}_{4}$]** (fully optimised) showing normalised distance and associated fingerprint plots. Percentage of each type of contact is shown in brackets.

S.5.3. [1-(2-methylbutane)][BAr$^F{}_4$]

[1-(2-methylbutane)][BAr$^F{}_4$] (Partially Optimised)

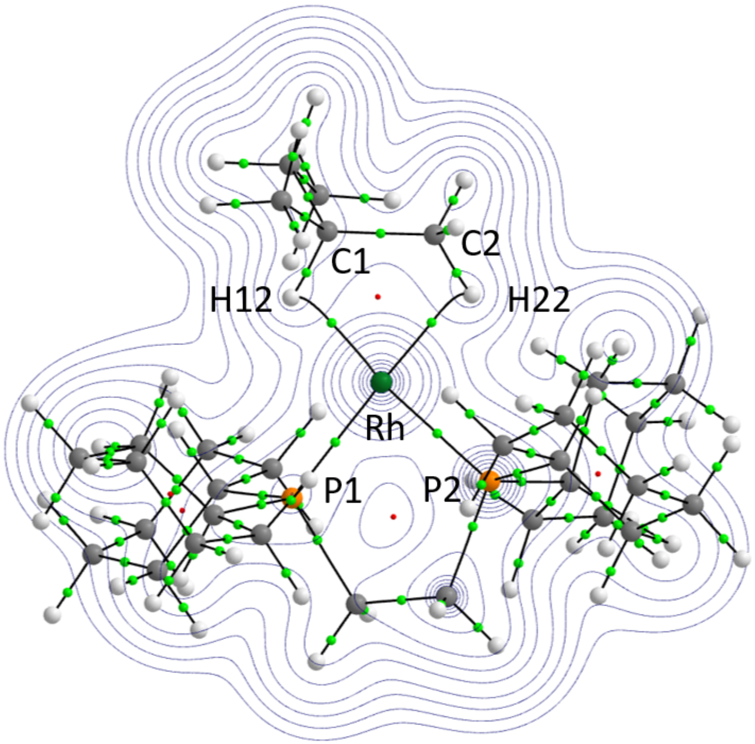

Figure S79: QTAIM molecular graph of **[1-(2-methylbutane)][BAr$^F{}_4$]** (partially optimised) with electron density contours in the H12–Rh–H22 plane.

Table S9: QTAIM BCP data and bond lengths for key bonds.

Bond Path	Interatomic Distance (Å)	ρ (e bohr^{-3})	$\nabla^2\rho$ (e bohr^{-5})	H(r)
C1 –H12	1.160	0.2312	-0.5990	-0.1992
H12–Rh	1.846	0.0646	0.2634	-0.0100
C1–Rh	2.390		**No BCP**	
Rh–P1	2.187	0.1237	0.0666	-0.0643
C2 –H22	1.145	0.2385	-0.6560	-0.2133
H22–Rh	1.878	0.0622	0.2580	-0.0085
C2–Rh	2.349		**No BCP**	
Rh–P2	2.185	0.1237	0.0699	-0.0643

[1-(2-methylbutane)][BAr$^F{}_4$] (Fully Optimised)

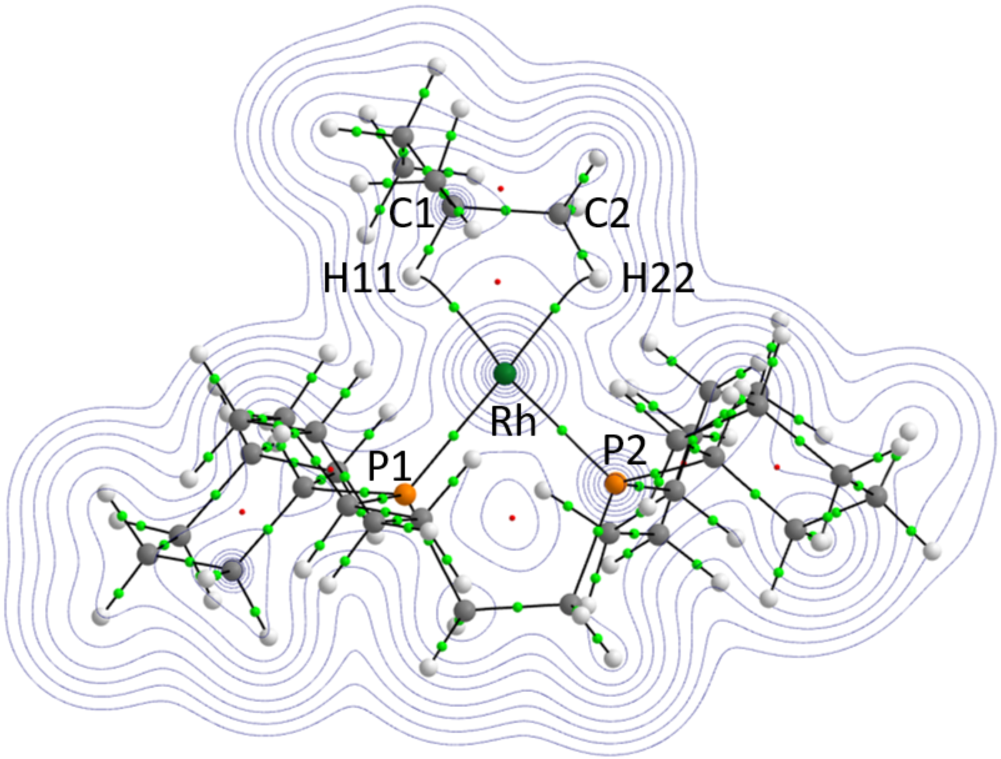

Figure S80: QTAIM molecular graph of **[1-(2-methylbutane)][BAr$^F{}_4$]** (fully optimised) with electron density contours in the H11–Rh–H22 plane.

Table S10: QTAIM BCP data and bond lengths for key bonds.

Bond Path	Interatomic Distance (Å)	ρ (e bohr^{-3})	∇²ρ (e bohr^{-5})	H(r)
C1 –H11	1.161	0.2304	-0.5978	-0.1979
H11–Rh	1.899	0.0568	0.2245	-0.0077
C1–Rh	2.475		**No BCP**	
Rh–P1	2.211	0.1182	0.0782	-0.0579
C2 –H22	1.150	0.2352	-0.6331	-0.2073
H22–Rh	1.909	0.0579	0.2413	-0.0068
C2–Rh	2.381		**No BCP**	
Rh–P2	2.200	0.1207	0.0708	-0.0608

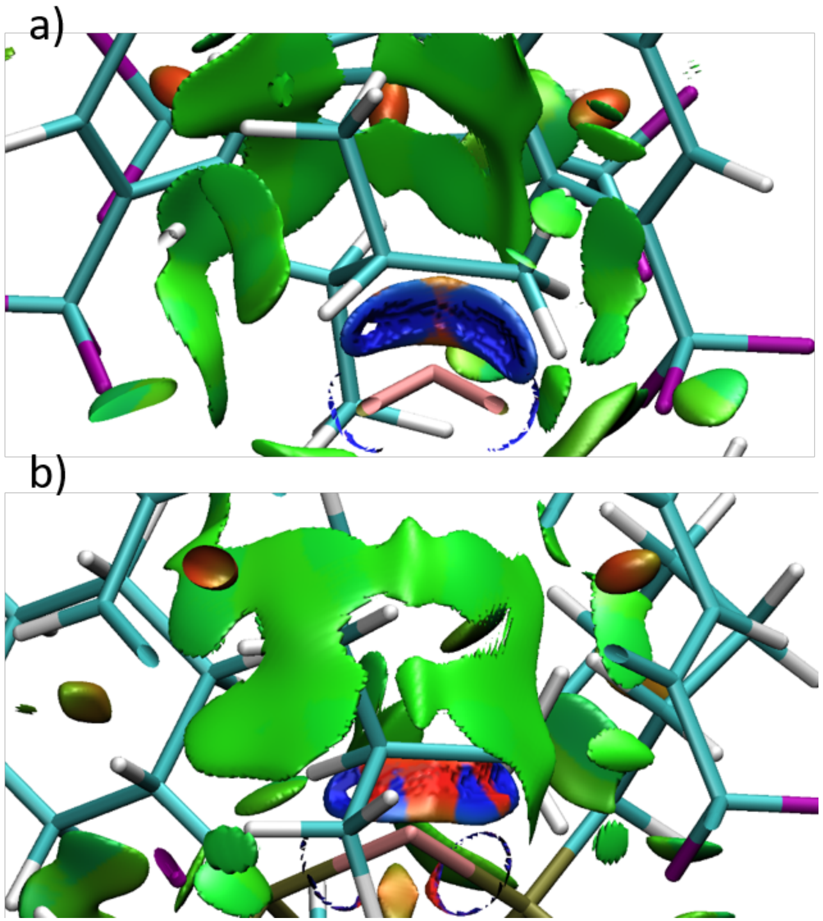

Figure S81: a) NCI Plot of **[1-(2-methylbutane)][BAr^F$_4$]** (partially optimised) with isosurfaces generated for s = 0.3 au and -0.07 < ρ < 0.07 au. View from Rh looking towards the alkane. **b)** View from above the alkane looking towards Rh.

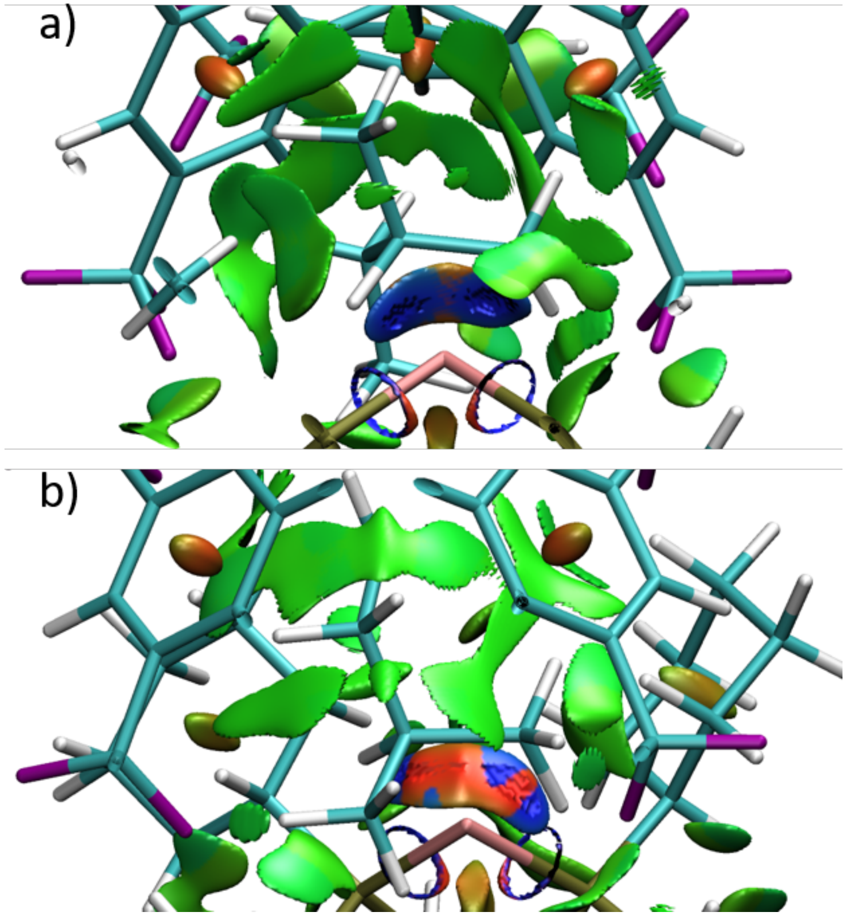

Figure S82: a) NCI Plot of **[1-(2-methylbutane)][BAr^F$_4$]** (fully optimised) with isosurfaces generated for s = 0.3 au and -0.07 < ρ < 0.07 au. View from Rh looking towards the alkane. **b)** View from above the alkane looking towards Rh.

Table S11: NBO data from **[1-(2-methylbutane)][BAr^F$_4$]** (partially optimised) showing the donating and accepting orbitals occupation and the 2nd order perturbation theory interaction energy.

Donating Orbital	Occupation	Accepting Orbital	Occupation	Energy (kcal/mol)
		C1–H11		
σ (C–H)	1.86	σ* (Rh-P_{trans})	0.29	22.71
σ (C–H)	1.86	σ* (Rh–P_{CIS})	0.28	0.30
LP (Rh)	1.97	σ* (C–H)	0.05	0.07
LP (Rh)	1.95	σ* (C–H)	0.05	0.18
LP (Rh)	1.87	σ* (C–H)	0.05	3.95
σ (Rh–P_{cis})	1.83	σ* (C–H)	0.05	4.14
σ (Rh–P_{trans})	1.83	σ* (C–H)	0.05	1.50
		C2–H22		
σ (C–H)	1.88	σ* (Rh–P_{trans})	0.28	24.74
σ (C–H)	1.88	σ* (Rh–P_{CIS})	0.29	0.28
LP (Rh)	1.95	σ* (C–H)	0.03	1.07
LP (Rh)	1.91	σ* (C–H)	0.03	0.24
LP (Rh)	1.87	σ* (C–H)	0.03	4.88
σ (Rh–P_{cis})	1.83	σ* (C–H)	0.03	3.34
σ (Rh-P_{trans})	1.83	σ* (C–H)	0.03	0.76

Table S12: NBO data from **[1-(2-methylbutane)][BAr^F$_4$]** (fully optimised) showing the donating and accepting orbitals occupation and the 2nd order perturbation theory interaction energy.

Donating Orbital	Occupation	Accepting Orbital	Occupation	Energy (kcal/mol)
		C1–H11		
σ (C–H)	1.86	σ* (Rh-P_{trans})	0.28	19.80
σ (C–H)	1.86	σ* (Rh–P_{Cis})	0.27	0.25
LP (Rh)	1.97	σ* (C–H)	0.05	0.09
LP (Rh)	1.95	σ* (C–H)	0.05	0.12
LP (Rh)	1.91	σ* (C–H)	0.05	0.44
LP (Rh)	1.88	σ* (C–H)	0.05	2.65
σ (Rh–P_{cis})	1.83	σ* (C–H)	0.05	3.67
σ (Rh-P_{trans})	1.83	σ* (C–H)	0.05	1.79
		C2–H22		
σ (C–H)	1.88	σ* (Rh-P_{trans})	0.27	21.93
σ (C–H)	1.88	σ* (Rh–P_{CIS})	0.28	0.24
LP (Rh)	1.95	σ* (C–H)	0.03	1.22
LP (Rh)	1.91	σ* (C–H)	0.03	1.01
LP (Rh)	1.88	σ* (C–H)	0.03	3.42
σ (Rh–P_{cis})	1.83	σ* (C–H)	0.03	2.45
σ (Rh-P_{trans})	1.83	σ* (C–H)	0.03	0.79

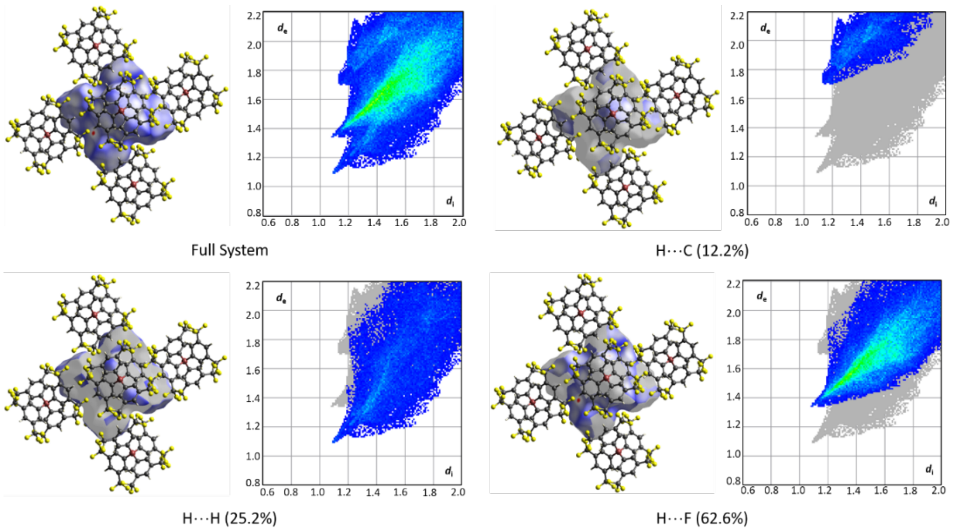

Figure S83: Hirshfeld Surfaces of **[1-(2-methylbutane)][BAr$^F_4$]** (partially optimised) showing normalised distance and associated fingerprint plots. Percentage of each type of contact is shown in brackets.

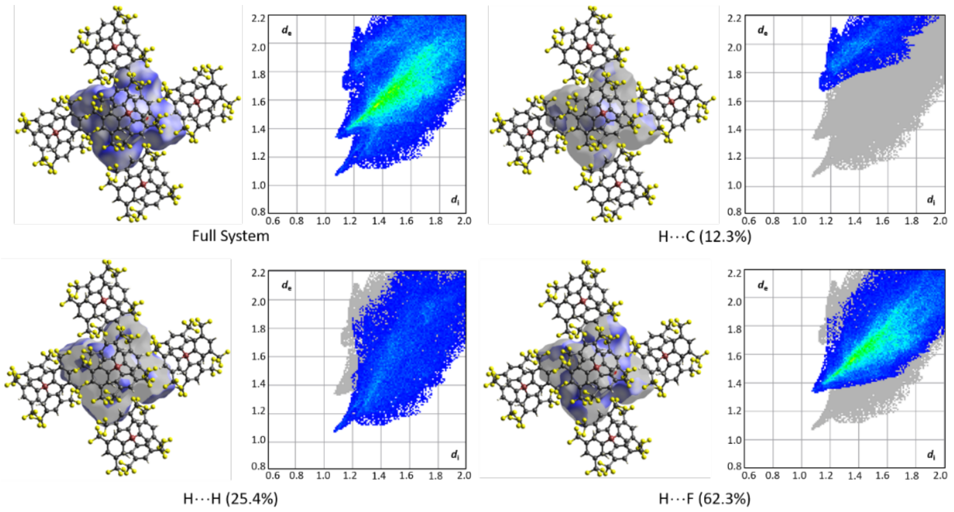

Figure S84: Hirshfeld Surfaces of **[1-(2-methylbutane)][BAr$^F_4$]** (fully optimised) showing normalised distance and associated fingerprint plots. Percentage of each type of contact is shown in brackets.

S.5.4. [1-hexane][BAr$^F_4$]

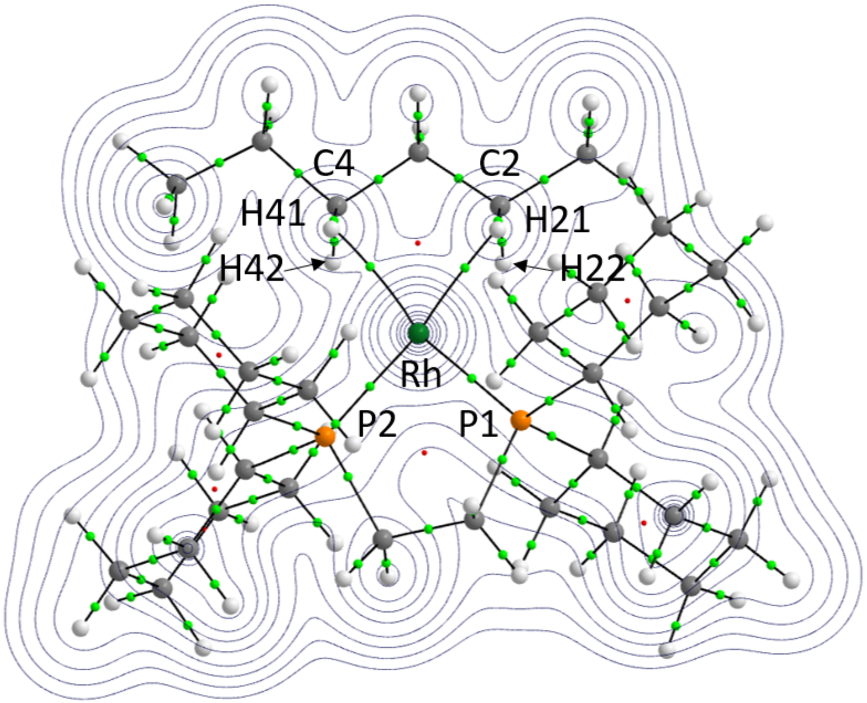

Figure S85: QTAIM molecular graph of **[1-hexane][BAr$^F_4$]** (partially optimised) with electron density contours in the H41–Rh–H21 plane.

Table S13: QTAIM BCP data and bond lengths for key bonds.

Bond Path	Interatomic Distance (Å)	ρ (e bohr^{-3})	$\nabla^2\rho$ (e bohr^{-5})	H(r)
C1 –H1	1.131	0.2489	-0.7298	-0.2304
H1–Rh	2.065	0.0418	0.1590	-0.0031
C1–Rh	2.550		**No BCP**	
Rh–P1	2.200	0.1204	0.0722	-0.0605
C2 –H2	1.142	0.2421	-0.6781	-0.2185
H2–Rh	1.981	0.0488	0.1794	-0.0060
C2–Rh	2.527		**No BCP**	
Rh–P2	2.191	0.1225	0.0617	-0.0631

[1-hexane][BAr^F$_4$] (Fully Optimised)

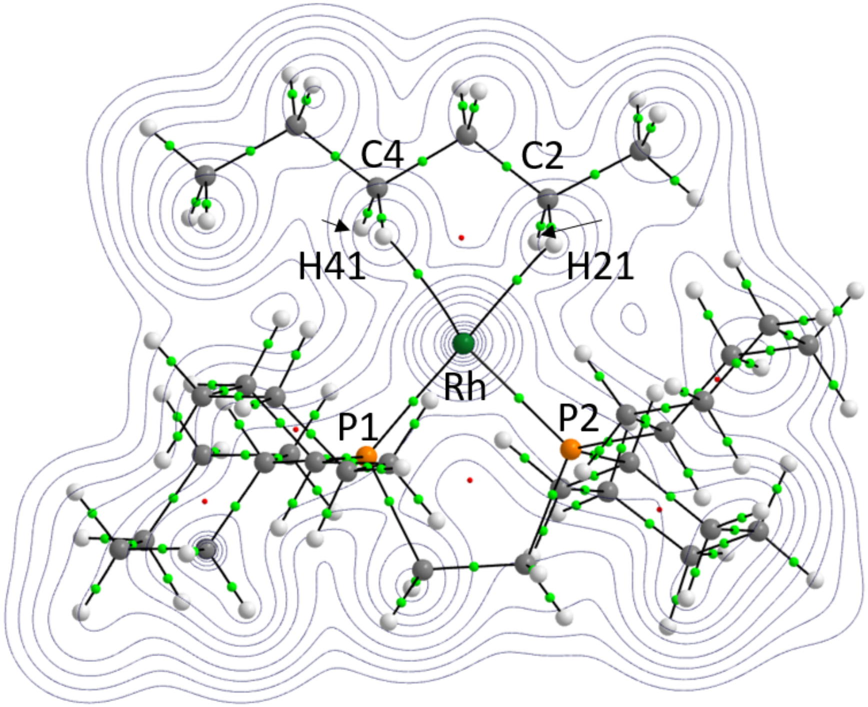

Figure S86: QTAIM molecular graph of **[1-hexane][BAr^F$_4$]** (fully optimised) with electron density contours in the H41–Rh–H21 plane.

Table S14: QTAIM BCP data and bond lengths for key bonds.

Bond Path	Interatomic Distance (Å)	ρ (e bohr^{-3})	∇²ρ (e bohr^{-5})	H(r)
C1 –H1	1.139	0.2430	-0.6881	-0.2203
H1–Rh	2.032	0.0430	0.1505	-0.0048
C1–Rh	2.621		**No BCP**	
Rh–P1	2.217	0.1168	0.0793	-0.0565
C2 –H2	1.143	0.2411	-0.6725	-0.2168
H2–Rh	1.973	0.0491	0.1774	-0.0065
C2–Rh	2.542		**No BCP**	
Rh–P2	2.205	0.1195	0.0674	-0.0595

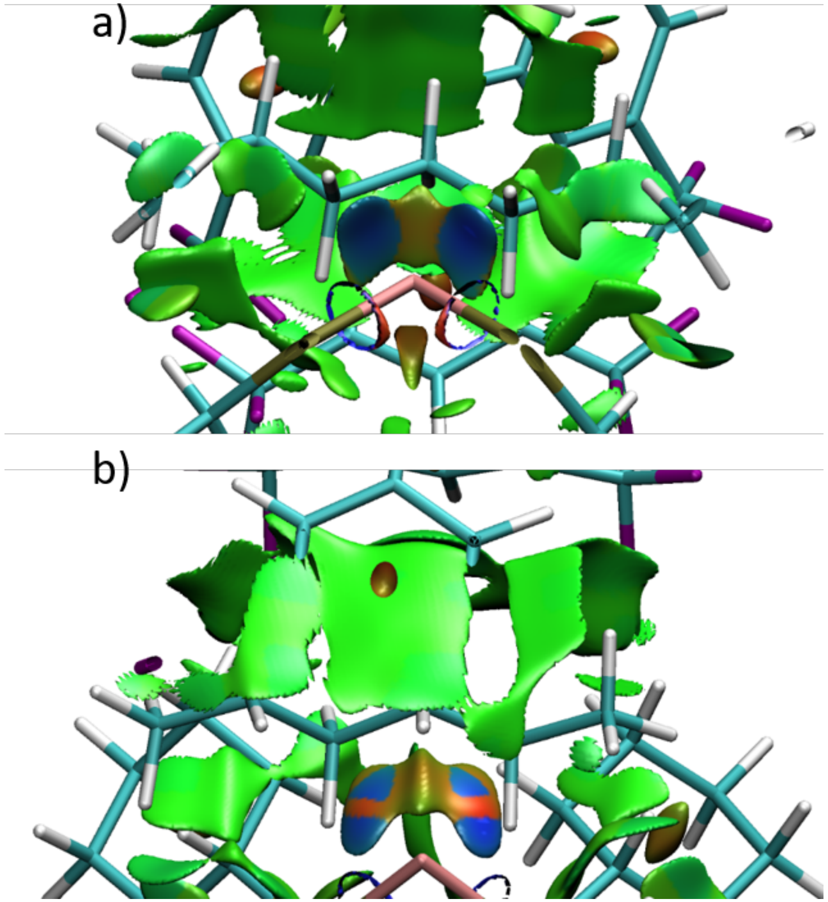

Figure S87: a) NCI Plot of **[1-hexane][BAr$^F{}_4$]** (partially optimised) with isosurfaces generated for s = 0.3 au and -0.07 < ρ < 0.07 au. View from Rh looking towards the alkane. **b)** View from above the alkane looking towards Rh.

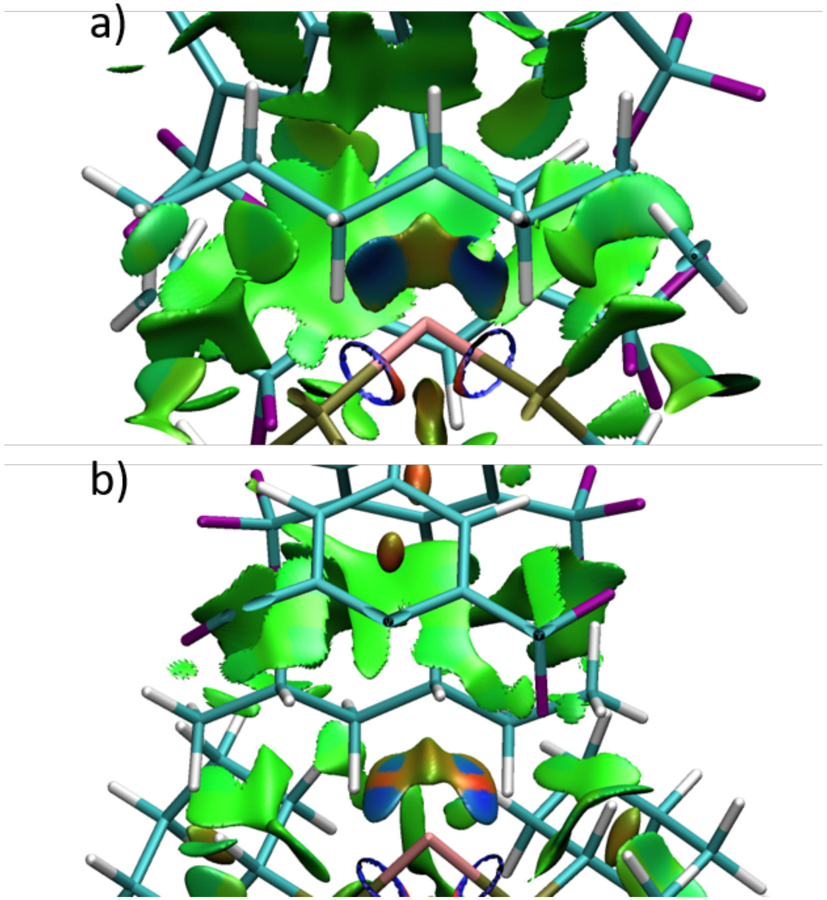

Figure S88: a) NCI Plot of **[1-hexane][BAr$^F{}_4$]** (fully optimised) with isosurfaces generated for s = 0.3 au and -0.07 < ρ < 0.07 au. View from Rh looking towards the alkane. **b)** View from above the alkane looking towards Rh.

Table S15: NBO data from **[1-hexane][BAr$^F_4$]** (partially optimised) showing the donating and accepting orbitals occupation and the 2nd order perturbation theory interaction energy.

**Donating Orbital**	**Occupation**	**Accepting Orbital**	**Occupation**	**Energy (kcal/mol)**
		C4–H41		
σ (C–H)	1.92	σ* (Rh–P_{trans})	0.26	10.14
σ (C–H)	1.92	σ* (Rh–P_{CIS})	0.25	0.10
LP (Rh)	1.96	σ* (C–H)	0.04	0.79
LP (Rh)	1.94	σ* (C–H)	0.04	1.42
LP (Rh)	1.89	σ* (C–H)	0.04	2.18
σ (Rh–P_{cis})	1.83	σ* (C–H)	0.04	1.79
σ (Rh–P_{trans})	1.83	σ* (C–H)	0.04	0.93
		C4–H42		
σ (C–H)	1.96	σ* (Rh–P_{trans})	0.25	2.11
LP (Rh)	1.96	σ* (C–H)	0.02	0.30
LP (Rh)	1.94	σ* (C–H)	0.02	0.27
LP (Rh)	1.90	σ* (C–H)	0.02	0.14
σ (Rh–P_{cis})	1.83	σ* (C–H)	0.02	0.26
σ (Rh–P_{trans})	1.83	σ* (C–H)	0.02	0.18
		C2–H21		
σ (C–H)	1.90	σ* (Rh–P_{trans})	0.26	13.78
LP (Rh)	1.96	σ* (C–H)	0.04	0.10
LP (Rh)	1.94	σ* (C–H)	0.04	2.34
LP (Rh)	1.89	σ* (C–H)	0.04	2.98
σ (Rh–P_{cis})	1.83	σ* (C–H)	0.04	2.43
σ (Rh–P_{trans})	1.83	σ* (C–H)	0.04	0.96
		C2–H22		
σ (C–H)	1.96	σ* (Rh–P_{trans})	0.26	2.38
LP (Rh)	1.96	σ* (C–H)	0.02	0.83
LP (Rh)	1.94	σ* (C–H)	0.02	0.11
LP (Rh)	1.90	σ* (C–H)	0.02	0.15
σ (Rh–P_{cis})	1.83	σ* (C–H)	0.02	0.41
σ (Rh–P_{trans})	1.83	σ* (C–H)	0.02	0.28

Table S16: NBO data from **[1-hexane][BAr$^F_4$]** (fully optimised) showing the donating and accepting orbitals occupation and the 2nd order perturbation theory interaction energy.

Donating Orbital	Occupation	Accepting Orbital	Occupation	Energy (kcal/mol)
		C4–H41		
σ (C–H)	1.91	σ* (Rh–P_{trans})	0.25	11.45
σ (C–H)	1.91	σ* (Rh–P_{CIS})	0.26	0.17
LP (Rh)	1.96	σ* (C–H)	0.04	0.49
LP (Rh)	1.94	σ* (C–H)	0.04	1.55
LP (Rh)	1.90	σ* (C–H)	0.04	0.23
LP (Rh)	1.89	σ* (C–H)	0.04	2.26
σ (Rh–P_{cis})	1.91	σ* (C–H)	0.04	2.44
σ (Rh–P_{trans})	1.91	σ* (C–H)	0.04	1.38
		C4–H42		
σ (C–H)	1.97	σ* (Rh–P_{trans})	0.25	1.35
LP (Rh)	1.97	σ* (C–H)	0.02	0.32
LP (Rh)	1.94	σ* (C–H)	0.02	0.18
LP (Rh)	1.90	σ* (C–H)	0.02	0.08
σ (Rh–P_{cis})	1.83	σ* (C–H)	0.02	0.27
σ (Rh-P_{trans})	1.83	σ* (C–H)	0.02	0.22
		C2–H21		
σ (C–H)	1.90	σ* (Rh–P_{trans})	0.26	14.12
LP (Rh)	1.97	σ* (C–H)	0.04	0.08
LP (Rh)	1.94	σ* (C–H)	0.04	2.44
LP (Rh)	1.90	σ* (C–H)	0.04	0.20
LP (Rh)	1.89	σ* (C–H)	0.04	2.91
σ (Rh–P_{cis})	1.83	σ* (C–H)	0.04	2.44
σ (Rh–P_{trans})	1.83	σ* (C–H)	0.04	0.95
		C2–H22		
σ (C–H)	1.97	σ* (Rh–P_{trans})	0.26	2.15
LP (Rh)	1.97	σ* (C–H)	0.02	0.81
LP (Rh)	1.94	σ* (C–H)	0.02	0.11
LP (Rh)	1.90	σ* (C–H)	0.02	0.14
σ (Rh–P_{cis})	1.83	σ* (C–H)	0.02	0.39
σ (Rh–P_{trans})	1.83	σ* (C–H)	0.02	0.25

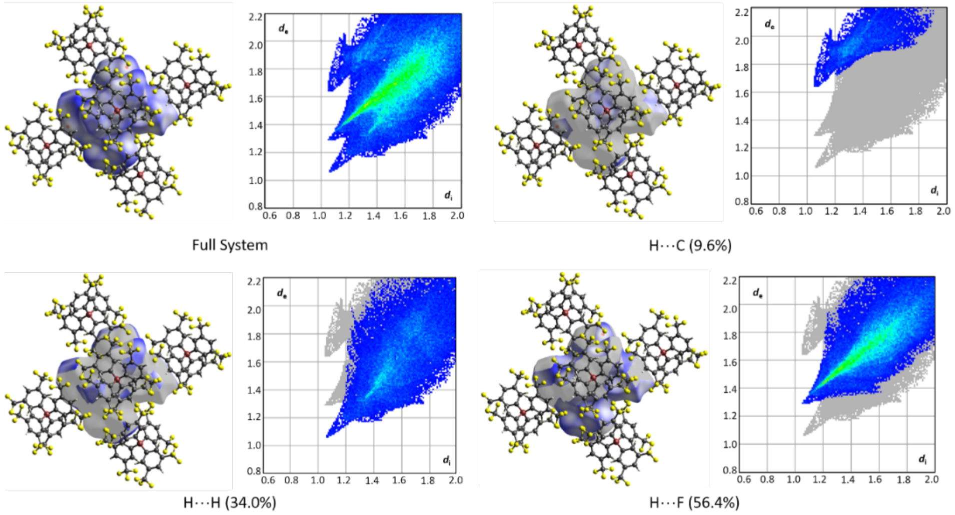

Figure S89: Hirshfeld Surfaces of **[1-hexane][BAr^F$_4$]** (partially optimised) showing normalised distance and associated fingerprint plots. Percentage of each type of contact is shown in brackets.

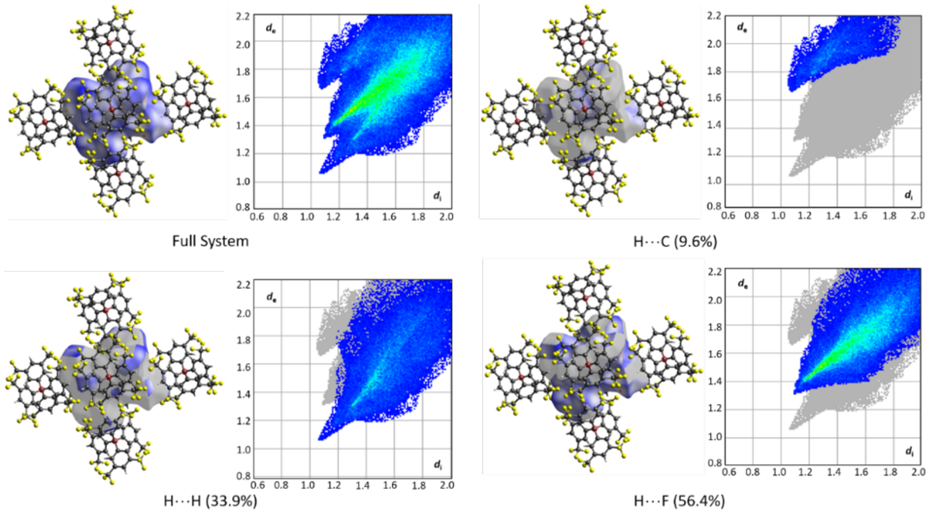

Figure S90: Hirshfeld Surfaces of **[1-hexane][BAr^F$_4$]** (fully optimised) showing normalised distance and associated fingerprint plots. Percentage of each type of contact is shown in brackets.

S.5.5. [1-(3-methylpentane)][BAr^F_4]

[1-(3-methylpentane)][BAr^F_4] (Partially Optimised)

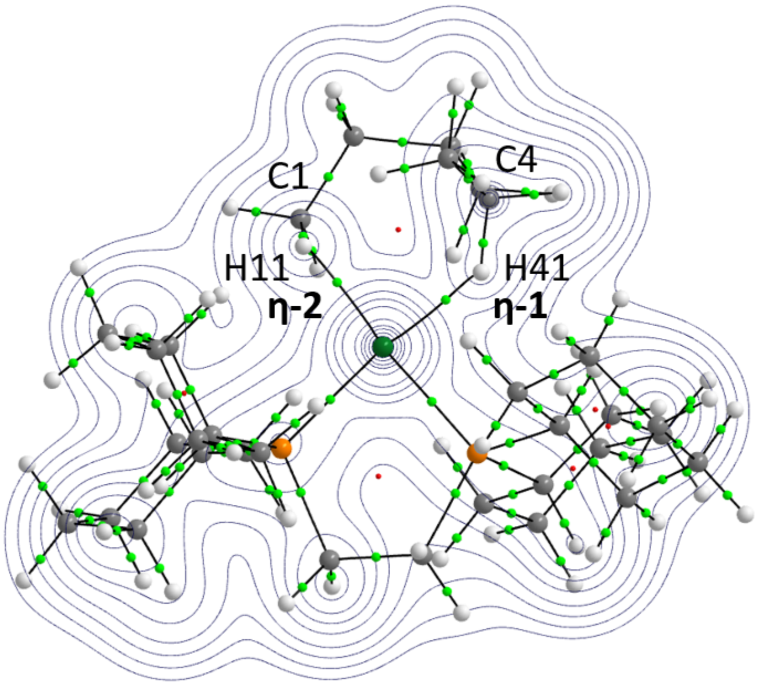

Figure S91: QTAIM molecular graph of **[1-(3-methylpentane)][BAr^F_4]** (partially optimised) with electron density contours in the H11–Rh–H41 plane.

Table S17: QTAIM BCP data and bond lengths for key bonds.

Bond Path	Interatomic Distance (Å)	ρ (e bohr^{-3})	$\nabla^2\rho$ (e bohr^{-5})	H(r)
C1 –H11	1.132	0.2472	-0.7132	-0.2282
H11–Rh	1.978	0.0520	0.2059	-0.0051
C1–Rh	2.429		**No BCP**	
Rh–P1	2.196	0.1210	0.0673	0.0615
C4–H41	1.136	0.2887	-0.6525	-0.2045
H41–Rh	1.885	0.0537	0.1782	-0.0104
C4–Rh	2.788		**No BCP**	
Rh–P2	2.196	0.1217	0.0728	0.0619

[1-(3-methylpentane)][BAr$^{F}_{4}$] (fully Optimised)

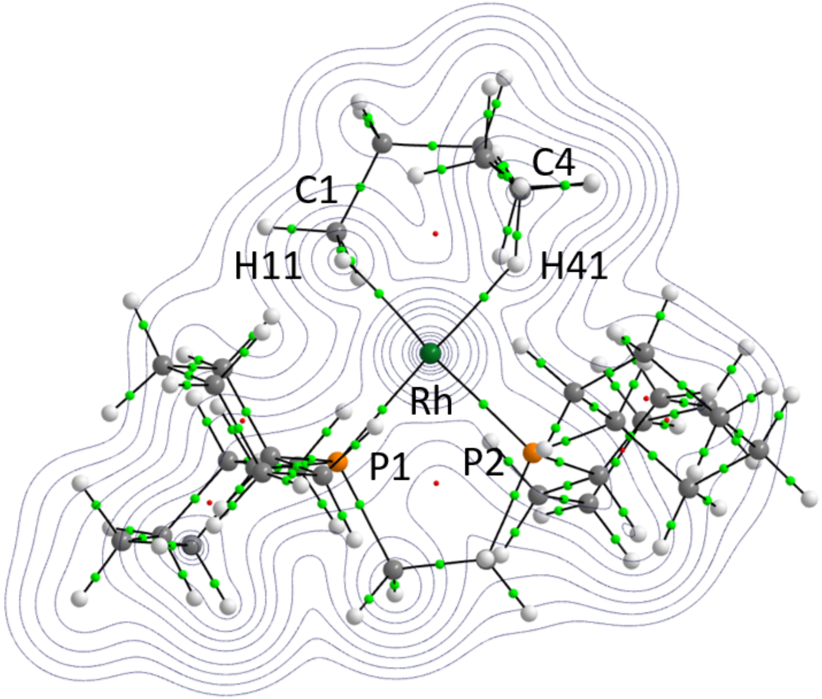

Figure S92: QTAIM molecular graph of **[1-(3-methylpentane)][BAr$^{F}_{4}$]** (fully optimised) with electron density contours in the H11–Rh–H41 plane.

Table S18: QTAIM BCP data and bond lengths for key bonds.

Bond Path	Interatomic Distance (Å)	ρ (e bohr^{-3})	$\nabla^2\rho$ (e bohr^{-5})	H(r)
C1 –H11	1.135	0.2450	-0.7003	-0.2244
H11–Rh	1.979	0.0504	0.1963	-0.0054
C1–Rh	2.463		**No BCP**	
Rh–P1	2.200	0.1201	0.0667	-0.0607
C4–H41	1.141	0.2360	-0.6339	-0.2094
H41–Rh	1.928	0.0492	0.1517	-0.0074
C4–Rh	2.891		**No BCP**	
Rh–P2	2.211	0.1185	0.0779	-0.0582

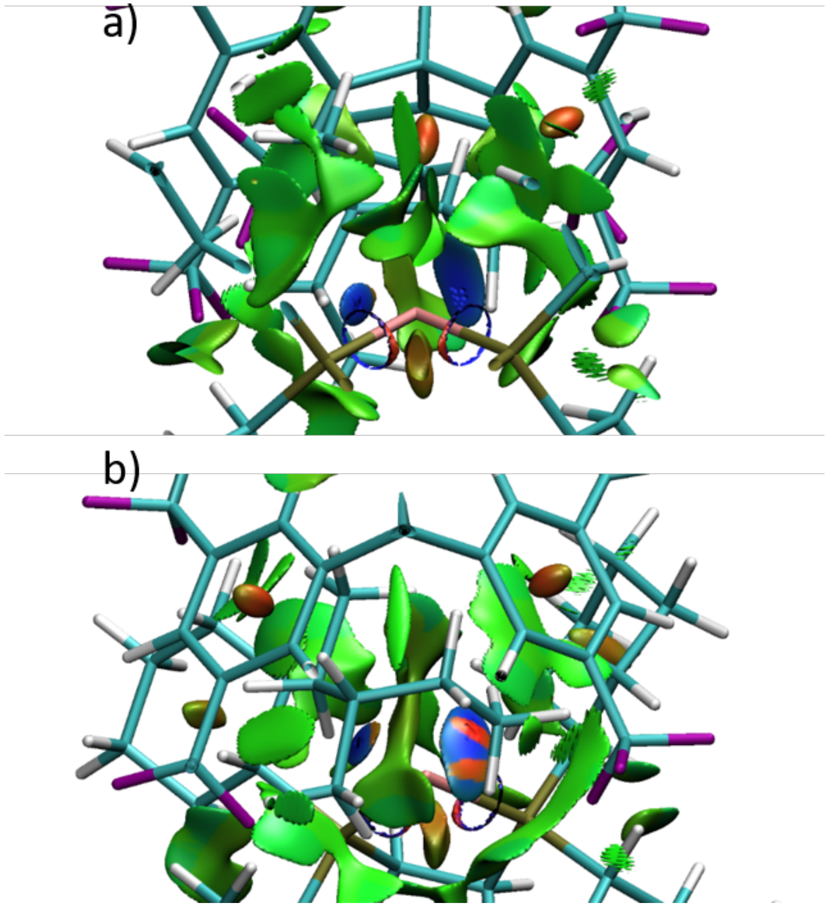

Figure S93: a) NCI Plot of **[1-(3-methylpentane)][BAr$^{F}{}_{4}$]** (partially optimised) with isosurfaces generated for s = 0.3 au and -0.07 < ρ < 0.07 au. View from Rh looking towards the alkane. **b)** View from above the alkane looking towards Rh.

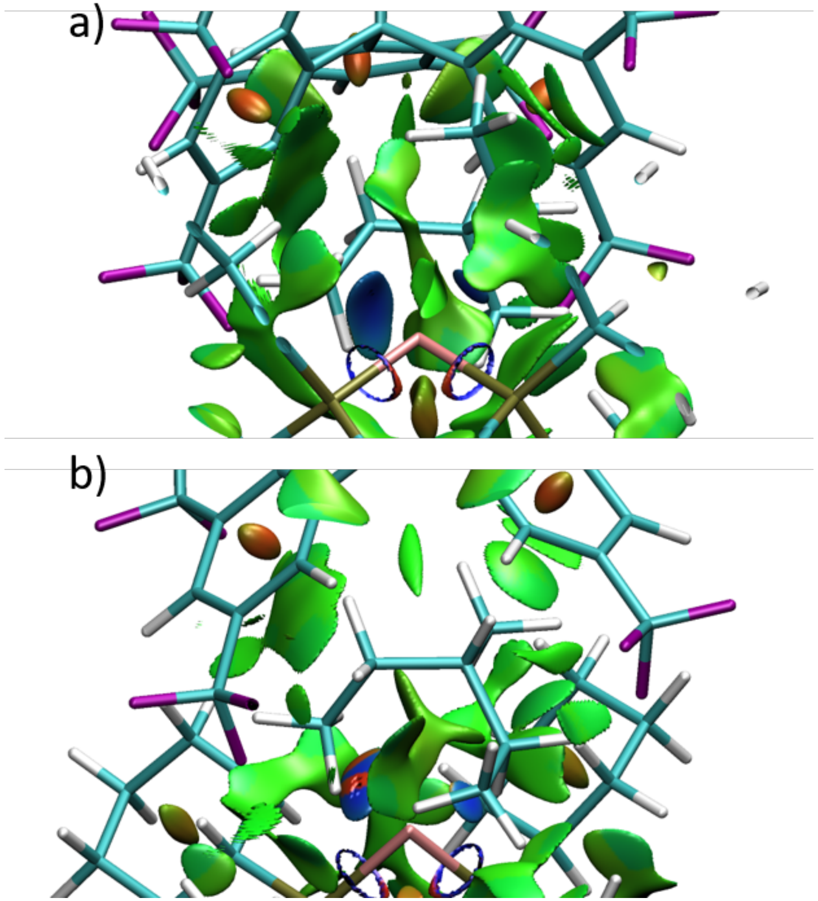

Figure S94: a) NCI Plot of **[1-(3-methylpentane)][BAr$^{F}{}_{4}$]** (fully optimised) with isosurfaces generated for s = 0.3 au and -0.07 < ρ < 0.07 au. View from Rh looking towards the alkane. **b)** View from above the alkane looking towards Rh.

Table S19: NBO data from **[1-(3-methylpentane)][BAr^F$_4$]** (partially optimised) showing the donating and accepting orbitals occupation and the 2nd order perturbation theory interaction energy.

**Donating Orbital**	**Occupation**	**Accepting Orbital**	**Occupation**	**Energy (kcal/mol)**
		C1–H11		
σ (C–H)	1.92	σ* (Rh–P_{trans})	0.28	13.44
LP (Rh)	1.96	σ* (C–H)	0.04	0.24
LP (Rh)	1.95	σ* (C–H)	0.04	1.98
LP (Rh)	1.90	σ* (C–H)	0.04	0.91
LP (Rh)	1.89	σ* (C–H)	0.04	3.05
σ (Rh–P_{cis})	1.81	σ* (C–H)	0.04	2.10
σ (Rh–P_{trans})	1.82	σ* (C–H)	0.04	0.84
		C1–H12		
σ (C–H)	1.95	σ* (Rh–P_{trans})	0.28	5.90
LP (Rh)	1.96	σ* (C–H)	0.03	2.03
LP (Rh)	1.95	σ* (C–H)	0.03	0.12
LP (Rh)	1.90	σ* (C–H)	0.03	0.69
LP (Rh)	1.89	σ* (C–H)	0.03	0.34
σ (Rh–P_{cis})	0.82	σ* (C–H)	0.03	0.96
σ (Rh–P_{trans})	0.81	σ* (C–H)	0.03	0.55
		C4–H41		
σ (C–H)	1.89	σ* (Rh–P_{trans})	0.28	14.23
LP (Rh)	1.96	σ* (C–H)	0.04	0.11
LP (Rh)	1.95	σ* (C–H)	0.04	0.38
LP (Rh)	1.90	σ* (C–H)	0.04	0.96
LP (Rh)	1.89	σ* (C–H)	0.04	1.19
σ (Rh–P_{cis})	1.82	σ* (C–H)	0.04	7.01
σ (Rh–P_{trans})	1.81	σ* (C–H)	0.04	2.89

Table S20: NBO data from **[1-(3-methylpentane)][BAr$^F{}_4$]** (fully optimised) showing the donating and accepting orbitals occupation and the 2nd order perturbation theory interaction energy. Selected donor-acceptor interactions are shown below.

**Donating Orbital**	**Occupation**	**Accepting Orbital**	**Occupation**	**Energy (kcal/mol)**
		C1–H11		
σ (C–H)	1.91	σ* (Rh–P_{trans})	0.28	13.75
LP (Rh)	1.96	σ* (C–H)	0.03	0.14
LP (Rh)	1.94	σ* (C–H)	0.03	2.07
LP (Rh)	1.90	σ* (C–H)	0.03	0.39
LP (Rh)	1.89	σ* (C–H)	0.03	3.54
σ (Rh–P_{cis})	0.82	σ* (C–H)	0.03	2.16
σ (Rh–P_{trans})	0.82	σ* (C–H)	0.03	0.85
		C1–H12		
σ (C–H)	1.96	σ* (Rh–P_{trans})	0.28	4.83
LP (Rh)	1.96	σ* (C–H)	0.02	1.85
LP (Rh)	1.94	σ* (C–H)	0.02	0.14
LP (Rh)	1.90	σ* (C–H)	0.02	0.32
LP (Rh)	1.89	σ* (C–H)	0.02	0.41
σ (Rh–P_{cis})	0.82	σ* (C–H)	0.02	0.82
σ (Rh–P_{trans})	0.82	σ* (C–H)	0.02	0.51
		C4–H41		
σ (C–H)	1.89	σ* (Rh–P_{trans})	0.28	12.61
σ (C–H)	1.89	σ* (Rh–P_{cis})	0.28	0.07
LP (Rh)	1.94	σ* (C–H)	0.04	1.22
LP (Rh)	1.90	σ* (C–H)	0.04	0.16
LP (Rh)	1.89	σ* (C–H)	0.04	0.98
σ (Rh–P_{cis})	1.82	σ* (C–H)	0.04	6.25[a]
σ (Rh–P_{trans})	1.82	σ* (C–H)	0.04	3.31[b]

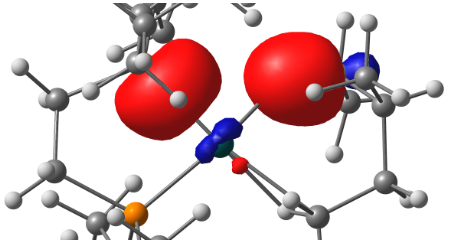

a = ΔE = 6.25 kcal/mol

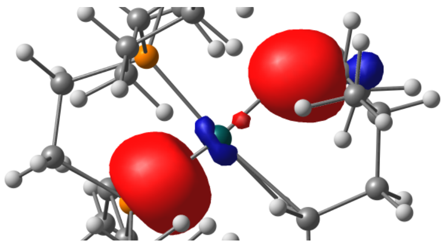

b = ΔE = 3.31 kcal/mol

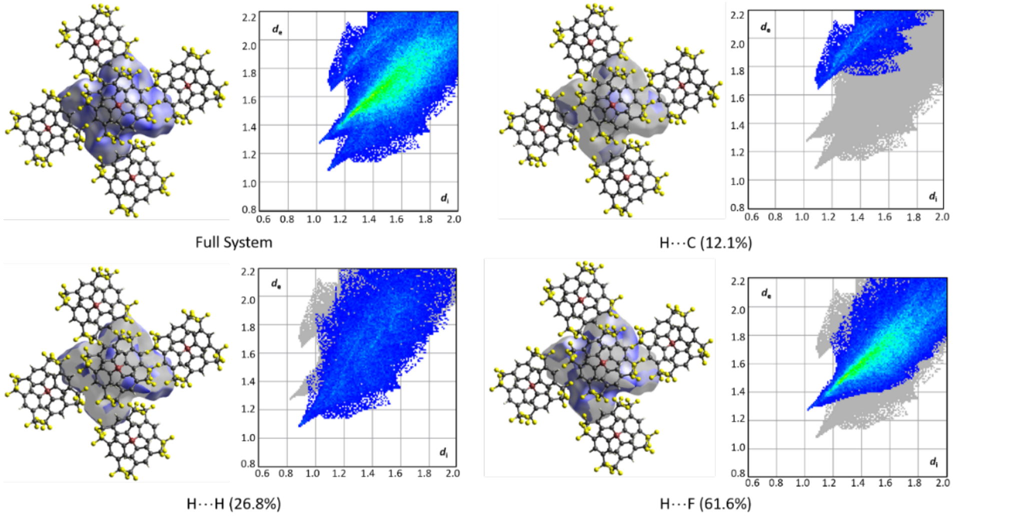

Figure S95: Hirshfeld Surfaces of **[1-(3-methylpentane)][BAr^F$_4$]** (partially optimised) showing normalised distance and associated fingerprint plots. Percentage of each type of contact is shown in brackets.

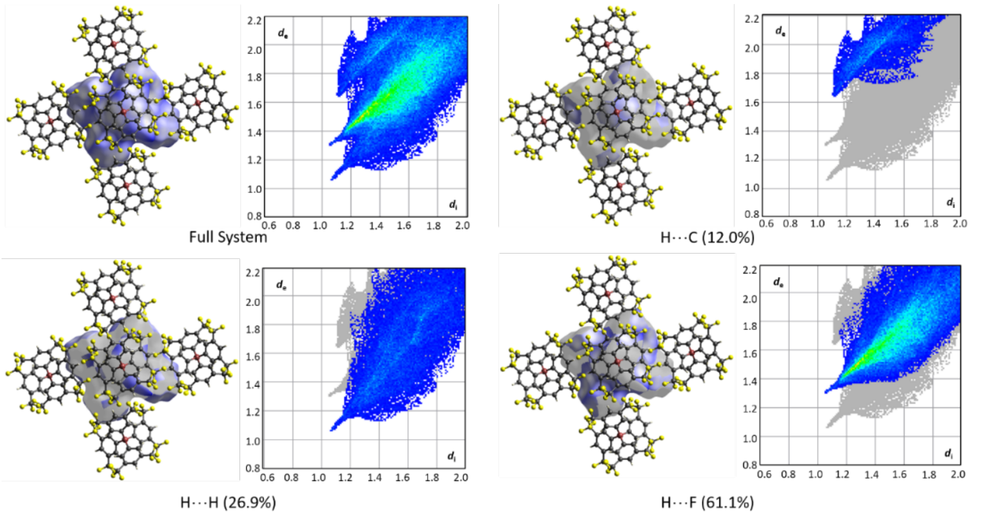

Figure S96: Hirshfeld Surfaces of **[1-(3-methylpentane)][BAr^F$_4$]** (fully optimised) showing normalised distance and associated fingerprint plots. Percentage of each type of contact is shown in brackets.

S.5.6. [1-cyclohexane][BAr^F_4]

Note: The structure of [Rh($Cy_2PCH_2CH_2PCy_2$)(C_6H_{12})][BAr^F_4], **[1-cylohexane][BAr^F_4]** has been previous reported.[S7] We now report results using the protocol adopted in this paper for consistency.

[1-cyclohexane][BAr^F_4] (Partially Optimised)

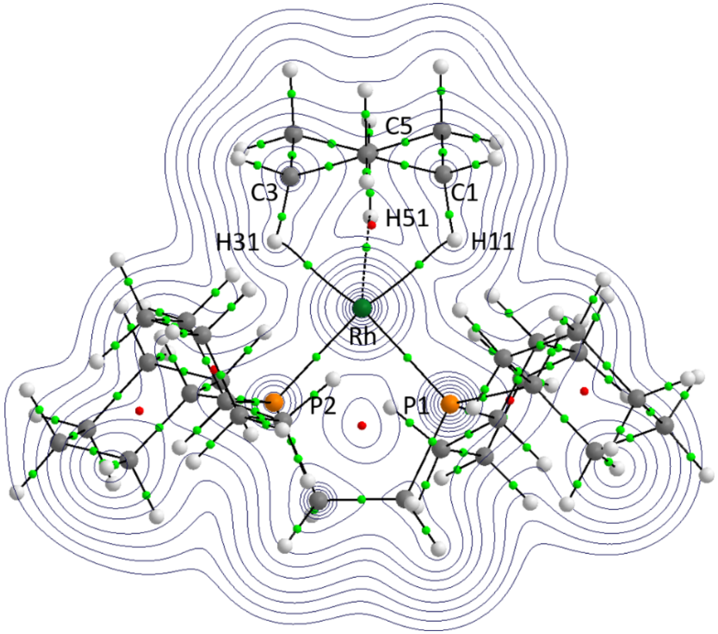

Figure S97: QTAIM molecular graph of **[1-cyclohexane][BAr^F_4]** (partially optimised) with electron density contours in the H11–Rh–H31 plane.

Table S21: QTAIM BCP data and bond lengths for key bonds.

Bond Path	Interatomic Distance (Å)	ρ (e bohr^{-3})	$\nabla^2\rho$ (e bohr^{-5})	H(r)
C1–H11	1.137	0.2415	-0.6706	-0.2186
H11–Rh	1.933	0.0503	0.1815	-0.0079
C1–Rh	2.642		**No BCP**	
Rh–P1	2.192	0.1228	0.0703	-0.0630
C3–H31	1.141	0.2388	-0.6476	-0.2139
H31–Rh	1.852	0.0610	0.2257	-0.0114
C3–Rh	2.530		**No BCP**	
Rh–P2	2.192	0.1226	0.0647	-0.0633
C5–H51	1.109	0.2660	-0.8574	-0.2599
H51–Rh	2.677	0.0148	0.0386	0.0000
C5–Rh	3.306		**No BCP**	

[1-cyclohexane][BAr$^F{}_4$] (Fully Optimised)

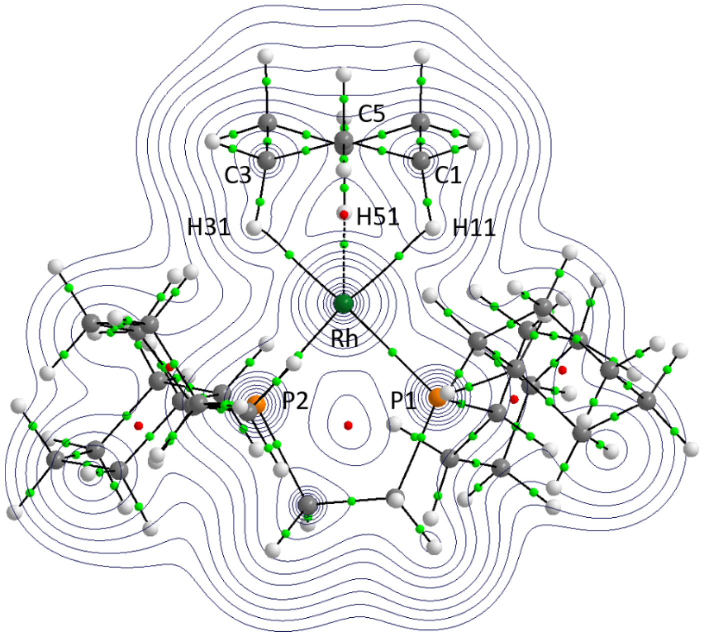

Figure S98: QTAIM molecular graph of **[1-cyclohexane][BAr$^F{}_4$]** (fully optimised) with electron density contours in the H11–Rh–H31 plane.

Table S22: QTAIM BCP data and bond lengths for key bonds.

Bond Path	Interatomic Distance (Å)	ρ (e bohr^{-3})	$\nabla^2\rho$ (e bohr^{-5})	H(r)
C1–H11	1.139	0.2392	-0.6538	-0.2148
H11–Rh	1.923	0.0505	0.1815	-0.0082
C1–Rh	2.681		**No BCP**	
Rh–P1	2.195	0.1216	0.0657	-0.0621
C3–H31	1.140	0.2388	-0.6514	-0.2141
H31–Rh	1.928	0.0495	0.1858	-0.0074
C3–Rh	2.679		**No BCP**	
Rh–P2	2.201	0.1202	0.0712	-0.0604
C5–H51	1.109	0.2660	-0.8574	-0.2599
H51–Rh	2.677	0.0148	0.0386	0.0000
C5–Rh	3.306		**No BCP**	

.

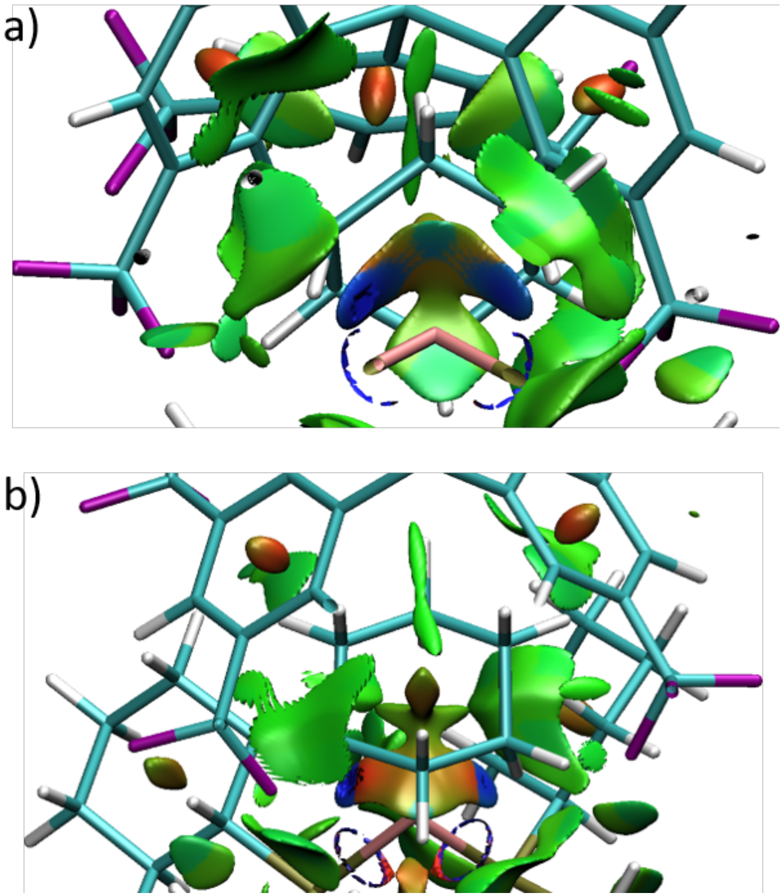

Figure S99: a) NCI Plot of **[1-cyclohexane][BAr$^F_4$]** (partially optimised) with isosurfaces generated for s = 0.3 au and -0.07 < ρ < 0.07 au. View from Rh looking towards the alkane. **b)** View from above the alkane looking towards Rh.

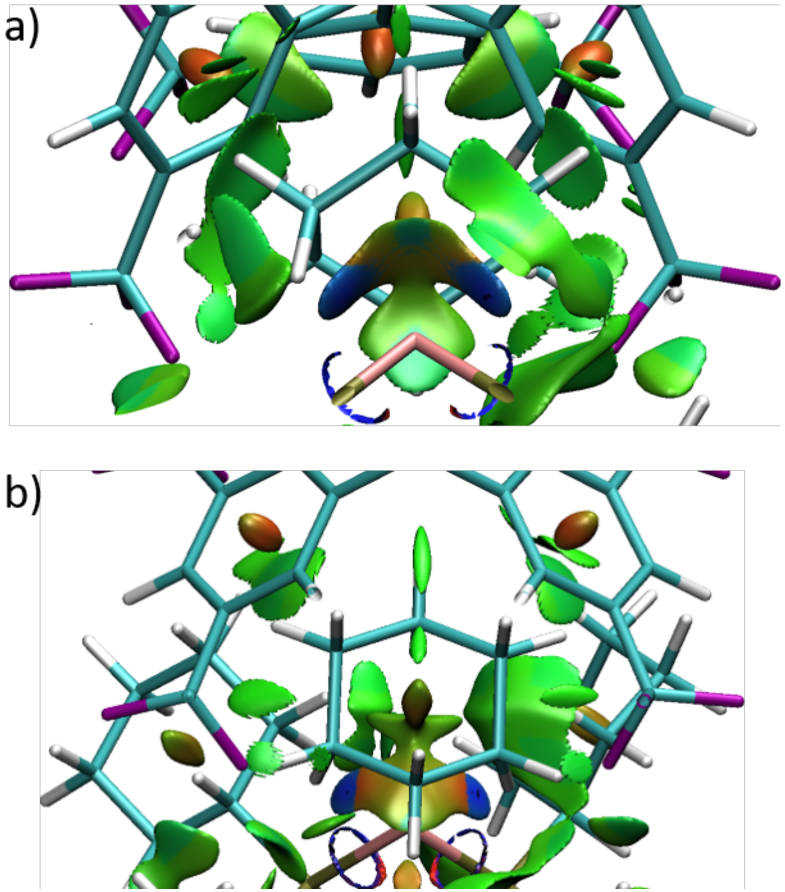

Figure S100: a) NCI Plot of **[1-cyclohexane][BAr$^F_4$]** (fully optimised) with isosurfaces generated for s = 0.3 au and -0.07 < ρ < 0.07 au. View from Rh looking towards the alkane. **b)** View from above the alkane looking towards Rh.

Table S23: NBO data from **[1-cyclohexane][BAr$^F_4$]** (partially optimised) showing the donating and accepting orbitals occupation and the 2nd order perturbation theory interaction energy.

**Donating Orbital**	**Occupation**	**Accepting Orbital**	**Occupation**	**Energy (kcal/mol)**
		C3–H31		
σ (C–H)	1.89	σ* (Rh–P_{trans})	0.28	15.13
LP (Rh)	1.97	σ* (C–H)	0.03	0.14
LP (Rh)	1.90	σ* (C–H)	0.03	0.50
LP (Rh)	1.88	σ* (C–H)	0.03	2.60
σ (Rh–P_{cis})	1.81	σ* (C–H)	0.03	5.45
σ (Rh–P_{trans})	1.81	σ* (C–H)	0.03	1.76
		C1–H11		
σ (C–H)	1.88	σ* (Rh–P_{trans})	0.29	20.03
LP (Rh)	1.97	σ* (C–H)	0.04	0.08
LP (Rh)	1.95	σ* (C–H)	0.04	0.42
LP (Rh)	1.90	σ* (C–H)	0.04	0.23
LP (Rh)	1.88	σ* (C–H)	0.04	4.26
σ (Rh–P_{cis})	1.81	σ* (C–H)	0.04	5.99
σ (Rh–P_{trans})	1.81	σ* (C–H)	0.04	1.63
		C5–H51		
σ (C–H)	1.97	σ* (Rh–P)	0.28	1.01
σ (C–H)	1.97	σ* (Rh–P)	0.29	0.68
LP (Rh)	1.97	σ* (C–H)	0.02	0.78
LP (Rh)	1.90	σ* (C–H)	0.02	0.46
LP (Rh)	1.88	σ* (C–H)	0.02	0.07
σ (Rh–P)	1.81	σ* (C–H)	0.02	0.89
σ (Rh–P)	1.81	σ* (C–H)	0.02	0.95

Table S24: NBO data from **[1-cyclohexane][BAr$^F_4$]** (fully optimised) showing the donating and accepting orbitals occupation and the 2nd order perturbation theory interaction energy. Selected donor-acceptor interactions are shown below.

**Donating Orbital**	**Occupation**	**Accepting Orbital**	**Occupation**	**Energy (kcal/mol)**
		C3–H31		
σ (C–H)	1.89	σ* (Rh–P_{trans})	0.28	15.81
LP (Rh)	1.97	σ* (C–H)	0.04	0.14
LP (Rh)	1.90	σ* (C–H)	0.04	0.21
LP (Rh)	1.89	σ* (C–H)	0.04	2.72[a]
σ (Rh–P_{cis})	1.81	σ* (C–H)	0.04	5.31[b]
σ (Rh–P_{trans})	1.82	σ* (C–H)	0.04	1.88
		C1–H11		
σ (C–H)	1.89	σ* (Rh–P_{trans})	0.28	16.51
σ (C–H)	1.89	σ* (Rh–P_{cis})	0.28	0.10
LP (Rh)	1.97	σ* (C–H)	0.03	0.08
LP (Rh)	1.95	σ* (C–H)	0.03	0.08
LP (Rh)	1.90	σ* (C–H)	0.03	0.10
LP (Rh)	1.89	σ* (C–H)	0.03	2.30
σ (Rh–P_{cis})	1.82	σ* (C–H)	0.03	5.54
σ (Rh–P_{trans})	1.81	σ* (C–H)	0.03	2.30
		C5–H51		
σ (C–H)	1.97	σ* (Rh–P)	0.28	0.81
σ (C–H)	1.97	σ* (Rh–P)	0.28	0.69
LP (Rh)	1.97	σ* (C–H)	0.02	1.01
LP (Rh)	1.90	σ* (C–H)	0.02	0.48
σ (Rh–P)	1.82	σ* (C–H)	0.02	0.89
σ (Rh–P)	1.81	σ* (C–H)	0.02	0.90

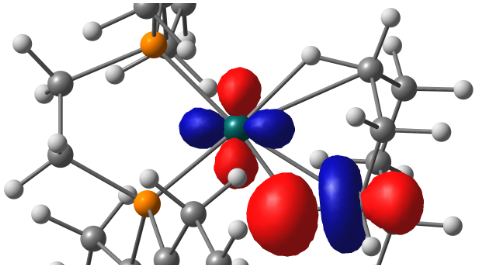

a = ΔE = 2.72 kcal/mol

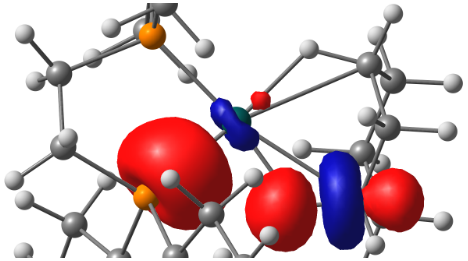

b = ΔE = 5.31 kcal/mol

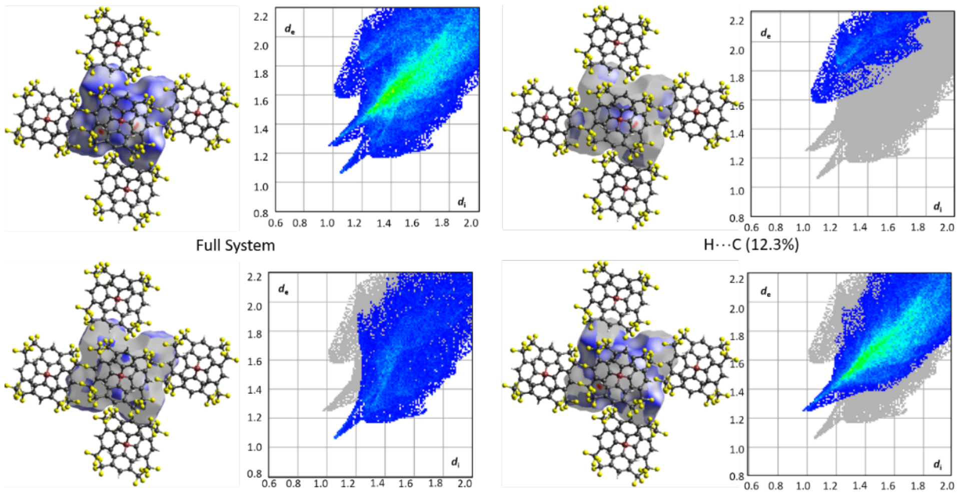

Figure S101: Hirshfeld Surfaces of **[1-cyclohexane][BAr^F$_4$]** (partially optimised) showing normalised distance and associated fingerprint plots. Percentage of each type of contact is shown in brackets.

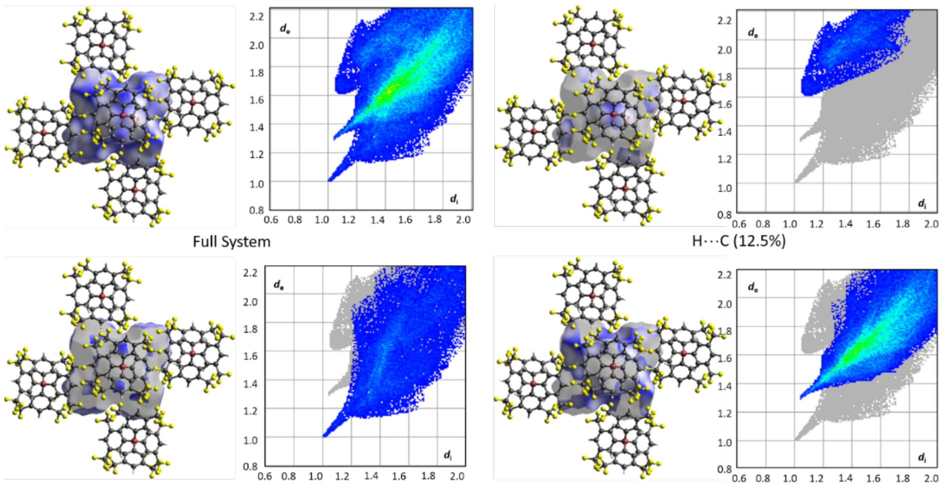

Figure S102: Hirshfeld Surfaces of **[1-cyclohexane][BAr^F$_4$]** (fully optimised) showing normalised distance and associated fingerprint plots. Percentage of each type of contact is shown in brackets.

S.5.7. [1-NBA][BAr$^F_4$]

Note: The structure of **[1-NBA][BAr$^F_4$]** has been previous reported.[S2] We now report results using the protocol adopted in this paper for consistency.

[1-NBA][BAr$^F_4$] (Partially Optimised)

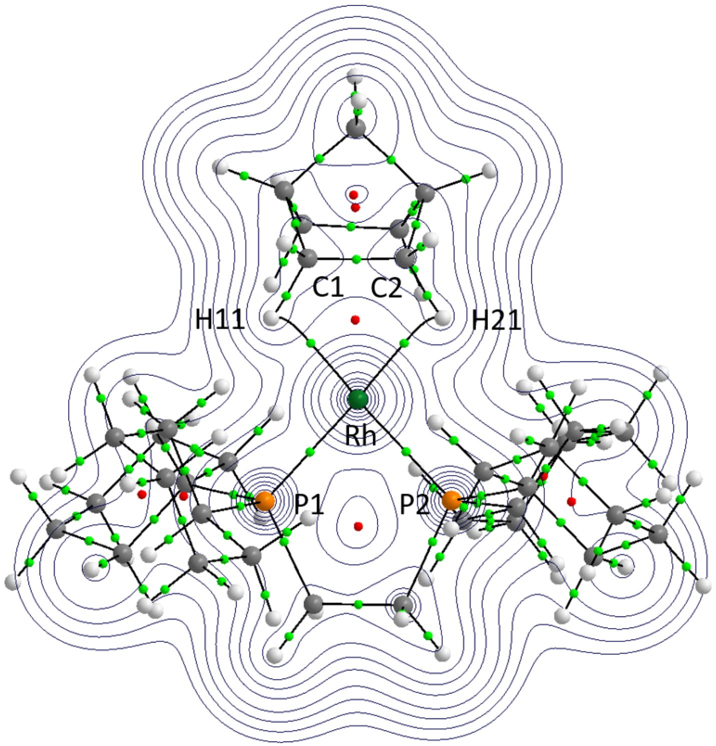

Figure S103: QTAIM molecular graph of **[1-NBA][BAr$^F_4$]** (partially optimised) with electron density contours in the H11–Rh–H21 plane.

Table S25: QTAIM BCP data and bond lengths for key bonds.

Bond Path	Interatomic Distance (Å)	ρ (e bohr^{-3})	∇^{2}ρ (e bohr^{-5})	H(r)
C1 –H11	1.153	0.2337	-0.6206	-0.2040
H11–Rh	1.889	0.0593	0.2463	-0.0075
C1–Rh	2.400		**No BCP**	
Rh–P1	2.195	0.1218	0.0733	-0.0619
C2 –H21	1.155	0.2321	-0.6088	-0.2012
H21–Rh	1.872	0.0617	0.2534	-0.0086
C2–Rh	2.389		**No BCP**	
Rh–P2	2.193	0.1221	0.0710	-0.0624

[1-NBA][BAr^F$_4$] (Fully Optimised)

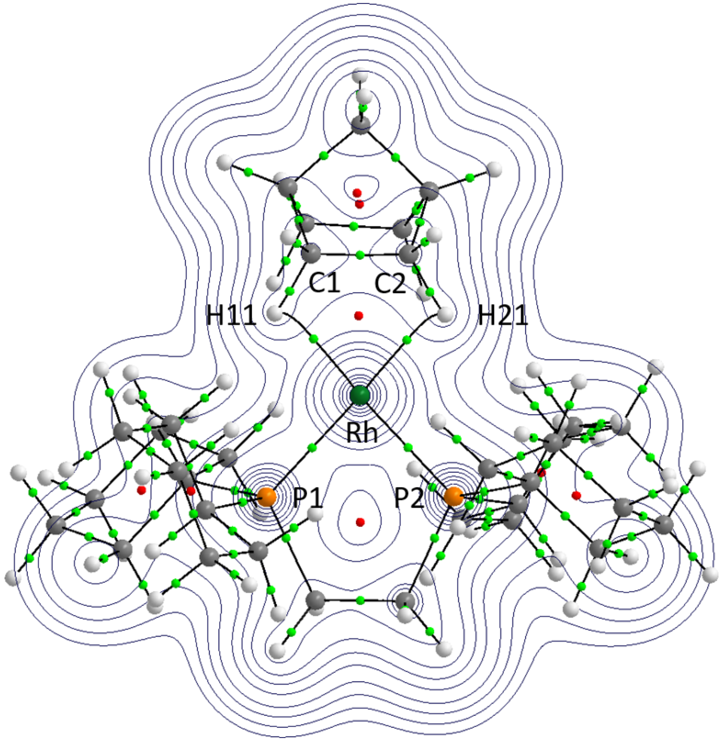

Figure S104: QTAIM molecular graph of **[1-NBA][BAr^F$_4$]** (fully optimised) with electron density contours in the H11–Rh–H21 plane.

Table S26: QTAIM BCP data and bond lengths for key bonds.

Bond Path	Interatomic Distance (Å)	ρ (e bohr^{-3})	$\nabla^2\rho$ (e bohr^{-5})	H(r)
C1 –H11	1.153	0.2336	-0.6209	-0.2038
H11–Rh	1.892	0.0584	0.2412	-0.0073
C1–Rh	2.415		**No BCP**	
Rh–P1	2.210	0.1186	0.0788	-0.0582
C2 –H21	1.154	0.2325	-0.6134	-0.2022
H21–Rh	1.877	0.0604	0.2474	-0.0083
C2–Rh	2.406		**No BCP**	
Rh–P2	2.210	0.1185	0.0774	-0.0583

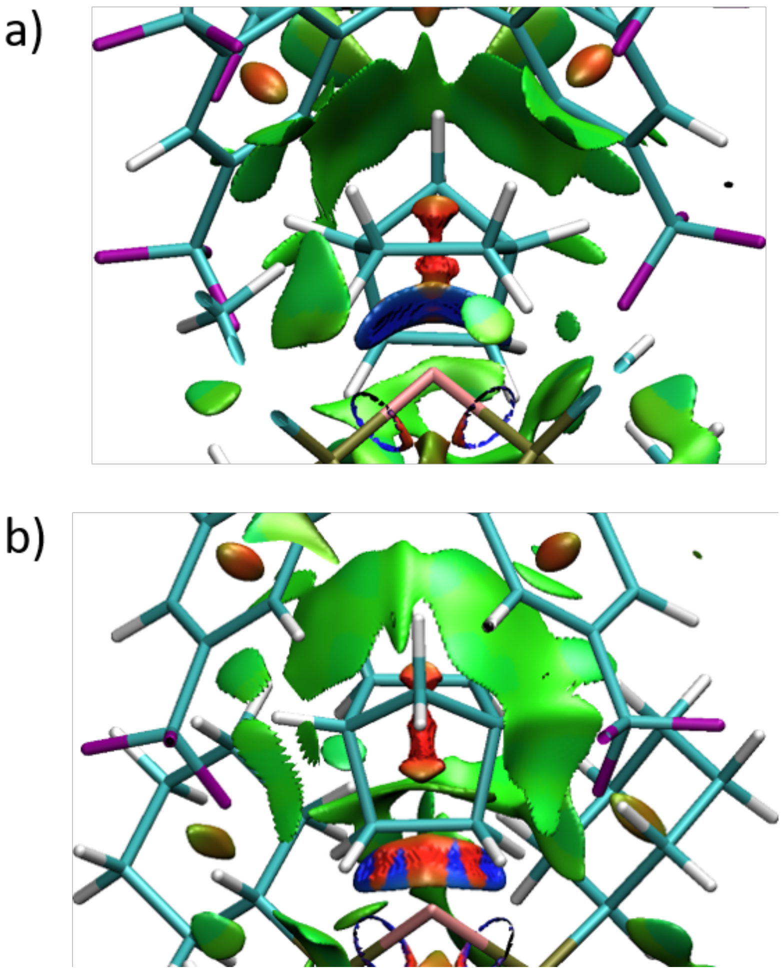

Figure S105: a) NCI Plot of **[1-NBA][BAr$^F{}_4$]** (partially optimised) with isosurfaces generated for s = 0.3 au and -0.07 < ρ < 0.07 au. View from Rh looking towards the alkane. **b)** View from above the alkane looking towards Rh.

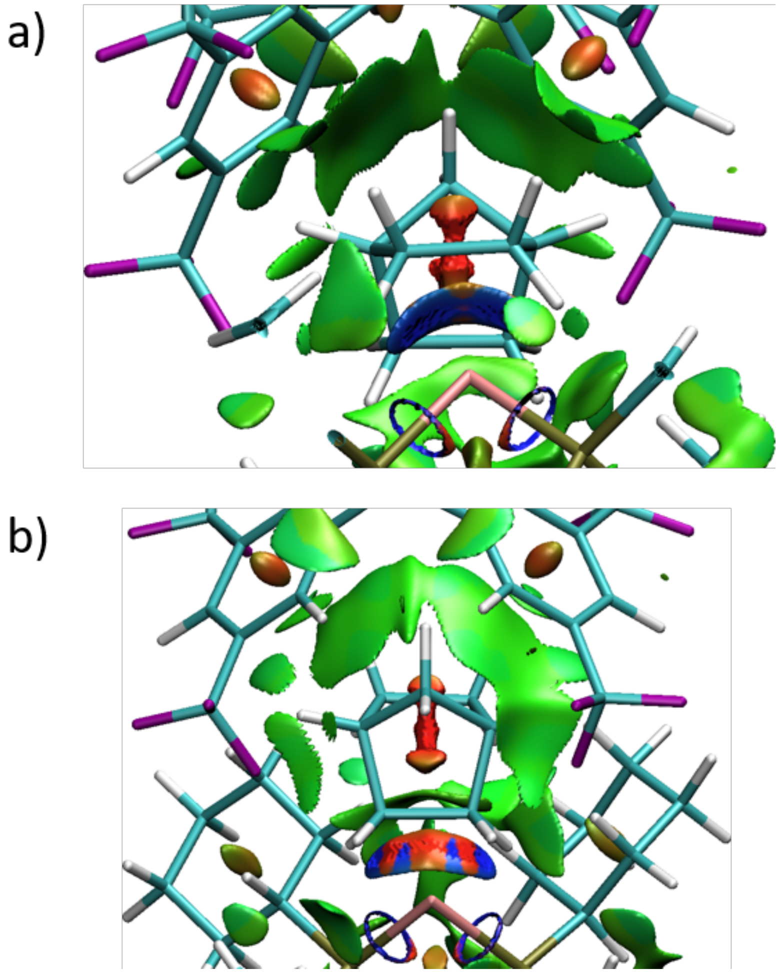

Figure S106: a) NCI Plot of **[1-NBA][BAr$^F{}_4$]** (fully optimised) with isosurfaces generated for s = 0.3 au and -0.07 < ρ < 0.07 au. View from Rh looking towards the alkane. **b)** View from above the alkane looking towards Rh.

Table S27: NBO data from **[1-NBA][BAr^F$_4$]** (partially optimised) showing the donating and accepting orbitals occupation and the 2nd order perturbation theory interaction energy.

**Donating Orbital**	**Occupation**	**Accepting Orbital**	**Occupation**	**Energy (kcal/mol)**
		C2–H21		
σ (C–H)	1.87	σ* (Rh–P_{trans})	0.29	20.83
σ (C–H)	1.87	σ* (Rh–P_{cis})	0.29	0.10
LP (Rh)	1.97	σ* (C–H)	0.04	0.05
LP (Rh)	1.95	σ* (C–H)	0.04	0.25
LP (Rh)	1.87	σ* (C–H)	0.04	4.15
σ (Rh–P_{cis})	1.83	σ* (C–H)	0.04	3.73
σ (Rh–P_{trans})	1.83	σ* (C–H)	0.04	1.18
		C1–H11		
σ (C–H)	1.87	σ* (Rh–P_{trans})	0.29	22.35
σ (C–H)	1.87	σ* (Rh–P_{cis})	0.29	0.14
LP (Rh)	1.95	σ* (C–H)	0.04	0.12
LP (Rh)	1.87	σ* (C–H)	0.04	4.65
σ (Rh–P_{cis})	1.83	σ* (C–H)	0.04	3.62
σ (Rh–P_{trans})	1.83	σ* (C–H)	0.04	1.07

Table S28: NBO data from **[1-NBA][BAr^F$_4$]** (fully optimised) showing the donating and accepting orbitals occupation and the 2nd order perturbation theory interaction energy.

**Donating Orbital**	**Occupation**	**Accepting Orbital**	**Occupation**	**Energy (kcal/mol)**
		C2–H21		
σ (C–H)	1.87	σ* (Rh–P_{trans})	0.28	20.46
σ (C–H)	1.87	σ* (Rh–P_{cis})	0.29	0.08
LP (Rh)	1.97	σ* (C–H)	0.04	0.06
LP (Rh)	1.95	σ* (C–H)	0.04	0.25
LP (Rh)	1.87	σ* (C–H)	0.04	3.99
σ (Rh–P_{cis})	1.83	σ* (C–H)	0.04	3.53
σ (Rh–P_{trans})	1.83	σ* (C–H)	0.04	1.16
		C1–H11		
σ (C–H)	1.87	σ* (Rh–P_{trans})	0.29	21.82
σ (C–H)	1.87	σ* (Rh–P_{cis})	0.28	0.10
LP (Rh)	1.97	σ* (C–H)	0.04	0.06
LP (Rh)	1.95	σ* (C–H)	0.04	0.07
LP (Rh)	1.87	σ* (C–H)	0.04	4.53
σ (Rh–P_{cis})	1.83	σ* (C–H)	0.04	3.49
σ (Rh–P_{trans})	1.83	σ* (C–H)	0.04	1.05

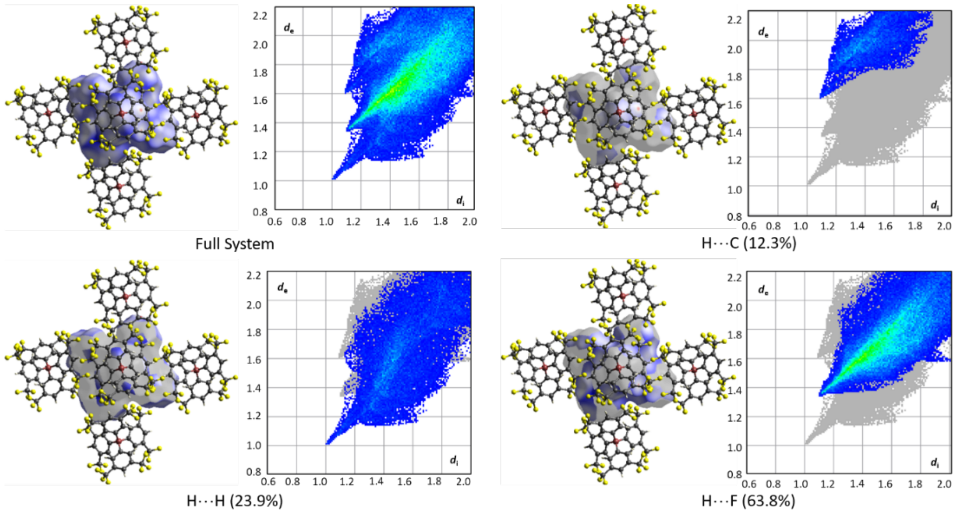

Figure S107: Hirshfeld Surfaces of **[1-NBA][BAr^F$_4$]** (partially optimised) showing normalised distance and associated fingerprint plots. Percentage of each type of contact is shown in brackets.

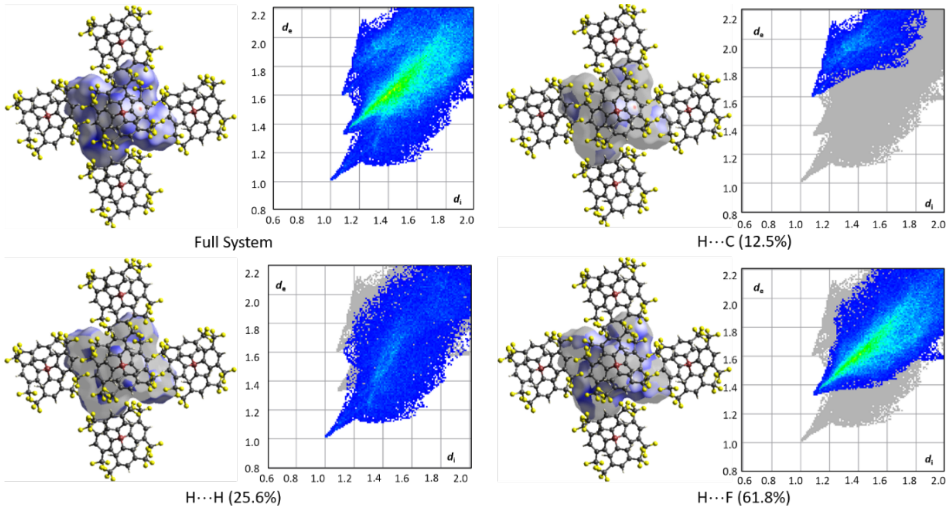

Figure S108: Hirshfeld Surfaces of **[1-NBA][BAr^F$_4$]** (fully optimised) showing normalised distance and associated fingerprint plots. Percentage of each type of contact is shown in brackets.

S.5.8. [1-isobutane][BAr$^F_4$]

Note: The structure of **[1-isobutane][BAr$^F_4$]** has been previous reported.[S7] We now report results using the protocol adopted in this paper for consistency.

Isobutane (Partially Optimised)

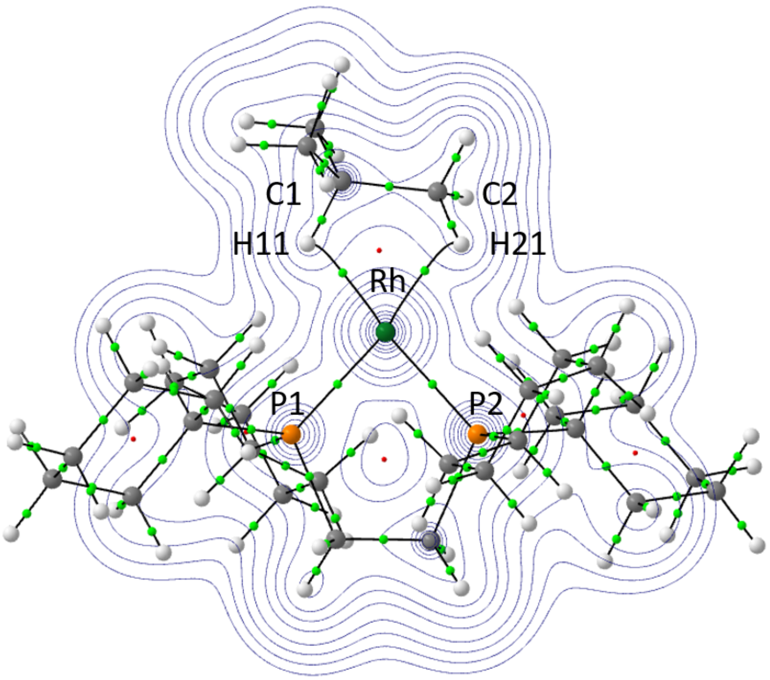

Figure S109: QTAIM molecular graph of **[1-isobutane][BAr$^F_4$]** (partially optimised) with electron density contours in the H11–Rh–H21 plane.

Table S29: QTAIM BCP data and bond lengths for key bonds.

Bond Path	Interatomic Distance (Å)	ρ (e bohr^{-3})	∇²ρ (e bohr^{-5})	H(r)
C1 –H11	1.160	0.2316	-0.6036	-0.1997
H11–Rh	1.873	0.0603	0.2414	-0.0090
C1–Rh	2.442		**No BCP**	
Rh–P1	2.191	0.1127	0.0679	-0.0631
C2 –H21	1.148	0.2364	-0.6433	-0.2093
H21–Rh	1.933	0.0562	0.2467	-0.0042
C2–Rh	2.363		**No BCP**	
Rh–P2	2.183	0.1243	0.0658	-0.0650

Isobutane (Fully Optimised)

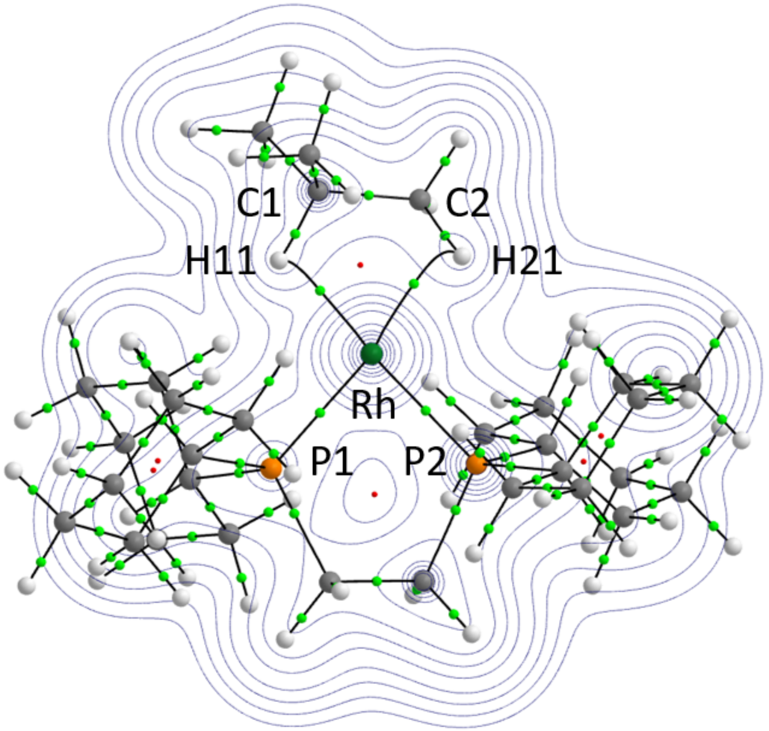

Figure S110: QTAIM molecular graph of **[1-isobutane][BAr$^F_4$]** (fully optimised) with electron density contours in the H11–Rh–H21 plane.

Table S30: QTAIM BCP data and bond lengths for key bonds.

Bond Path	Interatomic Distance (Å)	ρ (e bohr^{-3})	$\nabla^2\rho$ (e bohr^{-5})	H(r)
C1 –H11	1.159	0.2321	-0.6093	-0.2008
H11–Rh	1.898	0.0566	0.2253	-0.0078
C1–Rh	2.480		**No BCP**	
Rh–P1	2.207	0.1192	0.0754	-0.0590
C2 –H21	1.150	0.2353	-0.6356	-0.2073
H21–Rh	1.924	0.0572	0.2481	-0.0049
C2–Rh	2.361		**No BCP**	
Rh–P2	2.200	0.1260	0.0714	-0.0607

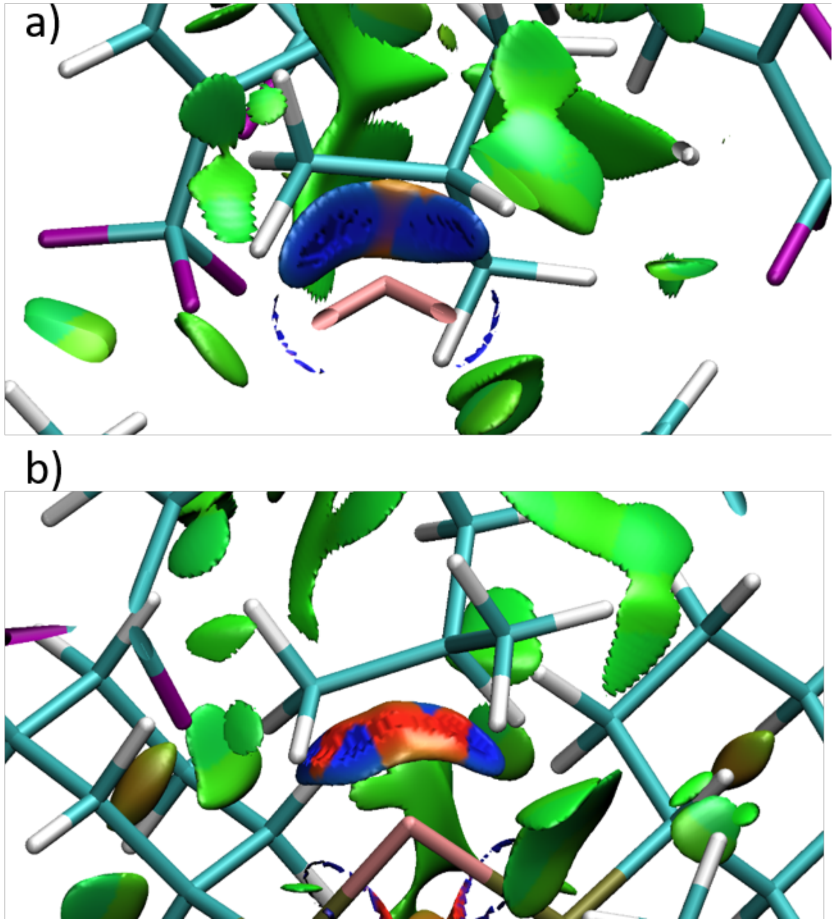

Figure S111: a) NCI Plot of **[1-isobutane][BAr$^F{}_4$]** (partially optimised) with isosurfaces generated for s = 0.3 au and -0.07 < ρ < 0.07 au. View from Rh looking towards the alkane. **b)** View from above the alkane looking towards Rh.

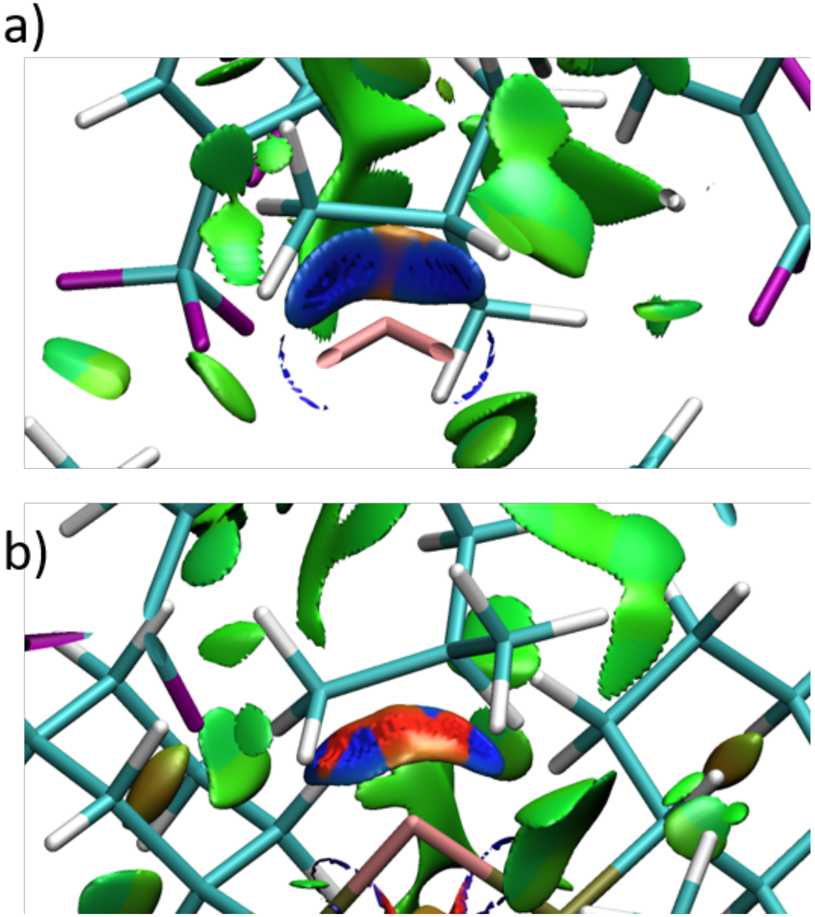

Figure S112: a) NCI Plot of **[1-isobutane][BAr$^F{}_4$]** (fully optimised) with isosurfaces generated for s = 0.3 au and -0.07 < ρ < 0.07 au. View from Rh looking towards the alkane. **b)** View from above the alkane looking towards Rh.

Table S31: NBO data from **[1-isobutane][BAr$^F_4$]** (partially optimised) showing the donating and accepting orbitals occupation and the 2nd order perturbation theory interaction energy.

Donating Orbital	Occupation	Accepting Orbital	Occupation	Energy (kcal/mol)
		C1–H11		
σ (C–H)	1.86	σ* (Rh–P_{trans})	0.28	21.41
σ (C–H)	1.86	σ* (Rh–P_{Cis})	0.27	0.22
LP (Rh)	1.97	σ* (C–H)	0.03	0.05
LP (Rh)	1.90	σ* (C–H)	0.03	0.38
LP (Rh)	1.87	σ* (C–H)	0.03	3.40
σ (Rh–P_{cis})	1.83	σ* (C–H)	0.03	3.89
σ (Rh–P_{trans})	1.83	σ* (C–H)	0.03	1.63
		C2–H21		
σ (C–H)	1.89	σ* (Rh–P_{trans})	0.27	22.30
σ (C–H)	1.89	σ* (Rh–P_{Cis})	0.28	0.40
LP (Rh)	1.95	σ* (C–H)	0.05	0.44
LP (Rh)	1.90	σ* (C–H)	0.05	0.72
LP (Rh)	1.87	σ* (C–H)	0.05	3.80
σ (Rh–P_{cis})	1.83	σ* (C–H)	0.05	2.54
σ (Rh–P_{trans})	1.83	σ* (C–H)	0.05	0.69

.

Table S32: NBO data from **[1-isobutane][BAr$^F_4$]** (fully optimised) showing the donating and accepting orbitals occupation and the 2nd order perturbation theory interaction energy.

Donating Orbital	Occupation	Accepting Orbital	Occupation	Energy (kcal/mol)
		C1–H11		
σ (C–H)	1.86	σ* (Rh–P_{trans})	0.28	19.75
σ (C–H)	1.86	σ* (Rh–P_{Cis})	0.27	0.17
LP (Rh)	1.97	σ* (C–H)	0.05	0.07
LP (Rh)	1.90	σ* (C–H)	0.05	0.29
LP (Rh)	1.87	σ* (C–H)	0.05	2.92
σ (Rh–P_{cis})	1.83	σ* (C–H)	0.05	3.70
σ (Rh–P_{trans})	1.83	σ* (C–H)	0.05	1.69
		C2–H21		
σ (C–H)	1.88	σ* (Rh–P_{trans})	0.27	22.55
σ (C–H)	1.88	σ* (Rh–P_{Cis})	0.28	0.43
LP (Rh)	1.95	σ* (C–H)	0.05	0.67
LP (Rh)	1.90	σ* (C–H)	0.05	1.08
LP (Rh)	1.87	σ* (C–H)	0.05	3.69
σ (Rh–P_{cis})	1.83	σ* (C–H)	0.05	2.34
σ (Rh–P_{trans})	1.83	σ* (C–H)	0.05	0.70

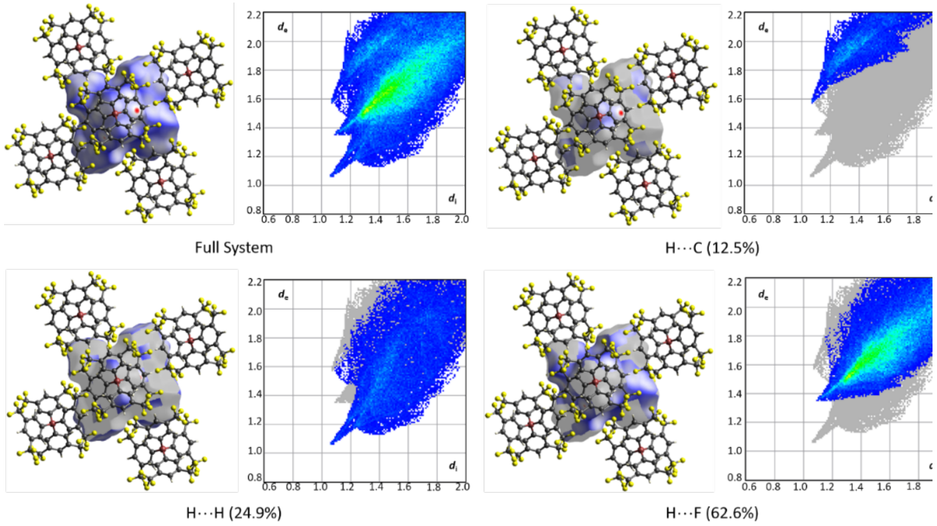

Figure S113: Hirshfeld Surfaces of **[1-isobutane][BAr^F$_4$]** (partially optimised) showing normalised distance and associated fingerprint plots. Percentage of each type of contact is shown in brackets.

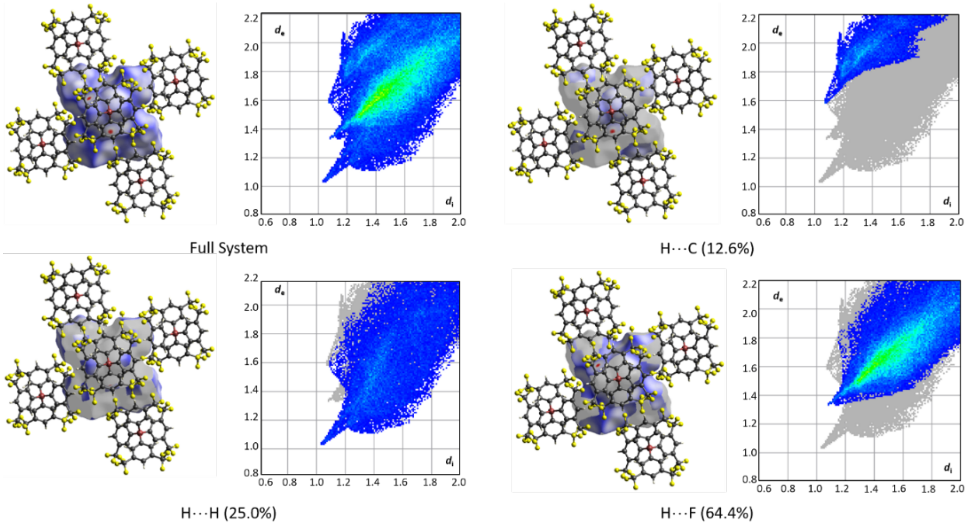

Figure S114: Hirshfeld Surfaces of **[1-isobutane][BAr^F$_4$]** (fully optimised) showing normalised distance and associated fingerprint plots. Percentage of each type of contact is shown in brackets.

S.6. References

S1 Chadwick, F. M.; Rees, N. H.; Weller, A. S.; Krämer, T.; Iannuzzi, M.; Macgregor, S. A. A Rhodium-Pentane Sigma-Alkane Complex: Characterization in the Solid State by Experimental and Computational Techniques. *Angew. Chem. Int. Ed.* **2016**, *55* (11), 3677–3681.

S2 Pike, S. D.; Chadwick, F. M.; Rees, N. H.; Scott, M. P.; Weller, A. S.; Krämer, T.; Macgregor, S. A. Solid-State Synthesis and Characterization of σ-Alkane Complexes, [Rh(L2)(H2,H2-C7H12)][BArF4] (L2 = Bidentate Chelating Phosphine). *J. Am. Chem. Soc.* **2015**, *137* (2), 820–833.

S3 Earl, W. L.; Vanderhart, D. L. Measurement Of13C Chemical Shifts in Solids. *J. Magn. Reson.* **1982**, *48* (1), 35–54.

S4 Lubben, A. T.; Scott McIndoe, J.; Weller, A. S. Coupling an Electrospray Ionwization Mass Spectrometer with a Glovebox: A Straightforward, Powerful, and Convenient Combination for Analysis of Air-Sensitive Organometallics. *Organometallics* **2008**, *27* (13), 3303–3306.

S5 Chadwick, F. M.; McKay, A. I.; Martinez-Martinez, A. J.; Rees, N. H.; Krämer, T.; Macgregor, S. A.; Weller, A. S. Solid-State Molecular Organometallic Chemistry. Single-Crystal to Single-Crystal Reactivity and Catalysis with Light Hydrocarbon Substrates. *Chem. Sci.* **2017**, *8* (9), 6014–6029.

S6 Nowell, H.; Barnett, S. A.; Christensen, K. E.; Teat, S. J.; Allan, D. R. I19, the Small-Molecule Single-Crystal Diffraction Beamline at Diamond Light Source. *J. Synchrotron Radiat.* **2012**, *19* (3), 435–441.

S7 McKay, A. I.; J. Bukvic, A.; E. Tegner, B.; L. Burnage, A.; J. Martínez-Martínez, A.; H. Rees, N.; A. Macgregor, S.; S. Weller, A. Room Temperature Acceptorless Alkane Dehydrogenation from Molecular σ-Alkane Complexes. *J. Am. Chem. Soc.* **2019**, *141* (29), 11700–11712.

S8 Benedict, J. B.; Coppens, P. Kinetics of the Single-Crystal to Single-Crystal Two-Photon Photodimerization of α-Trans-Cinnamic Acid to α-Truxillic Acid. *J. Phys. Chem. A* **2009**, *113* (13), 3116–3120.

S9 Hasegawa, K.; Asami, R.; Takahashi, K. 13C NMR Chemcial Shifts and Cationic Reactvity of Linear Conjugated Dienes. *Bull. Chem. Soc. Jpn.* **1978**, *51* (3), 916–920.

S10 (E)-3-methyl-1,3-pentadiene -- AIST: Spectral Database for Organic Compounds -- (SDBS No.: 293) -- Date Acc. 25-06-2020 https://sdbs.db.aist.go.jp/sdbs/cgi-bin/landingpage?sdbsno=293 (accessed Jun 25, 2020).

S11 (Z)-3-methyl-1,3-pentadiene -- AIST: Spectral Database for Organic Compounds -- (SDBS No.: 292) -- Date Acc. 25-06-2020 https://sdbs.db.aist.go.jp/sdbs/cgi-bin/landingpage?sdbsno=292 (accessed Jun 25, 2020).

S12 2-Ethyl-1-butene -- AIST: Spectral Database for Organic Compounds -- (SDBS No.: 10346) -- Date Acc. 03-06-2020 https://sdbs.db.aist.go.jp/sdbs/cgi-bin/landingpage?sdbsno=10346 (accessed Jun 3, 2020).

S13 3-Methyl-2-pentene, mixture of cis and trans -- Sigma-Aldrich -- (Cat. Number 111775) - Date Acc. 03-07-2020 https://www.sigmaaldrich.com/catalog/product/aldrich/111775?lang=en&region=GB (accessed Jul 3, 2020).

S14 3-Methyl-1-pentene -- Sigma-Aldrich -- (Cat. Number 111147) -- - Date Acc. 03-07-2020 https://www.sigmaaldrich.com/catalog/product/aldrich/111147?lang=en&region=GB (accessed Jul 3, 2020).

S15 *Oxford Diffraction Ltd.*; 2011.

S16 Sheldrick, G. M. A Short History of SHELX. *Acta Crystallogr. Sect. A Found. Crystallogr.* **2008**, *64* (1), 112–122.

S17 Sheldrick, G. M. SHELXT - Integrated Space-Group and Crystal-Structure Determination. *Acta*

Crystallogr. Sect. A Found. Crystallogr. **2015**, *71* (1), 3–8.

S18 Dolomanov, O. V.; Bourhis, L. J.; Gildea, R. J.; Howard, J. A. K.; Puschmann, H. OLEX2: A Complete Structure Solution, Refinement and Analysis Program. *J. Appl. Crystallogr.* **2009**, *42* (2), 339–341.

S19 Cosier, B. J.; Glazer, A. M. A Nitrogen-Gas-Stream Cryostat for General X-Ray Diffraction Studies. *J. Appl. Crystallogr.* **1986**, *19* (2), 105–107.

S20 Pike, S. D.; Thompson, A. L.; Algarra, A. G.; Apperley, D. C.; Macgregor, S. A.; Weller, A. S. Synthesis and Characterization of a Rhodium(I) σ-Alkane Complex in the Solid State. *Science* **2012**, *337*, 1648.

S21 McKay, A. I.; Krämer, T.; Rees, N. H.; Thompson, A. L.; Christensen, K. E.; Macgregor, S. A.; Weller, A. S. Formation of a σ-Alkane Complex and a Molecular Rearrangement in the Solid-State: [Rh(Cyp 2 PCH 2 CH 2 PCyp 2)(η 2 :η 2 -C 7 H 12)][BAr F 4]. *Organometallics* **2017**, *36* (1), 22–25.

S22 McKay, A. I.; Martínez-Martínez, A. J.; Griffiths, H. J.; Rees, N. H.; Waters, J. B.; Weller, A. S.; Krämer, T.; MacGregor, S. A. Controlling Structure and Reactivity in Cationic Solid-State Molecular Organometallic Systems Using Anion Templating. *Organometallics* **2018**, *37* (20), 3524–3532.

S23 Martínez-Martínez, A. J.; Tegner, B. E.; McKay, A. I.; Bukvic, A. J.; Rees, N. H.; Tizzard, G. J.; Coles, S. J.; Warren, M. R.; Macgregor, S. A.; Weller, A. S. Modulation of σ-Alkane Interactions in [Rh(L2)(Alkane)]+ Solid-State Molecular Organometallic (SMOM) Systems by Variation of the Chelating Phosphine and Alkane: Access to H2,H2-σ-Alkane Rh(I), H1-σ-Alkane Rh(III) Complexes, and Alkane Encapsulation. *J. Am. Chem. Soc.* **2018**, *140* (44), 14958–14970.

S24 Van de Vondele, J.; Krack, M.; Mohamed, F.; Parrinello, M.; Chassaing, T.; Hutter, J. *Comput. Phys. Commun.* **2005**, 167, 103–128.

S25 Hutter, J.; Iannuzzi, M.; Schiffmann, F.; VandeVondele, J. *Wires Comput. Mol. Sci.* **2014**, *4*, 15.

S26 VandeVondele, J.; Hutter, J. *J. Chem. Phys.* **2007**, *127*, 114105.

S27 Hartwigsen, C.; Goedecker, S.; Hutter, J. *Phys. Rev. B* **1998**, *58*, 3641.

S28 Goedecker, S.; Teter, M.; Hutter, J. *Phys. Rev. B* **1996**, *54*, 1703.

S29 Krack, M. *Theor. Chem. Acc.* **2005**, *114*, 145.

S30 Perdew, J. P.; Burke, K.; Ernzerhof, M. *Phys. Rev. Lett.* **1996**, *77*, 3865.

S31 Grimme, S.; Antony, J.; Ehrlich, S.; Krieg, H. *J. Chem. Phys.* **2010**, *132*, 154104.

S32 Mercury CSD 2.0, Macrae, C. F.; Bruno, I. J.; Chisholm, J. A.; Edgington, P. R.; McCabe, P.; Pidcock, E.; Rodriguez-Monge, L.; Taylor, R.; van de Streek, J.; Wood, P. A. *J. Appl. Cryst.,* **2008**, *41*, 466-470.

S33 Ghysels, A.; Verstraelen, T.; Hemelsoet, K.; Waroquier, M.; Van Speybroeck, V. *J. Chem. Inf. Model.* **2010**, 50, 1736-1750.

S34 VESTA 3, K. Momma and F. Izumi, *J. Appl. Cryst.* **2011**, 44, 1272-1276.

S35 CrystalExplorer 17, M. J. Turner, J. J. McKinnon, S. K. Wolff, D. J. Grimwood, P. R. Spackman, D. Jayatilaka and M. A. Spackman, **2017**, University of Western Australia.

S36 Gaussian 09 (Revision D.01); Frisch, M. J.; Trucks, G. W.; Schlegel, H. B.; Scuseria, G. E.; Robb, M. A.; Cheeseman, J. R.; Scalmani, G.; Barone, V.; Mennucci, B.; Petersson, G. A.; Nakatsuji, H.; Caricato, M.; Li, X.; Hratchian, H. P.; Izmaylov, A. F.; Bloino, J.; Zheng, G.; Sonnenberg, J. L.; Hada, M.; Ehara, M.; Toyota, K.; Fukuda, R.; Hasegawa, J.; Ishida, M.; Nakajima, T.; Honda, Y.; Kitao, O.; Nakai, H.; Vreven, T.; J. A. Montgomery, J.; Peralta, J. E.; Ogliaro, F.; Bearpark, M.; Heyd, J. J.; Brothers, E.; Kudin, K. N.; Staroverov, V. N.; Keith, T.; Kobayashi, R.; Normand, J.; Raghavachari, K.; Rendell, A.; Burant, J. C.; Iyengar, S. S.; Tomasi, J.; Cossi, M.; Rega, N.; Millam, J. M.; Klene, M.; Knox, J. E.; Cross, J. B.; Bakken, V.; Adamo, C.; Jaramillo, J.; Gomperts, R.; Stratmann, R. E.; Yazyev, O.; Austin, A. J.; Cammi, R.; Pomelli, C.; Ochterski, J. W.; Martin, R. L.; Morokuma, K.; Zakrzewski, V. G.; Voth, G. A.; Salvador, P.; Dannenberg, J. J.; Dapprich, S.; Daniels, A. D.; Farkas, O.; Foresman, J. B.; Ortiz, J. V.; Cioslowski, J.; Fox, D. J.; Gaussian Inc., Wallingford, CT, 2013.

S37 Becke, A. D. *Phys. Rev. A* **1988**, *38*, 3098.

S38 Perdew, J. P. *Phys. Rev. B* **1986**, *33*, 8822.

S39 Andrae, D.; Haussermann, U.; Dolg, M.; Stoll, H.; Preuss, H. *Theor. Chim. Acta* **1990**, *77*, 123.

S40 Hollwarth, A.; Bohme, M.; Dapprich, S.; Ehlers, A. W.; Gobbi, A.; Jonas, V.; Kohler, K. F.; Stegmann, R.; Veldkamp, A.; Frenking, G. *Chem. Phys. Lett.* **1993**, *208*, 237.

S41 Hehre, W. J.; Ditchfield, R.; Pople, J. A. *J. Chem. Phys.* **1972**, *56*, 2257.

S42 Hariharan, P. C.; Pople, J. A. *Theor. Chim. Acta* **1973**, *28*, 213.

S43 Bader, R. F. W. *Atoms in Molecules: A Quantum Theory*; Oxford University Press, 1990.

S44 AIMAll (Version 17.11.14), Todd A. Keith, TK Gristmill Software, Overland Park KS, USA, 2017 (aim.tkgristmill.com)

S45 NBO 6.0. E. D. Glendening, J. K. Badenhoop, A. E. Reed, J. E. Carpenter, J. A. Bohmann, C. M. Morales, C. R. Landis, and F. Weinhold (Theoretical Chemistry Institute, University of Wisconsin, Madison, WI, 2013); http://nbo6.chem.wisc.edu/

S46 E. R. Johnson, S. Keinan, P. Mori-Sanchez, J. Contreras-Garcia, A. J. Cohen, and W. Yang, *J. Am. Chem. Soc.*, **2010**, *132*, 6498-6506.

S47 J. Contreras-Garcia, E. R. Johnson, S. Keinan, R. Chaudret, J-P. Piquemal, D. N. Beratan, and W. Yang. *J. Chem. Theory Comput.* **2011**, *7*, 625-632.

S48 Chemcraft - graphical software for visualization of quantum chemistry computations. https://www.chemcraftprog.com
